# Genomic Assembly of Clinical Candida glabrata (Nakaseomyces glabrata) Isolates Reveals within-Species Structural Plasticity and Association with *In Vitro* Antifungal Susceptibility

**DOI:** 10.1128/spectrum.01827-22

**Published:** 2022-11-10

**Authors:** Irene Stefanini, Emily Stoakes, Houdini H. T. Wu, Li Xu-McCrae, Abid Hussain, John Moat, Christopher G. Dowson, Miruna D. David, Chrystala Constantinidou

**Affiliations:** a Department of Life Sciences and Systems Biology, University of Turin, Turin, Italy; b Warwick Medical School, University of Warwick, Coventry, United Kingdom; c UK Health Security Agency Public Health Laboratory, Birmingham, United Kingdom; d School of Life Sciences, University of Warwick, Coventry, United Kingdom; e University Hospitals Birmingham NHS Foundation Trust, Birmingham, United Kingdom; University of Guelph

**Keywords:** *Candida glabrata*, antifungals, antimicrobial resistance, genomics, population genetics

## Abstract

The opportunistic human pathogen Candida glabrata has become an increasingly important threat to human health, with infections globally characterized by high mortality rates and multidrug resistance. To face this threat, more efficient diagnostic and therapeutic approaches are required, underpinning research to help define the intraspecies epidemiology, genetic variability, and therefore, diagnostic and therapeutic target stability. Previous comparative genetics studies conducted on limited numbers of strains only revealed partial resolution of chromosomal settings. In this study, by combining short- and long-read genome sequencing, phenotypic characterization, and comparative genomics over a large set of strains, we detected strict relationships between large chromosomal rearrangements and phylogenetic clades, genes subjected to different selective pressures, and new sets of genes associated with resistance to antifungals. Overall, these results not only provide a fundamental contribution to our knowledge of C. glabrata evolution and epidemiology but may also lay the foundations for the future development of tailored therapeutic approaches.

**IMPORTANCE** The human pathogen Candida glabrata has become a global threat to human health, with infections characterized by high mortality and multidrug resistance. We have obtained nine fully assembled genomes from clinical isolates through a combination of short- and long-read sequencing approaches. The quality and completeness of such genomes and their subsequent comparison to the broadest set of genomes so far allowed us to pinpoint chromosomal rearrangements in several genomes and detect phylogenetic clades that were not associated with geographic location or isolation source. We identified a new set of genes associated with resistance to antifungals coding for adhesin or adhesin-like proteins, suggesting C. glabrata resists antifungals by forming aggregates or adhering to the host tissue. These results, which provide a fundamental contribution to our knowledge of C. glabrata evolution and epidemiology, may initiate the development of precision medicine interventions for patients with suspected or proven invasive fungal infections.

## INTRODUCTION

The opportunistic human pathogen Candida glabrata is the third most common cause, after Candida albicans and Candida parapsilosis, of candidiasis in the United Kingdom and many worldwide and European countries (1, 2). C. glabrata infections can colonize the gastrointestinal and urogenital tracts, as well as other body sites, and result in systemic infections such as candidemia and meningitis (3). As the diagnosis of systemic *Candida* infections is both clinically and technically challenging, especially in immunocompromised hosts, these infections are associated with high mortality, partly due to late or ineffective administration of empirically chosen antifungal treatments (4). The first line of treatment for C. glabrata infections (echinocandins and azoles) is increasingly becoming ineffective due to the emergence of resistance, which has been evaluated to proceed at higher rates compared to that in other species of the same genus (5). To face this threat, current efforts are aimed at monitoring the spread and increase of non-*albicans Candida* infections and their antifungal resistance, improving rapid laboratory diagnostics for organism identification and antifungal susceptibility, and facilitating treatments of higher efficacy, all of which contribute to improved patient outcomes (6). An efficient and globally shared approach for the identification of C. glabrata infective strains is lacking, with the most recent detailed global epidemiological report on C. glabrata infections dating back to 1999 (7); epidemiological studies are currently only carried out at the national level. However, defining a shared protocol for multilocus sequence typing (MLST) (8) and generating a curated database of C. glabrata sequence types (STs) (9) will help promote worldwide tracking of C. glabrata strain spread. Determining the level of genomic variability in the C. glabrata species is crucial in this process, as it would allow the identification of genomic features suitable for typing pathogenic strains and of genetic characteristics associated with the pathogenicity and development of antifungal resistance. Genomic analyses carried out so far on this species have shown that C. glabrata is phylogenetically closer to the GRAS (generally recognized as safe) yeast Saccharomyces cerevisiae than to other species of the *Candida* genus, suggesting different paths for the acquisition of virulence capabilities (10). Like S. cerevisiae, C. glabrata does not show genetic or phenotypic features indicating ecological niches to which the species could have adapted. Hence, the current consideration of C. glabrata as a commensal of humans has been called into question (11). C. glabrata genomic analyses have also highlighted frequent chromosomal aneuploidies and rearrangements, as also observed in C. albicans strains, and high levels of interclade genetic diversity with a higher frequency of genomic recombinations between clades than C. albicans. Also, genomic analyses did not confirm the relationship between genetic diversity and geographical distribution previously observed through MLST analyses (12). Despite these recent observations on C. glabrata genomics that suggest a lack of associations between evolution and geographical origin, pathogenic potential, or antifungal susceptibility, it must be considered that most of the studies have been carried out on limited sets of strains, usually isolated from a few geographical locations. A comprehensive comparison of the variants and genome structures of C. glabrata strains isolated from all around the world has yet to be carried out, but it would greatly improve our understanding of the evolution of antifungal susceptibility and pathogenicity.

To better assess the structural genomic and nucleotide variations within the C. glabrata species and their impact on relevant phenotypes, we have analyzed 30 C. glabrata strains isolated from 2 Birmingham, United Kingdom, hospitals and combined short- and long-read (Illumina and Nanopore, respectively) sequencing approaches and classical assays to evaluate the susceptibility of these clinical strains to commonly prescribed antifungals. The fully assembled genomes obtained in this study were subsequently instrumental in carrying out a large-scale comparison with 229 publicly available C. glabrata genomes. The analysis allowed for the identification of chromosomal rearrangements shared among multiple strains and of new genes potentially associated with resistance to the antifungals commonly used in the clinic.

## RESULTS AND DISCUSSION

### Genome assembly highlights severe chromosomal rearrangements in the genome of clinical Candida glabrata strains.

**FIG 1:**
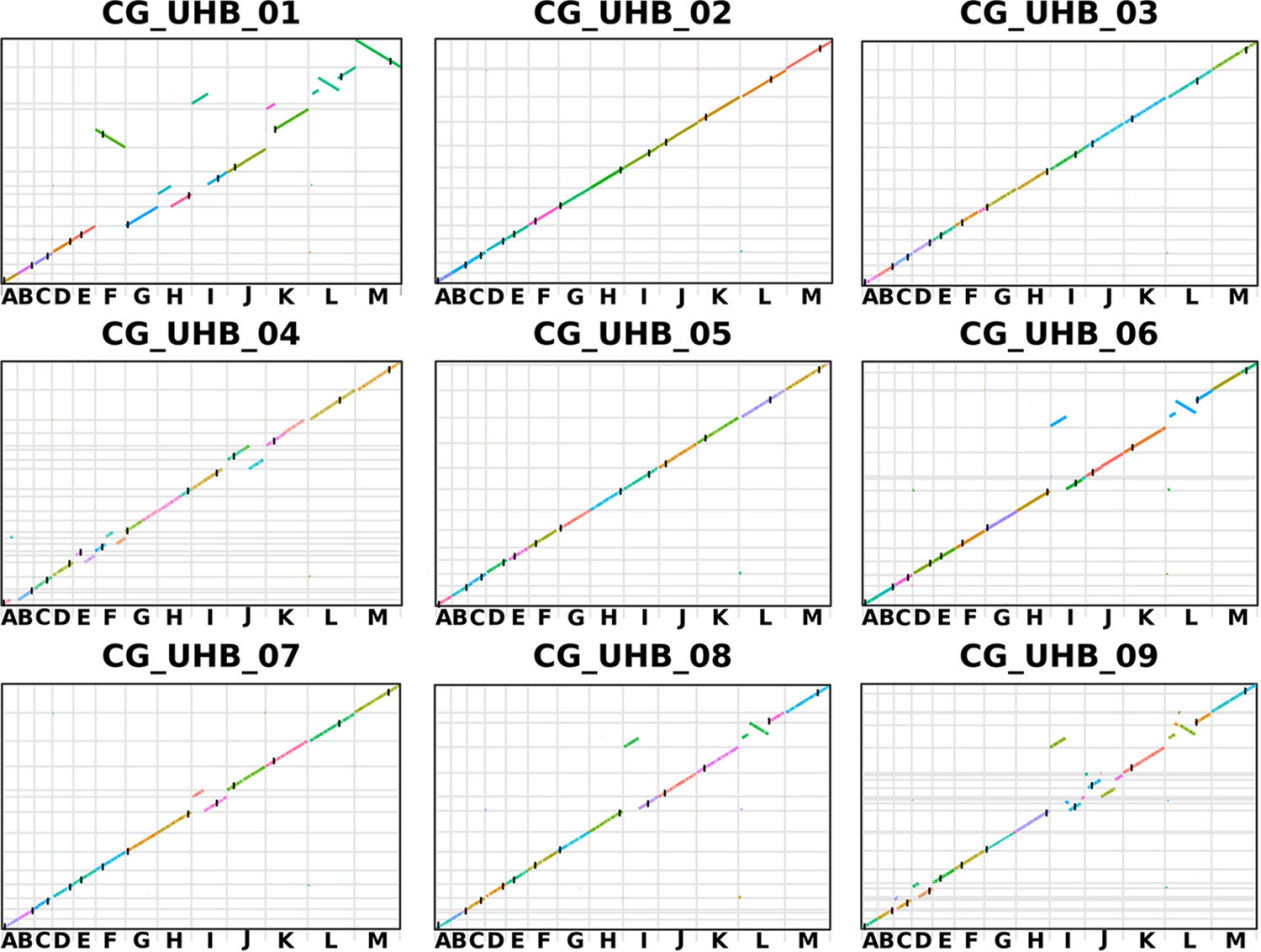
Synteny plots of the nine newly assembled genomes of Candida glabrata strains isolated during this study. Vertical black lines indicate centromeres (as located in the reference genome). For each strain genome, lines of different colors indicate different contigs.

The genomes of nine C. glabrata strains (CG_UHB_01 to -09) isolated from various clinical samples collected at the University Hospitals Birmingham NHS Trust were obtained through a combination of long-read Nanopore and short-read Illumina sequencing (see Table S1 in the supplemental material). The combination of the two approaches, for most of the isolates, allowed us to reconstruct entire chromosomes. We obtained a minimum of 14 contigs (for strains CG_UHB_01 and CG_UHB_07) to a maximum of 27 contigs (for the CG_UHB_09 strain), comparable to the number of chromosomes of the reference C. glabrata strain (CBS138), encompassing 13 nuclear chromosomes and the mitochondrial chromosome. The size of the assembled genomes ranged between 11.8 Mb (strain CG_UHB_04, 23 contigs) and 12.6 Mb (strains CG_UHB_01 and CG_UHB_02) (Table S1), similar to the size of the reference genome (12.3 Mb). Synteny plots and Assemblytics (13) analysis revealed severe chromosomal rearrangements in the sequenced strains, mostly affecting chromosomes L and I (Fig. 1 and Data Set S1). Four out of the nine newly assembled genomes (CG_UHB_01, CG_UHB_06, CG_UHB_08, and CG_UHB_09) showed a paracentric inversion within the sequence of chromosome L and a nonreciprocal translocation of the extremity of the left arm of chromosome I to the 5′ end of the left arm of chromosome L (Fig. 1). Another large chromosomal rearrangement was observed in the CG_UHB_01 strain: the fusion of chromosomes F and K (Fig. 1). However, an inspection of Illumina reads mapping against the potential chromosomal fusion showed low coverage in this region, suggesting an inaccurate assembly (Fig. S1). The comparison of the Canu and Minimap2 assemblies of the new genomes confirmed the rearrangements for most genomes (Fig. S2). We took advantage of the approach proposed in the software STAR, commonly used to pinpoint intergenic splice junctions from transcriptomics data (14), to detect reads mapping partly against a region of a chromosome and partly against a distant part of the same chromosome or another chromosome. The STAR analysis allowed us to confirm the absence of the fusion of chromosomes F and K in strain CG_UHB_01while confirming the presence of the other observed chromosomal rearrangements (Data set S2). STAR analysis carried out on Illumina reads highlighted that in all the strains whose genome assembly showed the presence of an inversion of part of chromosome L, the inverted region encompassed the chromosomal portion from 308,989 to 939,613 of the reference genome. Notably, none of these chromosomal rearrangements disrupted coding regions. The coordinates of both the beginning and the end of the inverted region in chromosome L were located in intergenic regions. Position 308,989 is located between the CAGL0L02607g and the CAGL0L02629g genes. The former expresses a protein (XP_448884.1) which has domains with predicted hydrolase activity and a role in the nucleotide catabolic process and the latter gene is similar to *Saccharomyces cerevisiae CDC4* cell division control gene (Fig. S3a). The end of the inverted region at position 939,613 is located downstream to the gene CAGL0L08602g that expresses a protein (XP_449150.1) similar to *S. cerevisiae PPX*, orthologs of which have exopolyphosphatase activity, a role in the polyphosphate catabolic process, and cytoplasm localization (Fig. S3b). Similarly, the translocation of the 5′ extremity of the left arm of chromosome I to the 5′ extremity of the left arm of chromosome L, for every strain bearing this translocation, ranged from the extremity to position 489,298W of the reference strain chromosome I. Even in this case, the rearrangement did not corrupt any coding regions, as it was located within an intergenic region, between the CAGL0I05148g gene (similar to S. cerevisiae *DLD1* d-lactate ferricytochrome C oxidoreductase; 486,625W to 488,349W) and the CAGL0I05170g gene (XP_447475.1; some similarities with S. cerevisiae *CST6* ATF/CREB activator; 490,959W to 493,064W) (Fig. S3c). We assessed the presence of the rearrangements in 21 additional strains isolated during this study and in 229 strains whose genomes were sequenced through an Illumina approach in previous studies (Table S1). A total of 20 strains showed the inversion of chromosome L (Table S2). Interestingly, 133 out of 229 strains whose Illumina reads were available from previous studies showed the inversion in chromosome L, a rearrangement that was not highlighted previously. The presence of chromosomal rearrangements (Table S3) did not correlate with the country of isolation (Table S1, results in Fig. S4, Chi-square *P* = 0.3388), with the year of isolation (Fig. S4, Chi-square *P* = 0.194), or with the isolation source (Fig. S4, Chi-square *P* = 0.991). The comparison with three recently released C. glabrata genome assemblies (15, 16) confirmed the presence of the observed rearrangements also in the two additional clinical isolates BG2 and BG3993 (16) (Fig. S5). In addition, the comparison with the recently assembled genomes of CBS138, BG2, and BG3993 highlighted the presence of subtelomeric rearrangements in most of our newly assembled genomes (Fig. S5).

### Genomic variability of C. glabrata strains reveals hints on evolution.

**FIG 2:**
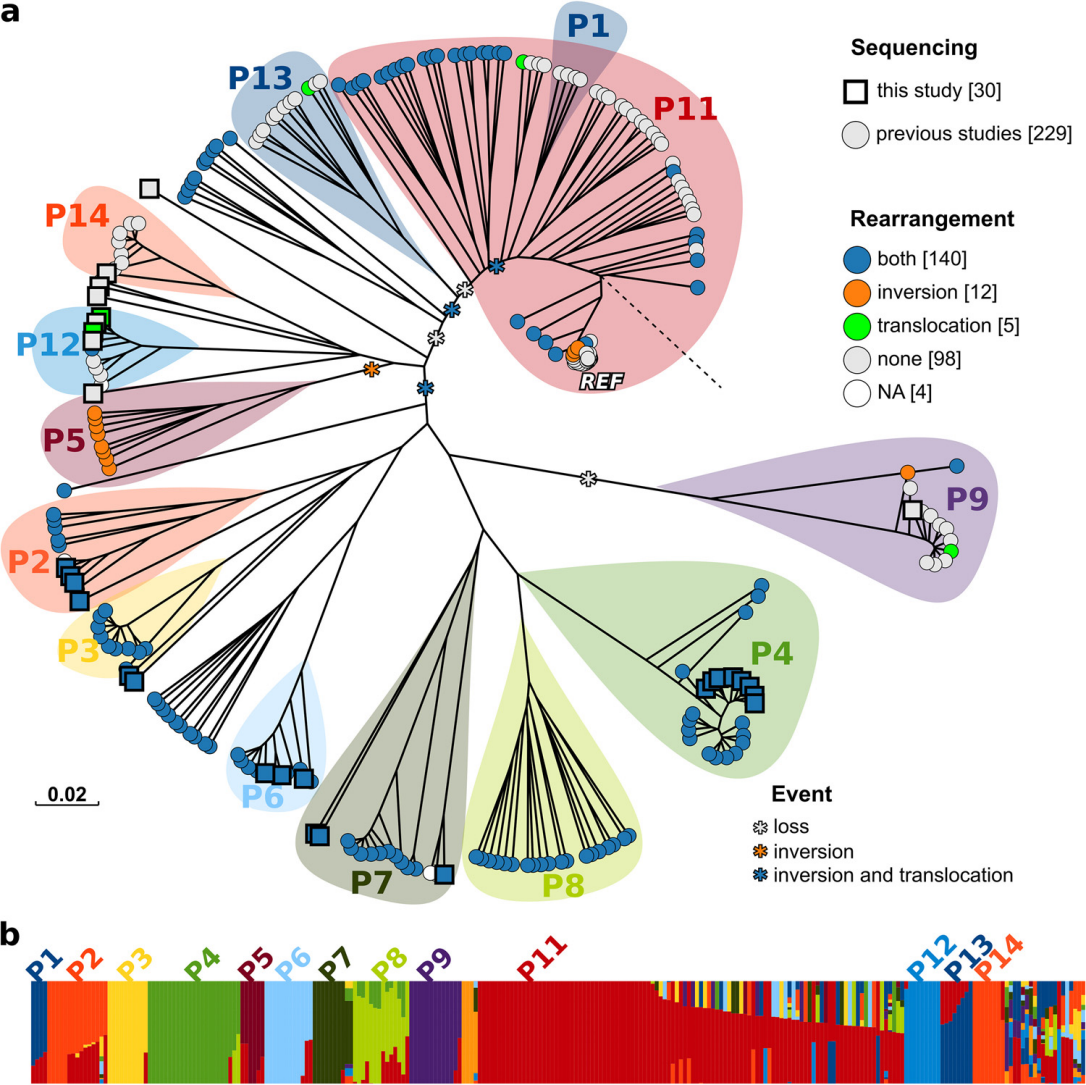
Phylogenetic tree of C. glabrata genomes and populations (P) inferred with fastSTRUCTURE. (a) Distances among genomes were calculated on genetic variants of the strains (SNPs, indels). Each point, colored according to the presence/absence of the chromosomal rearrangements as reported in the legend, indicates a different strain. The dotted black line indicates the root of the tree (S. cerevisiae). Translocation, nonreciprocal translocation of the left arm of chromosome I to chromosome L; inversion, paracentric inversion within the sequence of chromosome L; both, strain bearing both the translocation and the inversion; none, strain bearing neither the inversion nor the translocation; NA, impossible to determine the presence of chromosomal rearrangements with the available sequencing data. Colored areas show the strains clustering as inferred through fastSTRUCTURE analysis and reported in (panel b). (b) Ancestry analysis based on genomic variants and inferred with fastSTRUCTURE (18).

The relationship between the chromosomal rearrangement and evolution of the C. glabrata species was assessed. This was investigated by reviewing the genomic variations identified by aligning the Illumina reads against the reference genome (strain CBS138, assembly ASM254v2 downloaded from NCBI in January 2021) (Table S1). The phylogenetic tree obtained on single-nucleotide polymorphisms (SNPs) and indels found across the entire set of analyzed genomes (Fig. 2a) was supported by bootstrap analysis (Fig. S6), and the phylogeny was fairly consistent when comparing the neighbor-joining and maximum likelihood-based clusterings (Fig. S7). The phylogenetic analysis based on whole-genome variations and fastSTRUCTURE analysis revealed the best partition of genomes into 14 clades (Fig. 2b). This was defined as the minimum number of clusters associated with the highest likelihood and maximizing the ΔK (17), an *ad hoc* quantity related to the second-order rate of change of the log probability of data compared to the number of clusters) (Fig. S8). To assess the ancestral clades (the closest clades to an outgroup species), we inferred the phylogeny of the entire set of C. glabrata strains and the reference S. cerevisiae strain (S288c) based on the variations in four genes previously identified as sufficient to recapitulate the genetic divergence within the Ascomycota phylum (18): CAGL0M13409g (YHR186C), CAGL0M07722g (YMR012W), CAGL0J01111g (YJL029C), and CAGL0G00726g (YAR007C) (Fig. S9). For every genome sequenced during this study, the copy number of these genes was higher than the average copy number of the rest of the genes of the same genomes, (which could compromise the reliability of clustering due to the number of paralogs present. However, the clustering based on the comparison of these genes, and including S. cerevisiae as the root (Fig. S10), showed the same clades observed in the phylogenetic tree based on the entire set of genomic variants (Fig. 2), supporting the reliability of the clustering based on these marker genes. The phylogenetic analysis based on these selected variants allowed us to observe that, among these clades, the clade closest to the tree root (as defined by the position of the out-group S. cerevisiae [Fig. S9], the tree annotated with bootstrap values is in Fig. S10) encompassed the C. glabrata reference strain (“REF” in Fig. 2a), together with a few additional strains which evolved under various environmental stresses (19). It is worth noting that some strains (those not included in the highlighted clusters in Fig. 2a) could not be clearly assigned to any of the identified clades, as they were inferred to have descended from multiple ancestors (Fig. 2b). Interestingly, this situation, referred to as mosaicism, is usually considered the result of mating events among strains descending from different ancestors, hence supporting the recently proposed hypothesis that C. glabrata can actually mate (11). Whereas the genomic tree did not highlight the grouping of strains according to isolation source, country of isolation, or year of collection (Fig. S11), a clear clustering according to the presence/absence of the chromosomal rearrangements was present (Chi-square *P* < 0.01, Fig. 2). The number of genetic variants present in the chromosomal regions involved in the rearrangements was significantly higher in genomes bearing the rearrangements than in the same portions of genomes not bearing the rearrangements (Fig. S12). However, the phylogenetic trees drawn on distances calculated on all the genomic variations except those located in the rearranged regions differed only slightly from the phylogenetic tree based on the entire set of genomic variations (Fig. S13). These observations suggest that the insurgence of the observed chromosomal rearrangements is associated with C. glabrata evolution. Despite the other clusters of strains showing a clear distribution according to the presence/absence of the chromosomal rearrangements, there was no obvious evolution pattern. Instead, several nodes grouping strains with either translocation, the inversion, or both, were evident (highlighted with asterisks in Fig. 2). Three strains that evolved under environmental stresses from the CBS138 reference strain (ATCC 2001) bore the inversion in chromosome L (orange points in the REF cluster of Fig. 2). In particular, the strains subjected to a periodic challenge with 80 mM hydrogen peroxide for 1 h and two strains independently subjected to a periodic challenge at 47°C for 30 min (two out of three independent biological replicates were subjected to this environment) showed the inversion within chromosome L. Furthermore, three strains belonging to the REF cluster bore both the translocation and the inversion: F1822_CANGA, a strain isolated from blood in North America (12), and two strains sequenced at Cornell University in the same study—Y648, isolated from blood in Viborg, Denmark (ERR1938065) (20), and the reference strain CBS138 (ERR1938089) (public data, unpublished work). It has to be considered, however, that the CBS138 strain genome ERR1938089 showed a relatively higher number of variants (724 SNPs/indels) for a genome resequence, hence suggesting that the strain sequenced in that study had diverged from the original reference strain sequenced in other studies included in this comparison (15, 20).

Previous reports indicated that aneuploidies are frequent in the species and are involved in virulence (20). Conversely, coverage analysis carried out in our study revealed that only strain CG_UHB_14 showed aneuploidy, with chromosome E showing a higher coverage than the other chromosomes of the same strain (NC_006028.2) (Mann-Whitney false-discovery rate [FDR] < 0.05, Fig. S14).

For each newly sequenced genome, genes were predicted with AUGUSTUS and annotated through the best match finding with blastn and blastp (Data Set S3). A total of 28 genes (Table S3) showed a ratio of nonsynonymous to synonymous evolutionary changes (*dN*/*dS* ratio) (full results in Data Set S4) equal to 0 in the set of C. glabrata strains isolated in this study, meaning that none of the corresponding sequences bore nonsynonymous mutations. Of these 28 genes with a *dN*/*dS* ratio of 0, 19 also showed nonsynonymous variants in at least one of the strains whose genomes were sequenced previously (Table S3). Only 2 among these 19 genes did not bear any genetic variants (not even synonymous variants): CAGL0E04752g and CAGL0F02255g. The remaining 17 genes did carry at least 1 synonymous mutation in at least 1 genome out of the 30 sequenced here. Nine of the genes with a *dN*/*dS* ratio of 0 (CAGL0A04521g, CAGL0B04345g, CAGL0D00440g, CAGL0E04752g, CAGL0F02673g, CAGL0H08327g, CAGL0J03212g, CAGL0K08140g, and CAGL0M12386g) were highly conserved in all the strains whose sequence is currently available. Out of these nine genes, four were essential in S. cerevisiae, corresponding to 44% of the highly conserved C. glabrata genes (Table S3). This is an interesting aspect, especially considering that only 11.67% of C. glabrata genes with an ortholog in S. cerevisiae are essential (514 out of 4,407). The remaining five C. glabrata highly conserved genes do not appear to have a corresponding ortholog in the S. cerevisiae essential genes. These genes in S. cerevisiae have either paralog genes or encode proteins involved in metabolic processes. This observation suggests two different scenarios indicating the path leading to the high gene conservation observed in C. glabrata. In the first case, the presence of paralog genes in S. cerevisiae may have masked the fact that these genes are involved in essential functions, which could explain the high conservation in C. glabrata. In the other case, the high conservation of genes involved in metabolic processes may suggest that these genes are essential only for C. glabrata (not for S. cerevisiae), possibly because of the different metabolic features of the two species. In particular, the gene encoding a triosephosphate isomerase fundamental for glycolysis (CAGL0H08327g, ortholog of the *TPI1* S. cerevisiae gene) and the gene encoding a mitochondrial aldehyde dehydrogenase involved in regulation or biosynthesis of electron transport chain components and acetate formation (CAGL0J03212g, ortholog of the *ALD5* S. cerevisiae gene) were conserved in C. glabrata and not essential in S. cerevisiae. The roles of the proteins encoded by these two genes would suggest that C. glabrata strains strongly rely on aerobic metabolism, unlike the phylogenetically close S. cerevisiae, which can easily switch among the two metabolisms (21).

Interestingly, we observed that 19 genes showing a *dN*/*dS* ratio equal to 0 in the 30 strains isolated and sequenced in this study bear nonsynonymous mutations in previously sequenced strains. Thus, these genes were highly conserved only in the subset of strains isolated in this study (Table S3). Considering that the strains sequenced here were not inferred to belong to a single clade (Fig. 2), this information may indicate that these genes are relevant in the studied environment, rather than that they are conserved in a genetically similar set of strains. However, the genes were not conserved in previously sequenced strains isolated from similar sources (clinical specimens such as blood, fluid, and mouth), hence not supporting the hypothesis that the associated functions are fundamental in such an environment. It is worth mentioning that only one of the clinical strains previously sequenced was isolated in the United Kingdom, as opposed to every strain sequenced in this study (Table S1). This may suggest a geographically driven different selection of the 19 genes highly conserved or population drift, a hypothesis that needs to be further assessed by expanding the set of sequenced UK strain genomes.

The *dN*/*dS* analysis also highlighted 5 genes with a *dN*/*dS* ratio higher than 1 (Table S3), suggesting that the conservation of the sequence of these genes is not crucial in the set of strains under investigation. Two (CAGL0I04136g and CAGL0L08068g) of these five genes have an ortholog in S. cerevisiae (*MTC3* and *RIM1*, respectively), and interestingly, they are predicted to code proteins involved in mitochondrial functions. This may further indicate relevant divergences between C. glabrata and S. cerevisiae metabolism; whereas C. glabrata may strongly rely on highly efficient mitochondrial functions, S. cerevisiae may withstand a less efficient aerobic metabolism, as it preferentially ferments most carbon sources. Delving into the efficiency of the products of these genes is necessary to further disclose the metabolic differences between these two species.

### Antimicrobial resistance.

**FIG 3:**
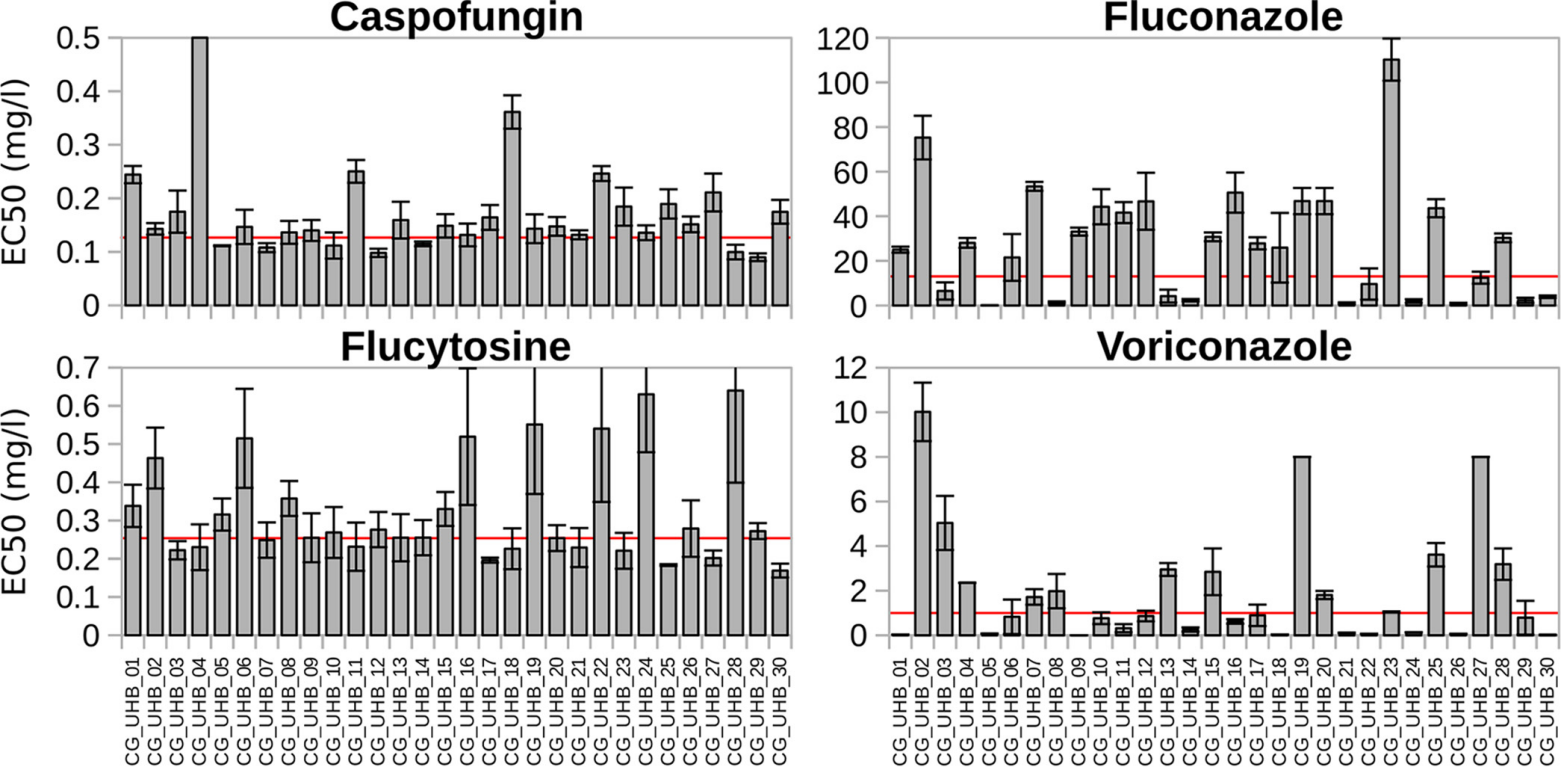
MICs of each tested antifungal drug against Candida glabrata clinical strains isolated during this study. Red horizontal lines indicate the clinical breakpoints as reported in Materials and Methods.

Considering the growing global concern related to the spread of antifungal resistance among clinical isolates, we evaluated the susceptibility of the strains isolated in this study against a set of antifungals (fluconazole, voriconazole, caspofungin, and flucytosine). Every isolate was susceptible to at least one of the tested antifungals. In addition, no isolate was susceptible to fluconazole; according to the EUCAST clinical breakpoints (v9.0) (22), 12 of the isolates showed an intermediate response, and the other 18 strains were resistant (Fig. 3). A total of 13 strains were susceptible and 17 strains were resistant to voriconazole; 11 strains were susceptible and 19 were resistant to flucytosine. When susceptibility to caspofungin was measured, only 1 strain was resistant (CG_UHB_04, also resistant to fluconazole and voriconazole but susceptible to flucytosine), 7 strains were susceptible, and 22 strains showed intermediate MICs (Fig. 3).

Previous studies have identified genetic variants in 14 genes (*CDR1*, *ERG9*, *ERG11*, *FKS1*, *FKS2*, *FKS3*, *FCY1*, *FCY2*, *FLR1*, *FPS1*, *FPS2*, *FUR1*, *PDR1*, and *SNQ2*) as associated with the resistance to classes of antifungals (azoles, echinocandin, and flucytosine). We could not find nonsynonymous mutations in the *FUR1* and *FCY1* genes. Even though nonsynonymous mutations were found in the other genes, the vast majority of these were not consistently associated with either resistance or susceptibility to the tested antifungals. The exceptions to this were the Arg137Leu mutation in the *FCY2* gene, Phe659Ser in the *FKS2* gene, Leu935Ser in the *PDR1* gene, and the frameshift mutation (also introducing a stop codon) in the *SNQ2* gene, all of which were found only in the unique strain resistant to caspofungin (Table S4).

To identify other potential correlations between the genetic features of the strains and their antifungal susceptibility, we carried out a genome-wide association study (GWAS) on the 30 strains isolated and whose genome was sequenced over this study (quantile-quantile [QQ] and Manhattan plots in Fig. S15). This analysis revealed some genetic variants significantly associated with the response to the tested antifungals (Table S5). Of note, none of the nonsynonymous SNPs identified as being associated with the response to the tested antifungals were located in the coding DNA sequences (CDS) of the genes previously known to be associated with the response to the antifungal, suggesting the acquisition of new mechanisms of resistance. A total of 41 nonsynonymous SNPs in 28 genes (Fig. S16), 26 nonsynonymous SNPs in 19 genes (Fig. S17), 96 nonsynonymous SNPs in 79 genes (Fig. S18), and 11 nonsynonymous SNPs in 8 genes (Fig. S19) were found to be associated with the response to caspofungin, fluconazole, voriconazole, and flucytosine, respectively. As expected, strains bearing multiple nonsynonymous SNPs found to be associated with the response to the tested antifungals were less susceptible to the antifungal (Pearson correlation *P* < 0.05; Fig. S20), suggesting a cumulative effect of the identified nonsynonymous SNPs. Pathway and Gene Ontology (GO) term enrichment analyses revealed an enrichment only for nonsynonymous SNPs associated with the response to caspofungin, which were enriched in genes involved in the yeast meiosis pathway (Table S5). However, it is interesting to note that several of the nonsynonymous SNPs found to be associated with the response to the tested antifungals were included in the CDS of genes coding adhesin or adhesin-like proteins (Table S5) or involved in the development of filamentous structures or biofilm: CAGL0H10626g, CAGL0E00187g, CAGL0J02508g, and CAGL0J02530g, associated with the response to caspofungin; CAGL0A01716g for fluconazole; CAGL0G10219g, CAGL0F08833g, and CAGL0G09361g for voriconazole; and CAGL0E00231g, CAGL0H00110g, CAGL0H10626g, CAGL0J01774g, and CAGL0J05159g for flucytosine (Table S5). This observation was particularly striking when considering the nonsynonymous SNPs associated with the response to flucytosine; despite the antifungal affecting targets involved in RNA and DNA biosynthesis, 8 of the 11 identified SNPs were located in genes coding adhesin or adhesin-like proteins. Such an observation is in line with previous reports on SNPs in other adhesin genes, *EPA1* (CAGL0E06644g), *EPA6* (CAGL0C00110g), *PWP2* (CAGL0I10246g), and *PWP5* (CAGL0I10340g), not found in this study, being associated with the response to echinocandins and azoles (23) and could indicate that C. glabrata strains can acquire resistance to multiple classes of antifungals by forming stronger aggregates between each other or adhering better to the host tissue, thus shielding the cells located in the core of the aggregate from the entry of the antifungal.

Considering the wide rearrangements observed for the newly sequenced genomes, we assessed whether such changes are associated with the acquisition of resistance toward the tested antifungals. Neither the presence of the inversion of part of chromosome L nor the nonreciprocal translocation of the left arm of chromosome I to chromosome L, nor the combination of them was significantly associated with either a higher resistance or susceptibility to the tested antifungals (Fig. S21).

**Conclusion.** As a result of the intersection of multiple sequencing approaches, we obtained fully assembled genomes, allowing us to identify and precisely locate large chromosomal rearrangements on the L chromosome of C. glabrata. By comparing 259 publicly available genomes of C. glabrata strains isolated from various specimens all over the world, which to our knowledge represents the broadest genomic comparison for this species to date, we detected a strong population structure, with 14 clades encompassing a large part of the entire population and not mimicking the geographical origin of the isolates previously observed (12). Our data revealed that some C. glabrata strains have mosaic genomes, a feature ascribed to microorganisms and mating among different lineages (24), hence supporting the recently proposed hypothesis that C. glabrata strains, unlike what previously believed, can mate (11). The observation of a neat association between the genomic clustering and the presence of the large chromosomal rearrangements not only supports the hypothesized plasticity of C. glabrata occurring at a fast rate but also highlights that the drastic rearrangements of chromosomal structures occur at a similar pace to smaller variations (e.g., short indels and SNPs).

Overall, these observations on genomic variants and structures also highlighted the fact that the strain commonly used as the reference for C. glabrata, CBS138, may not provide a good representation of the entire species. In fact, not only was CBS138 shown to be among the strains closest to the S. cerevisiae reference strain and hence not representing the broad genetic variability of the other C. glabrata cospecific strains, but it also bears atypical chromosome structures, with a large number (153 out of 259) of strains showing a different arrangement of the L chromosome. The identification of high or poor conservation of genes involved in aerobic metabolism, mostly not essential to the close S. cerevisiae species, may indicate that, despite C. glabrata being sometimes used as a starter for fermentation as the bakery yeast (25), S. cerevisiae may rely on a drastically different metabolism. Further in-depth and dedicated studies of the highlighted genes, potentially validating the current information by expanding the set of analyzed strains, comparison of paralog genes in other species, and assessment of the impact of mutations in these genes will contribute to the assessment of metabolic differences between C. glabrata and S. cerevisiae.

This study has identified new genes associated with resistance to antifungals. Despite all the preventative measures we adopted (e.g., correction/penalization based on the relatedness matrix and filtering of the genomic variants to remove tightly linked variants) which should prevent gross oversights, it is worth considering that the associations identified here should be confirmed on an independent set of strains, as the strains tested in this study could be representative of only a subset of C. glabrata strains. Furthermore, the experimental validation, through allele swapping (substituting the allele associated with resistance with an allele associated with susceptibility and vice-versa and then assessing changes in the response to the antifungal) would definitively confirm the findings. However, the fact that several of the genomic variants found to be associated with antifungal resistance were located in genes involved in processes required for adherence to surfaces (e.g., the host’s tissues) and in forming biofilms, together with the confutation of the previously proposed association of some key genes with antifungal resistance, suggests that C. glabrata may have some preferential paths for the acquisition of antifungal resistance. In particular, this may indicate that one of the major routes for the acquisition of resistance in C. glabrata (or, again, in our subset of strains) is the formation of biofilms, preventing the antifungal from accessing the shielded internal cells.

Fully appreciating the genomic variability and plasticity of C. glabrata is central to better understanding the evolutionary rate and triggers for this species. Data generated by this and subsequent studies could spearhead the discovery and adoption of new therapies. These agents, together with infection control, enshrined in a robust antifungal resistance strategy will be critical in limiting the spread and impact of these infections in the future.

## MATERIALS AND METHODS

### Sample collection and informed consent.

A total of 30 clinical isolates (CG_UHB_01 to CG_UHB_30) from sterile sites (e.g., blood cultures, ascetic fluid, etc.) with phenotypic antifungal resistance were selected from both the UK Health Security Agency, Public Health Laboratory (Birmingham) (UKHSA PHLB) (20) and UHB (10) sites from January 2015 to March 2017. Informed consent was not needed to work on pure microorganisms isolated in culture.

### Strain isolation and culturing.

The strains were isolated from routine sterile specimens using selective agar. They were then frozen as part of routine laboratory practice.

Frozen strains were maintained as 30% glycerol stocks at −80°C and maintained in culture on YPD (1% yeast extract, 2% Bacto-peptone, and 2% d-glucose) agar plates.

### DNA extraction.

For DNA extraction, 5 mL overnight culture in YPD was diluted to an optical density at 550 nm (OD_550_) of 0.05 in 150 mL YPD and grown to an OD_550_ of ~1.6. DNA was extracted using a Qiagen Genomic Tip 100/G according to the manufacturer’s instructions with isopropanol-precipitated DNA recovered by spooling to minimize shearing.

### Nanopore sequencing and genome assembly and analysis.

The clinical isolates whose genomes were sequenced in this study are listed in Table S1.

A total amount of 1 μg of isolated high-molecular-weight genomic DNA extract per strain was used for sequencing library preparation. Library preparation was performed using the rapid sequencing kit (SQK-RAD004) from Oxford Nanopore Technologies (ONT) with no size selection or shearing being applied. Nanopore sequencing was performed locally for 24 h on the MinION Mk1B sequencer using a primed SpotONFlow Cell Mk1 (R9.4) as per the manufacturer’s instructions. Reads were assembled using Minimap2 (26) and Canu (27). For each strain, the qualities of Minimap and Canu assemblies were compared, and the assemblies obtained with Minimap were chosen for further analyses because they showed the greatest *N*_50_ value and the lowest number of contigs (Table S1). If the *N*_50_ value and the number of contigs were similar among the two assemblies, the assembly resulting in the highest number of complete chromosomes, as observed with the synteny plots described below, was chosen. Draft genomes assemblies were improved by using Pilon software (28) to correct bases, fix misassemblies, and fill gaps based on the Illumina reads obtained as described below. Genes were predicted on the resulting corrected contigs with AUGUSTUS (29) after training the software with the reference C. glabrata genome (CBS138 strain version 2, NCBI ID 354578, downloaded in March 2018) (Table S6). Predicted genes were then annotated with a blastn or blastp search on the sequence of genes or on the translated amino acid sequence (percentage of identity, ≥98%). The Nanopore-assembled genomes have been deposited at GenBank under the project accession PRJNA589840 (Table S1).

### Illumina library preparation, Illumina read quality control, SNP calling, and coverage analysis.

Strains whose genomes were sequenced in this study are listed in Table S1.

Short-insert paired-end libraries were prepared following manufacturer’s instructions using the Illumina Nextera XT DNA sample preparation kit. Whole-genome sequencing was performed using two Illumina NextSeq 500/550 midoutput kit v2 (2 × 150 cycles) cartridges. The sequencing runs generated 9,966,996 ± 3,108,910 quality paired-end sequences per strain, with a read length average of 133.51 ± 2.86 bp (excluding the primer sequences) (Table S1). Illumina reads were subjected to quality control (filtering and trimming) using Trimmomatic v0.32 (30). Paired reads were filtered with the parameters LEADING:3 TRAILING:3 SLIDINGWINDOW:4:15 MINLEN:36, allowing the elimination of reads with a Phred quality score lower than 33 for more than 30% of their lengths. Paired reads were mapped to the reference genome (Candida glabrata CBS138 strain version 2, NCBI ID 354578, downloaded in March 2018) using Bowtie 2 v2.2.6 (31). The Genome Analysis Toolkit (GATK v2.1) was used for base quality score recalibration, indel (insertion or deletion) realignment, and duplicate removal and to perform SNP and indel discovery (minimum Phred-scaled confidence threshold –stand_call_conf = 30, –base-quality-score-threshold = 6, glm BOTH) (32), resulting in strain-specific vcf files. The resulting SNPs/indels were further filtered with VCFtools (v0.1.13; –max-missing 0.5 –mac 3 –minQ 30).

For genome population analysis and comparison, as well as for GWAS analysis, variants were further filtered with plink (–map3 –indep-pairwise 100b 10 0.8 –allow-no-sex –maf 0.05). Copy number variations (CNVs) were calculated after aligning the Illumina reads against the corresponding newly assembled genomes (obtained as described above): depth of coverage was calculated for each nucleotide of the genome with the SAMtools depth function (33). Depth of coverage was then calculated in sliding windows (size 5,000 bp, 1,000 bp sliding) and then normalized by dividing each value by the geometric mean of the depth of coverage for the corresponding sequencing. CNVs (coverages significantly different between or within chromosomes) were identified by means of the Mann-Whitney U-test and corrected for false-discovery rate. The *dN*/*dS* ratio was calculated by using the software SNPGenie (Table 1 in reference 34). First, all the genetic variants identified by comparing the genome of each strain against the reference genome were merged into single vcf files; two vcf files, one for the forward and one for the complementary reverse sequence, were generated for each chromosome of the reference genome (Table 1 in reference 34). Then, SNPGenie was run on each chromosome, separately for the forward and complementary reverse sequences, by providing the respective vcf, gff (including the reference genome annotations), and the fasta sequence of the reference chromosome. The SNPGenie analysis used the –minfreq: 0.05 option, indicating that the SNP to include in the analysis had to be present at least in the 5% of the population under analysis (Table 1 in reference 34). The *dN*/*dS* ratio was then obtained as the ratio of the calculated mean_dN_vs_ref on the calculated mean_dS_vs_ref.

### Phylogeny and population structure analysis.

SNPs/indels (from vcf files obtained as described above) were concatenated to obtain a single sequence per strain. Pairwise distances were then calculated on the concatenated sequences according to the maximum likelihood method (with the Tamura-Nei model for nucleotide substitution) with MEGA X v10.2.2 (35). Hence, the thus-obtained distance matrices were used to draw the neighbor-joining tree with GrapeTree (36). The analysis was also performed with the Jukes-Cantor, Kimura 2 parameters, and Tamura 3 parameters models, providing substantially identical results (same clustering) compared to the results based on the Tamura-Nei model. The maximum likelihood method was also used to infer the genome phylogeny with IQ-TREE 2 (37), and the neighbor-joining and maximum likelihood-based clusterings were compared with Phylo.io (38). FastSTRUCTURE was used to infer the population structure from the genetic variants among C. glabrata strains (39). The number of clusters best describing the population was inferred according to the method described by Evanno and colleagues (17). In particular, the number of clusters (K) was identified as the minimum number of clusters associated with the highest log likelihood of the data according to the Markov chain Monte Carlo method and maximizing the ΔK (an *ad hoc* quantity related to the second-order rate of change of the log probability of data compared to the number of clusters) (17).

To better dissect the evolutionary relationship among C. glabrata strains, we located the root of the C. glabrata tree by including S. cerevisiae as the out-group. When including S. cerevisiae as the root, we compared the sequences of four marker genes that were shown to be sufficient to recapitulate the genetic divergence within the Ascomycota phylum (18): YHR186C, YMR012W, YJL029C, and YAR007C. The presence of multiple copies of the genes could negatively impact the reliability of the phylogenesis based on these markers (e.g., because of the presence of different alleles). Hence, we first evaluated the copy number of the genes in the analyzed genomes by calculating the gene copy number with the STAR software (–quantMode GeneCounts –sjdbGTFfeatureExon exon –sjdbGTFtagExonParentTranscript transcript_id –sjdbGTFtagExonParentGene transcript_id –sjdbGTFtagExonParentGeneName gene). Then, for each of the four markers, we carried out a one-sample *t* test to assess whether the copy number of the marker was significantly different from the copy number of the entire set of genes in the same genome. The copy number of the marker genes was significantly higher than that of the rest of the genes in most of the genomes (*P* < 0.05); however, this did not affect the assessment of genetic relationships among strains, as suggested by the observation in the tree obtained on the 4 markers, of the same clusters observed in the tree obtained on the entire set of genomic variants. To obtain the phylogenetic tree on the four marker genes, the variants relative to the informative genes (18) were gathered from the full list of variants obtained as described above for each genome. Then, the maximum likelihood (ML) tree was inferred by using the Tamura-Nei model nucleotide substitution model, and the phylogeny was tested over 1,000 bootstrap iterations with MEGA X v10.2.2 (35). The analysis was also performed with the Jukes-Cantor, Kimura 2 parameters, and Tamura 3 parameters models, providing substantially identical results (same clustering) compared to the results based on the Tamura-Nei model. The study by Capella-Gutierrez and colleagues (18) highlighted that multiple copies of the four marker genes were present in the analyzed genomes.

### Chromosomal rearrangements.

Chromosomal rearrangements were inspected by aligning the genome assembly against the reference C. glabrata genome with NUCmer (40) and visualized through a synteny plot generated thanks to a custom Python script (to convert the NUCmer output) and an R script to plot with the ggplot2 R package (41); both scripts are available in the supplemental material. To locate precisely the chromosomal rearrangements (translocations and inversions), we used the STAR software (14). Thanks to the STAR software, we could identify reads mapping partly against a region of a chromosome and partly against a distant part of the same chromosome or another chromosome; hence, the two chromosomal regions were close to each other in the new assembly (in the newly sequenced strain). We initially determined the coordinates of the rearrangements observed through synteny plots in the newly assembled genomes and then searched for junctions at the same locations in other C. glabrata strains sequenced with Illumina in this or previous studies (please refer to Table S1 for the full list of analyzed strains). To further confirm the chromosomal rearrangements, the newly assembled genomes were also compared with three recently assembled genomes: CBS138, BG2, and BG3993 (15, 16). For this purpose, the protocol used in the work by Xu and colleagues (16) was used: each newly assembled genome was aligned against the three new references with NUCmer (-maxmatch -l 100 -c 500); then, the variants were filtered and analyzed with Assemblytics (unique seq length = 5,000, max var size = 50,000, min var size = 50) (13), and the variants were plotted with Circos (42). The linear arrangement of the genes containing the breakpoints located in chromosomes L and I was further visualized with the Integrative Genomics Viewer (IGV).

### Antibiogram profiling.

The susceptibility of the C. glabrata strains isolated during this study was assessed using the serial dilution method published in the EUCAST protocol v7.3.2 (43). In particular, the isolates were inoculated in RPMI 1640-2% glucose overnight with shaking; the next day, a 10^5^ cells/mL suspension of each strain was subjected, in 96-wells plates, to various concentrations of the tested antibiotics. The tested antibiotics and concentrations were caspofungin (1 mg/L, 0.5 mg/L, 0.25 mg/L, 0.125 mg/L, 0.0625 mg/L, 0.03125 mg/L, 0.015625 mg/L, untreated), fluconazole (128 mg/L, 64 mg/L, 16 mg/L, 8 mg/L, 32 mg/L, 4 mg/L, 2 mg/L, untreated), voriconazole (8.192 mg/L, 4.096 mg/L, 2.048 mg/L, 1.024 mg/L, 0.512 mg/L, 0.256 mg/L, 0.128 mg/L, untreated), and flucytosine (65 mg/L, 32.5 mg/L, 16.25 mg/L, 8.125 mg/L, 4.0625 mg/L, 2.03125 mg/L, 1.015625 mg/L, untreated). Then, 24 h after the beginning of the treatment, the optical density (OD_600_) of the cultures was measured and the MICs were determined. The same measurement was repeated after 48 h of treatment, but the results did not differ from the ones obtained after 24 h of treatment. After 48 h of treatment, the death of treated cells was confirmed by plating 50 μL of the culture treated with the MIC onto YPD medium. To determine whether a strain was susceptible (S), intermediate (I), or resistant (R) to each antifungal agent, we used the clinical breakpoint values released by CLSI (44) or EUCAST (v9.0). Using the ED function of the drc R package (45), the results of the serial dilution assay were used to assess the antibiogram profile of each strain, using the MIC of each tested antifungal (caspofungin, fluconazole, voriconazole, and flucytosine [5-fluorocytosine]) against each isolate in triplicate.

### Analysis of genes known to be associated with susceptibility to antifungals.

Previous studies have identified 14 genes whose nucleotide variations have been associated with resistance to antifungals (23). In particular, variations of the sequences of the genes *FKS1*, *FKS2*, and *FKS3* have been shown to be associated with resistance to echinocandin, variations in the genes *FCY1*, *FCY2*, *FUR1*, *FPS1*, and *FPS2* are associated with resistance to 5-fluorocytosine, and the genes *ERG9*, *ERG11*, *CDR1*, *PDR1*, *FLR1*, and *SNQ2* are associated with resistance to azoles (23). For each of these genes, we identified the nonsynonymous mutations in each genome sequenced over this study. Then, we searched for associations between the presence of each identified nonsynonymous variant and determined the susceptibility of the strain against the tested antifungals by searching for variants that were consistently (preferentially or always) present in strains categorized as R, I, or S.

### Genome-wide association analyses.

The genetic variants resulting from Illumina sequencing analysis was tested for associations with the measured quantitative phenotype (MIC values assessed as described in previous sections) by using GEMMA (46), allowing correction for sample relatedness and population stratification. At first, the centered relatedness matrix was calculated from genotypes (-gk 1), and then it was used for the association test with the univariate linear model (-lmm 4).

The relatedness matrix for genotypes (**K**) was calculated with GEMMA as shown in equation 1:

(1) $$K=\frac{1}{p}\overset{p}{\underset{i=1}{\sum }}\left(x_{i}-1_{n}x{¯}_{i}\right){\left(x_{i}-1_{n}x{¯}_{i}\right)}^{T}$$

where *n* is the number of individuals, *p* denotes the genetic markers, **x** is the *n *×* p* matrix of genotypes, ***x***_i_ is its *i*th column (vector) representing genotypes of the *i*th SNP, *x* is the sample mean, and 1*_n_* is an *n *× 1 vector of 1s.

GEMMA was used to fit a univariate linear mixed model shown in equation 2:

(2) $$y=W\alpha +x\beta +u+\epsilon ;\;u\sim MVN_{n}\left(0,\lambda \tau^{-1}K\right),\epsilon \sim MVN_{n}\left(0,\tau^{-1}I_{n}\right)$$

where *n* is the number of individuals, *c* is the number of covariates, **y** is an *n *× 1 vector of quantitative traits, **W** (**w**_1_,…, **w**_c_) is an *n *×* c* matrix of covariates including a column of 1s, **α** is a *c *× 1 vector of the corresponding coefficients including the intercept, **x** is an *n *× 1 vector of marker genotypes, *β* is the effect size of the marker, **Z** is an *n *×* m* loading matrix, **u** is an *n *× 1 vector of effects, **ε** is an *n *× 1 vector of errors, *τ*^−1^ is the variance of the residual errors, *λ* is the ratio between the two variance components, **K** is the *n *×* n* relatedness matrix previously calculated, and **I***_n_* is an *n *×* n* identity matrix. MVN denotes the *n*- dimensional multivariate normal distribution. To run GWAS analysis, genomic variants were filtered with PLINK with the constraint –indep-pairwise 100b 10 0.8 (window size = 100bp, step = 10bp, r^2 threshold = 0.8, to filter tightly linked variants) –allow-no-sex –maf 0.05 (minimum allele frequency) –nonfounders (considering all the individuals as founder in the Hardy-Weinberg equilibrium tests). No additional covariates were included in the model. The analysis was carried out on each set of MIC values (for each strain against each tested antifungal) obtained with three independent biological analyses. Variants were considered associated with the phenotype when showing a *P* value of <0.001. In addition, only the variants significantly associated with the phenotype in all the three biological replicates were maintained in the analysis.

### Data availability.

Illumina reads were deposited in the SRA NCBI database under the BioProject no. PRJNA589840 and BioSample no. SAMN18953774 to SAMN18953803 (Table S1).

## References

[1] Raja NS. 2021. Epidemiology, risk factors, treatment and outcome of *Candida* bloodstream infections because of *Candida albicans* and *Candida* non-*albicans* in two district general hospitals in the United Kingdom. Int J Clin Pract 75:e13655. doi:10.1111/ijcp.13655. 32869497

[2] Koehler P, Stecher M, Cornely OA, Koehler D, Vehreschild MJGT, Bohlius J, Wisplinghoff H, Vehreschild JJ. 2019. Morbidity and mortality of candidaemia in Europe: an epidemiologic meta-analysis. Clin Microbiol Infect 25:1200–1212. doi:10.1016/j.cmi.2019.04.024. 31039444

[3] Wee LE, Wong CSL, Tan AL, Oh HM-L. 2021. Negative cerebrospinal fluid β-d-glucan levels as an indicator for treatment cessation ahead of biochemical resolution: a case report of *Candida glabrata* meningitis. Med Mycol Case Rep 32:47–49. doi:10.1016/j.mmcr.2021.03.003. 33786294 PMC7994442

[4] Farmakiotis D, Kyvernitakis A, Tarrand JJ, Kontoyiannis DP. 2015. Early initiation of appropriate treatment is associated with increased survival in cancer patients with *Candida glabrata* fungaemia: a potential benefit from infectious disease consultation. Clin Microbiol Infect 21:79–86. doi:10.1016/j.cmi.2014.07.006. 25636931

[5] Gamal A, Chu S, McCormick TS, Borroto-Esoda K, Angulo D, Ghannoum MA. 2021. Ibrexafungerp, a novel oral triterpenoid antifungal in development: overview of antifungal activity against *Candida glabrata*. Front Cell Infect Microbiol 11:642358. doi:10.3389/fcimb.2021.642358. 33791244 PMC8006402

[6] Logan C, Martin-Loeches I, Bicanic T. 2020. Invasive candidiasis in critical care: challenges and future directions. Intensive Care Med 46:2001–2014. doi:10.1007/s00134-020-06240-x. 32990778

[7] Fidel PL, Jr, Vazquez JA, Sobel JD. 1999. *Candida glabrata*: review of epidemiology, pathogenesis, and clinical disease with comparison to *C. albicans*. Clin Microbiol Rev 12:80–96. doi:10.1128/CMR.12.1.80. 9880475 PMC88907

[8] Dodgson AR, Pujol C, Denning DW, Soll DR, Fox AJ. 2003. Multilocus sequence typing of *Candida glabrata* reveals geographically enriched clades. J Clin Microbiol 41:5709–5717. doi:10.1128/JCM.41.12.5709-5717.2003. 14662965 PMC309006

[9] Gabaldón T, Gómez-Molero E, Bader O. 2019. Molecular typing of *Candida glabrata*. Mycopathologia 185:755–764. doi:10.1007/s11046-019-00388-x. 31617105

[10] Gabaldón T, Carreté L. 2016. The birth of a deadly yeast: tracing the evolutionary emergence of virulence traits in *Candida glabrata*. FEMS Yeast Res 16:fov110. doi:10.1093/femsyr/fov110. 26684722 PMC5815135

[11] Gabaldón T, Fairhead C. 2019. Genomes shed light on the secret life of *Candida glabrata*: not so asexual, not so commensal. Curr Genet 65:93–98. doi:10.1007/s00294-018-0867-z. 30027485 PMC6342864

[12] Carreté L, Ksiezopolska E, Pegueroles C, Gómez-Molero E, Saus E, Iraola-Guzmán S, Loska D, Bader O, Fairhead C, Gabaldón T. 2018. Patterns of genomic variation in the opportunistic pathogen *Candida glabrata* suggest the existence of mating and a secondary association with humans. Curr Biol 28:15–27.e7. doi:10.1016/j.cub.2017.11.027. 29249661 PMC5772174

[13] Nattestad M, Schatz MC. 2016. Assemblytics: a web analytics tool for the detection of variants from an assembly. Bioinformatics 32:3021–3023. doi:10.1093/bioinformatics/btw369. 27318204 PMC6191160

[14] Dobin A, Davis CA, Schlesinger F, Drenkow J, Zaleski C, Jha S, Batut P, Chaisson M, Gingeras TR. 2013. STAR: ultrafast universal RNA-seq aligner. Bioinformatics 29:15–21. doi:10.1093/bioinformatics/bts635. 23104886 PMC3530905

[15] Xu Z, Green B, Benoit N, Schatz M, Wheelan S, Cormack B. 2020. *De novo* genome assembly of *Candida glabrata* reveals cell wall protein complement and structure of dispersed tandem repeat arrays. Mol Microbiol 113:1209–1224. doi:10.1111/mmi.14488. 32068314

[16] Xu Z, Green B, Benoit N, Sobel JD, Schatz MC, Wheelan S, Cormack BP. 2021. Cell wall protein variation, break-induced replication, and subtelomere dynamics in *Candida glabrata*. Mol Microbiol 116:260–276. doi:10.1111/mmi.14707. 33713372

[17] Evanno G, Regnaut S, Goudet J. 2005. Detecting the number of clusters of individuals using the software structure: a simulation study. Mol Ecol 14:2611–2620. doi:10.1111/j.1365-294X.2005.02553.x. 15969739

[18] Capella-Gutierrez S, Kauff F, Gabaldón T. 2014. A phylogenomics approach for selecting robust sets of phylogenetic markers. Nucleic Acids Res 42:e54. doi:10.1093/nar/gku071. 24476915 PMC3985644

[19] Huang M, Khan J, Kaur M, Vanega JDT, Patiño OAA, Ramasubramanian AK, Kao KC. 2019. CgSTE11 mediates cross tolerance to multiple environmental stressors in *Candida glabrata*. Sci Rep 9:17036. doi:10.1038/s41598-019-53593-5. 31745168 PMC6863853

[20] Guo X, Zhang R, Li Y, Wang Z, Ishchuk OP, Ahmad KM, Wee J, Piskur J, Shapiro JA, Gu Z. 2020. Understand the genomic diversity and evolution of fungal pathogen *Candida glabrata* by genome-wide analysis of genetic variations. Methods 176:82–90. doi:10.1016/j.ymeth.2019.05.002. 31059831

[21] Otterstedt K, Larsson C, Bill RM, Ståhlberg A, Boles E, Hohmann S, Gustafsson L. 2004. Switching the mode of metabolism in the yeast *Saccharomyces cerevisiae*. EMBO Rep 5:532–537. doi:10.1038/sj.embor.7400132. 15071495 PMC1299050

[22] The European Committee on Antimicrobial Susceptibility Testing, Antifungal Agents, Breakpoint tables for interpretation of MICs, Version 9.0, valid 2018–2020. https://www.eucast.org/fileadmin/src/media/PDFs/EUCAST_files/AFST/Clinical_breakpoints/Antifungal_breakpoints_v_9.0_180212.pdf.

[23] Biswas C, Chen SC-A, Halliday C, Kennedy K, Playford EG, Marriott DJ, Slavin MA, Sorrell TC, Sintchenko V. 2017. Identification of genetic markers of resistance to echinocandins, azoles and 5-fluorocytosine in *Candida glabrata* by next-generation sequencing: a feasibility study. Clin Microbiol Infect 23:676.e7–676.e10. doi:10.1016/j.cmi.2017.03.014. 28344162

[24] Casci T. 2009. A portrait of yeast. Nat Rev Genet 10:223–223. doi:10.1038/nrg2561.

[25] Tam P, Gee K, Piechocinski M, Macreadie I. 2015. *Candida glabrata*, friend and foe. J Fungi (Basel) 1:277–292. doi:10.3390/jof1020277. 29376912 PMC5753114

[26] Li H. 2018. Minimap2: pairwise alignment for nucleotide sequences. Bioinformatics 34:3094–3100. doi:10.1093/bioinformatics/bty191. 29750242 PMC6137996

[27] Koren S, Walenz BP, Berlin K, Miller JR, Bergman NH, Phillippy AM. 2017. Canu: scalable and accurate long-read assembly via adaptive k-mer weighting and repeat separation. Genome Res 27:722–736. doi:10.1101/gr.215087.116. 28298431 PMC5411767

[28] Walker BJ, Abeel T, Shea T, Priest M, Abouelliel A, Sakthikumar S, Cuomo CA, Zeng Q, Wortman J, Young SK, Earl AM. 2014. Pilon: an integrated tool for comprehensive microbial variant detection and genome assembly improvement. PLoS One 9:e112963. doi:10.1371/journal.pone.0112963. 25409509 PMC4237348

[29] Stanke M, Tzvetkova A, Morgenstern B. 2006. AUGUSTUS at EGASP: using EST, protein and genomic alignments for improved gene prediction in the human genome. Genome Biol 7:S11. doi:10.1186/gb-2006-7-s1-s11. PMC1810548 16925833

[30] Bolger AM, Lohse M, Usadel B. 2014. Trimmomatic: a flexible trimmer for Illumina sequence data. Bioinformatics 30:2114–2120. doi:10.1093/bioinformatics/btu170. 24695404 PMC4103590

[31] Langmead B, Salzberg SL. 2012. Fast gapped-read alignment with Bowtie 2. Nat Methods 9:357–359. doi:10.1038/nmeth.1923. 22388286 PMC3322381

[32] McKenna A, Hanna M, Banks E, Sivachenko A, Cibulskis K, Kernytsky A, Garimella K, Altshuler D, Gabriel S, Daly M, DePristo MA. 2010. The Genome Analysis Toolkit: a MapReduce framework for analyzing next-generation DNA sequencing data. Genome Res 20:1297–1303. doi:10.1101/gr.107524.110. 20644199 PMC2928508

[33] Li H, Handsaker B, Wysoker A, Fennell T, Ruan J, Homer N, Marth G, Abecasis G, Durbin R, 1000 Genome Project Data Processing Subgroup. 2009. The Sequence Alignment/Map format and SAMtools. Bioinformatics 25:2078–2079. doi:10.1093/bioinformatics/btp352. 19505943 PMC2723002

[34] Nelson CW, Moncla LH, Hughes AL. 2015. SNPGenie: estimating evolutionary parameters to detect natural selection using pooled next-generation sequencing data. Bioinformatics 31:3709–3711. doi:10.1093/bioinformatics/btv449. 26227143 PMC4757956

[35] Kumar S, Stecher G, Li M, Knyaz C, Tamura K. 2018. MEGA X: Molecular Evolutionary Genetics Analysis across computing platforms. Mol Biol Evol 35:1547–1549. doi:10.1093/molbev/msy096. 29722887 PMC5967553

[36] Zhou Z, Alikhan N-F, Sergeant MJ, Luhmann N, Vaz C, Francisco AP, Carriço JA, Achtman M. 2018. GrapeTree: visualization of core genomic relationships among 100,000 bacterial pathogens. Genome Res 28:1395–1404. doi:10.1101/gr.232397.117. 30049790 PMC6120633

[37] Minh BQ, Schmidt HA, Chernomor O, Schrempf D, Woodhams MD, von Haeseler A, Lanfear R. 2020. IQ-TREE 2: new models and efficient methods for phylogenetic inference in the genomic era. Mol Biol Evol 37:1530–1534. doi:10.1093/molbev/msaa015. 32011700 PMC7182206

[38] Robinson O, Dylus D, Dessimoz C. 2016. Phylo.io: interactive viewing and comparison of large phylogenetic trees on the web. Mol Biol Evol 33:2163–2166. doi:10.1093/molbev/msw080. 27189561 PMC4948708

[39] Raj A, Stephens M, Pritchard JK. 2014. fastSTRUCTURE: variational inference of population structure in large SNP data sets. Genetics 197:573–589. doi:10.1534/genetics.114.164350. 24700103 PMC4063916

[40] Delcher AL, Phillippy A, Carlton J, Salzberg SL. 2002. Fast algorithms for large-scale genome alignment and comparison. Nucleic Acids Res 30:2478–2483. doi:10.1093/nar/30.11.2478. 12034836 PMC117189

[41] Wickham H. 2009. ggplot2. Springer, New York, NY.

[42] Krzywinski M, Schein J, Birol İ, Connors J, Gascoyne R, Horsman D, Jones SJ, Marra MA. 2009. Circos: an information aesthetic for comparative genomics. Genome Res 19:1639–1645. doi:10.1101/gr.092759.109. 19541911 PMC2752132

[43] Arendrup MC, Cuenca-Estrella M, Lass-Flörl C, Hope W, EUCAST-AFST. 2012. EUCAST technical note on the EUCAST definitive document EDef 7.2: method for the determination of broth dilution minimum inhibitory concentrations of antifungal agents for yeasts EDef 7.2 (EUCAST-AFST). Clin Microbiol Infect 18:E246–E247. doi:10.1111/j.1469-0691.2012.03880.x. 22563750

[44] CLSI. 2020. Performance standards for antifungal susceptibility testing of yeasts, 2nd ed. M60. Clinical and Laboratory Standards Institute, Wayne, PA.

[45] Ritz C, Baty F, Streibig JC, Gerhard D. 2015. Dose-response analysis using R. PLoS One 10:e0146021. doi:10.1371/journal.pone.0146021. 26717316 PMC4696819

[46] Zhou X, Stephens M. 2012. Genome-wide efficient mixed-model analysis for association studies. Nat Genet 44:821–824. doi:10.1038/ng.2310. 22706312 PMC3386377

