# Supplementary material for: Genomic Assembly of Clinical Candida glabrata (Nakaseomyces glabrata) Isolates Reveals within-Species Structural Plasticity and Association with In Vitro Antifungal Susceptibility
Source: Microbiol Spectr. 2022 Nov 10;10(6):e01827-22. doi: 10.1128/spectrum.01827-22 (PMC9769630; doi:10.1128/spectrum.01827-22)
Supplement: Supplemental file 1 — Fig. S1 to S21, custom script, and Tables S1 to S5. Download spectrum.01827-22-s0001.pdf, PDF file, 5.3 MB [file spectrum.01827-22-s0001.pdf]

Supplementary information for

Genomic assembly of clinical [*Candida*] *glabrata* (*Nakaseomyces glabrata*) isolates reveals within-species structural plasticity and association with in vitro antifungal susceptibility

Irene Stefanini[1], Emily Stoakes[2], Houdini H.T. Wu[3], Li Xu-McCrae[3], Abid Hussain[3], John Moat[2], Christopher Dowson[2], Miruna D. David[4] and Chrystala Constantinidou[2]

[1]Department of Life Sciences and Systems Biology, University of Turin, Turin, IT;
[2]University of Warwick, Coventry, UK;
[3]UK Health Security Agency Public Health Laboratory, Birmingham, UK;
[4]University Hospitals Birmingham NHS Foundation Trust, UK

This pdf includes (click on the blue underlined text to move to the corresponding item):

Supplementary Figures

Supplementary figure 1: Depth of coverage of the region supposed to include a chromosomal fusion in one of the contig (utg000005l) of the assembled genome of strain CG_UHB_01.

Supplementary figure 2: Comparison of synteny plots obtained on the genomes assembled with Canu and Minimap2.

Supplementary figure 3: Linear arrangement of the genes containing the breakpoints located in chromosomes L and I.

Supplementary figure 4 Correlations among the presence of chromosomal rearrangements and strains characteristics.

Supplementary figure 5: Rearrangements of the genomes assembled in this study in comparison with the CBS138, BG2, and BG3993 genomes.

Supplementary figure 6: Phylogenetic tree of *Candida glabrata* isolates annotated with bootstrap values (100 iterations).

Supplementary figure 7: Comparison of Neighbor-Joining and Maximum Likelihood-based clustering obtained on the entire set of genetic variants among the studied genomes.

Supplementary figure 8: Selection of the best partition of genomes into clades.

Supplementary figure 9: Phylogenetic tree of *Candida glabrata* isolates using *Saccharomyces cerevisiae* as the root of the tree.

Supplementary figure 10: Phylogenetic tree of *Candida glabrata* isolates using *Saccharomyces cerevisiae* as the root of the tree, annotated with bootstrap support values calculated over 1000 bootstrap iterations.

Supplementary figure 11: Phylogenetic trees of *Candida glabrata* isolates.

Supplementary figure 12: Comparison of genetic variants in the rearranged regions in genomes bearing the rearrangements and in genomes not bearing the rearrangements.

Supplementary figure 13: Comparison of phylogenetic trees of *Candida glabrata* isolates based on sets of genomic variants including or not including these located in the rearranged regions.
Supplementary figure 14: Coverage depth of sequenced strains.
Supplementary figure 15: Manhattan and QQ plots of GWAS analysis.
Supplementary figure 16: Alleles identified through GWAS analysis as associated with the response to caspofungin.
Supplementary figure 17: Alleles identified through GWAS analysis as associated with the response to fluconazole.
Supplementary figure 18: Alleles identified through GWAS analysis as associated with the response to voriconazole
Supplementary figure 19: Alleles identified through GWAS analysis as associated with the response to flucytosine
Supplementary figure 20: Number of missense SNPs per strain found to be associated with the response to the tested antifungals and relation with the response to the antifungal.
Supplementary figure 21: Relations between the presence of chromosomal rearrangements and the response to the tested antifungals.
Custom scripts: Custom Python and R script to generate syntheny plots from nucmer outputs.

Additional supplementary information

Supplementary Tables and Data sets

Supplementary Table 1: Strains analyzed in this study and sequencing information.
Supplementary Table 2: Information on the presence/absence of chromosomal rearrangements in the *C. glabrata* strains isolated in this or previous studies.
Supplementary Table 3: Information on genes highly conserved (dN/dS=0) or subjected to positive selection (dN/dS=1) in the 30 *C. glabrata* strains genomes sequenced in this study.
Supplementary Table 4: Associations between genetic variations (non-synonymous mutations) in genes known to be associated with the resistance to antifungals and the susceptibility of the strains isolated in this study against the tested antifungals.
Supplementary Table 5: List of missense (non-synonymous) mutations found to be associated with the phenotype (resistance to antifungals).
Data Set S1: Results of Assemblytics analysis. (file “Data_set_S1.xlsx”).
Data Set S2: Results of STAR analysis for the identification of chromosomal rearrangements. (file “Data_set_S2.xlsx”).
Data Set S3: Annotations of new genomes. (file “Data_set_S3.xlsx”).
Data Set S4: Results of snpgenie analysis on dN/dS (file “Data_set_S4.xlsx”).

Supplementary information

Supplementary figure 1

Depth of coverage of the region supposed to include a chromosomal fusion in one of the contig (utg000005l) of the assembled genome of strain CG_UHB_01.

a) screenshot of the Illumina reads obtained with the IGV software. b) Depth of coverage calculated with the samtools depth function by aligning the Illumina reads against the contig utg000005l and computing the average depth of coverage over sliding windows (size 1000bp, 100bp sliding).

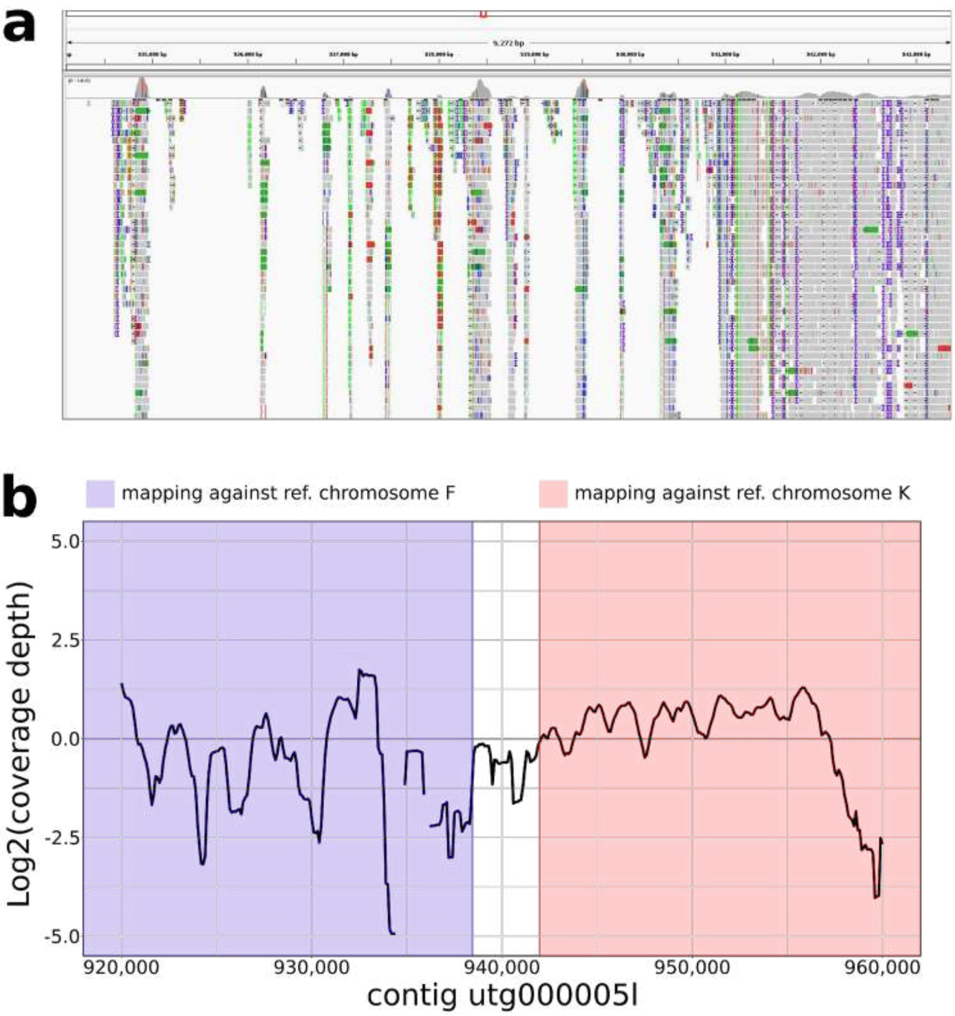

Back to the index

Supplementary information

Supplementary figure 2
Comparison of synteny plots obtained on the genomes assembled with Canu and Minimap2.

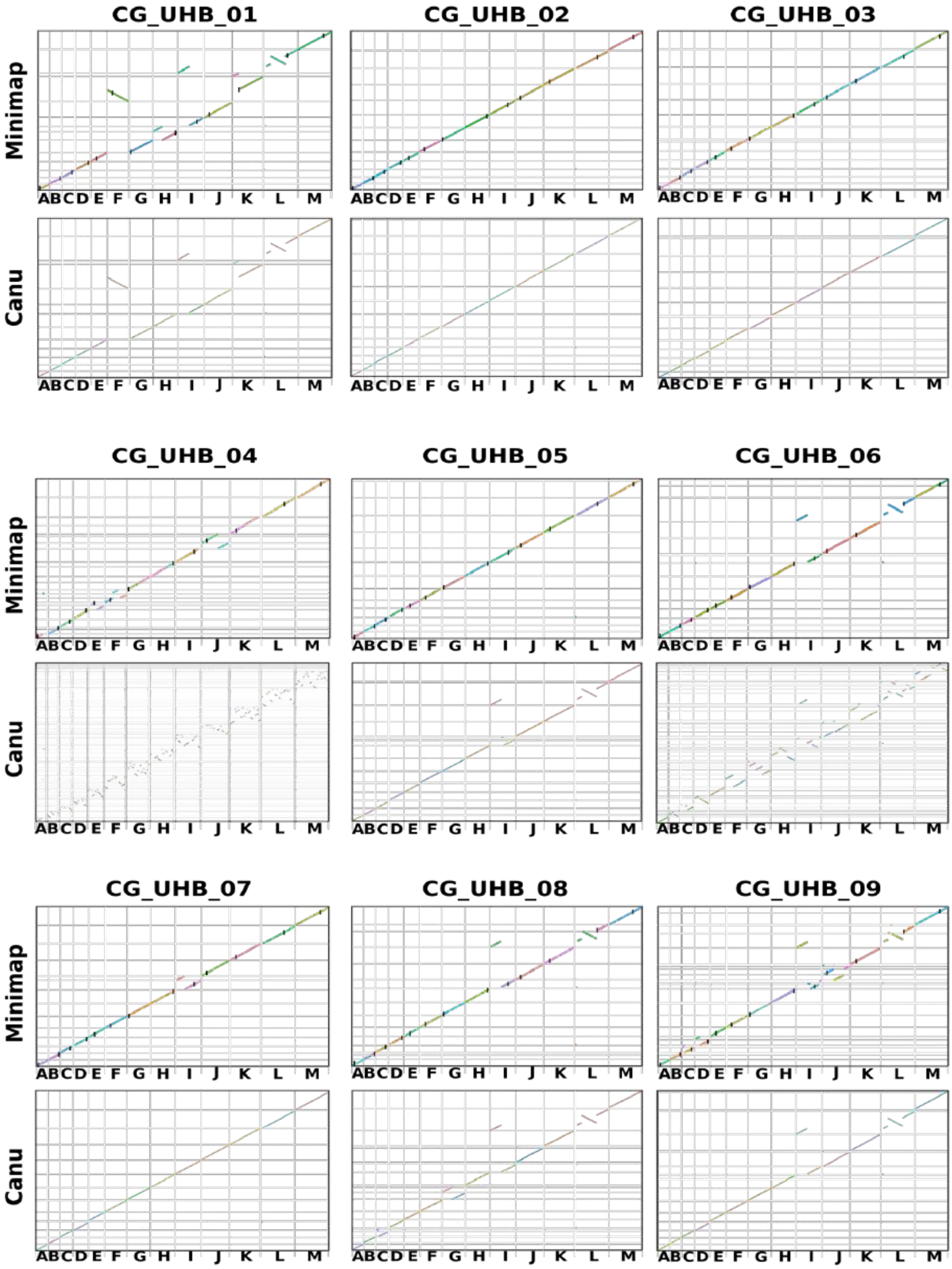

Back to the index

Supplementary figure 3

Linear arrangement of the genes containing the breakpoints located in chromosomes L and I. The alignment of the Illumina reads of two representative genomes (of strain CG_UHB_01, including the rearrangements, and CG_UHB_02, not including the rearrangements) are shown. The alignment against the reference genome (CBS138) was visualized with IGV (Robinson et al. 2011). The vertical yellow line indicates the position of the breakpoint at **a)** the beginning of the inversion in chromosome L, **b)** the end of the inversion in chromosome L, and **c)** the location of the translocation in chromosome I. The numbers in the yellow circles indicate the genomic features (e.g. genes, mRNA, five_prime_UTR, CDS, three_prime_UTR,...) as reported in the reference genome's gff file (*Candida glabrata* CBS138 strain version 2, NCBI ID = 354578, downloaded on March 2018).

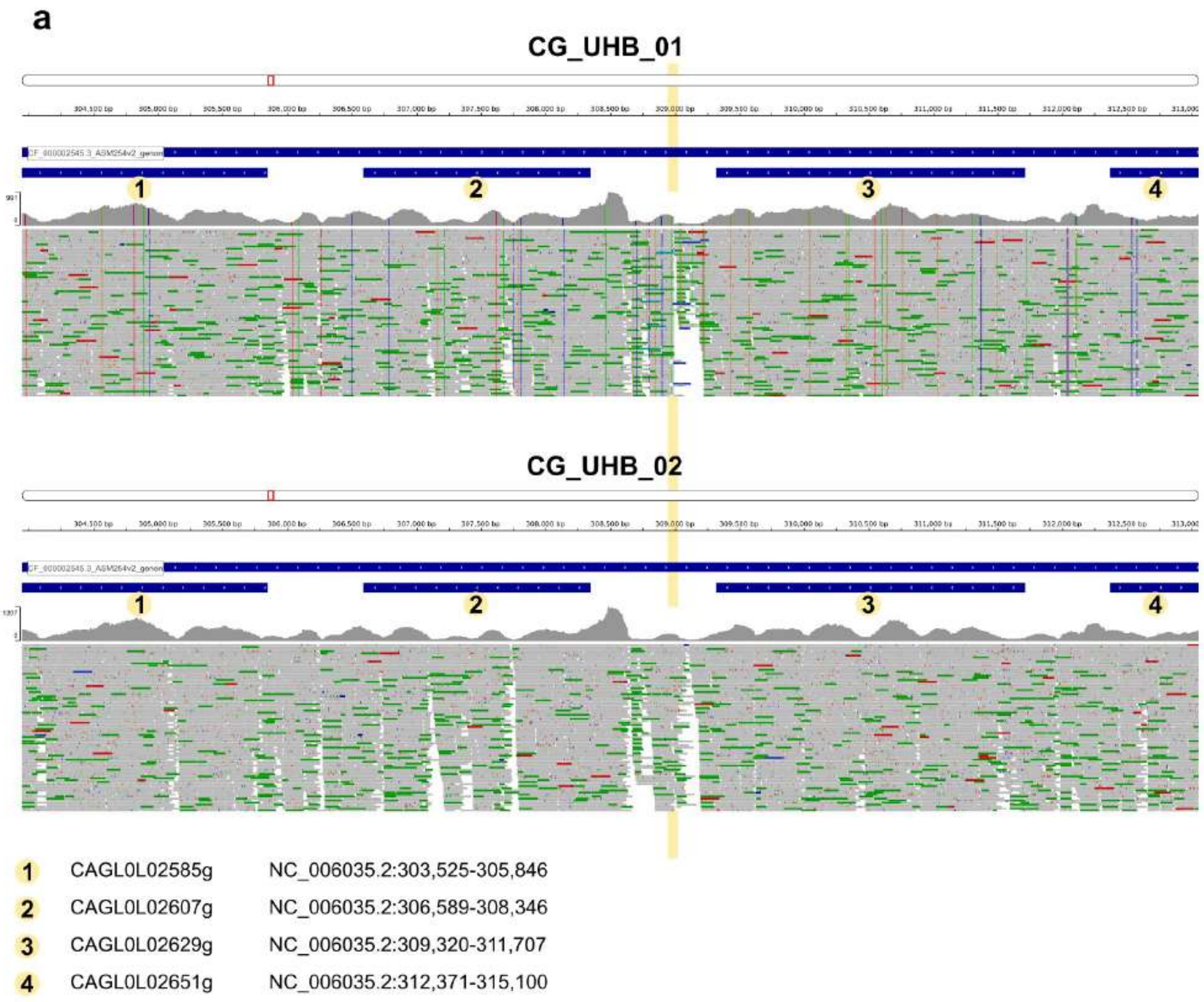

Supplementary information

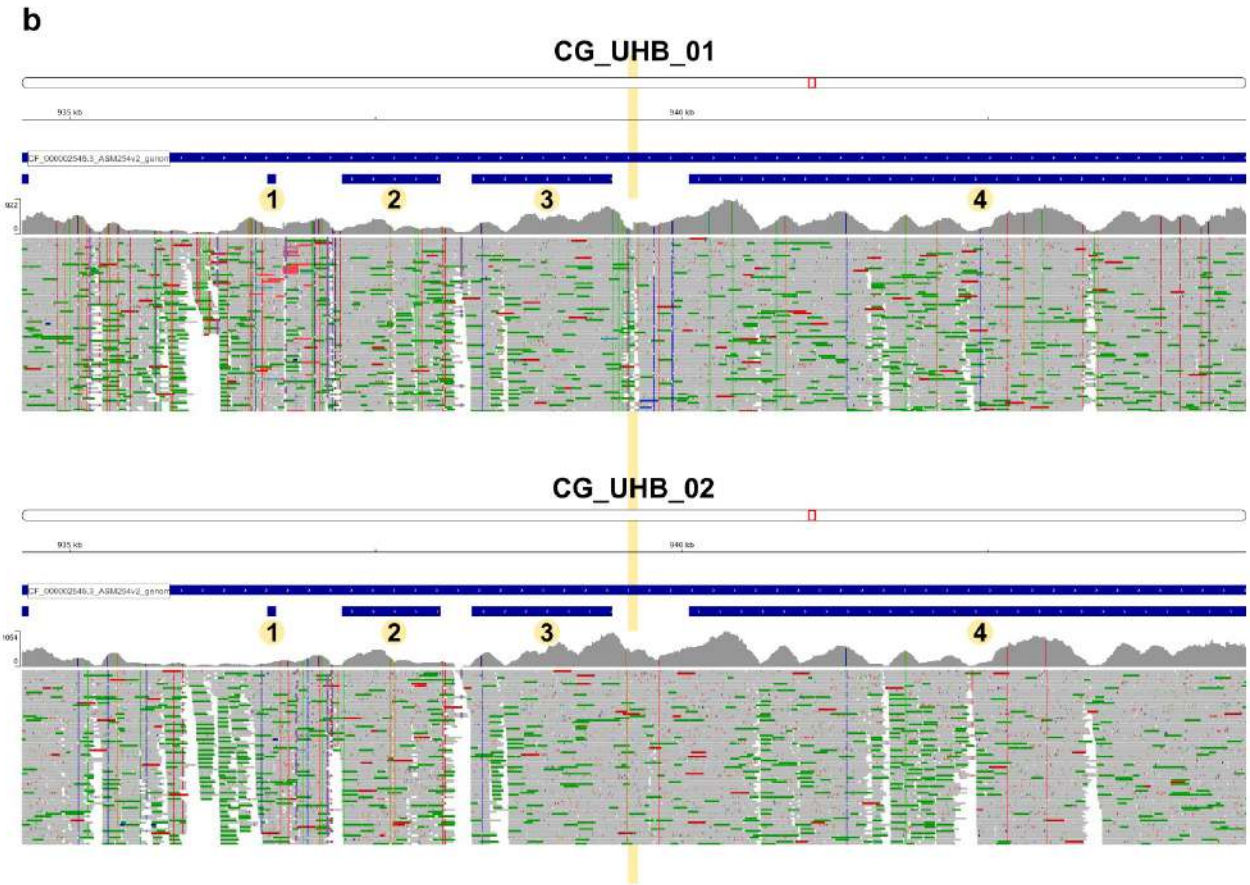

1	tRNA-Ile (AAT)	NC_006035.2:936,615-936,688
2	CAGL0L08580g	NC_006035.2:937,224-938,030
3	CAGL0L08602g	NC_006035.2:938,288-939,430
4	CAGL0L08624g	NC_006035.2:940,058-946,312

c

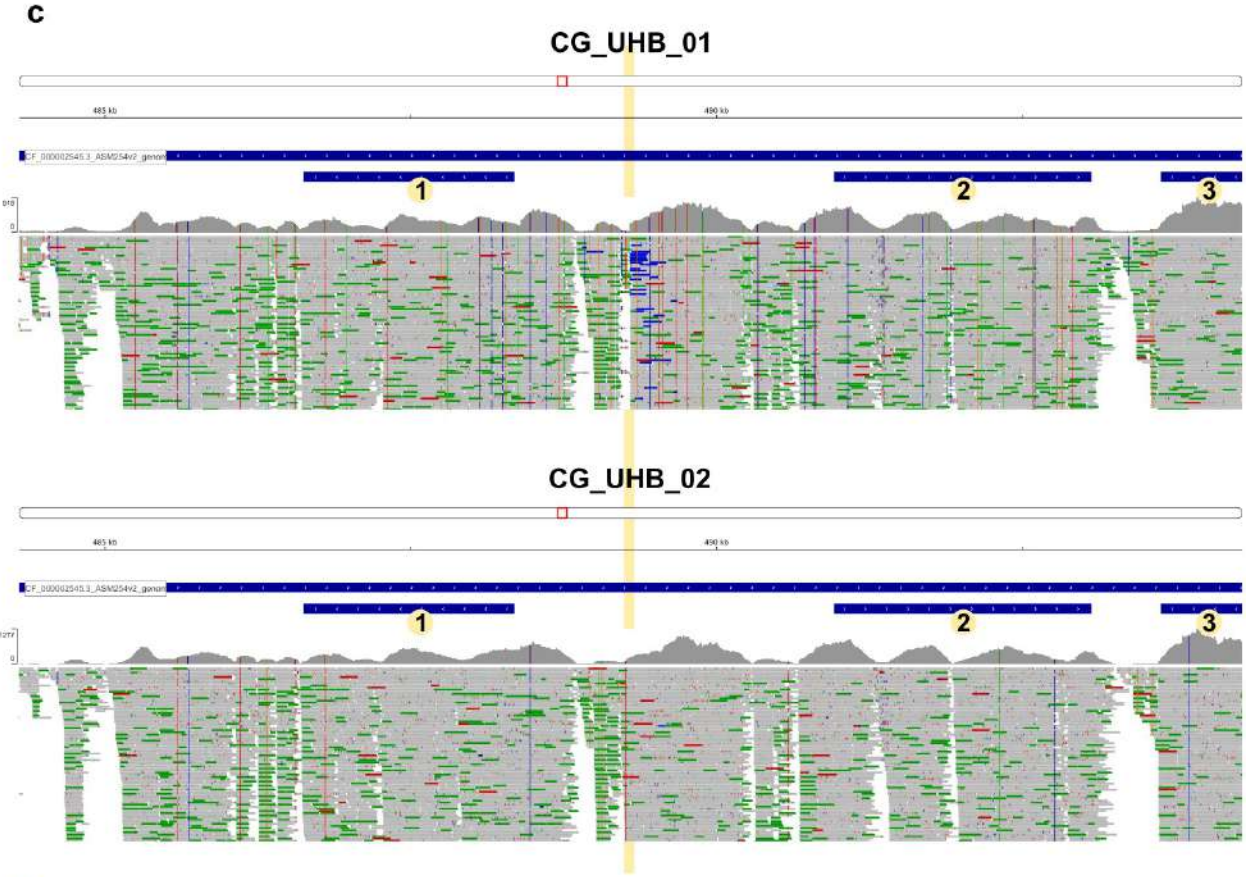

1	CAGL0I05148g	NC_006032.2:486,625-488,349
2	CAGL0I05170g	NC_006032.2:490,959-493,064
3	CAGL0I05192g	NC_006032.2:493,635-494,756

Back to the index

Supplementary figure 4

Correlations among the presence of chromosomal rearrangements and strains characteristics. a) Country of isolation, b) year of isolation, c) source of isolation, and d) genomic clusters. Upper plots show graphical representations, plotted with the balloonplot function of the gplots R package (Warnes et al. 2020), of the contingency tables, with the radius of blue dots being proportional to the number of isolates corresponding to the relative group. Lower plots show the Pearson's residuals calculated with the chisq.test R function of the stat R package (R core team 2020) and plotted with the corrplot function of the corrplot R package (Wei and Simko 2017).

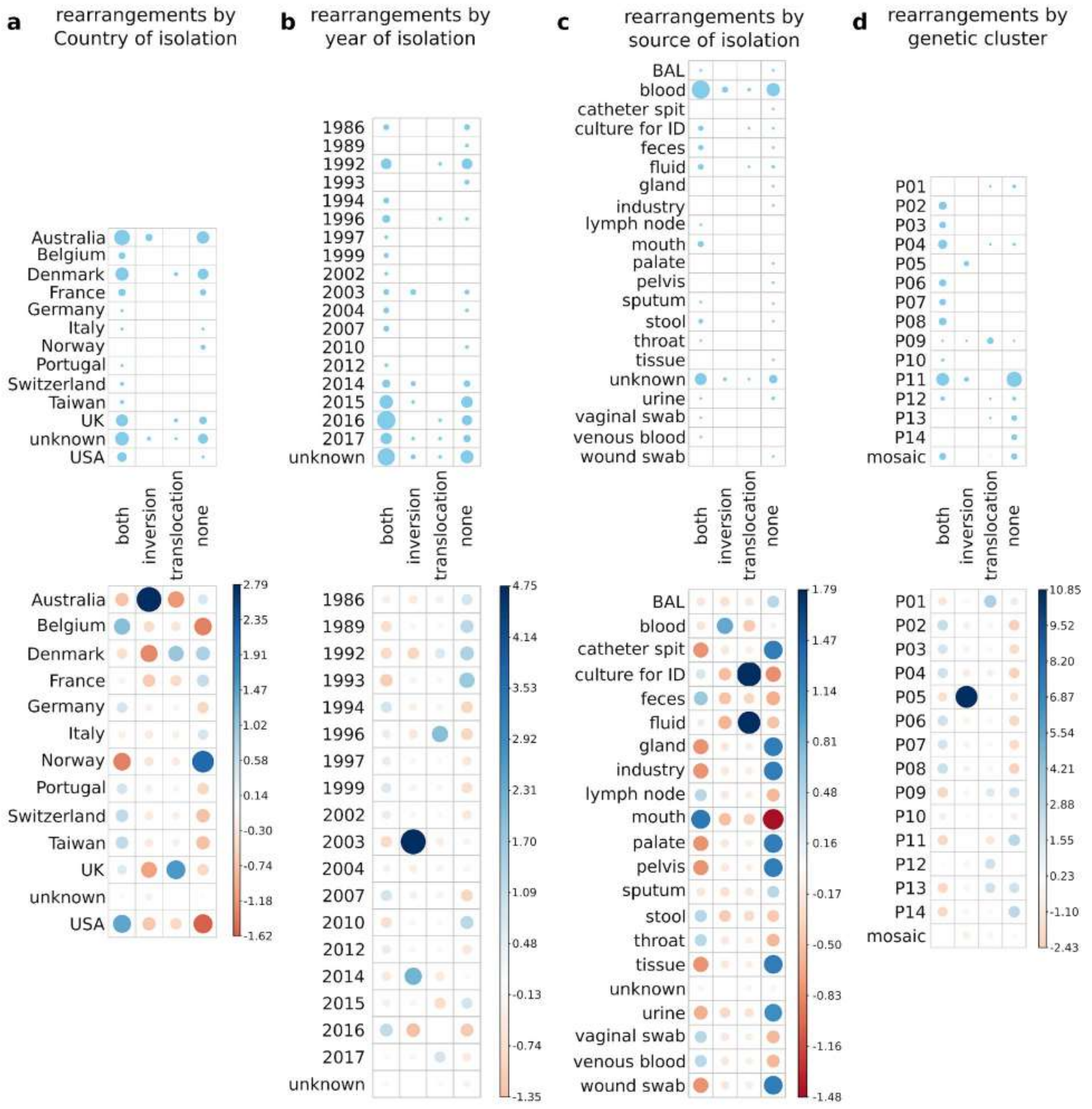

Back to the index

Supplementary figure 5

Rearrangements of the genomes assembled over this study in comparison with the CBS138, BG2, and BG3993 genomes. The strips indicate chromosomal rearrangements. The color of the strip corresponds to the color of the chromosome in the reference genome. The white numbers in the blue rectangles - indicating the contigs of the genomes assembled in this study - correspond to the contig ids as reported in the sequences deposited in the GenBank database (PRJNA589840). The arrangements were identified with Assemblytics (Nattesdad and Schatz 2016) and visualized with circos (Krzywinski et al. 2009).

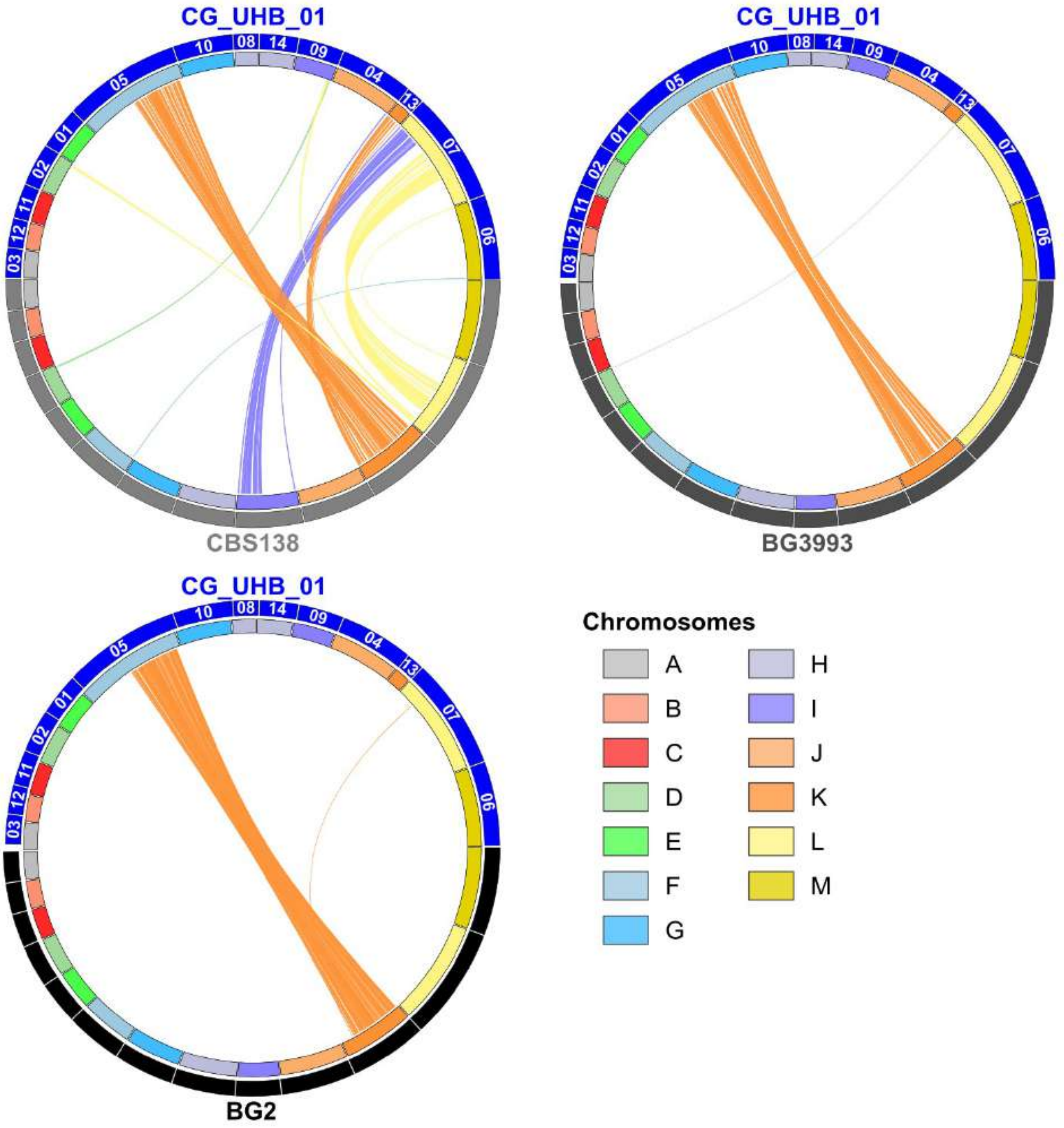

Supplementary information

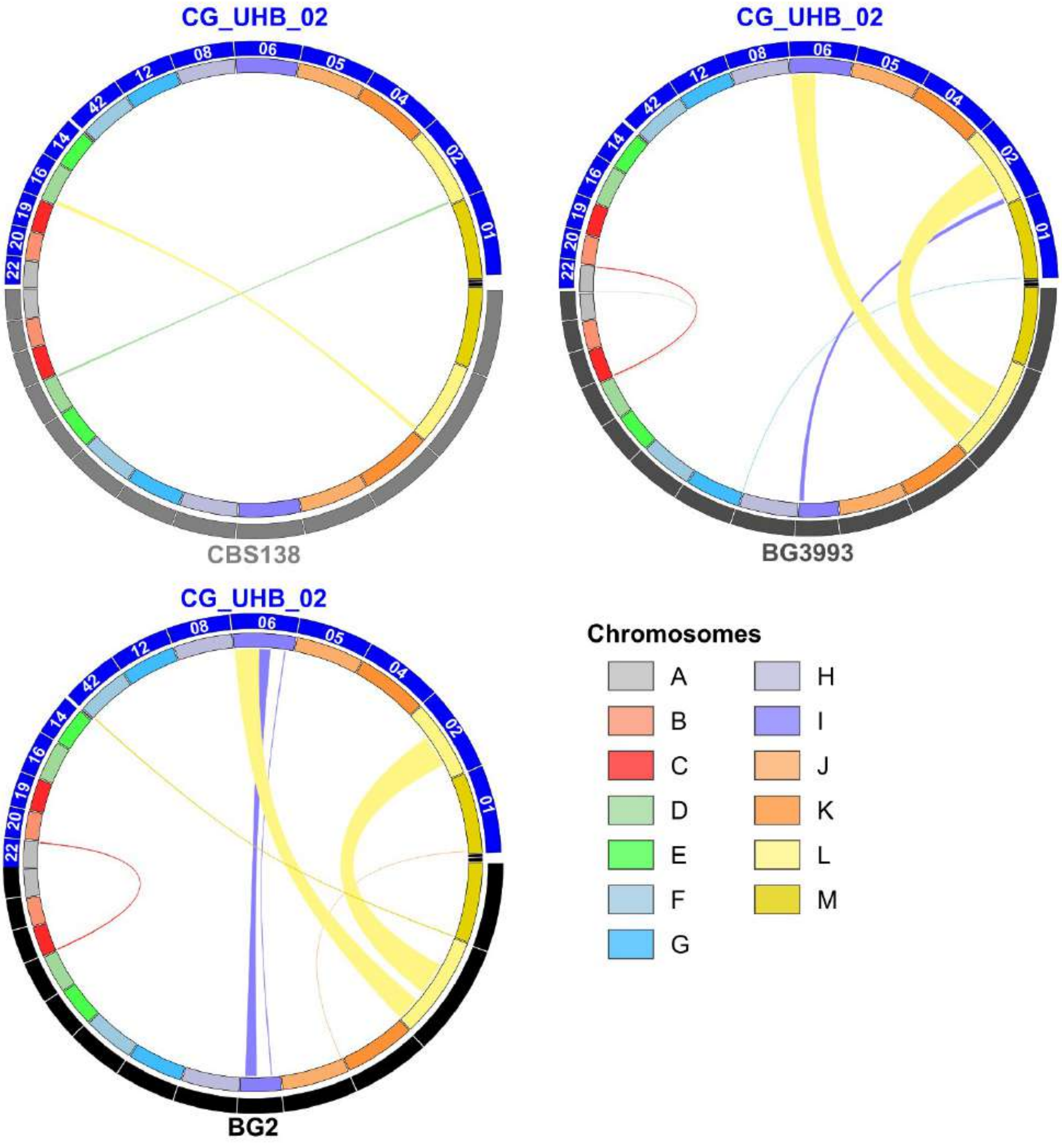

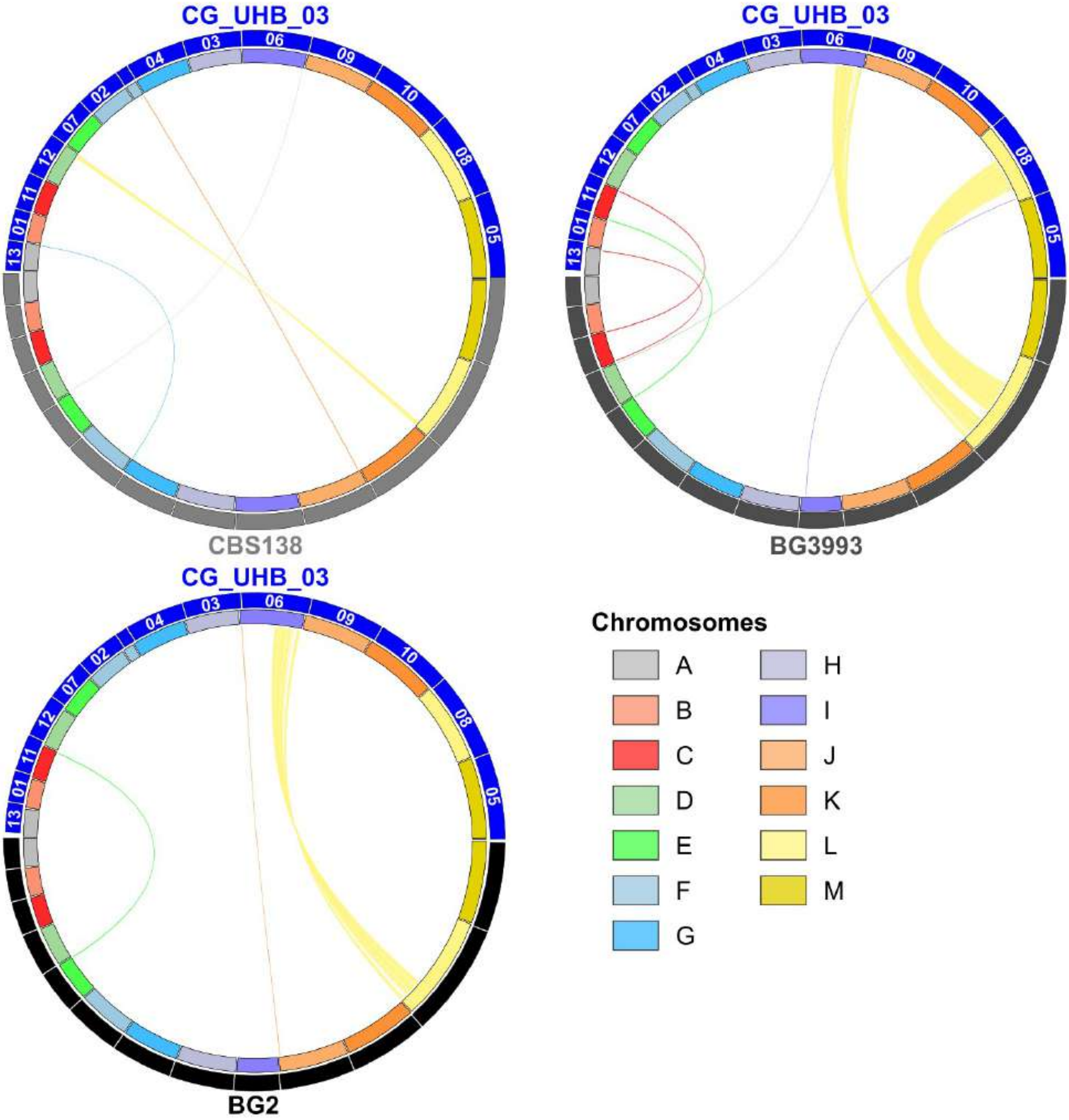
CG_UHB_03
06
09
10
08
05
13
01
11
12
07
02
04
03
CBS138
BG3993
BG2
Chromosomes
A
B
C
D
E
F
G
H
I
J
K
L
M

Supplementary information

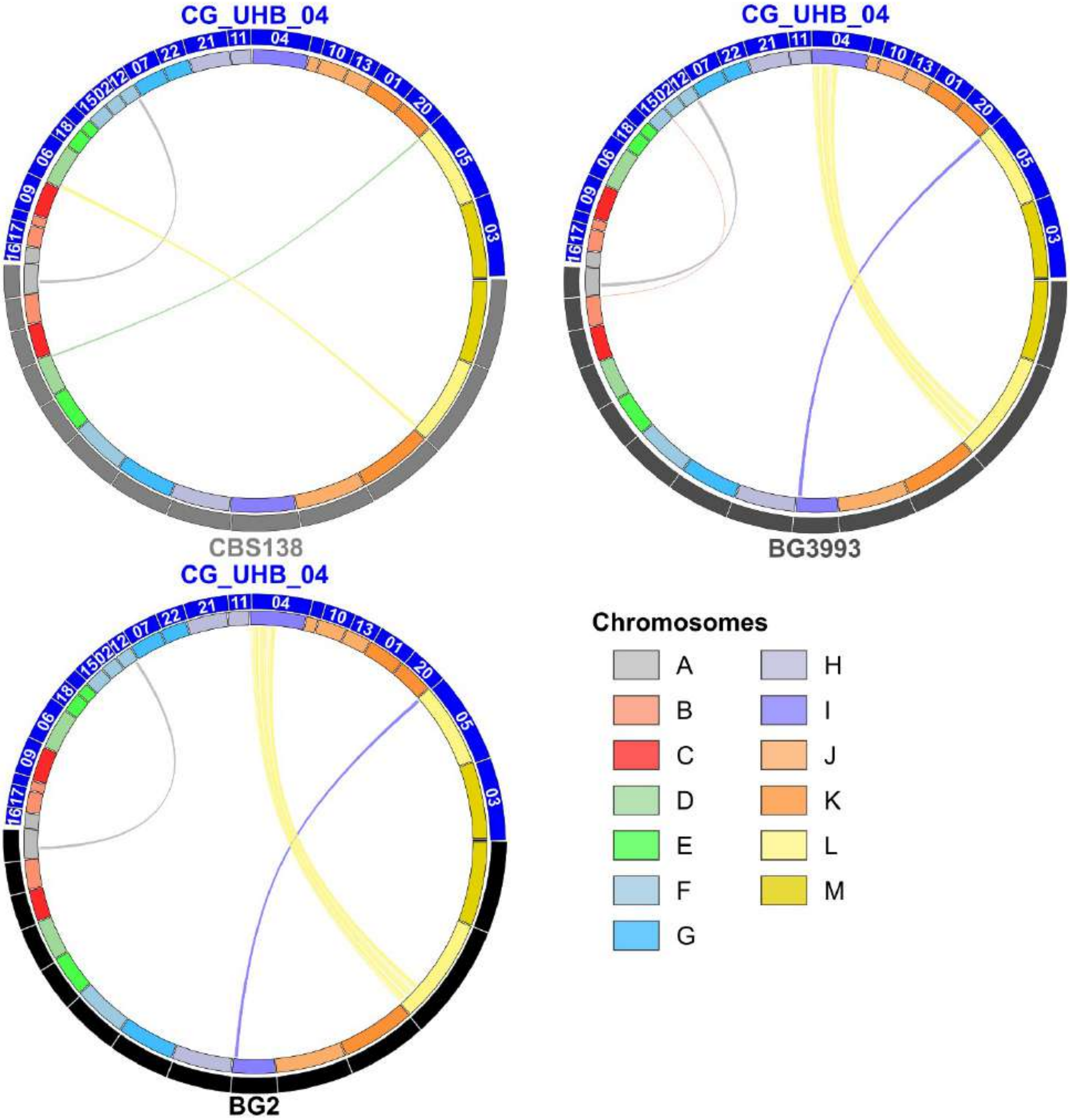

Supplementary information

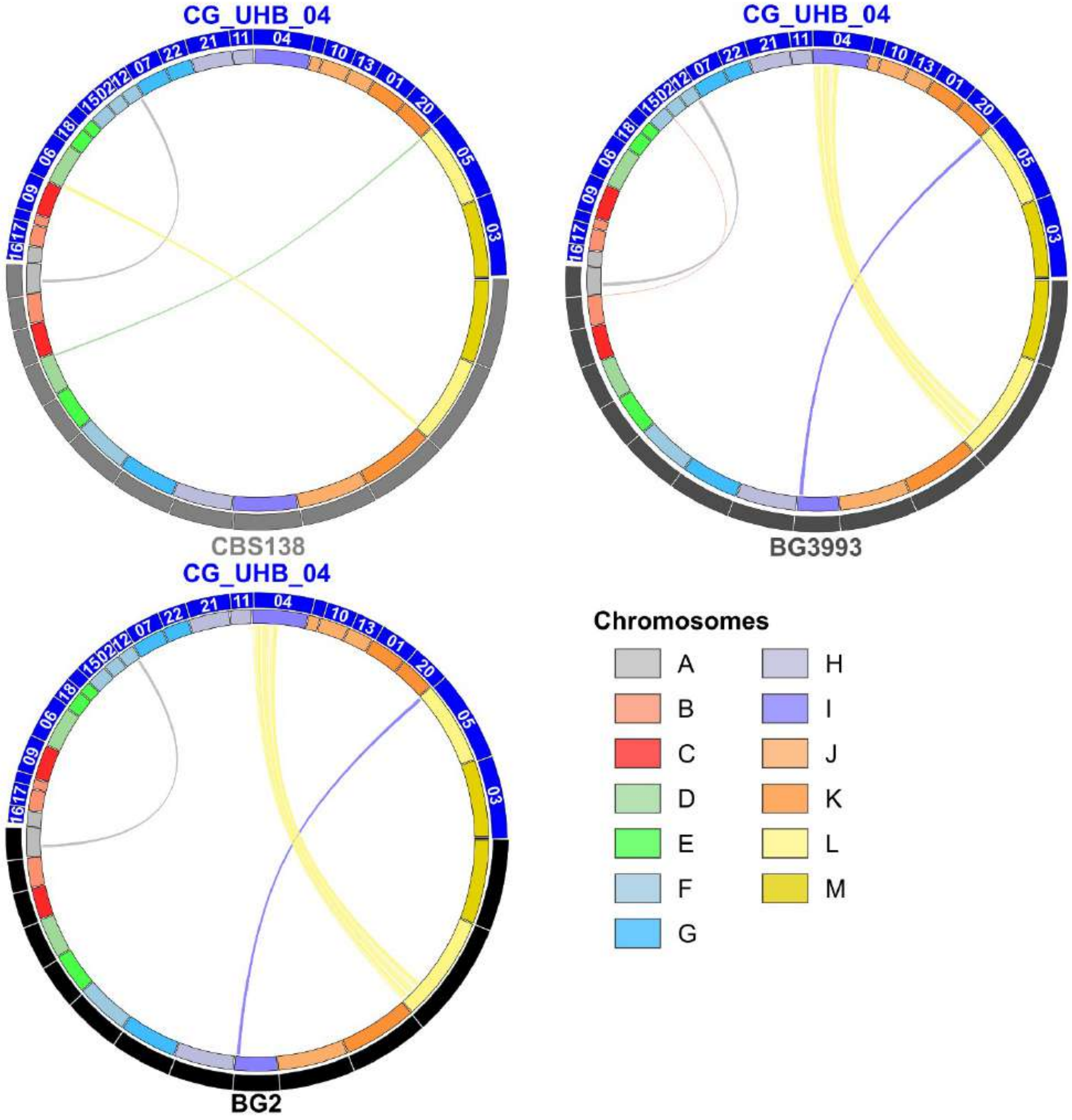

Supplementary information

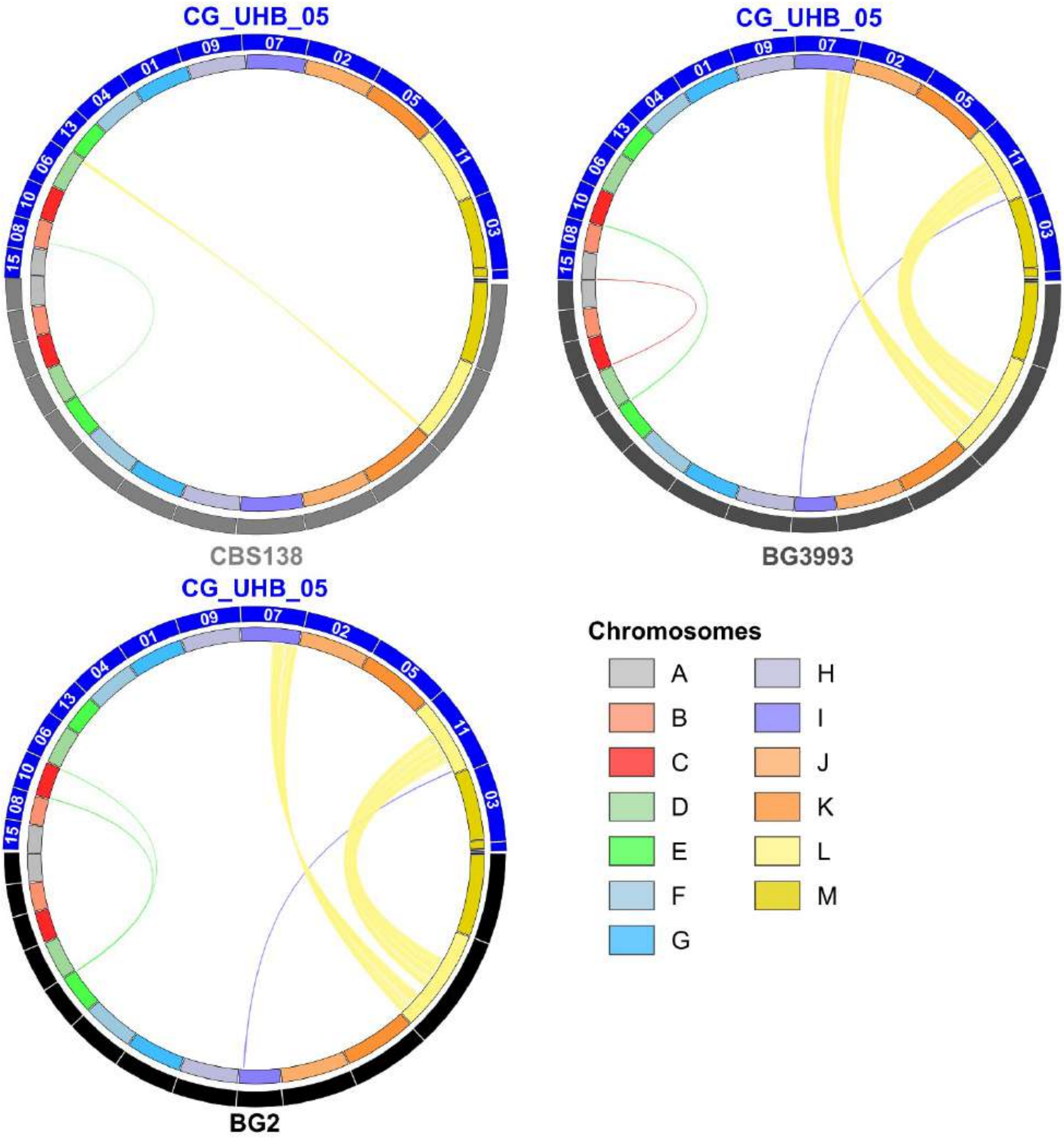

Supplementary information

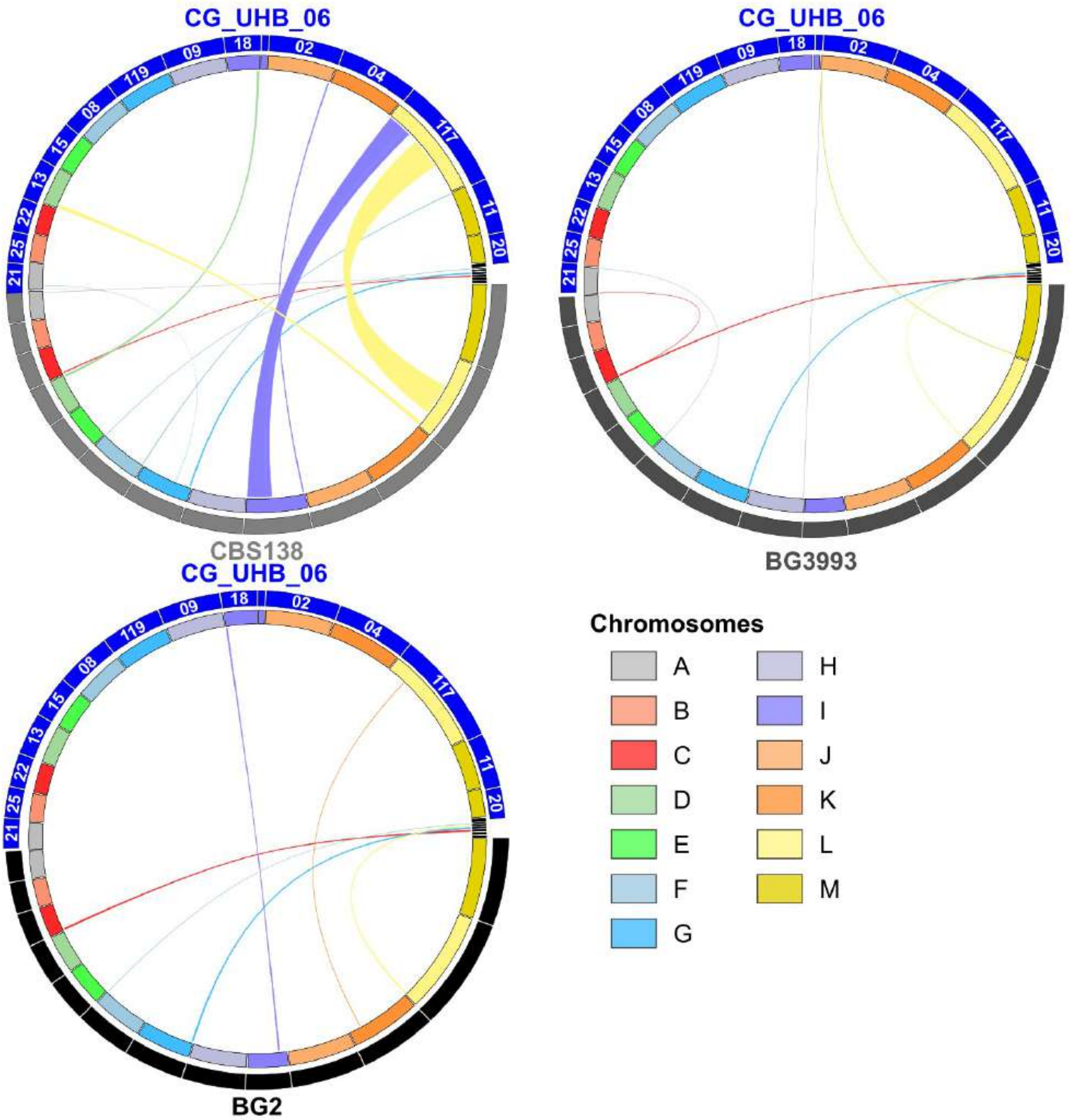

Supplementary information

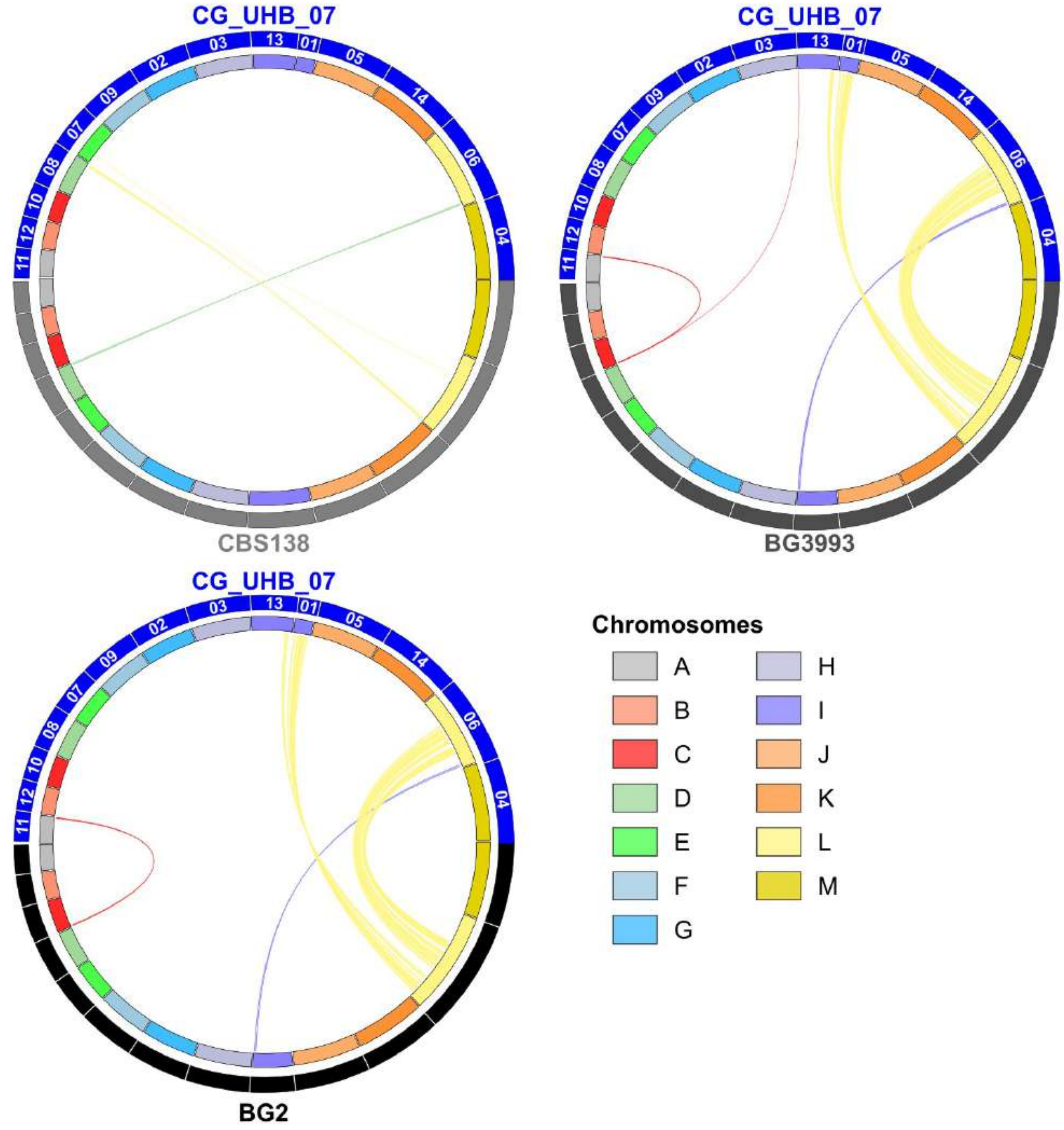

Supplementary information

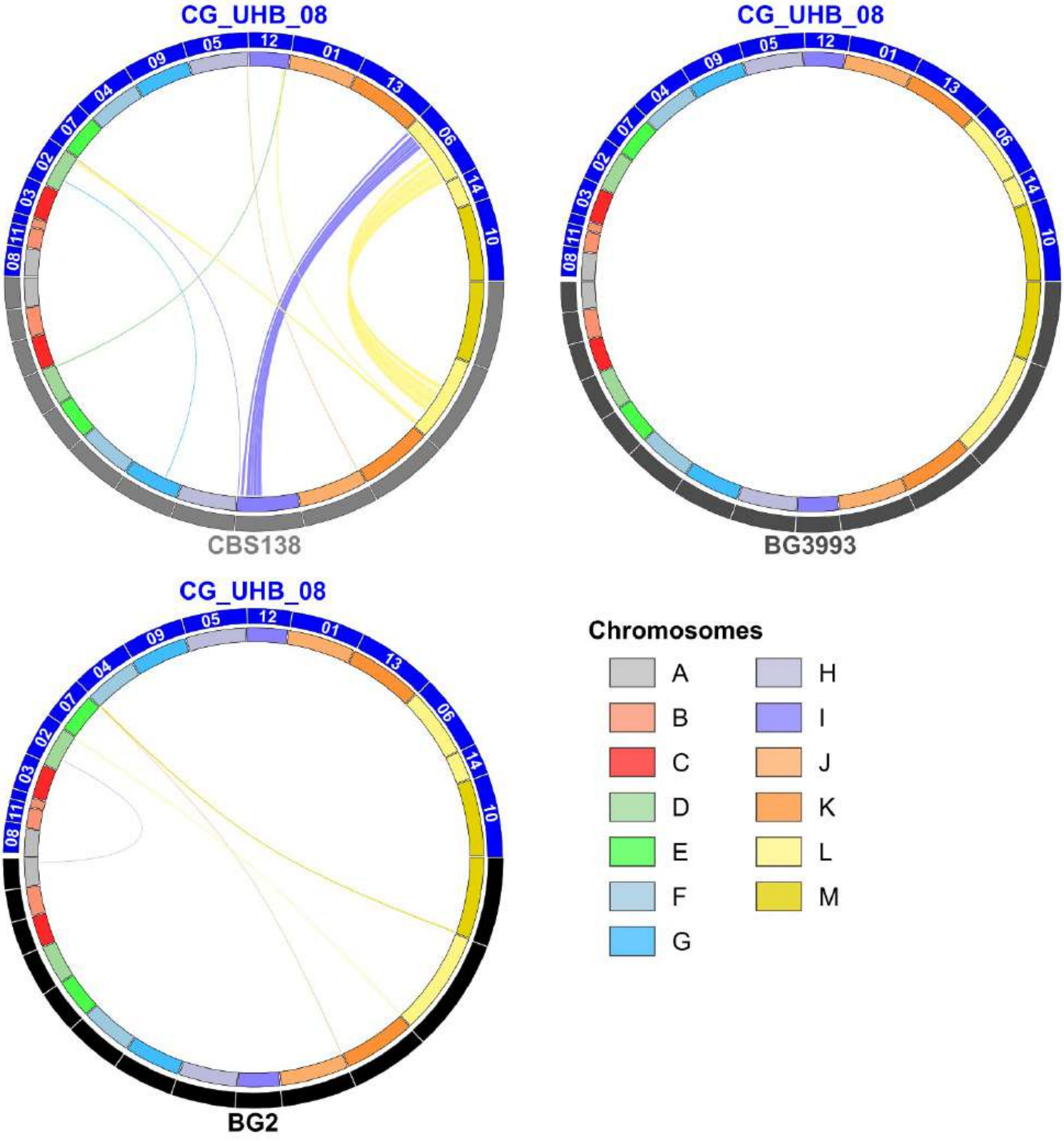

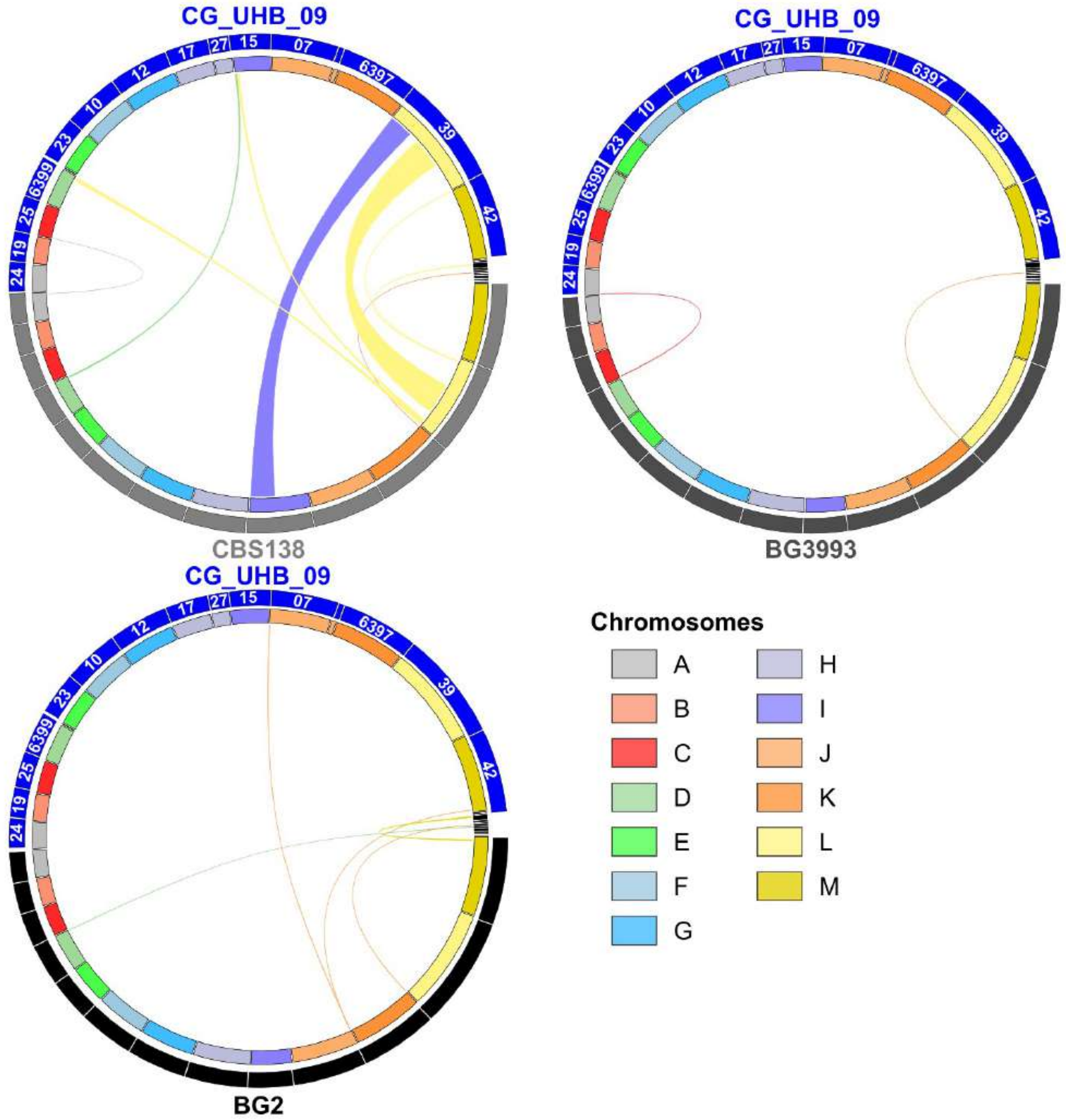

Back to the index

Supplementary information

Supplementary Figure 6

Phylogenetic tree of *Candida glabrata* isolates annotated with bootstrap values (100 iterations).

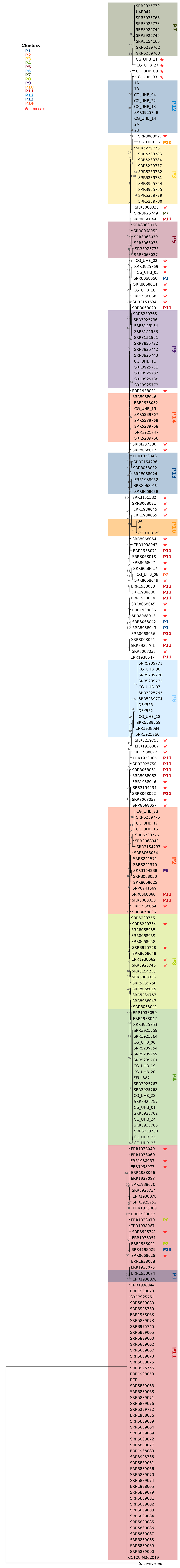

Supplementary figure 10: Phylogenetic tree of *Candida glabrata* isolates using *Saccharomyces cerevisiae* as the root of the tree, annotated with bootstrap support values calculated over 1000 bootstrap iterations.

Back to the index

Supplementary Figure 7

Comparison of Neighbor-Joining and Maximum Likelihood-based clustering obtained on the entire set of genetic variants among the studied genomes.

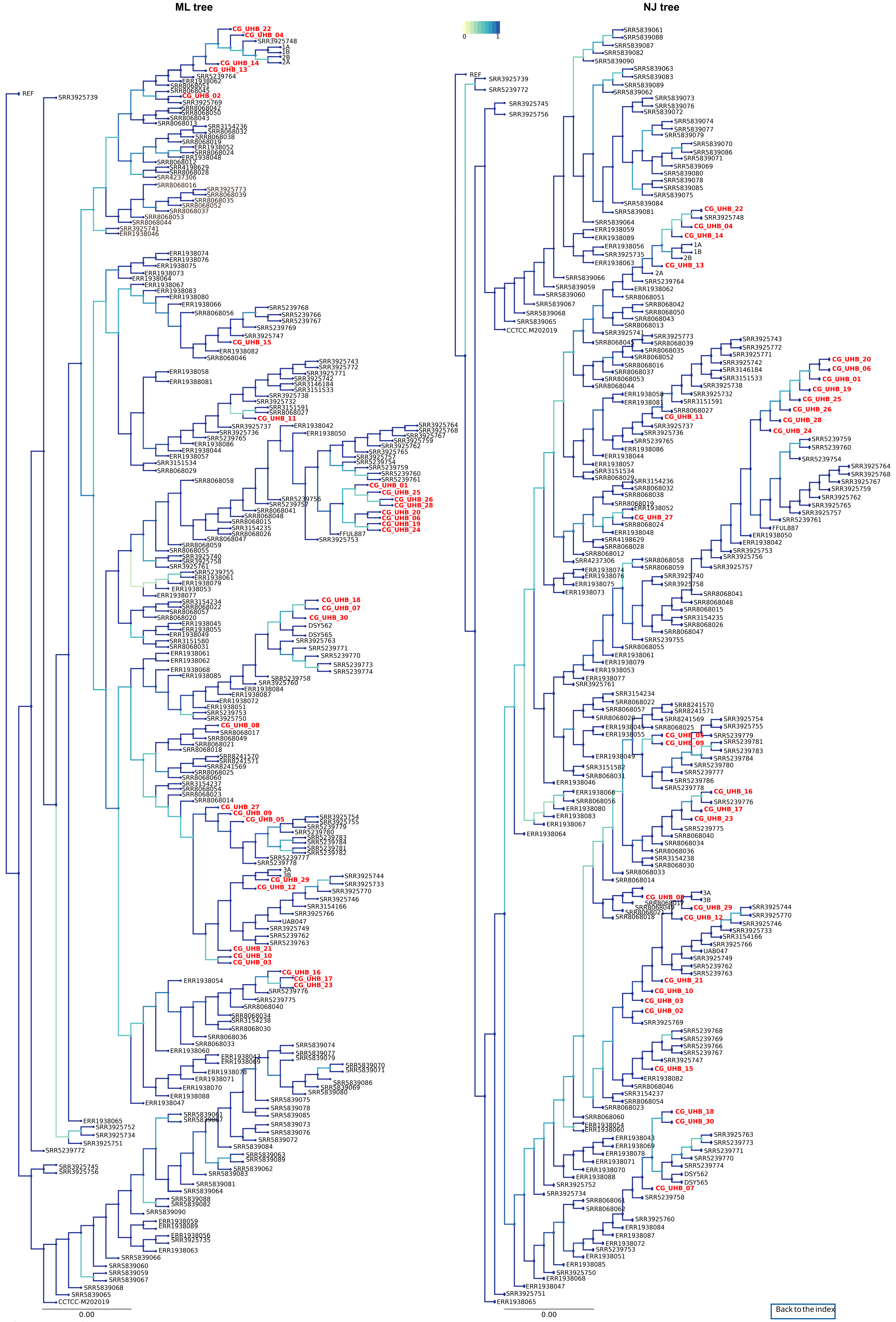

Back to the index

Supplementary Figure 7

Comparison of Neighbor-Joining and Maximum Likelihood based clusterings obtained on the entire set of genetic variants among the studied genomes. The comparison was carried out by using Phylo.io (Robinson et al. 2016). The yellow to blue color scheme (used to color the branches of the trees) indicates the similarity of best matching subtrees between the two trees.

Supplementary figure 8

Selection of the best partition of genomes into clades. The likelihood of cluster partitioning was carried out with the software fastStructure. The upper plot shows the log(likelihood) of each partitioning according to the results of the Markov chain Monte Carlo calculation. The best partitioning is identified as the lowest K maximizing the log(likelihood). The plot at the bottom shows the absolute value of the ΔK, an ad hoc quantity related to the second-order rate of change of the log probability of data with respect to the number of clusters. The highest local variation of the ΔK indicates the best partitioning. The red dots indicate the K identified as the best partitioning of clusters.

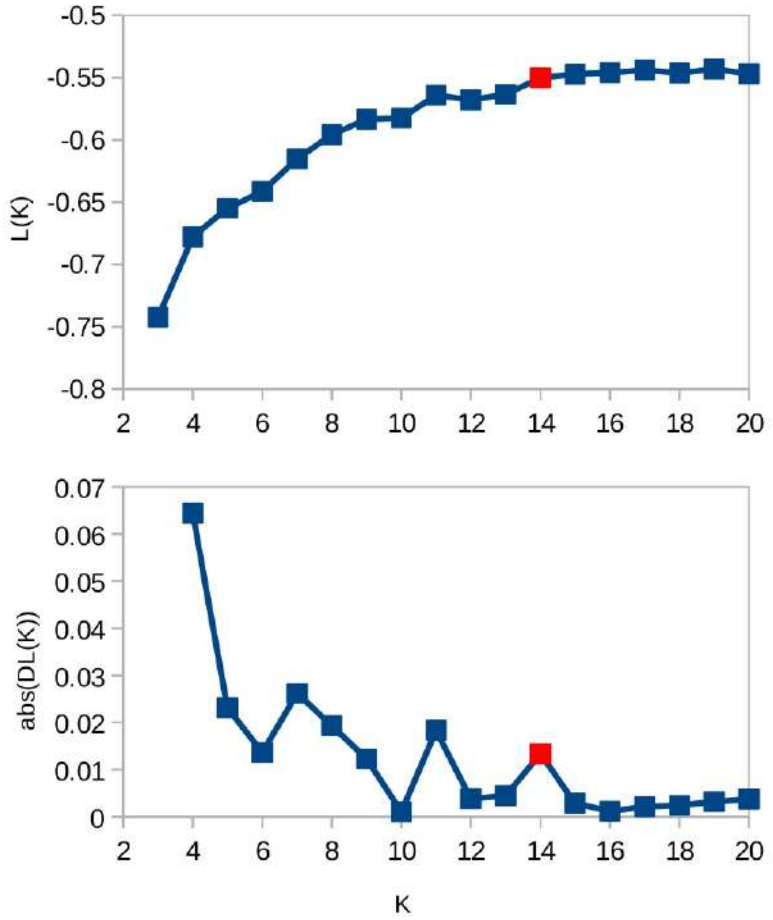

Back to the index

Supplementary figure 9

Phylogenetic tree of *Candida glabrata* isolates using *Saccharomyces cerevisiae* as the root of the tree. We inferred the phylogenetic relationships among strains compared to the close species *S. cerevisiae* based on the comparison of the sequence of four marker genes sufficient to recapitulate the genetic divergence within the Ascomycota phylum (Capella-Gutierrez et al. 2014): YHR186C, YMR012W, YJL029C, and YAR007C. The tree was drawn with the GrapeTree web tool (Zhou et al. 2018). Thanks to this analysis we were capable of positioning the root in the phylogenetic tree based on genomic variations (SNPs and indels) among *C. glabrata* strains (**Figure 2**).

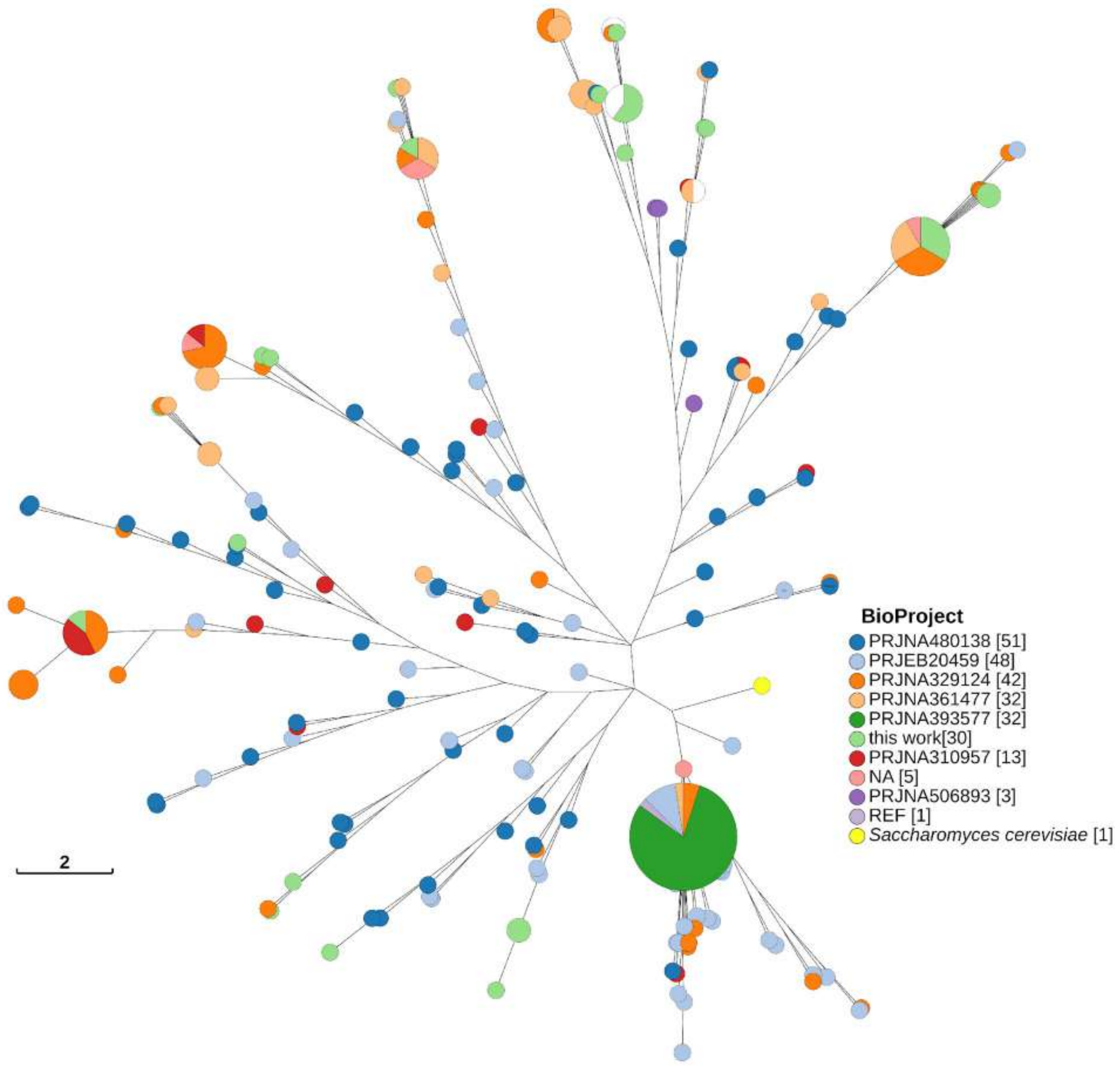

Back to the index

Supplementary Figure 10
Phylogenetic tree of *Candida glabrata* isolates using *Saccharomyces cerevisiae* as the root of the tree, annotated with bootstrap support values calculated over 1000 bootstrap iterations.

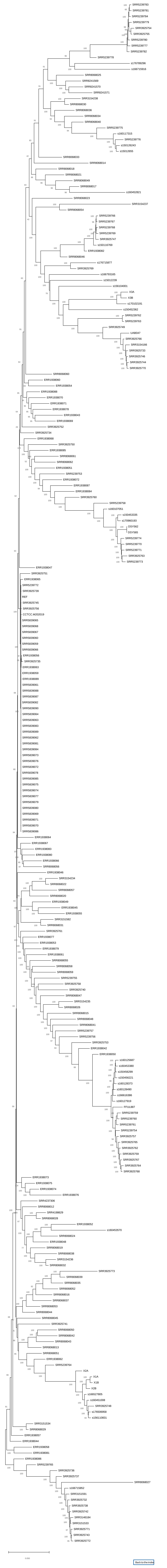

Back to the index

Supplementary figure 11

Phylogenetic trees of *Candida glabrata* isolates. Phylogenetic trees based on genomic distances among *C. glabrata* strains isolated over this and previous studies. Isolates are indicated as points colored according to a) the isolation source, b) the Country of isolation, and c) the collection date. The trees were drawn with the GrapeTree web tool (Zhou et al. 2018).

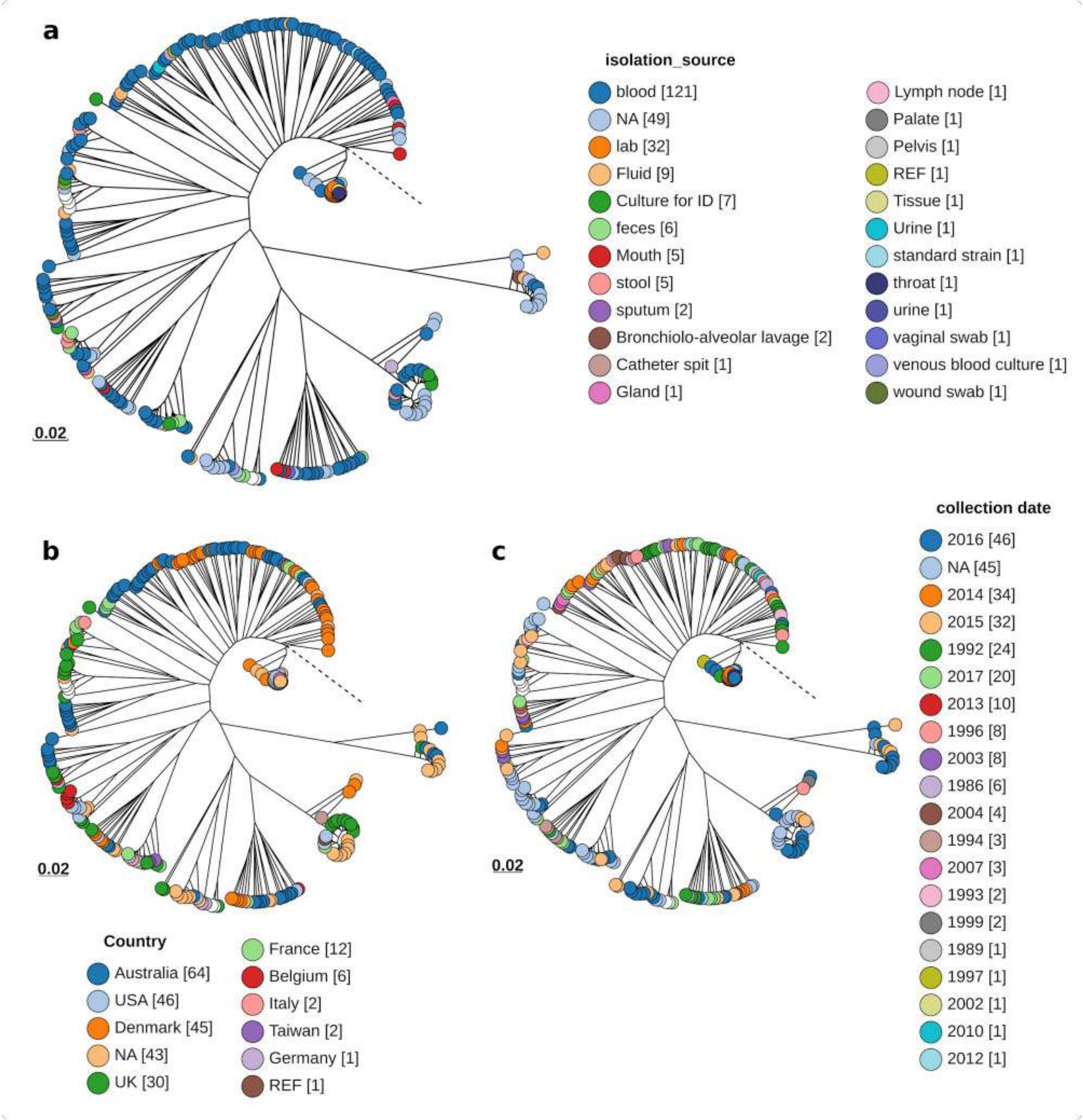

Back to the index

Supplementary figure 12

Comparison of genetic variants in the rearranged regions in genomes bearing the rearrangements and in genomes not bearing the rearrangements. a) percentage of SNPs present in the inverted region of chromosome L in genomes bearing the inversion (“yes”) or not (“no”); **b)** percentage of SNPs present in the region translocated from chromosome I to chromosome L in genomes bearing the inversion (“yes”) or not (“no”). Both contrasts were significantly different (Wilcox-Mann-Whitney $p<0.05$).

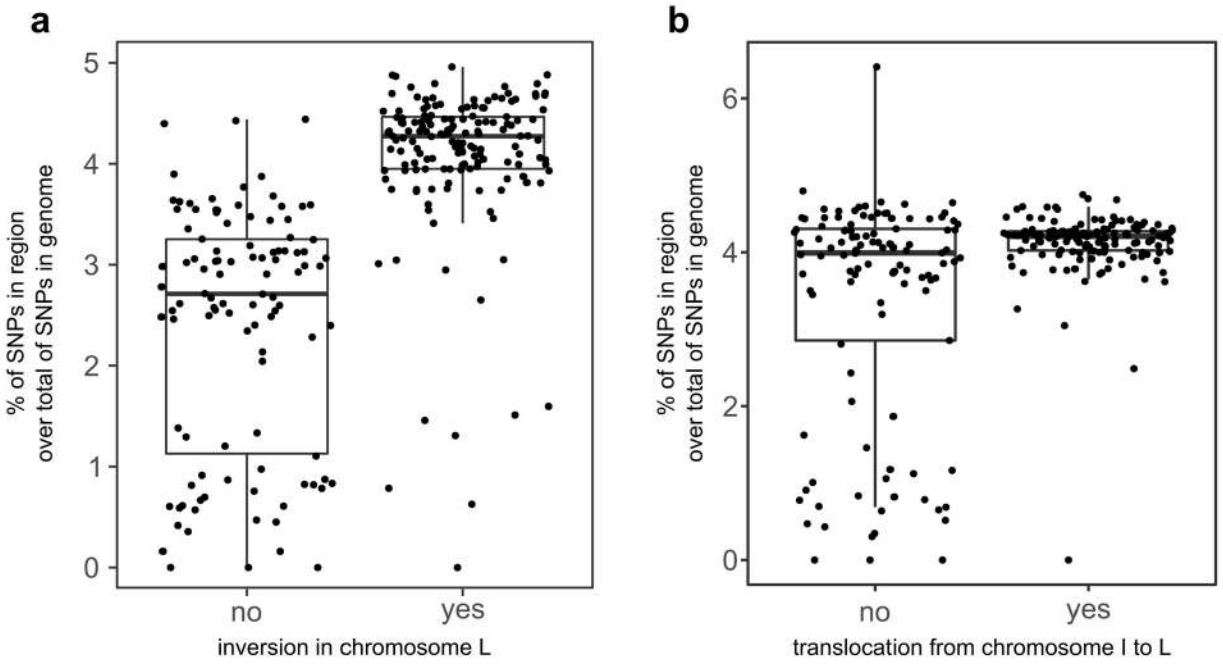

Back to the index

Supplementary figure 13

Comparison of phylogenetic trees of *Candida glabrata* isolates based on sets of genomic variants including or not including these located in the rearranged regions. Trees were drawn on the distances calculated on the entire set of genomic variations ("complete"), **a)** on the SNPs not including the inverted region of chromosome L, **b)** on the SNPs not including the region translocated between chromosome I and L, and **c)** on the SNPs not including the rearranged regions. Trees were drawn and compared with the online tool Phylo.io (Robinson et al. 2016).

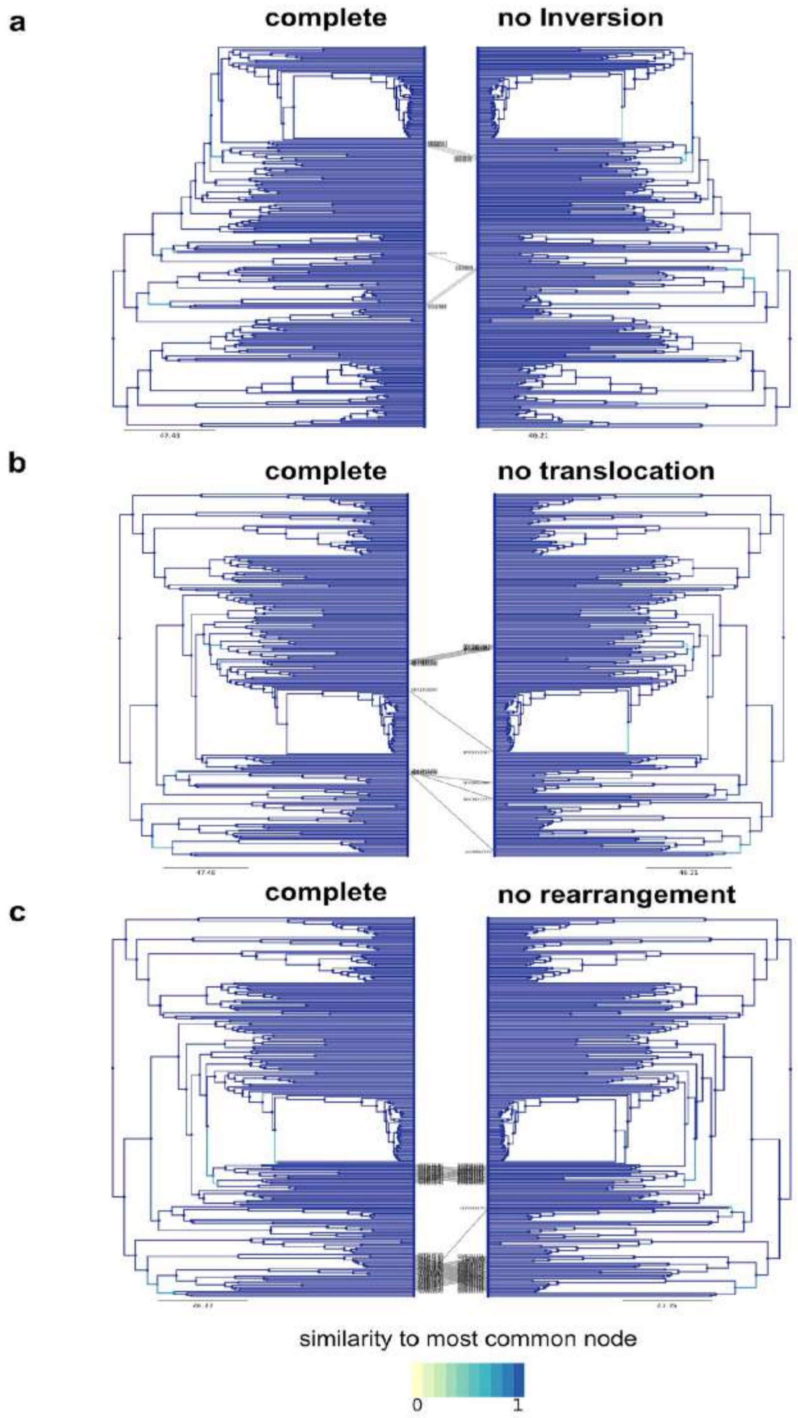

Back to the index

Supplementary figure 14

Coverage depth of sequenced strains. Coverage depths were calculated based on Illumina reads with the samtools depth function (Li et al. 2009). Depth of coverage was then calculated in sliding windows (size 5000bp, 1000bp sliding), then normalized by dividing each value by the geometric mean of the depth of coverage for the corresponding sequencing. *Mann-Whitney fdr<0.05.

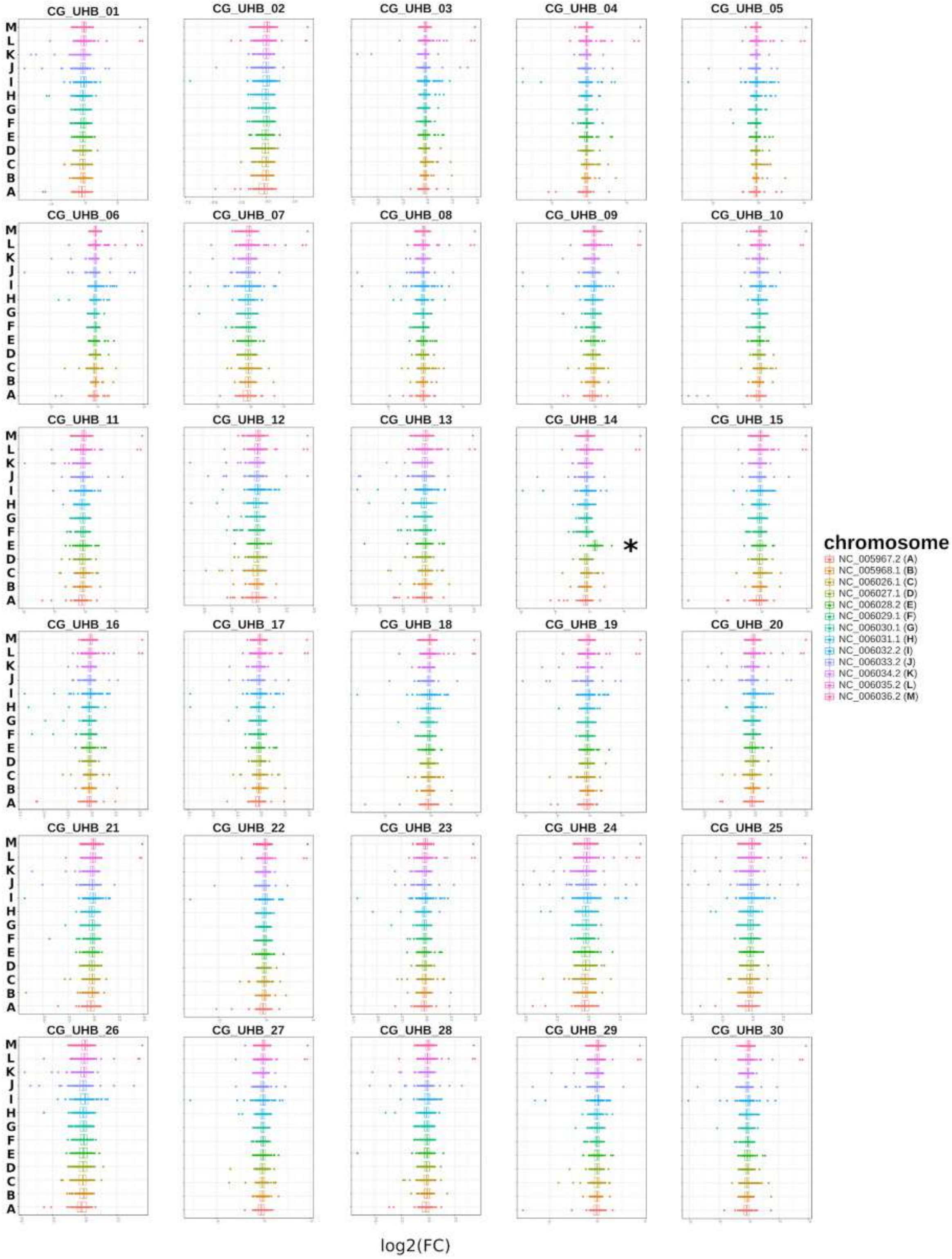

Back to the index

Supplementary Figure 15

Manhattan and QQ plots of GWAS analysis. A-C) QQ (left) and Manhattan (right) plots of the results of GWAS analyses on the three biological replicates for caspofungin MIC quantification. **D-F)** QQ (left) and Manhattan (right) plots of the results of GWAS analyses on the three biological replicates for fluconazole MIC quantification. **G-I)** QQ (left) and Manhattan (right) plots of the results of GWAS analyses on the two biological replicates for flucytosine MIC quantification. **J-L)** QQ (left) and Manhattan (right) plots of the results of GWAS analyses on the three biological replicates for voriconazole MIC quantification. NS= not significant (p-value>0.001).
Most of the plots show a p-value deflation, indicating that the effects of the loci are lower than expected from the normal distribution. This type of deviation is commonly expected in highly quantitative traits and related to the trait heritability, characteristics likely present in our dataset, and justifies the use of p-value thresholds more lenient than usual (Bian and Holland, 2017).

Supplementary information

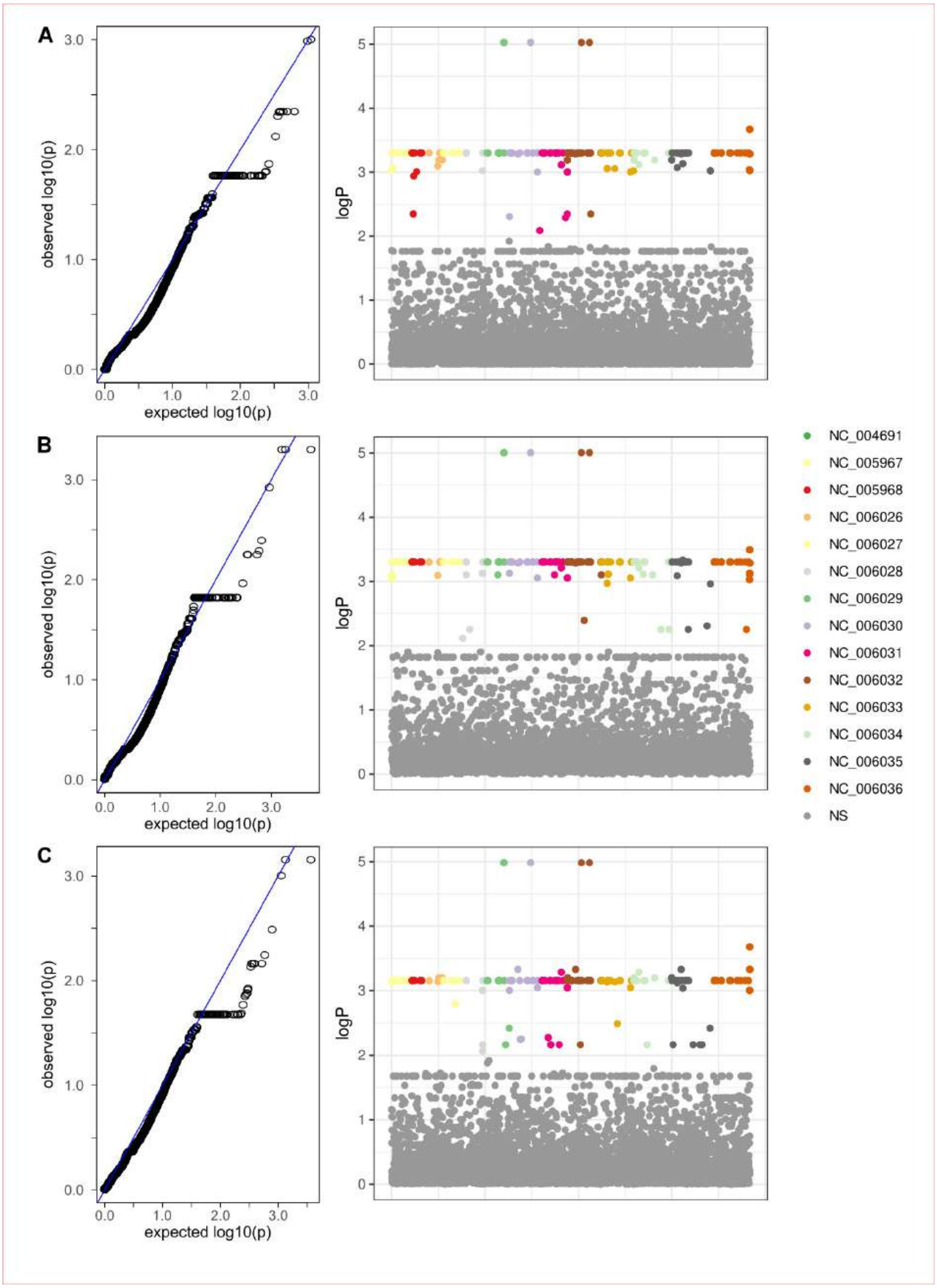

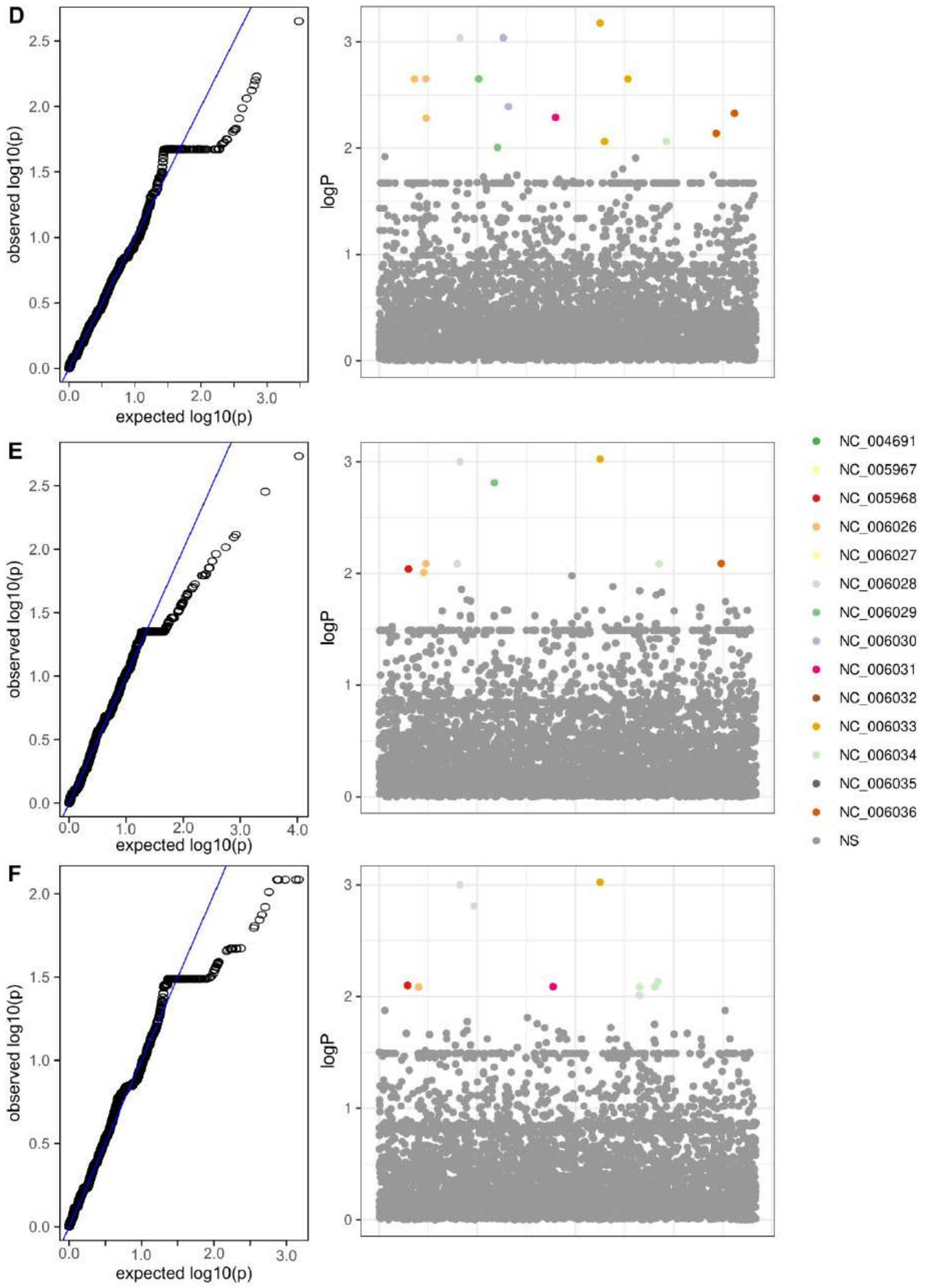
D
observed log10(p)
expected log10(p)
logP
E
F
NC_004691
NC_005967
NC_005968
NC_006026
NC_006027
NC_006028
NC_006029
NC_006030
NC_006031
NC_006032
NC_006033
NC_006034
NC_006035
NC_006036
NS

Supplementary information

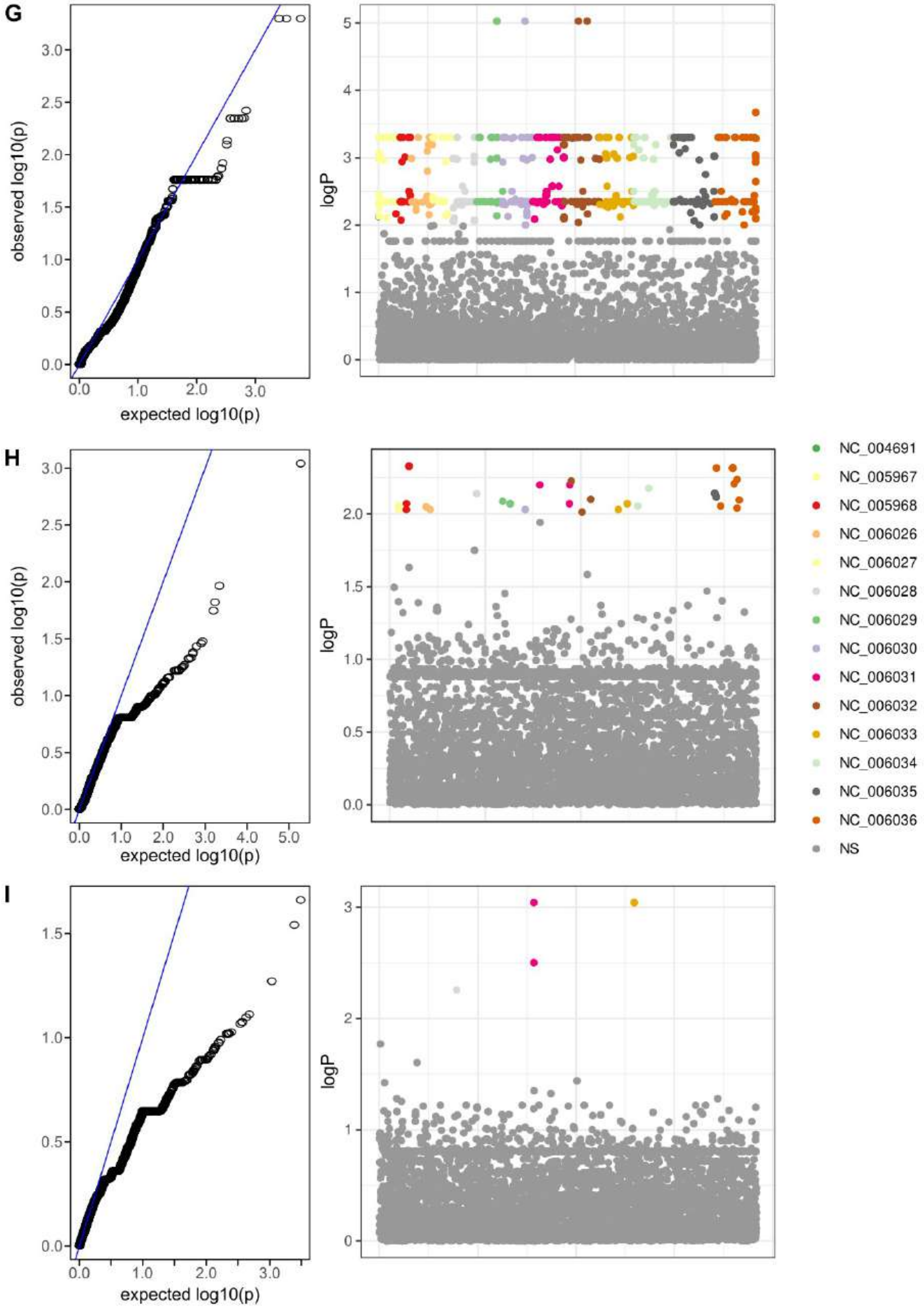

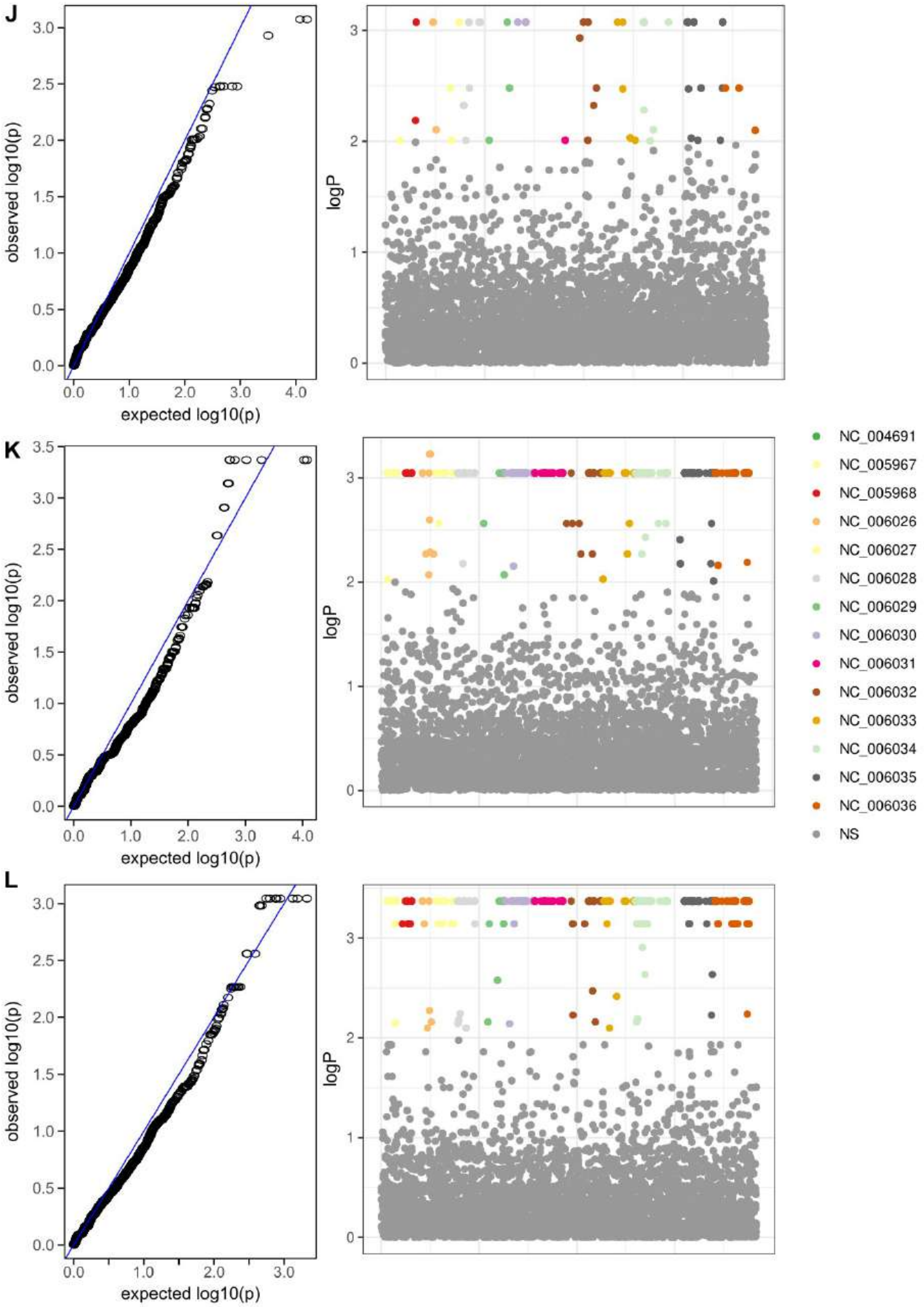

Back to the index

[Supplementary figure 16](#)

Alleles identified through GWAS analysis as associated with the response to caspofungin. For each locus, the variants found in the genomes of *Candida glabrata* strains sequenced over this study are shown. For each allele of each locus, the EC50 of strains bearing the corresponding allele are shown as points colored, as reported in the legend, according to the strain.

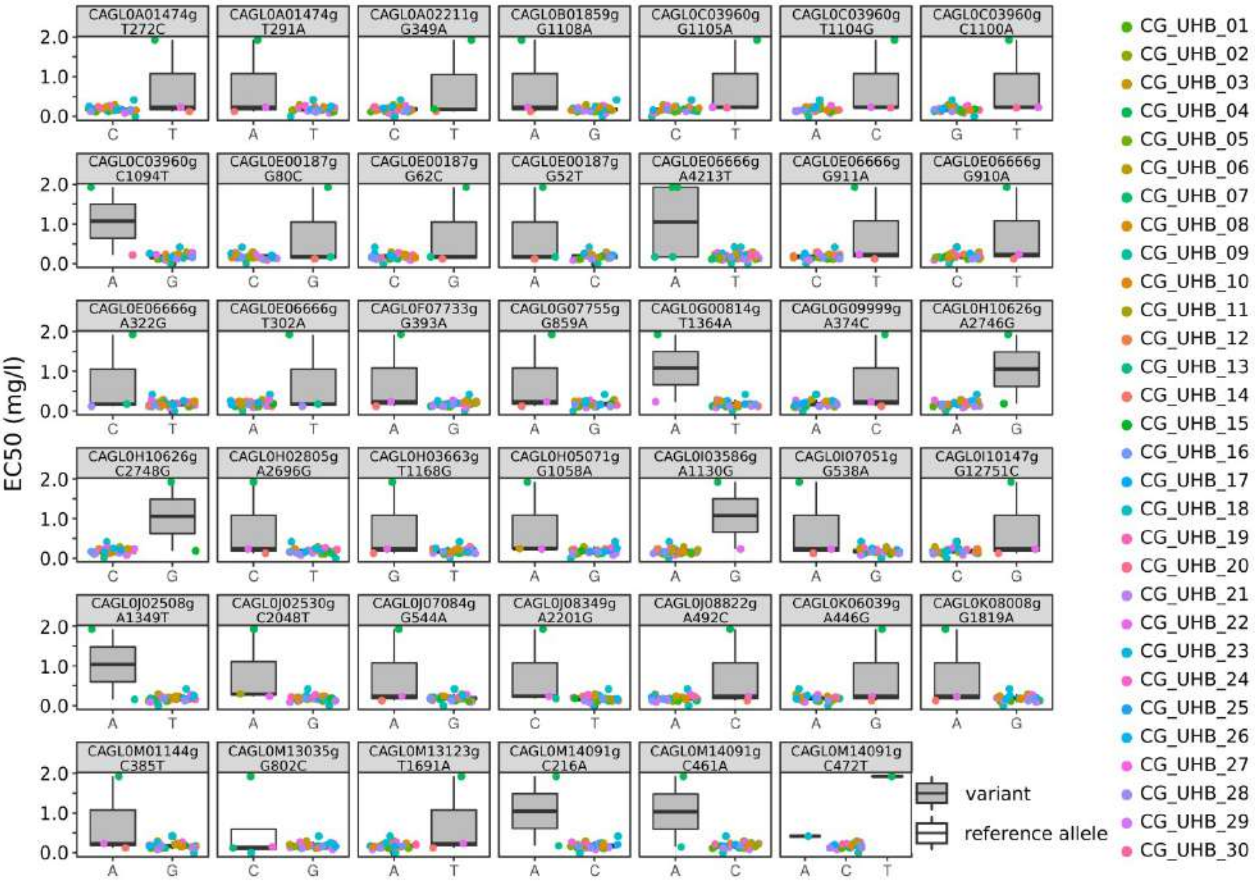

Back to the index

Supplementary figure 17

Loci identified through GWAS analysis as associated with the response to fluconazole. For each locus, the variants found in the genomes of *Candida glabrata* strains sequenced over this study are shown. For each allele of each locus, the EC50 of strains bearing the corresponding allele are shown as points colored, as reported in the legend, according to the strain.

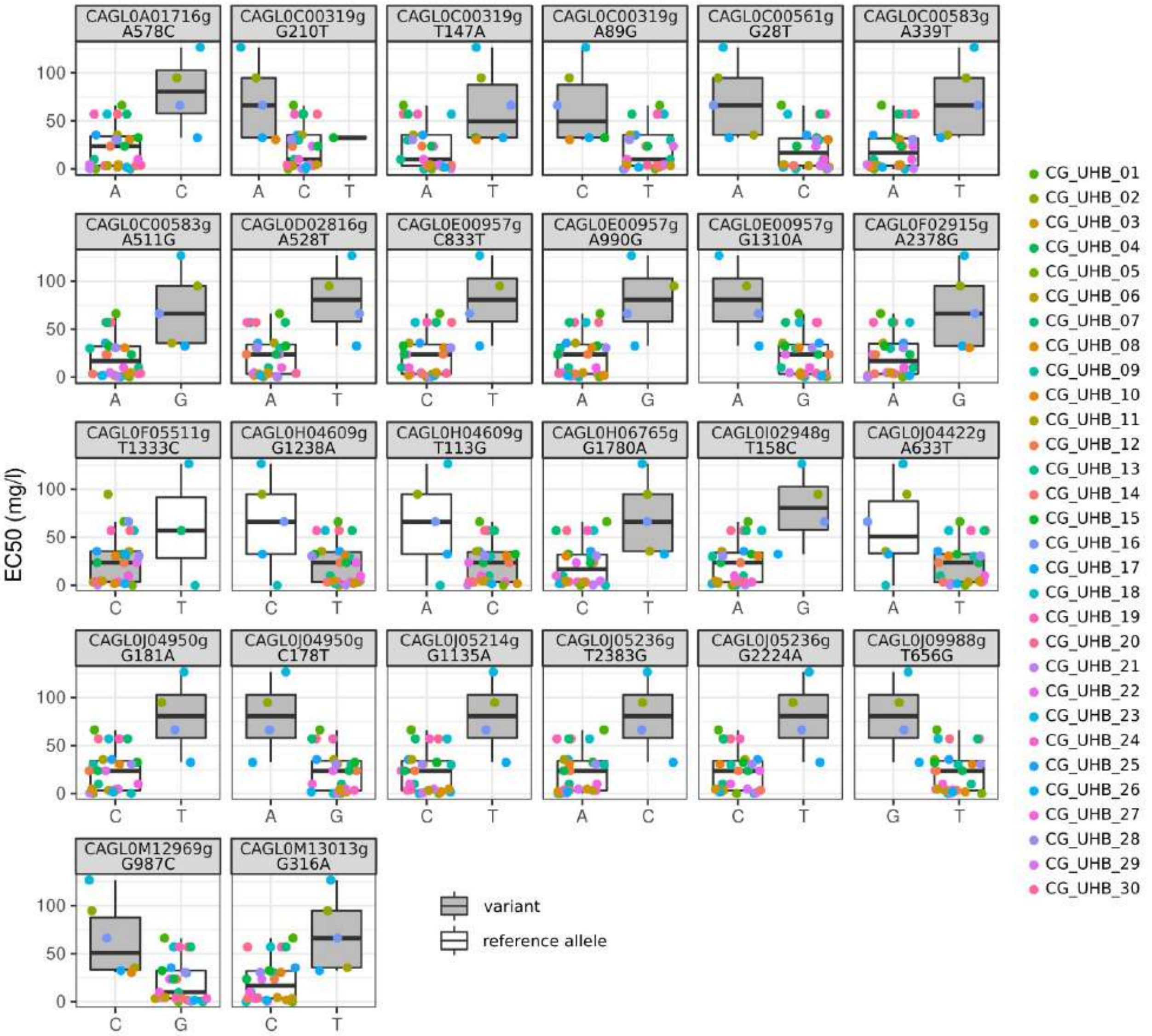

Back to the index

Supplementary figure 18

Alleles identified through GWAS analysis as associated with the response to voriconazole (part 1). For each locus, the variants found in the genomes of *Candida glabrata* strains sequenced over this study are shown. For each allele of each locus, the EC50 of strains bearing the corresponding allele are shown as points colored, as reported in the legend, according to the strain.

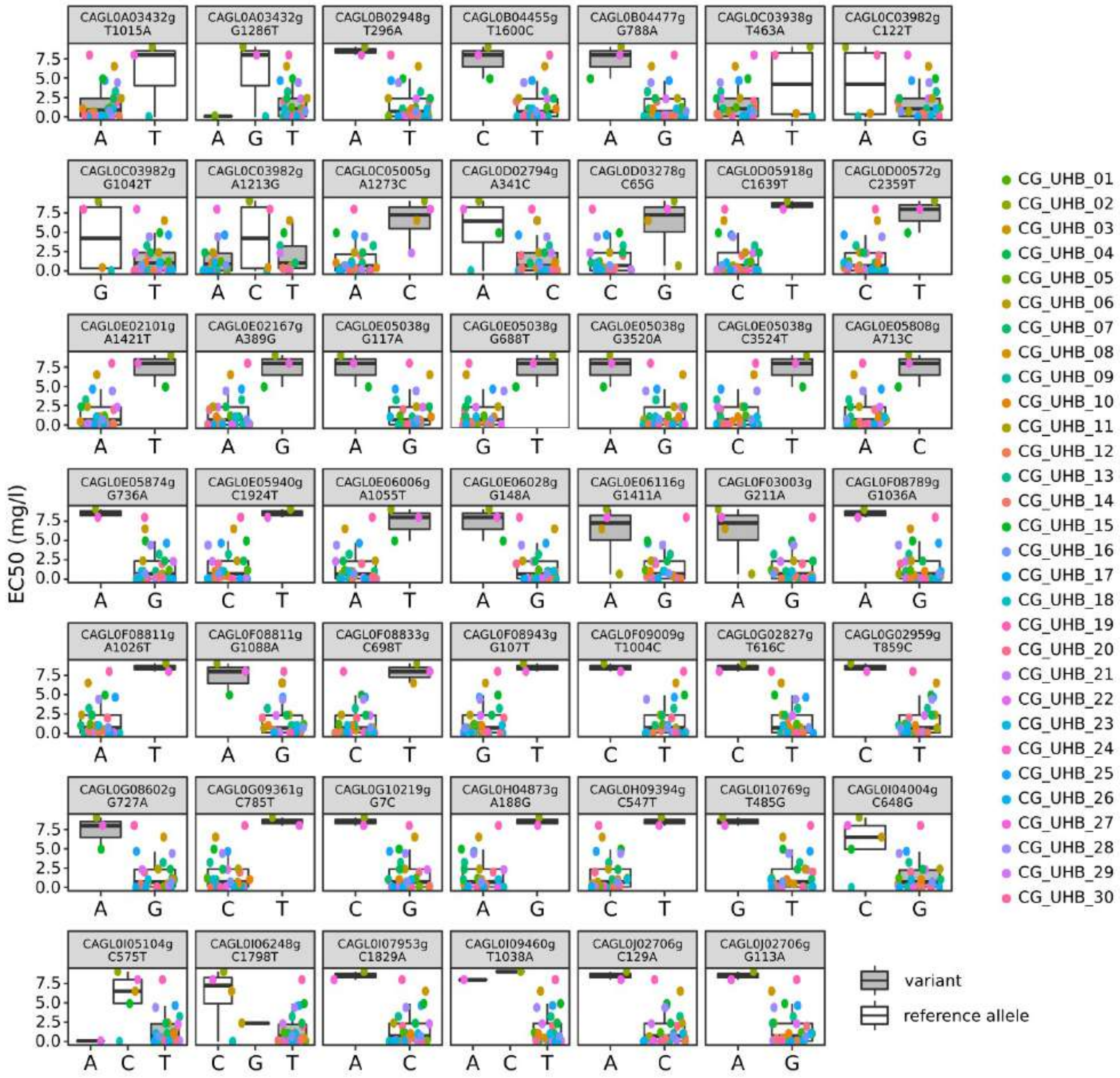

Supplementary figure 18

Alleles identified through GWAS analysis as associated with the response to voriconazole (part 2). For each locus, the variants found in the genomes of *Candida glabrata* strains sequenced over this study are shown. For each allele of each locus, the EC50 of strains bearing the corresponding allele are shown as points colored, as reported in the legend, according to the strain.

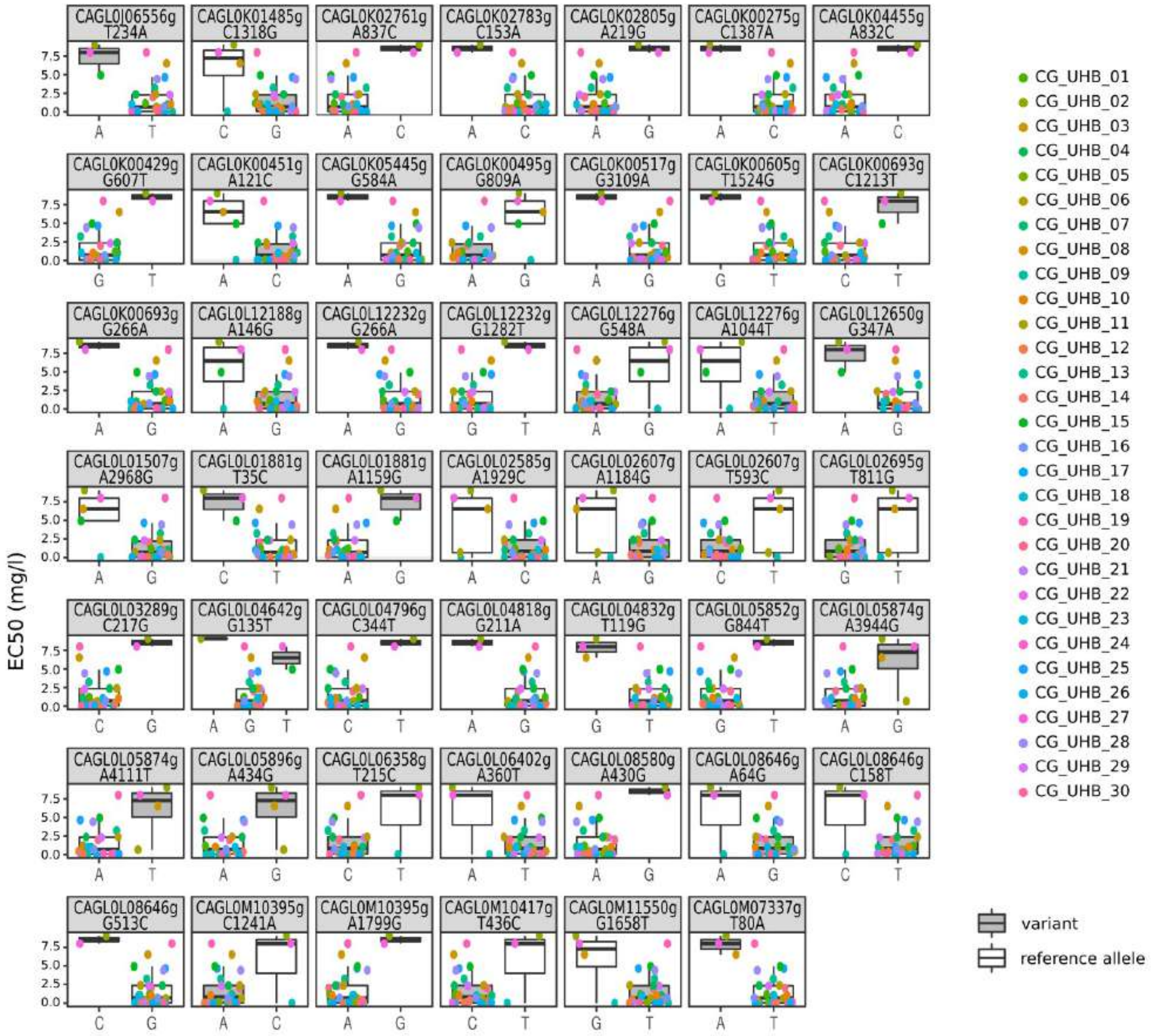

Back to the index

Supplementary figure 19

Alleles identified through GWAS analysis as associated with the response to flucytosine. For each locus, the variants found in the genomes of *Candida glabrata* strains sequenced over this study are shown. For each allele of each locus, the EC50 of strains bearing the corresponding allele are shown as points colored, as reported in the legend, according to the strain.

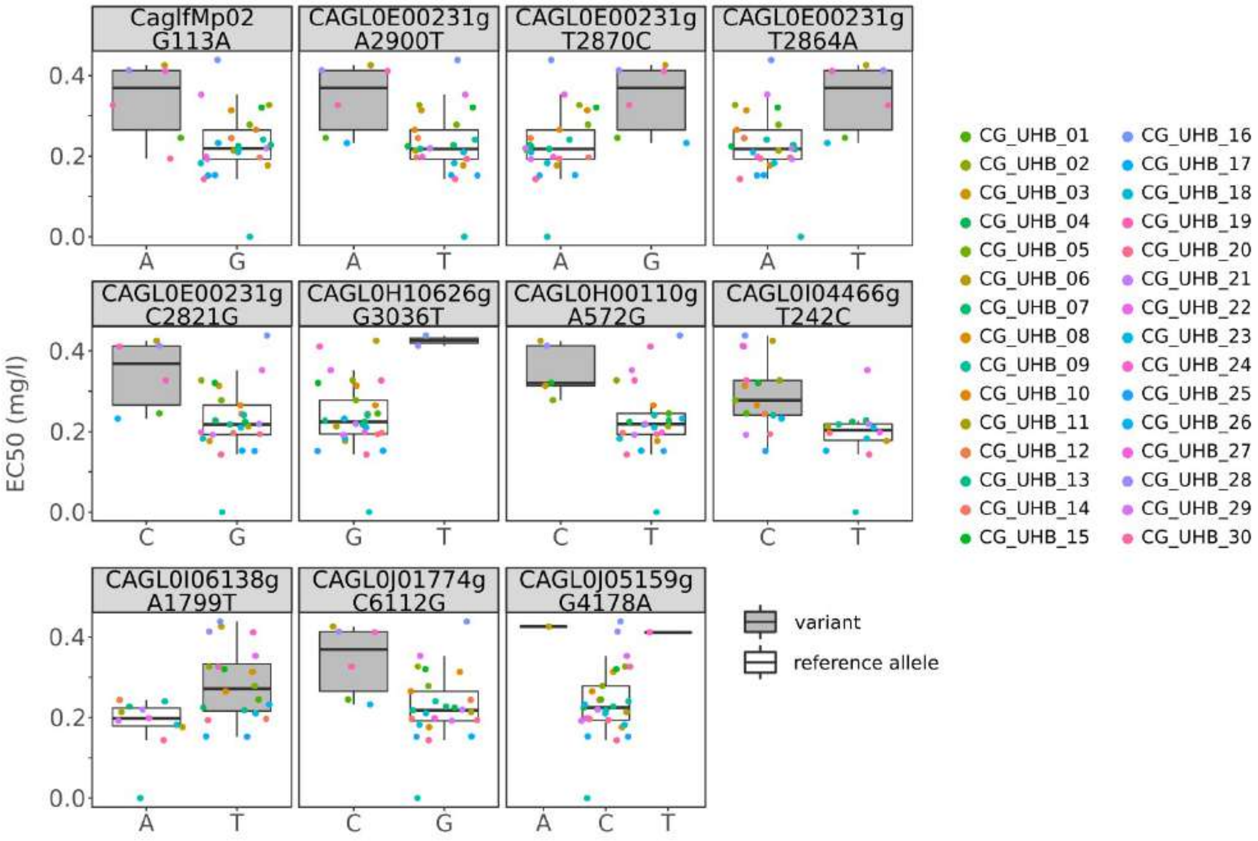

Back to the index

Supplementary figure 20

Number of missense SNPs per strain found to be associated with the response to the tested antifungals and relation with the response to the antifungal.

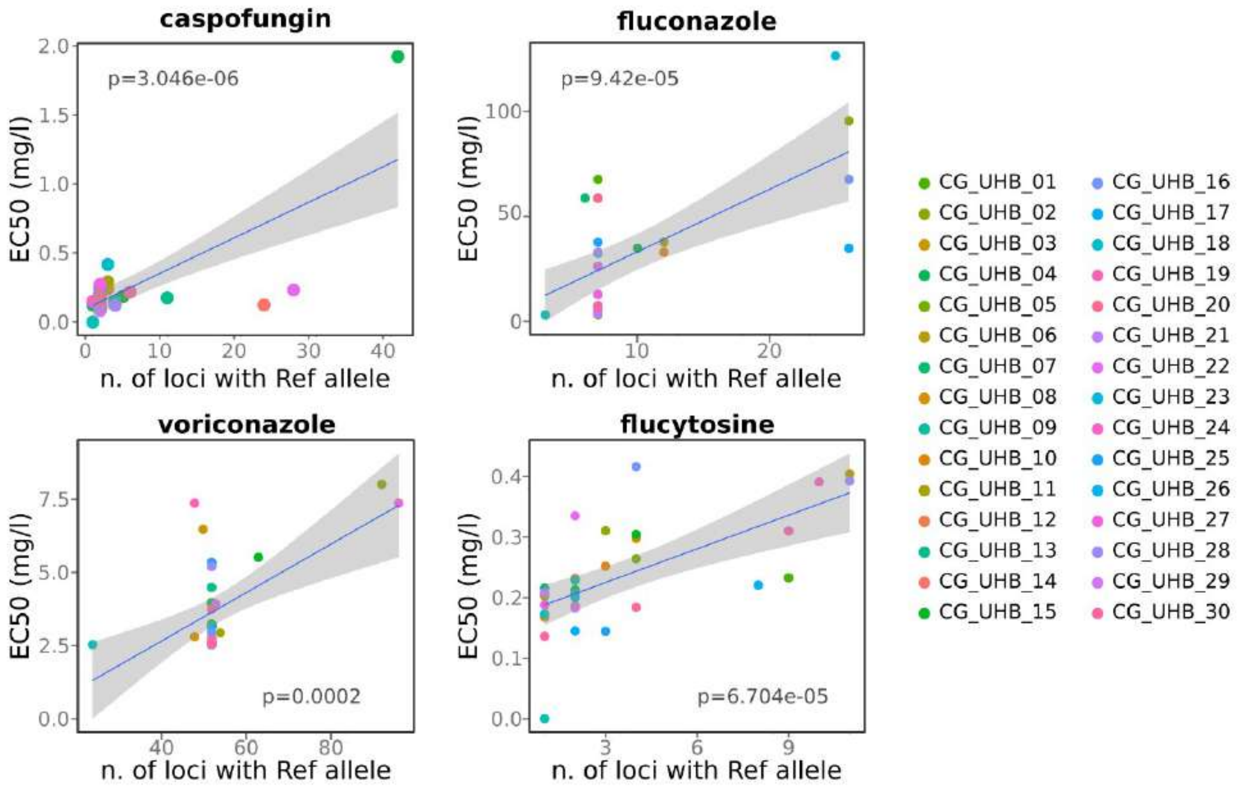

Back to the index

Supplementary information

[Supplementary figure 21](#)

Relations between the presence of chromosomal rearrangements and the response to the tested antifungals.

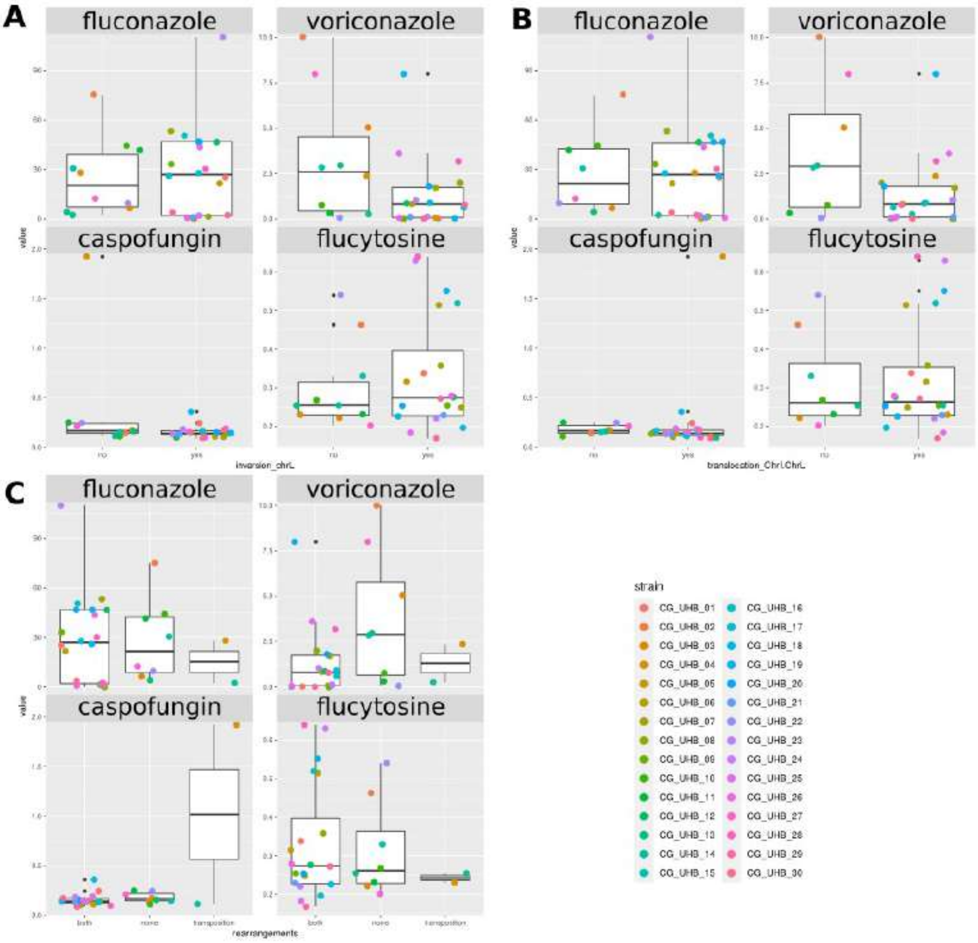

Back to the index

Supplementary information

Custom scripts

Custom Python and R script to generate syntheny plots from nucmer outputs. a) Python script generated to convert the nucmer output into an input suitable for R analysis. b) custom script to generate syntheny plots from the nucmer results modified with the custom Python script.

a. Python script

```
###usage: python script.py <nucmer coords output> <chromosome length file>
<fasta file contigs>

import operator
import sys
inputs=sys.argv

NucmerInFile=inputs[1]
NucmerIn=open(NucmerInFile)
NucmerInFileNew=NucmerInFile+"_forR"

Nucmerout=open(NucmerInFileNew,"w")
Nucmerout.write("AbsolStart1\tAbsolEnd1\tAbsolStart2\tAbsolEnd2\tS1\tE1\tS2\tE2\
tLEN1\tLEN2\tIDY\tTAG1\tTAG2\n")

ChromLengthFile=inputs[2]
ChromLength=open(ChromLengthFile)

ContigsFile=inputs[3]
Contigs=open(ContigsFile)
ContigsStart={}
ContigsFile_Coords=ContigsFile+"_coords"
ContigsFileOut=open(ContigsFile_Coords,"w")

spaceInFastaHeader=inputs[4]

newSeq=""
contig_name=""
first="yes"
ContigsLength={}
contigsList=[]
for conts in Contigs:
    if conts.startswith(">") and first=="yes":
        if spaceInFastaHeader=="yes":
            contig_name=conts[1:].split(' ')[0].strip()
        else:
            contig_name=conts[1:].strip()
        newSeq=""
        first="no"
        start=0
        contigsList.append(contig_name)
    elif conts.startswith(">") and first=="no":
        ContigsLength[contig_name]=len(newSeq)
        if spaceInFastaHeader=="yes":
            contig_name=conts[1:].split(" ")[0].strip()
        else:
            contig_name=conts[1:].strip()
        contigsList.append(contig_name)
        newSeq=""
    else:
```

```
        newSeq=newSeq+conts.strip()
ContigsLength[contig_name]=len(newSeq)

print(contigsList)
chromLengthDictStart={}
chromContigsAssoc={}
chromoList=[]

for line in ChromLength:
    if not line.startswith("#"):
        chromLengthDictStart[line.split("\t")[0]]=line.split("\t")[3]
        chromContigsAssoc[line.split("\t")[0]]={}
        if not line.split("\t")[0] in chromoList:
            chromoList.append(line.split("\t")[0])
        for contigs in range(0,len(contigsList)):
            chromContigsAssoc[line.split("\t")[0]][contigsList[contigs]]=0

contigChromAssoc={}
for contigs1 in range(0,len(contigsList)):
    contigChromAssoc[contigsList[contigs1]]={}
    for Chromos in range(0,len(chromoList)):
        contigChromAssoc[contigsList[contigs1]][chromoList[Chromos]]=0

for line1 in NucmerIn:
    if not line1.startswith("=") and not '[S1]' in line1 and not 
line1.startswith("/") and not line1.startswith("NUCMER")  and not 
line1.startswith("\n"):
        newLine=line1.replace(" | ","")
        newLine=newLine.replace("          ","\t")
        newLine=newLine.replace("         ","\t")
        newLine=newLine.replace("        ","\t")
        newLine=newLine.replace("       ","\t")
        newLine=newLine.replace("      ","\t")
        newLine=newLine.replace("     ","\t")
        newLine=newLine.replace("    ","\t")
        newLine=newLine.replace("   ","\t")
        newLine=newLine.replace("  ","\t")
        newLine=newLine.replace(" ","\t")
        RefChrom=newLine.split("\t")[8]
        Contig=newLine.split("\t")[9].strip()
        chromContigsAssoc[RefChrom][Contig]=int(chromContigsAssoc[RefChrom]
[Contig])+int(newLine.split("\t")[5])
        contigChromAssoc[Contig][RefChrom]=int(contigChromAssoc[Contig]
[RefChrom])+int(newLine.split("\t")[6])

sortedContigs=[]
for Chromos in range(0,len(chromoList)):
    sorted_chromContigsAssoc = 
sorted(chromContigsAssoc[chromoList[Chromos]].items(), 
key=operator.itemgetter(1))
    for matches in range(1,len(sorted_chromContigsAssoc)):
        ContigID=sorted_chromContigsAssoc[len(sorted_chromContigsAssoc)-matches]
[0]
        if 
max(contigChromAssoc[ContigID],key=contigChromAssoc[ContigID].get)==chromoList[C
hromos] and int(sorted_chromContigsAssoc[len(sorted_chromContigsAssoc)-matches]
[1])>10000 and not ContigID in sortedContigs:
            sortedContigs.append(ContigID)
```

```
for allContigs in range(0,len(contigsList)):
    if not contigsList[allContigs] in sortedContigs:
        sortedContigs.append(contigsList[allContigs])

newSeq=""
contig_name=""
first="yes"
ContigsStart={}
totLength=0
for ContigsAll in range(0,len(sortedContigs)):
    ContigsStart[sortedContigs[ContigsAll]]=totLength
    ContigsFileOut.write(sortedContigs[ContigsAll]+"\t"+str(totLength)+"\n")
    length=ContigsLength[sortedContigs[ContigsAll]]
    totLength=totLength+length

NucmerIn=open(NucmerInFile)
for line1 in NucmerIn:
    if not line1.startswith("=") and not '[S1]' in line1 and not 
line1.startswith("/") and not line1.startswith("NUCMER")  and not 
line1.startswith("\n"):
        newLine=line1.replace(" | ","")
        newLine=newLine.replace("          ","\t")
        newLine=newLine.replace("         ","\t")
        newLine=newLine.replace("        ","\t")
        newLine=newLine.replace("       ","\t")
        newLine=newLine.replace("      ","\t")
        newLine=newLine.replace("     ","\t")
        newLine=newLine.replace("    ","\t")
        newLine=newLine.replace("   ","\t")
        newLine=newLine.replace("  ","\t")
        newLine=newLine.replace(" ","\t")
        startCoordRef=newLine.split("\t")[1]
        endCoordRef=newLine.split("\t")[2]
        RefChrom=newLine.split("\t")[8]
        AbsoluteStart=chromLengthDictStart.get(RefChrom)
        newStart1=int(startCoordRef)+int(AbsoluteStart)
        newEnd1=int(endCoordRef)+int(AbsoluteStart)
        Contig=newLine.split("\t")[9].strip()
        startCoordContig=newLine.split("\t")[3]
        endCoordContig=newLine.split("\t")[4]
        chromContigsAssoc[RefChrom][Contig]=int(chromContigsAssoc[RefChrom]
[Contig])+int(newLine.split("\t")[5])
        contigChromAssoc[Contig][RefChrom]=int(contigChromAssoc[Contig]
[RefChrom])+int(newLine.split("\t")[6])
        AbsoluteStartContig=ContigsStart.get(Contig)
        newStart2=int(startCoordContig)+int(AbsoluteStartContig)
        newEnd2=int(endCoordContig)+int(AbsoluteStartContig)
        outLine=str(newStart1)+"\t"+str(newEnd1)+"\t"+str(newStart2)+"\
t"+str(newEnd2)+"\t"+newLine[1:]
        Nucmerout.write(outLine)
```

b. R script

```
library(ggplot2)
chromoInfo=read.table(reference_chromosome_length_file*,header=F,sep="\t")
head(chromoInfo)
colnames(chromoInfo)=c("chromoName","ChromoID","size","absoluteStart","absoluteE
nd")
```

```
contigs_coords=read.table(fasta_coords_nucmer_file,header=F,sep="\t")
Mummer_data=read.table(nucmer_file_converted,header=T,sep="\t")

ggplot(Mummer_data)+
geom_segment(aes(x=AbsolStart1,y=AbsolStart2,xend=AbsolEnd1,yend=AbsolEnd2,col=T
AG2),size=1)+
  theme_bw()+
  theme(panel.grid.major = element_blank(), panel.grid.minor = element_blank())+
  geom_vline(xintercept = chromoInfo$absoluteStart,col="gray",size=0.5)+
  geom_vline(xintercept = chromoInfo$absoluteEnd,col="gray",size=0.5)+
geom_hline(yintercept=as.numeric(as.character(contigs_coords$V2)),col="lightgray
",size=0.5)+
  xlab("chromosomes [reference]")+
  ylab("contigs [strain]")
```

```
*reference_chromosome_length_file
#CHROMO      CHROMO_NAME LENGTH      START_ABSOL END_ABSOL
NC_005967.2 A     491328      0     491328
NC_005968.1 B     502101      491329      993430
NC_006026.1 C     558804      993431      1552235
NC_006027.1 D     651701      1552236     2203937
NC_006028.2 E     687738      2203938     2891676
NC_006029.1 F     927101      2891677     3818778
NC_006030.1 G     992211      3818779     4810990
NC_006031.1 H     1050361     4810991     5861352
NC_006032.2 I     1100349     5861353     6961702
NC_006033.2 J     1195132     6961703     8156835
NC_006034.2 K     1302831     8156836     9459667
NC_006035.2 L     1455689     9459668     10915357
NC_006036.2 M     1402899     10915358    12318257
NC_004691.1 mito  20063 12318258    12338321
```

Back to the index

Supplementary information

Bibliography

- Bian, Y., Holland, J.B. (2017). Enhancing genomic prediction with genome-wide association studies in multiparental maize populations. Heredity (Edinb) 118(6):585-593. doi: 10.1038/hdy.2017.4
- Capella-Gutierrez, S., Kauff, F., Gabaldón, T. (2014) A phylogenomics approach for selecting robust sets of phylogenetic markers. Nucleic Acids Research 42(7):e54, doi: 10.1093/nar/gku071
- Krzywinski, M.I., Schein, J.E., Birol, I., Connors, J., Gascoyne, R., Horsman, D., Jones, S.J., Marra, M.A. (2009) Circos: An information aesthetic for comparative genomics. Genome Res. 19(9):1639-45, doi:10.1101/gr.092759.109.
- Nattestad, M., Schatz, M.C. (2016) Assemblytics: a web analytics tool for the detection of variants from an assembly. Bioinformatics. 32(19):3021-3. doi: 10.1093/bioinformatics/btw369.
- R Core Team (2020). R: A language and environment for statistical computing. R Foundation for Statistical Computing, Vienna, Austria. URL https://www.R-project.org/.
- Robinson, J.T., Thorvaldsdóttir, H., Winckler, W., Guttman, M., Lander, E.S., Getz, G., Mesirov, J.P. (2011) Integrative Genomics Viewer. Nature Biotechnology 29, 24–26 (2011).
- Robinson, O., Dylus, D., Dessimoz C. (2016) Phylo.io: Interactive Viewing and Comparison of Large Phylogenetic Trees on the Web. Mol Biol Evol 33(8):2163-2166, doi: 10.1093/molbev/msw080
- Warnes, G.R., Bolker, B., Bonebakker, L., Gentleman, R., Huber, W., Liaw, A., Lumley, T., Maechler, M., Magnusson, A., Moeller, S., Schwartz, M., Venables, B. (2020). gplots: Various R Programming Tools for Plotting Data. R package version 3.1.1. https://CRAN.R-project.org/package=gplots
- Wei T. and Simko V. (2017). R package "corrplot": Visualization of a Correlation Matrix (Version 0.84). Available from https://github.com/taiyun/corrplot
- Zhou, Z., Alikhan, N.F., Sergeant, M.J., Luhmann, N., Vaz, C., Francisco, A.P., Carriço, J.A., Achtman, M. (2018) GrapeTree: visualization of core genomic relationships among 100,000 bacterial pathogens. Genome Res. 28(9):1395-1404. doi: 10.1101/gr.232397.117.

Supplementary information

Supplementary Table 1
Strains analyzed in this study and sequencing information.

Table S1: details on isolates and genomic sequencing

Isolates. Details of strains analyzed over this study

Illumina sequencing. List of strains whose genomes were sequenced with Illumina HiSeq and details on the obtained reads.

Nanopore sequencing and assembly. information on Nanopore sequencing and assembly.

Compared genomes. Full list and metadata of compared *Candida glabrata* genomes (encompassing new genomes sequenced with Illumina over this study and publicly available genomes published previously).

Isolates

Raw Sequences were submitted in NCBI under the Bioproject accession number PRJNA589840.

Strain_ID	Sample type	Collection date	Sample location	Illumina sequencing	MinIOn sequencing	**NCBI BioSample Accession**	**SRA Accession Illumina**	**GenBank Accession**
CG_UHB_01	Hickman line Blood Culture	12-Dec-2015	PHE Birm (Heartlands)	yes	yes	SAMN18953774	SRR14381471	JAKCVZ000000000
CG_UHB_02	Sputum	31-Jan-2015	UHB	yes	yes	SAMN18953775	SRR14381470	JAKCVY000000000
CG_UHB_03	ascitic fluid	24-Nov-2016	UHB	yes	yes	SAMN18953776	SRR14381458	JAKCVX000000000
CG_UHB_04	drain	27-Mar-2017	UHB	yes	yes	SAMN18953777	SRR14381451	JAKCVW000000000
CG_UHB_05	sputum	02-Dec-2016	UHB	yes	yes	SAMN18953778	SRR14381447	JAKCVV000000000
CG_UHB_06	Culture for ID	09-Jun-2016	PHE Birm (Heartlands)	yes	yes	SAMN18953779	SRR14381446	JAKCVU000000000
CG_UHB_07	peripheral blood culture	20-Mar-2016	PHE Birm (Heartlands)	yes	yes	SAMN18953780	SRR14381445	JAKCVT000000000
CG_UHB_08	Culture for ID	10-May-2016	PHE Birm (Heartlands)	yes	yes	SAMN18953781	SRR14381444	JAKCVS000000000
CG_UHB_09	ascitic fluid	28-Mar-2017	UHB	yes	yes	SAMN18953782	SRR14381443	JAKCVR000000000
CG_UHB_10	peripheral blood culture	12-Oct-2015	PHE Birm (Heartlands)	yes	no	SAMN18953783	SRR14381442	NA
CG_UHB_11	BAL	30-Nov-2016	UHB	yes	no	SAMN18953784	SRR14381469	NA
CG_UHB_12	Fluid	28-Apr-2015	PHE Birm (Heartlands)	yes	no	SAMN18953785	SRR14381468	NA
CG_UHB_13	wound swab	03-Dec-2016	UHB	yes	no	SAMN18953786	SRR14381466	NA
CG_UHB_14	Culture for ID	18-Feb-2016	PHE Birm (Heartlands)	yes	no	SAMN18953787	SRR14381465	NA
CG_UHB_15	Blood Culture	28-Jul-2015	PHE Birm (Heartlands)	yes	no	SAMN18953788	SRR14381464	NA
CG_UHB_16	peripheral blood culture	02-Dec-2015	PHE Birm (Heartlands)	yes	no	SAMN18953789	SRR14381467	NA
CG_UHB_17	Blood Culture	02-Dec-2015	PHE Birm	yes	no	SAMN18953790	SRR14381463	NA

			(Heartlands)					
CG_UHB_18	Culture for ID	29-Sep-2015	PHE Birm (Heartlands)	yes	no	SAMN18953791	SRR14381462	NA
CG_UHB_19	Culture for ID	09-Nov-2015	PHE Birm (Heartlands)	yes	no	SAMN18953792	SRR14381461	NA
CG_UHB_20	Culture for ID	05-Nov-2015	PHE Birm (Heartlands)	yes	no	SAMN18953793	SRR14381460	NA
CG_UHB_21	Blood culture	05-Mar-2015	UHB	yes	no	SAMN18953794	SRR14381457	NA
CG_UHB_22	Blood culture	25-Jun-2015	UHB	yes	no	SAMN18953795	SRR14381456	NA
CG_UHB_23	venous blood culture	25-Jul-2016	PHE Birm (Heartlands)	yes	no	SAMN18953796	SRR14381459	NA
CG_UHB_24	peripheral blood culture	07-Dec-2016	PHE Birm (Heartlands)	yes	no	SAMN18953797	SRR14381454	NA
CG_UHB_25	central line blood culture	12-Dec-2016	PHE Birm (Heartlands)	yes	no	SAMN18953798	SRR14381455	NA
CG_UHB_26	central line blood culture	13-Dec-2016	PHE Birm (Heartlands)	yes	no	SAMN18953799	SRR14381453	NA
CG_UHB_27	Culture for ID	13-Jun-2016	PHE Birm (Heartlands)	yes	no	SAMN18953800	SRR14381452	NA
CG_UHB_28	Blood culture	21-Sep-2016	UHB	yes	no	SAMN18953801	SRR14381450	NA
CG_UHB_29	Blood Culture	24-Jan-2017	PHE Birm (Heartlands)	yes	no	SAMN18953802	SRR14381449	NA
CG_UHB_30	Fluid drain	11-Jan-2017	PHE Birm (Heartlands)	yes	no	SAMN18953803	SRR14381448	NA

Illumina sequencing. List of strains whose genomes were sequenced with Illumina HiSeq and details on the obtained reads. total reads= total reads after filtering; "Alignment %"= percentage of total reads mapping against the reference genome; "total SNPs/indels"= number of SNPs and indels found in the corresponding strain compared to the reference genome; depth of sequencing was calculated as (total number of reads * average read length) / total length of genome.

Strain_ID	Isolate name	NCBI BioSample Accession	NCBI Bioproject Accession	SRA Accession Illumina	Total reads	average length R1	average length R2	Alignment %	average depth of sequencing	total snps/indels
CG_UHB_01	15.0126687	SAMN18953774	PRJNA589840	SRR14381471	14,491,535	128.808	127.372	98.11	148.06	86,296
CG_UHB_02	17.6715877	SAMN18953775	PRJNA589840	SRR14381470	13,293,815	131.751	132.616	98.48	140.69	62,155
CG_UHB_03	16.6793165	SAMN18953776	PRJNA589840	SRR14381458	9,757,263	134.337	136.825	98.36	105.79	63,273
CG_UHB_04	17.6506958	SAMN18953777	PRJNA589840	SRR14381451	5,905,497	133.66	135.489	98.25	63.48	64,269
CG_UHB_05	16.6715916	SAMN18953778	PRJNA589840	SRR14381447	8,758,659	133.196	135.591	97.97	93.76	86,427
CG_UHB_06	16.045338	SAMN18953779	PRJNA589840	SRR14381446	10,127,137	134.462	135.681	97.62	108.56	85,750
CG_UHB_07	16.0107051	SAMN18953780	PRJNA589840	SRR14381445	2,296,314	133.603	131.031	97.03	23.97	71,542
CG_UHB_08	16.0452821	SAMN18953781	PRJNA589840	SRR14381444	9,817,139	136.093	139.559	97.88	107.67	85,483
CG_UHB_09	17.6789296	SAMN18953782	PRJNA589840	SRR14381443	13,192,480	131.669	133.746	98.36	140.00	85,722
CG_UHB_10	15.012239	SAMN18953783	PRJNA589840	SRR14381442	15,122,125	128.968	127.616	98.67	155.63	65,529
CG_UHB_11	16.6715852	SAMN18953784	PRJNA589840	SRR14381469	9,154,851	132.262	133.215	98.55	97.36	62,222
CG_UHB_12	15.0452362	SAMN18953785	PRJNA589840	SRR14381468	7,086,113	134.582	137.876	97.53	76.54	78,884
CG_UHB_13	16.6527805	SAMN18953786	PRJNA589840	SRR14381466	7,064,358	135.462	137.79	98.47	77.27	60,654
CG_UHB_14	16.0451008	SAMN18953787	PRJNA589840	SRR14381465	9,864,232	136.17	136.86	98.42	107.75	63,042
CG_UHB_15	15.0116769	SAMN18953788	PRJNA589840	SRR14381464	16,028,500	132.165	133.829	98.75	171.15	63,730
CG_UHB_16	15.0126243	SAMN18953789	PRJNA589840	SRR14381467	8,347,303	133.663	135.188	98.19	89.58	82,836
CG_UHB_17	15.0126255	SAMN18953790	PRJNA589840	SRR14381463	7,268,511	132.931	134.492	98.16	77.56	81,818
CG_UHB_18	15.0455335	SAMN18953791	PRJNA589840	SRR14381462	12,220,886	130.569	130.569	97.76	126.82	85,666
CG_UHB_19	15.0456221	SAMN18953792	PRJNA589840	SRR14381461	10,850,630	131.081	131.071	97.75	113.03	85,377

CG_UHB_20	15.0456299	SAMN18953793	PRJNA589840	SRR14381460	9,368,951	130.593	131.334	97.62	97.38	85,212
CG_UHB_21	15.6104001	SAMN18953794	PRJNA589840	SRR14381457	11,012,886	131.772	132.763	98.44	116.58	71,884
CG_UHB_22	15.6110831	SAMN18953795	PRJNA589840	SRR14381456	12,709,998	132.377	132.913	98.68	135.26	64,720
CG_UHB_23	16.0117315	SAMN18953796	PRJNA589840	SRR14381459	5,897,934	132.703	134.11	98.23	62.84	79,389
CG_UHB_24	16.0127919	SAMN18953797	PRJNA589840	SRR14381454	6,714,500	132.363	133.863	97.89	71.13	83,580
CG_UHB_25	16.0128373	SAMN18953798	PRJNA589840	SRR14381455	8,530,231	131.348	132.31	98.17	89.75	84760
CG_UHB_26	16.012849	SAMN18953799	PRJNA589840	SRR14381453	11,419,252	129.877	129.139	97.92	117.73	83,813
CG_UHB_27	16.045357	SAMN18953800	PRJNA589840	SRR14381452	10,991,506	132.852	133.491	98.33	117.02	72,757
CG_UHB_28	16.6616386	SAMN18953801	PRJNA589840	SRR14381450	13,759,297	132.829	133.412	98.18	146.20	84,138
CG_UHB_29	17.0102191	SAMN18953802	PRJNA589840	SRR14381449	10,891,772	132.939	133.77	98.26	116.03	83,180
CG_UHB_30	17.0450193	SAMN18953803	PRJNA589840	SRR14381448	7,066,203	131.57	131.849	97.53	73.80	85,263

Nanopore sequencing and assembly. information on Nanopore sequencing and assembly.

	CG_UHB_01				CG_UHB_02			
	Nanopore reads: 89,759				Nanopore reads: 79,787			
	Canu	Canu corrected	Minimap	Minimap corrected	Canu	Canu corrected	Minimap	Minimap corrected
# contigs (>= 0 bp)	19	19	14	14	16	16	17	17
# contigs (>= 1000 bp)	19	19	14	14	16	16	17	17
# contigs (>= 5000 bp)	18	18	14	14	16	16	16	16
# contigs (>= 10000 bp)	18	18	14	14	16	16	15	15
# contigs (>= 25000 bp)	14	14	14	14	15	15	14	14
# contigs (>= 50000 bp)	13	13	14	14	14	14	14	14
Total length (>= 0 bp)	12,615,368	12,657,694	12,687,630	12,593,896	12,623,690	12,656,330	12,424,109	12,353,006
Total length (>= 1000 bp)	12,615,368	12,657,694	12,687,630	12,593,896	12,623,690	12,656,330	12,424,109	12,353,006
Total length (>= 5000 bp)	12,610,439	12,652,750	12,687,630	12,593,896	12,623,690	12,656,330	12,419,413	12,348,341
Total length (>= 10000 bp)	12,610,439	12,652,750	12,687,630	12,593,896	12,623,690	12,656,330	12,413,563	12,342,516
Total length (>= 25000 bp)	12,553,271	12,595,548	12,687,630	12,593,896	12,608,377	12,641,007	12,403,215	12,332,207
Total length (>= 50000 bp)	12,526,491	12,568,726	12,687,630	12,593,896	12,572,988	12,605,590	12,403,215	12,332,207
# contigs	19	19	14	**14**	16	**16**	17	17
Largest contig	1,956,851	1,964,056	2,008,570	1,992,118	1,463,423	1,467,590	1,390,482	1,382,390
Total length	12,615,368	12,657,694	12,687,630	12,593,896	12,623,690	12,656,330	12,424,109	12,353,006
GC (%)	38.84	38.88	39.12	39.13	38.87	38.88	38.79	38.86
N50	1,237,151	1,241,336	1,244,701	**1,234,668**	1,137,053	**1,139,324**	1,088,063	1,081,332
N75	696,793	699,238	703,298	698,358	952,733	955,523	711,578	706,907
L50	4	4	4	4	5	5	5	5
L75	8	8	8	8	8	8	9	9
# N's per 100 kbp	0	0	0	0	0	0	0	0
Illumina Fold coverage				34				35
Nanopore Fold coverage			40				48	

	CG_UHB_03				CG_UHB_04			
	Nanopore reads: 90,411				Nanopore reads: 161,103			
	Canu	Canu corrected	Minimap	Minimap corrected	Canu	Canu corrected	Minimap	Minimap corrected
# contigs (>= 0 bp)	24	24	15	15	258	258	23	23
# contigs (>= 1000 bp)	24	24	15	15	258	258	23	23
# contigs (>= 5000 bp)	22	22	15	15	223	223	23	23
# contigs (>= 10000 bp)	21	21	14	14	185	185	23	23
# contigs (>= 25000 bp)	19	19	14	14	126	126	22	22
# contigs (>= 50000 bp)	15	15	14	14	59	59	22	22
Total length (>= 0 bp)	12,759,300	12,804,635	12,425,753	12,334,482	8,890,666	8,902,862	12,059,061	11,809,434
Total length (>= 1000 bp)	12,759,300	12,804,635	12,425,753	12,334,482	8,890,666	8,902,862	12,059,061	11,809,434
Total length (>= 5000 bp)	12,751,169	12,796,482	12,425,753	12,334,482	8,784,193	8,796,633	12,059,061	11,809,434
Total length (>= 10000 bp)	12,743,761	12,789,070	12,417,521	12,326,331	8,507,472	8,520,216	12,059,061	11,809,434
Total length (>= 25000 bp)	12,714,871	12,760,160	12,417,521	12,326,331	7,485,751	7,498,101	12,035,854	11,786,658
Total length (>= 50000 bp)	12,580,691	12,625,800	12,417,521	12,326,331	5,058,536	5,067,173	12,035,854	11,786,658
# contigs	24	24	15	**15**	258	258	23	**23**
Largest contig	1,442,756	1,448,131	1,437,347	1,427,272	176,394	176,335	1,497,485	1,465,877
Total length	12,759,300	12,804,635	12,425,753	12,334,482	8,890,666	8,902,862	12,059,061	11,809,434
GC (%)	38.98	39	38.76	38.81	38.4	38.35	40.58	40.23
N50	1,137,593	1,140,402	1,157,012	**1,147,541**	57,990	58,087	630,253	**616,564**
N75	702,871	705,347	706,673	701,938	33,495	33,548	451,043	441,167
L50	5	5	5	5	48	48	7	7
L75	9	9	9	9	99	99	12	12
# N's per 100 kbp	0	0	0	0	0	0	0	0
Illumina Fold coverage				27				15
Nanopore Fold coverage			48				44	

	CG_UHB_05				CG_UHB_06			
	Nanopore reads: 150,266				Nanopore reads: 23,779			
	Canu	Canu corrected	Minimap	Minimap corrected	Canu	Canu corrected	Minimap	Minimap corrected
# contigs (>= 0 bp)	29	29	17	17	22	22	53	53
# contigs (>= 1000 bp)	29	29	17	17	22	22	53	53
# contigs (>= 5000 bp)	27	27	16	16	22	22	53	53
# contigs (>= 10000 bp)	26	26	15	15	21	21	53	53
# contigs (>= 25000 bp)	21	21	14	14	20	20	51	51
# contigs (>= 50000 bp)	17	17	14	14	16	16	48	48
Total length (>= 0 bp)	12,675,375	12,740,611	12,424,109	12,337,459	12,645,259	12,710,449	11,919,786	11,919,786
Total length (>= 1000 bp)	12,675,375	12,740,611	12,424,109	12,337,459	12,645,259	12,710,449	11,919,786	11,919,786
Total length (>= 5000 bp)	12,670,333	12,735,548	12,419,413	12,332,814	12,645,259	12,710,449	11,919,786	11,919,786
Total length (>= 10000 bp)	12,662,565	12,727,762	12,413,563	12,327,007	12,639,789	12,704,936	11,919,786	11,919,786
Total length (>= 25000 bp)	12,570,367	12,635,369	12,403,215	12,316,715	12,618,629	12,683,731	11,887,599	11,887,599
Total length (>= 50000 bp)	12,425,720	12,490,459	12,403,215	12,316,715	12,496,059	12,560,946	11,769,021	11,769,021
# contigs	29	29	17	**17**	22	**22**	53	53
Largest contig	1,823,564	1,832,755	1,390,482	1,381,055	1,899,731	1,909,957	754,378	754,378
Total length	12,675,375	12,740,611	12,424,109	12,337,459	12,645,259	12,710,449	11,919,786	11,919,786
GC (%)	38.81	38.9	38.79	38.84	38.67	38.76	38.55	38.55
N50	1,052,062	1,058,020	1,088,063	**1,080,062**	998,264	**1,003,213**	336,329	336,329
N75	665,658	669,257	711,578	705,897	682,783	686,456	213,873	213,873
L50	5	5	5	5	5	5	14	14
L75	9	9	9	9	9	9	25	25
# N's per 100 kbp	0	0	0	0	0	0	0	0
Illumina Fold coverage				25				15
Nanopore Fold coverage			52				16	

	CG_UHB_07				CG_UHB_08			
	Nanopore reads: 86,597				Nanopore reads: 56,097			
	Canu	Canu corrected	Minimap	Minimap corrected	Canu	Canu corrected	Minimap	Minimap corrected
# contigs (>= 0 bp)	20	20	14	14	17	17	15	15
# contigs (>= 1000 bp)	20	20	14	14	17	17	15	15
# contigs (>= 5000 bp)	16	16	14	14	17	17	15	15
# contigs (>= 10000 bp)	16	16	14	14	17	17	15	15
# contigs (>= 25000 bp)	14	14	14	14	17	17	15	15
# contigs (>= 50000 bp)	14	14	14	14	13	13	15	15
Total length (>= 0 bp)	12,669,334	12,702,247	12,670,119	12,580,045	12,781,699	12,818,212	12,535,127	12,468,906
Total length (>= 1000 bp)	12,669,334	12,702,247	12,670,119	12,580,045	12,781,699	12,818,212	12,535,127	12,468,906
Total length (>= 5000 bp)	12,657,092	12,689,988	12,670,119	12,580,045	12,781,699	12,818,212	12,535,127	12,468,906
Total length (>= 10000 bp)	12,657,092	12,689,988	12,670,119	12,580,045	12,781,699	12,818,212	12,535,127	12,468,906
Total length (>= 25000 bp)	12,626,893	12,659,773	12,670,119	12,580,045	12,781,699	12,818,212	12,535,127	12,468,906
Total length (>= 50000 bp)	12,626,893	12,659,773	12,670,119	12,580,045	12,625,385	12,661,699	12,535,127	12,468,906
# contigs	20	20	14	**14**	17	17	15	**15**
Largest contig	1,447,304	1,451,120	1,466,898	1,456,735	1,899,230	1,905,306	1,392,668	1,385,062
Total length	12,669,334	12,702,247	12,670,119	12,580,045	12,781,699	12,818,212	12,535,127	12,468,906
GC (%)	38.84	38.86	39.17	39.18	38.77	38.81	38.82	38.91
N50	1,132,572	1,134,977	1,083,747	**1,075,062**	1,074,435	1,078,207	1,077,343	**1,072,409**
N75	951,427	954,117	728,380	722,648	739,581	738,962	714,689	710,797
L50	5	5	5	5	5	5	5	5
L75	8	8	9	9	8	8	9	9
# N's per 100 kbp	0	0	0	0	0	0	0	0
Illumina Fold coverage				8				26
Nanopore Fold coverage			51				42	

	CG_UHB_09			
	Nanopore reads: 49,465			
	Canu	Canu corrected	Minimap	Minimap corrected
# contigs (>= 0 bp)	27	27	28	28
# contigs (>= 1000 bp)	27	27	28	28
# contigs (>= 5000 bp)	23	23	28	28
# contigs (>= 10000 bp)	20	20	28	28
# contigs (>= 25000 bp)	17	17	28	28
# contigs (>= 50000 bp)	16	16	27	27
Total length (>= 0 bp)	12,571,638	12,633,394	12,561,390	12,476,723
Total length (>= 1000 bp)	12,571,638	12,633,394	12,561,390	12,476,723
Total length (>= 5000 bp)	12,557,198	12,618,945	12,561,390	12,476,723
Total length (>= 10000 bp)	12,533,520	12,595,214	12,561,390	12,476,723
Total length (>= 25000 bp)	12,483,152	12,544,799	12,561,390	12,476,723
Total length (>= 50000 bp)	12,453,066	12,514,674	12,533,639	12,448,970
# contigs	27	**27**	28	28
Largest contig	1,839,122	1,847,993	1,298,925	1,289,505
Total length	12,571,638	12,633,394	12,561,390	12,476,723
GC (%)	38.69	38.78	38.71	38.8
N50	993,250	**998,655**	922,535	917,618
N75	695,109	698,270	445,368	441,763
L50	5	5	6	6
L75	9	9	12	12
# N's per 100 kbp	0	0	0	0
Illumina Fold coverage				36
Nanopore Fold coverage			42	
Nanopore reads	49,465			

Compared genomes. Full list and metadata of compared *Candida glabrata* genomes (encompassing new genomes sequenced with Illumina over this study and publicly available genomes published previously).

Sample Name	Run	NCBI BioProject	NCBI BioSample	Experiment	Country	Host disease	Instrument	Isolation source	Sample accession	SRA Study	cluster	reference
1A	1A	NA	NA	NA	Norway	Candidemia	NextSeq 500	blood	NA	NA	P12	
1B	1B	NA	NA	NA	Norway	Candidemia	NextSeq 501	blood	NA	NA	P12	
2A	2A	NA	NA	NA	Norway	Candidemia	NextSeq 502	blood	NA	NA	P12	
2B	2B	NA	NA	NA	Norway	Candidemia	NextSeq 503	blood	NA	NA	P12	
3A	3A	NA	NA	NA	Norway	Candidemia	NextSeq 504	blood	NA	NA	P10	
3B	3B	NA	NA	NA	Norway	Candidemia	NextSeq 505	blood	NA	NA	P10	
CCTCC-M202019	CCTCC-M202019	NA	NA	NA	NA	Candidemia	NextSeq 506	industry	NA	NA	P11	
DSY562	DSY562	NA	NA	NA	Switzerland	Candidemia	NextSeq 507	Mouth	NA	NA	P06	
DSY565	DSY565	NA	NA	NA	Switzerland	Candidemia	NextSeq 508	Mouth	NA	NA	P06	
SAMEA103980802	ERR1938042	PRJEB20459	SAMEA103980802	ERX1998575	Denmark	NA	ILLUMINA	NA	ERS1669970	ERP022614	P04	NA
SAMEA103980803	ERR1938043	PRJEB20459	SAMEA103980803	ERX1998576	Denmark	NA	ILLUMINA	NA	ERS1669971	ERP022614	Qmosaic	NA
SAMEA103980804	ERR1938044	PRJEB20459	SAMEA103980804	ERX1998577	Denmark	NA	ILLUMINA	NA	ERS1669972	ERP022614	P11	NA
SAMEA103980805	ERR1938045	PRJEB20459	SAMEA103980805	ERX1998578	Denmark	NA	ILLUMINA	NA	ERS1669973	ERP022614	Qmosaic	NA
SAMEA103980806	ERR1938046	PRJEB20459	SAMEA103980806	ERX1998579	Denmark	NA	ILLUMINA	NA	ERS1669974	ERP022614	Qmosaic	NA

SAMEA103980807	ERR1938047	PRJEB20459	SAMEA103980807	ERX1998580	Denmark	NA	ILLUMINA	NA	ERS1669975	ERP022614	P11	NA
SAMEA103980808	ERR1938048	PRJEB20459	SAMEA103980808	ERX1998581	Denmark	NA	ILLUMINA	NA	ERS1669976	ERP022614	P13	NA
SAMEA103980809	ERR1938049	PRJEB20459	SAMEA103980809	ERX1998582	Denmark	NA	ILLUMINA	NA	ERS1669977	ERP022614	Qmosaic	NA
SAMEA103980810	ERR1938050	PRJEB20459	SAMEA103980810	ERX1998583	Denmark	NA	ILLUMINA	NA	ERS1669978	ERP022614	P04	NA
SAMEA103980811	ERR1938051	PRJEB20459	SAMEA103980811	ERX1998584	Denmark	NA	ILLUMINA	NA	ERS1669979	ERP022614	P11	NA
SAMEA103980812	ERR1938052	PRJEB20459	SAMEA103980812	ERX1998585	Denmark	NA	ILLUMINA	NA	ERS1669980	ERP022614	P13	NA
SAMEA103980813	ERR1938053	PRJEB20459	SAMEA103980813	ERX1998586	Denmark	NA	ILLUMINA	NA	ERS1669981	ERP022614	Qmosaic	NA
SAMEA103980814	ERR1938054	PRJEB20459	SAMEA103980814	ERX1998587	Denmark	NA	ILLUMINA	blood	ERS1669982	ERP022614	Qmosaic	NA
SAMEA103980815	ERR1938055	PRJEB20459	SAMEA103980815	ERX1998588	Denmark	NA	ILLUMINA	NA	ERS1669983	ERP022614	Qmosaic	NA
SAMEA103980816	ERR1938056	PRJEB20459	SAMEA103980816	ERX1998589	Denmark	NA	ILLUMINA	NA	ERS1669984	ERP022614	P11	NA
SAMEA103980817	ERR1938057	PRJEB20459	SAMEA103980817	ERX1998590	Denmark	NA	ILLUMINA	NA	ERS1669985	ERP022614	P11	NA
SAMEA103980818	ERR1938058	PRJEB20459	SAMEA103980818	ERX1998591	Denmark	NA	ILLUMINA	NA	ERS1669986	ERP022614	Qmosaic	NA
SAMEA10398	ERR1938059	PRJEB20459	SAMEA103980819	ERX1998592	Denmark	NA	ILLUMINA	NA	ERS1669987	ERP022614	P11	NA

0819												
SAMEA103980820	ERR1938060	PRJEB20459	SAMEA103980820	ERX1998593	Denmark	NA	ILLUMINA	NA	ERS1669988	ERP022614	P11	NA
SAMEA103980821	ERR1938061	PRJEB20459	SAMEA103980821	ERX1998594	Denmark	NA	ILLUMINA	NA	ERS1669989	ERP022614	P08	NA
SAMEA103980822	ERR1938062	PRJEB20459	SAMEA103980822	ERX1998595	Denmark	NA	ILLUMINA	NA	ERS1669990	ERP022614	Qmosaic	NA
SAMEA103980823	ERR1938063	PRJEB20459	SAMEA103980823	ERX1998596	Denmark	NA	ILLUMINA	NA	ERS1669991	ERP022614	P11	NA
SAMEA103980824	ERR1938064	PRJEB20459	SAMEA103980824	ERX1998597	Denmark	NA	ILLUMINA	NA	ERS1669992	ERP022614	P11	NA
SAMEA103980825	ERR1938065	PRJEB20459	SAMEA103980825	ERX1998598	Denmark	NA	ILLUMINA	NA	ERS1669993	ERP022614	P11	NA
SAMEA103980826	ERR1938066	PRJEB20459	SAMEA103980826	ERX1998599	Denmark	NA	ILLUMINA	NA	ERS1669994	ERP022614	P11	NA
SAMEA103980827	ERR1938067	PRJEB20459	SAMEA103980827	ERX1998600	Denmark	NA	ILLUMINA	NA	ERS1669995	ERP022614	P11	NA
SAMEA103980828	ERR1938068	PRJEB20459	SAMEA103980828	ERX1998601	Denmark	NA	ILLUMINA	NA	ERS1669996	ERP022614	P11	NA
SAMEA103980829	ERR1938069	PRJEB20459	SAMEA103980829	ERX1998602	Denmark	NA	ILLUMINA	NA	ERS1669997	ERP022614	P11	NA
SAMEA103980830	ERR1938070	PRJEB20459	SAMEA103980830	ERX1998603	Denmark	NA	ILLUMINA	NA	ERS1669998	ERP022614	P11	NA
SAMEA103980831	ERR1938071	PRJEB20459	SAMEA103980831	ERX1998604	Denmark	NA	ILLUMINA	NA	ERS1669999	ERP022614	P11	NA

SAMEA103980832	ERR1938072	PRJEB20459	SAMEA103980832	ERX1998605	Denmark	NA	ILLUMINA	NA	ERS1670000	ERP022614	Qmosaic	NA
SAMEA103980833	ERR1938073	PRJEB20459	SAMEA103980833	ERX1998606	Denmark	NA	ILLUMINA	NA	ERS1670001	ERP022614	P11	NA
SAMEA103980834	ERR1938074	PRJEB20459	SAMEA103980834	ERX1998607	Denmark	NA	ILLUMINA	NA	ERS1670002	ERP022614	Qmosaic	NA
SAMEA103980835	ERR1938075	PRJEB20459	SAMEA103980835	ERX1998608	Denmark	NA	ILLUMINA	NA	ERS1670003	ERP022614	P11	NA
SAMEA103980836	ERR1938076	PRJEB20459	SAMEA103980836	ERX1998609	Denmark	NA	ILLUMINA	NA	ERS1670004	ERP022614	P01	NA
SAMEA103980837	ERR1938077	PRJEB20459	SAMEA103980837	ERX1998610	Denmark	NA	ILLUMINA	feces	ERS1670005	ERP022614	Qmosaic	NA
SAMEA103980838	ERR1938078	PRJEB20459	SAMEA103980838	ERX1998611	Denmark	NA	ILLUMINA	NA	ERS1670006	ERP022614	P11	NA
SAMEA103980839	ERR1938079	PRJEB20459	SAMEA103980839	ERX1998612	Denmark	NA	ILLUMINA	NA	ERS1670007	ERP022614	P08	NA
SAMEA103980840	ERR1938080	PRJEB20459	SAMEA103980840	ERX1998613	Denmark	NA	ILLUMINA	NA	ERS1670008	ERP022614	P11	NA
SAMEA103980841	ERR1938081	PRJEB20459	SAMEA103980841	ERX1998614	Denmark	NA	ILLUMINA	NA	ERS1670009	ERP022614	Qmosaic	NA
SAMEA103980842	ERR1938082	PRJEB20459	SAMEA103980842	ERX1998615	Denmark	NA	ILLUMINA	blood	ERS1670010	ERP022614	P14	NA
SAMEA103980843	ERR1938083	PRJEB20459	SAMEA103980843	ERX1998616	Denmark	NA	ILLUMINA	blood	ERS1670011	ERP022614	P11	NA
SAMEA10398	ERR1938084	PRJEB20459	SAMEA103980844	ERX1998617	Denmark	NA	ILLUMINA	blood	ERS1670012	ERP022614	P06	NA

0844												
SAMEA103980845	ERR1938085	PRJEB20459	SAMEA103980845	ERX1998618	UK	NA	ILLUMINA	NA	ERS1670013	ERP022614	P11	NA
SAMEA103980846	ERR1938086	PRJEB20459	SAMEA103980846	ERX1998619	Iowa	NA	ILLUMINA	blood	ERS1670014	ERP022614	Qmosaic	NA
SAMEA103980847	ERR1938087	PRJEB20459	SAMEA103980847	ERX1998620	Denmark	NA	ILLUMINA	blood	ERS1670015	ERP022614	Qmosaic	NA
SAMEA103980848	ERR1938088	PRJEB20459	SAMEA103980848	ERX1998621	Denmark	NA	ILLUMINA	blood	ERS1670016	ERP022614	P11	NA
SAMEA103980849	ERR1938089	PRJEB20459	SAMEA103980849	ERX1998622	NA	NA	ILLUMINA	NA	ERS1670017	ERP022614	P11	NA
FFUL887	FFUL887	NA	NA	NA	Portugal	Candidemia	NextSeq 509	urine	NA	NA	P04	
ATCC_90030	SRR3146184	PRJNA310957	SAMN04457271	SRX1561768	Australia	Candidemia	NextSeq 500	blood	SRS1275878	SRP069313	P09	Carreté, L., Ksiezopolska, E., Pegueroles, C., Gómez-Molero, E., Saus, E., Iraola-Guzmán, S., Loska, D., Bader, O., Fairhead, C., Gabaldón, T. (2018) Patterns of genomic variation in the opportunistic pathogen Candida glabrata suggest the existence of mating and a secondary association with humans. Curr Biol. 28(1): 15–27.e7. doi:10.1016/j.cub.2017.11.027
CMRL-3	SRR3151533	PRJNA310957	SAMN04457319	SRX1564588	Australia	Candidemia	NextSeq 500	blood	SRS1278892	SRP069313	P09	
CMRL-4	SRR3151534	PRJNA310957	SAMN04457320	SRX1564602	Australia	Candidemia	NextSeq 500	blood	SRS1278901	SRP069313	Qmosaic	
CMRL-7	SRR3151582	PRJNA310957	SAMN04457396	SRX1564605	Australia	Candidemia	NextSeq 500	blood	SRS1278904	SRP069313	Qmosaic	
CMRL-8	SRR3151591	PRJNA310957	SAMN04457444	SRX1564606	Australia	Candidemia	NextSeq 500	Fluid	SRS1278905	SRP069313	P09	
CMRL-1	SRR3154166	PRJNA310957	SAMN04457317	SRX1561935	Australia	Candidemia	NextSeq 500	blood	SRS1276569	SRP069313	P07	
CMRL-2	SRR3154234	PRJNA310957	SAMN04457318	SRX1564549	Australia	Candidemia	NextSeq 500	blood	SRS1278848	SRP069313	Qmosaic	
CMRL-9	SRR3154235	PRJNA310957	SAMN04457445	SRX1564608	Australia	Candidemia	NextSeq 500	blood	SRS1278907	SRP069313	P08	
CMRL-10	SRR3154236	PRJNA310957	SAMN04457446	SRX1564609	Australia	Candidemia	NextSeq 500	blood	SRS1278908	SRP069313	P13	
CMRL-	SRR3154	PRJNA	SAMN044574	SRX1564610	Australia	Candidemia	NextSeq 500	blood	SRS1278909	SRP069313	Qmosai	

11	237	310957	48								c	
CMRL-12	SRR3154238	PRJNA310957	SAMN04457449	SRX1564611	Australia	Candidemia	NextSeq 500	blood	SRS1278910	SRP069313	P02	
CAS08-0012	SRR3925732	PRJNA329124	SAMN05407972	SRX1951972	NA	NA	Illumina HiSeq 2500	NA	SRS1566068	SRP078607	P09	NA
CAS08_0016	SRR3925733	PRJNA329124	SAMN05392109	SRX1951973	NA	NA	Illumina HiSeq 2500	NA	SRS1566069	SRP078607	P07	NA
CAS08_0270	SRR3925734	PRJNA329124	SAMN05392118	SRX1951974	NA	NA	Illumina HiSeq 2500	NA	SRS1566070	SRP078607	P11	NA
CAS08_0297	SRR3925735	PRJNA329124	SAMN05392119	SRX1951975	NA	NA	Illumina HiSeq 2500	NA	SRS1566072	SRP078607	P11	NA
CAS08_0425	SRR3925736	PRJNA329124	SAMN05392120	SRX1951976	NA	NA	Illumina HiSeq 2500	NA	SRS1566071	SRP078607	P09	NA
CAS08_0430	SRR3925737	PRJNA329124	SAMN05392121	SRX1951977	NA	NA	Illumina HiSeq 2500	NA	SRS1566073	SRP078607	P09	NA
CAS08_0439	SRR3925738	PRJNA329124	SAMN05392122	SRX1951978	NA	NA	Illumina HiSeq 2500	NA	SRS1566074	SRP078607	P09	NA
CAS09_0820	SRR3925739	PRJNA329124	SAMN05392123	SRX1951979	NA	NA	Illumina HiSeq 2500	NA	SRS1566075	SRP078607	P11	NA
CAS09_0869	SRR3925740	PRJNA329124	SAMN05392124	SRX1951980	NA	NA	Illumina HiSeq 2500	NA	SRS1566076	SRP078607	Qmosaic	NA
CAS09_0876	SRR3925741	PRJNA329124	SAMN05392125	SRX1951981	NA	NA	Illumina HiSeq 2500	NA	SRS1566077	SRP078607	Qmosaic	NA
CAS09_1083	SRR3925742	PRJNA329124	SAMN05392126	SRX1951982	NA	NA	Illumina HiSeq 2500	NA	SRS1566078	SRP078607	P09	NA
CAS09_1084	SRR3925743	PRJNA329124	SAMN05392127	SRX1951983	NA	NA	Illumina HiSeq 2500	NA	SRS1566079	SRP078607	P09	NA
CAS08_0030	SRR3925744	PRJNA329124	SAMN05392110	SRX1951984	NA	NA	Illumina HiSeq 2500	NA	SRS1566080	SRP078607	P07	NA
CAS09_1104	SRR3925745	PRJNA329124	SAMN05392128	SRX1951985	NA	NA	Illumina HiSeq 2500	NA	SRS1566081	SRP078607	P11	NA
CAS09_1648	SRR3925746	PRJNA329124	SAMN05392129	SRX1951986	NA	NA	Illumina HiSeq 2500	NA	SRS1566082	SRP078607	P07	NA
CAS09_1784	SRR3925747	PRJNA329124	SAMN05392130	SRX1951987	NA	NA	Illumina HiSeq 2500	NA	SRS1566083	SRP078607	P14	NA
CAS09	SRR3925	PRJNA	SAMN053921	SRX1951988	NA	NA	Illumina HiSeq	NA	SRS1566084	SRP078607	P12	NA

_1786	748	329124	31				2500					
CAS11_2978	SRR3925749	PRJNA329124	SAMN05392132	SRX1951989	NA	NA	Illumina HiSeq 2500	NA	SRS1566085	SRP078607	P07	NA
CAS11_3112	SRR3925750	PRJNA329124	SAMN05392133	SRX1951990	NA	NA	Illumina HiSeq 2500	NA	SRS1566086	SRP078607	P11	NA
CAS11_3129	SRR3925751	PRJNA329124	SAMN05392134	SRX1951991	NA	NA	Illumina HiSeq 2500	NA	SRS1566087	SRP078607	P11	NA
CAS11_3144	SRR3925752	PRJNA329124	SAMN05392135	SRX1951992	NA	NA	Illumina HiSeq 2500	NA	SRS1566088	SRP078607	P11	NA
CAS11_3145	SRR3925753	PRJNA329124	SAMN05392136	SRX1951993	NA	NA	Illumina HiSeq 2500	NA	SRS1566089	SRP078607	P04	NA
CAS11_3302	SRR3925754	PRJNA329124	SAMN05392137	SRX1951994	NA	NA	Illumina HiSeq 2500	NA	SRS1566090	SRP078607	P03	NA
CAS08_0037	SRR3925755	PRJNA329124	SAMN05392111	SRX1951995	NA	NA	Illumina HiSeq 2500	NA	SRS1566091	SRP078607	P03	NA
CAS11_3389	SRR3925756	PRJNA329124	SAMN05392138	SRX1951996	NA	NA	Illumina HiSeq 2500	NA	SRS1566092	SRP078607	P11	NA
CAS11_3527	SRR3925757	PRJNA329124	SAMN05392139	SRX1951997	NA	NA	Illumina HiSeq 2500	NA	SRS1566093	SRP078607	P04	NA
CAS11_3570	SRR3925758	PRJNA329124	SAMN05392140	SRX1951998	NA	NA	Illumina HiSeq 2500	NA	SRS1566094	SRP078607	Qmosaic	NA
CAS11_3606	SRR3925759	PRJNA329124	SAMN05392141	SRX1951999	NA	NA	Illumina HiSeq 2500	NA	SRS1566095	SRP078607	P04	NA
CAS11_3609	SRR3925760	PRJNA329124	SAMN05392142	SRX1952000	NA	NA	Illumina HiSeq 2500	NA	SRS1566096	SRP078607	P06	NA
CAS11_4416	SRR3925761	PRJNA329124	SAMN05392143	SRX1952001	NA	NA	Illumina HiSeq 2500	NA	SRS1566097	SRP078607	P11	NA
CAS12_4417	SRR3925762	PRJNA329124	SAMN05392144	SRX1952002	NA	NA	Illumina HiSeq 2500	NA	SRS1566098	SRP078607	P04	NA
CAS12_4418	SRR3925763	PRJNA329124	SAMN05392145	SRX1952003	NA	NA	Illumina HiSeq 2500	NA	SRS1566099	SRP078607	P06	NA
CAS13_4503	SRR3925764	PRJNA329124	SAMN05392146	SRX1952004	NA	NA	Illumina HiSeq 2500	NA	SRS1566100	SRP078607	P04	NA
CAS13_5051	SRR3925765	PRJNA329124	SAMN05392147	SRX1952005	NA	NA	Illumina HiSeq 2500	NA	SRS1566101	SRP078607	P04	NA
CAS08	SRR3925	PRJNA	SAMN053921	SRX1952006	NA	NA	Illumina HiSeq	NA	SRS1566103	SRP078607	P07	NA

_0092	766	329124	12				2500					
CAS13_5084	SRR3925767	PRJNA329124	SAMN05392148	SRX1952007	NA	NA	Illumina HiSeq 2500	NA	SRS1566104	SRP078607	P04	NA
CAS14_5795	SRR3925768	PRJNA329124	SAMN05392149	SRX1952008	NA	NA	Illumina HiSeq 2500	NA	SRS1566102	SRP078607	P04	NA
CAS08_0094	SRR3925769	PRJNA329124	SAMN05392113	SRX1952009	NA	NA	Illumina HiSeq 2500	NA	SRS1566105	SRP078607	Qmosaic	NA
CAS08_0199	SRR3925770	PRJNA329124	SAMN05392114	SRX1952010	NA	NA	Illumina HiSeq 2500	NA	SRS1566106	SRP078607	P07	NA
CAS08_0205	SRR3925771	PRJNA329124	SAMN05392115	SRX1952011	NA	NA	Illumina HiSeq 2500	NA	SRS1566107	SRP078607	P09	NA
CAS08_0209	SRR3925772	PRJNA329124	SAMN05392116	SRX1952012	NA	NA	Illumina HiSeq 2500	NA	SRS1566108	SRP078607	P09	NA
CAS08_0253	SRR3925773	PRJNA329124	SAMN05392117	SRX1952013	NA	NA	Illumina HiSeq 2500	NA	SRS1566109	SRP078607	P05	NA
CMRL-5	SRR4198629	PRJNA310957	SAMN04457321	SRX1564603	Australia	Candidemia	NextSeq 500	Pelvis	SRS1278902	SRP069313	P13	Carreté, L., Ksiezopolska, E., Pegueroles, C., Gómez-Molero, E., Saus, E., Iraola-Guzmán, S., Loska, D., Bader, O., Fairhead, C., Gabaldón, T. (2018) Patterns of genomic variation in the opportunistic pathogen Candida glabrata suggest the existence of mating and a secondary association with humans. Curr Biol. 28(1): 15–27.e7. doi:10.1016/j.cub.2017.11.027
CMRL-6	SRR4237306	PRJNA310957	SAMN04457333	SRX1564604	Australia	Candidemia	NextSeq 500	urine	SRS1278903	SRP069313	Qmosaic	
M17_CANGA	SRR5239753	PRJNA361477	SAMN06115393	SRX2546657	USA	missing	Illumina Genome Analyzer IIx	blood	SRS1965355	NA	Qmosaic	
EF0313Blo1_CANGA	SRR5239754	PRJNA361477	SAMN06233985	SRX2546658	France	missing	Illumina Genome Analyzer IIx	blood	SRS1965356	NA	P04	
EF1521Blo1_CANGA	SRR5239755	PRJNA361477	SAMN06233962	SRX2546659	France	missing	Illumina Genome Analyzer IIx	blood	SRS1965357	NA	P08	
E1114_CANGA	SRR5239756	PRJNA361477	SAMN06234143	SRX2546660	Belgium	missing	Illumina Genome Analyzer IIx	Mouth	SRS1965358	NA	P08	
BG2_CANGA	SRR5239757	PRJNA361477	SAMN06233961	SRX2546661	USA	missing	Illumina Genome Analyzer IIx	blood	SRS1965359	NA	P08	
F2229_CANGA	SRR5239758	PRJNA361477	SAMN06234144	SRX2546662	France	missing	Illumina HiSeq 2000	blood	SRS1965360	NA	P06	

EG01004Sto_CANGA	SRR5239759	PRJNA361477	SAMN06234138	SRX2546663	Germany	missing	Illumina HiSeq 2000	stool	SRS1965361	NA	P04	
CST110_CANGA	SRR5239760	PRJNA361477	SAMN06234135	SRX2546664	USA	missing	Illumina HiSeq 2000	blood	SRS1965362	NA	P04	
M6_CANGA	SRR5239761	PRJNA361477	SAMN06233986	SRX2546665	USA	missing	Illumina HiSeq 2000	blood	SRS1965363	NA	P04	
P35_3_CANGA	SRR5239762	PRJNA361477	SAMN06233960	SRX2546666	Taiwan	missing	Illumina HiSeq 2000	Mouth	SRS1965366	NA	P07	
P35_2_CANGA	SRR5239763	PRJNA361477	SAMN06233959	SRX2546667	Taiwan	missing	Illumina HiSeq 2000	Mouth	SRS1965364	NA	P07	
EF1117Blo1_CANGA	SRR5239764	PRJNA361477	SAMN06233958	SRX2546668	France	missing	Illumina HiSeq 2000	blood	SRS1965365	NA	Qmosaic	
EF1535Blo1_CANGA	SRR5239765	PRJNA361477	SAMN06233957	SRX2546669	France	missing	Illumina HiSeq 2000	blood	SRS1965369	NA	P09	
EF1620Sto_CANGA	SRR5239766	PRJNA361477	SAMN06233956	SRX2546670	France	missing	Illumina HiSeq 2000	stool	SRS1965367	NA	P14	
EF0616Blo1_CANGA	SRR5239767	PRJNA361477	SAMN06233955	SRX2546671	France	missing	Illumina HiSeq 2000	blood	SRS1965368	NA	P14	
EF1237Blo1_CANGA	SRR5239768	PRJNA361477	SAMN06233954	SRX2546672	France	missing	Illumina HiSeq 2000	blood	SRS1965370	NA	P14	
EI1815Blo1_CANGA	SRR5239769	PRJNA361477	SAMN06233945	SRX2546673	Italy	missing	Illumina HiSeq 2000	blood	SRS1965371	NA	P14	
I1718_CANGA	SRR5239770	PRJNA361477	SAMN06233931	SRX2546674	Italy	missing	Illumina HiSeq 2000	blood	SRS1965372	NA	P06	
CST78_CANGA	SRR5239771	PRJNA361477	SAMN06233926	SRX2546675	USA	missing	Illumina HiSeq 2000	blood	SRS1965373	NA	P06	

F1822_CANGA	SRR5239772	PRJNA361477	SAMN06233918	SRX2546676	USA	missing	Illumina HiSeq 2000	blood	SRS1965374	NA	P11	
F1019_CANGA	SRR5239773	PRJNA361477	SAMN06135318	SRX2546677	France	missing	Illumina HiSeq 2000	blood	SRS1965375	NA	P06	
M12_CANGA	SRR5239774	PRJNA361477	SAMN06115384	SRX2546678	USA	missing	Illumina HiSeq 2000	blood	SRS1965376	NA	P06	
CST35_CANGA	SRR5239775	PRJNA361477	SAMN06115381	SRX2546679	USA	missing	Illumina HiSeq 2000	blood	SRS1965377	NA	P02	
EB0911Sto_CANGA	SRR5239776	PRJNA361477	SAMN06115077	SRX2546680	Belgium	missing	Illumina HiSeq 2000	stool	SRS1965378	NA	P02	
CST109_CANGA	SRR5239777	PRJNA361477	SAMN06115074	SRX2546681	USA	missing	Illumina HiSeq 2000	blood	SRS1965379	NA	P03	
CST34_CANGA	SRR5239778	PRJNA361477	SAMN06115073	SRX2546682	USA	missing	Illumina HiSeq 2000	blood	SRS1965381	NA	P03	
M7_CANGA	SRR5239779	PRJNA361477	SAMN06115072	SRX2546683	USA	missing	Illumina HiSeq 2000	blood	SRS1965380	NA	P03	
CST80_CANGA	SRR5239780	PRJNA361477	SAMN06115071	SRX2546684	USA	missing	Illumina HiSeq 2000	blood	SRS1965382	NA	P03	
BO101S_CANGA	SRR5239781	PRJNA361477	SAMN06115070	SRX2546685	Belgium	missing	Illumina HiSeq 2000	stool	SRS1965383	NA	P03	
EB101M_CANGA	SRR5239782	PRJNA361477	SAMN06114929	SRX2546686	Belgium	missing	Illumina HiSeq 2000	Mouth	SRS1965384	NA	P03	
B1012S_CANGA	SRR5239783	PRJNA361477	SAMN06114790	SRX2546687	Belgium	missing	Illumina HiSeq 2000	stool	SRS1965385	NA	P03	
B1012M_CANGA	SRR5239784	PRJNA361477	SAMN06114788	SRX2546688	Belgium	missing	Illumina HiSeq 2000	Mouth	SRS1965386	NA	P03	

T3-24Y	SRR5839059	PRJNA393577	SAMN07337702	SRX3015149	USA		Illumina HiSeq 2500	lab	SRS2364824	SRP112804	P11	Guo, X., Zhang, R., Li, Y., Wang, Z., Ishchuk, O.P., Ahmad, K.M., Wee, J., Piskur, J., Shapiro, J.A., Gu, Z. (2020) Understand the genomic diversity and evolution of fungal pathogen Candida glabrata by genome-wide analysis of genetic variations. Methods. 176:82-90. doi: 10.1016/j.ymeth.2019.05.002
T2-27G	SRR5839060	PRJNA393577	SAMN07337701	SRX3015148	USA		Illumina HiSeq 2500	lab	SRS2364823	SRP112804	P11	
T3-12	SRR5839061	PRJNA393577	SAMN07337694	SRX3015147	USA		Illumina HiSeq 2500	lab	SRS2364822	SRP112804	P11	
T3-5	SRR5839062	PRJNA393577	SAMN07337693	SRX3015146	USA		Illumina HiSeq 2500	lab	SRS2364821	SRP112804	P11	
T3-24	SRR5839063	PRJNA393577	SAMN07337696	SRX3015145	USA		Illumina HiSeq 2500	lab	SRS2364820	SRP112804	P11	
T3-13	SRR5839064	PRJNA393577	SAMN07337695	SRX3015144	USA		Illumina HiSeq 2500	lab	SRS2364819	SRP112804	P11	
T2-5G	SRR5839065	PRJNA393577	SAMN07337698	SRX3015143	USA		Illumina HiSeq 2500	lab	SRS2364818	SRP112804	P11	
T1-27G	SRR5839066	PRJNA393577	SAMN07337697	SRX3015142	USA		Illumina HiSeq 2500	lab	SRS2364817	SRP112804	P11	
T2-17G	SRR5839067	PRJNA393577	SAMN07337700	SRX3015141	USA		Illumina HiSeq 2500	lab	SRS2364816	SRP112804	P11	
T2-10G	SRR5839068	PRJNA393577	SAMN07337699	SRX3015140	USA		Illumina HiSeq 2500	lab	SRS2364815	SRP112804	P11	
H2-22Y	SRR5839069	PRJNA393577	SAMN07337714	SRX3015139	USA		Illumina HiSeq 2500	lab	SRS2364814	SRP112804	P11	
H2-18Y	SRR5839070	PRJNA393577	SAMN07337713	SRX3015138	USA		Illumina HiSeq 2500	lab	SRS2364813	SRP112804	P11	
H2-8Y	SRR5839071	PRJNA393577	SAMN07337711	SRX3015137	USA		Illumina HiSeq 2500	lab	SRS2364812	SRP112804	P11	
H2-11G	SRR5839072	PRJNA393577	SAMN07337712	SRX3015136	USA		Illumina HiSeq 2500	lab	SRS2364811	SRP112804	P11	
H3-8	SRR5839073	PRJNA393577	SAMN07337707	SRX3015135	USA		Illumina HiSeq 2500	lab	SRS2364810	SRP112804	P11	
H3-11	SRR5839074	PRJNA393577	SAMN07337708	SRX3015134	USA		Illumina HiSeq 2500	lab	SRS2364809	SRP112804	P11	
H3-18	SRR5839075	PRJNA393577	SAMN07337709	SRX3015133	USA		Illumina HiSeq 2500	lab	SRS2364808	SRP112804	P11	
H3-22	SRR5839076	PRJNA393577	SAMN07337710	SRX3015132	USA		Illumina HiSeq 2500	lab	SRS2364807	SRP112804	P11	

H2-8	SRR5839077	PRJNA393577	SAMN07337703	SRX3015131	USA		Illumina HiSeq 2500	lab	SRS2364806	SRP112804	P11	
H2-11	SRR5839078	PRJNA393577	SAMN07337704	SRX3015130	USA		Illumina HiSeq 2500	lab	SRS2364805	SRP112804	P11	
H2-18	SRR5839079	PRJNA393577	SAMN07337705	SRX3015129	USA		Illumina HiSeq 2500	lab	SRS2364804	SRP112804	P11	
H2-22	SRR5839080	PRJNA393577	SAMN07337706	SRX3015128	USA		Illumina HiSeq 2500	lab	SRS2364803	SRP112804	P11	
T2-27	SRR5839081	PRJNA393577	SAMN07337692	SRX3015127	USA		Illumina HiSeq 2500	lab	SRS2364802	SRP112804	P11	
T2-17	SRR5839082	PRJNA393577	SAMN07337691	SRX3015126	USA		Illumina HiSeq 2500	lab	SRS2364801	SRP112804	P11	
T1-10	SRR5839083	PRJNA393577	SAMN07337686	SRX3015125	USA		Illumina HiSeq 2500	lab	SRS2364800	SRP112804	P11	
T1-5	SRR5839084	PRJNA393577	SAMN07337685	SRX3015124	USA		Illumina HiSeq 2500	lab	SRS2364799	SRP112804	P11	
MHCg-G	SRR5839085	PRJNA393577	SAMN07337684	SRX3015123	USA		Illumina HiSeq 2500	lab	SRS2364798	SRP112804	P11	
MHCg-Y	SRR5839086	PRJNA393577	SAMN07337683	SRX3015122	USA		Illumina HiSeq 2500	lab	SRS2364797	SRP112804	P11	
T2-10	SRR5839087	PRJNA393577	SAMN07337690	SRX3015121	USA		Illumina HiSeq 2500	lab	SRS2364796	SRP112804	P11	
T2-5	SRR5839088	PRJNA393577	SAMN07337689	SRX3015120	USA		Illumina HiSeq 2500	lab	SRS2364795	SRP112804	P11	
T1-27	SRR5839089	PRJNA393577	SAMN07337688	SRX3015119	USA		Illumina HiSeq 2500	lab	SRS2364794	SRP112804	P11	
T1-17	SRR5839090	PRJNA393577	SAMN07337687	SRX3015118	USA		Illumina HiSeq 2500	lab	SRS2364793	SRP112804	P11	
WM_18.54	SRR8068012	PRJNA480138	SAMN10245837	SRX4896388	Australia	Candidaemia	NextSeq 500	blood	SRS3942957	SRP165946	Qmosaic	Biswas, C., Marcelino, V.R., Van Hal, S., Halliday, C., Martinez, E., Wang, Q., Kidd, S., Kennedy, K., Marriott, D., Morrissey, C.O., Arthur, I., Weeks, K.,
WM_18.53	SRR8068013	PRJNA480138	SAMN10245836	SRX4896387	Australia	Candidaemia	NextSeq 500	blood	SRS3942956	SRP165946	Qmosaic	
WM_18.45	SRR8068014	PRJNA480138	SAMN10245829	SRX4896386	Australia	Candidaemia	NextSeq 500	blood	SRS3942955	SRP165946	Qmosaic	
WM_18.44	SRR8068015	PRJNA480138	SAMN10245828	SRX4896385	Australia	Candidaemia	NextSeq 500	blood	SRS3942954	SRP165946	P08	

WM_18.48	SRR8068016	PRJNA480138	SAMN10245831	SRX4896384	Australia	Candidaemia	NextSeq 500	blood	SRS3942953	SRP165946	P05	Slavin, M.A., Sorrell, T.C., Sintchenko, V., Meyer, W., C.-A. Chen, S. (2018) Whole genome sequencing of Australian Candida glabrata isolates reveals genetic diversity and Novel Sequence Types. Front Microbiol. 9: 2946. doi: 10.3389/fmicb.2018.02946
WM_18.47	SRR8068017	PRJNA480138	SAMN10245830	SRX4896383	Australia	Candidaemia	NextSeq 500	blood	SRS3942952	SRP165946	Qmosaic	
WM_18.50	SRR8068018	PRJNA480138	SAMN10245833	SRX4896382	Australia	Candidaemia	NextSeq 500	blood	SRS3942951	SRP165946	P11	
WM_18.49	SRR8068019	PRJNA480138	SAMN10245832	SRX4896381	Australia	Candidaemia	NextSeq 500	blood	SRS3942950	SRP165946	P13	
WM_18.52	SRR8068020	PRJNA480138	SAMN10245835	SRX4896380	Australia	Candidaemia	NextSeq 500	Fluid	SRS3942949	SRP165946	P11	
WM_18.51	SRR8068021	PRJNA480138	SAMN10245834	SRX4896379	Australia	Candidaemia	NextSeq 500	blood	SRS3942948	SRP165946	Qmosaic	
WM_18.24	SRR8068022	PRJNA480138	SAMN10245811	SRX4896378	Australia	Candidaemia	NextSeq 500	blood	SRS3942947	SRP165946	P11	
WM_05.155	SRR8068023	PRJNA480138	SAMN10245810	SRX4896377	Australia	Candidaemia	NextSeq 500	blood	SRS3942946	SRP165946	Qmosaic	
WM_05.113	SRR8068024	PRJNA480138	SAMN10245809	SRX4896376	Australia	Candidaemia	NextSeq 500	blood	SRS3942945	SRP165946	P13	
WM_05.111	SRR8068025	PRJNA480138	SAMN10245808	SRX4896375	Australia	Candidaemia	NextSeq 500	blood	SRS3942943	SRP165946	P02	
WM_18.30	SRR8068026	PRJNA480138	SAMN10245815	SRX4896374	Australia	Candidaemia	NextSeq 500	blood	SRS3942942	SRP165946	P08	
WM_18.29	SRR8068027	PRJNA480138	SAMN10245814	SRX4896373	Australia	Candidaemia	NextSeq 500	Fluid	SRS3942941	SRP165946	Qmosaic	
WM_18.27	SRR8068028	PRJNA480138	SAMN10245813	SRX4896372	Australia	Candidaemia	NextSeq 500	blood	SRS3942940	SRP165946	Qmosaic	
WM_18.26	SRR8068029	PRJNA480138	SAMN10245812	SRX4896371	Australia	Candidaemia	NextSeq 500	blood	SRS3942939	SRP165946	P11	
WM_18.33	SRR8068030	PRJNA480138	SAMN10245817	SRX4896370	Australia	Candidaemia	NextSeq 500	blood	SRS3942938	SRP165946	P02	
WM_18.31	SRR8068031	PRJNA480138	SAMN10245816	SRX4896369	Australia	Candidaemia	NextSeq 500	blood	SRS3942937	SRP165946	Qmosaic	
WM_18.67	SRR8068032	PRJNA480138	SAMN10245848	SRX4896368	Australia	Candidaemia	NextSeq 500	Tissue	SRS3942936	SRP165946	P13	
WM_04.387	SRR8068033	PRJNA480138	SAMN10245807	SRX4896367	Australia	Candidaemia	NextSeq 500	blood	SRS3942935	SRP165946	Qmosaic	

WM_04.242	SRR8068034	PRJNA480138	SAMN10245806	SRX4896366	Australia	Candidaemia	NextSeq 500	blood	SRS3942934	SRP165946	P02	
WM_03.419	SRR8068035	PRJNA480138	SAMN10245799	SRX4896365	Australia	Candidaemia	NextSeq 500	blood	SRS3942933	SRP165946	P05	
WM_03.308	SRR8068036	PRJNA480138	SAMN10245423	SRX4896364	Australia	Candidaemia	NextSeq 500	blood	SRS3942932	SRP165946	P02	
WM_03.450	SRR8068037	PRJNA480138	SAMN10245801	SRX4896363	Australia	Candidaemia	NextSeq 500	blood	SRS3942931	SRP165946	P05	
WM_03.449	SRR8068038	PRJNA480138	SAMN10245800	SRX4896362	Australia	Candidaemia	NextSeq 500	blood	SRS3942930	SRP165946	P13	
WM_03.707	SRR8068039	PRJNA480138	SAMN10245803	SRX4896361	Australia	Candidaemia	NextSeq 500	blood	SRS3942929	SRP165946	P05	
WM_03.698	SRR8068040	PRJNA480138	SAMN10245802	SRX4896360	Australia	Candidaemia	NextSeq 500	blood	SRS3942928	SRP165946	P02	
WM_04.194	SRR8068041	PRJNA480138	SAMN10245805	SRX4896359	Australia	Candidaemia	NextSeq 500	blood	SRS3942927	SRP165946	P08	
WM_04.113	SRR8068042	PRJNA480138	SAMN10245804	SRX4896358	Australia	Candidaemia	NextSeq 500	blood	SRS3942926	SRP165946	P01	
WM_18.42	SRR8068043	PRJNA480138	SAMN10245826	SRX4896357	Australia	Candidaemia	NextSeq 500	blood	SRS3942925	SRP165946	P01	
WM_18.43	SRR8068044	PRJNA480138	SAMN10245827	SRX4896356	Australia	Candidaemia	NextSeq 500	blood	SRS3942924	SRP165946	P11	
WM_18.36	SRR8068045	PRJNA480138	SAMN10245820	SRX4896355	Australia	Candidaemia	NextSeq 500	blood	SRS3942923	SRP165946	Qmosaic	
WM_18.37	SRR8068046	PRJNA480138	SAMN10245821	SRX4896354	Australia	Candidaemia	NextSeq 500	blood	SRS3942922	SRP165946	P14	
WM_18.34	SRR8068047	PRJNA480138	SAMN10245818	SRX4896353	Australia	Candidaemia	NextSeq 500	blood	SRS3942921	SRP165946	P08	
WM_18.35	SRR8068048	PRJNA480138	SAMN10245819	SRX4896352	Australia	Candidaemia	NextSeq 500	blood	SRS3942920	SRP165946	P08	
WM_18.40	SRR8068049	PRJNA480138	SAMN10245824	SRX4896351	Australia	Candidaemia	NextSeq 500	blood	SRS3942919	SRP165946	Qmosaic	
WM_18.41	SRR8068050	PRJNA480138	SAMN10245825	SRX4896350	Australia	Candidaemia	NextSeq 500	blood	SRS3942918	SRP165946	P01	
WM_18.38	SRR8068051	PRJNA480138	SAMN10245822	SRX4896349	Australia	Candidaemia	NextSeq 500	blood	SRS3942917	SRP165946	Qmosaic	

WM_18 .39	SRR8068 052	PRJNA 480138	SAMN102458 23	SRX4896348	Australia	Candidaemia	NextSeq 500	blood	SRS3942916	SRP165946	P05	
WM_18 .65	SRR8068 053	PRJNA 480138	SAMN102458 46	SRX4896347	Australia	Candidaemia	NextSeq 500	blood	SRS3942915	SRP165946	Qmosai c	
WM_18 .66	SRR8068 054	PRJNA 480138	SAMN102458 47	SRX4896346	Australia	Candidaemia	NextSeq 500	blood	SRS3942914	SRP165946	Qmosai c	
WM_18 .55	SRR8068 055	PRJNA 480138	SAMN102458 38	SRX4896345	Australia	Candidaemia	NextSeq 500	blood	SRS3942913	SRP165946	P08	
WM_18 .56	SRR8068 056	PRJNA 480138	SAMN102458 39	SRX4896344	Australia	Candidaemia	NextSeq 500	blood	SRS3942912	SRP165946	P11	
WM_18 .57	SRR8068 057	PRJNA 480138	SAMN102458 40	SRX4896343	Australia	Candidaemia	NextSeq 500	blood	SRS3942911	SRP165946	Qmosai c	
WM_18 .59	SRR8068 058	PRJNA 480138	SAMN102458 41	SRX4896342	Australia	Candidaemia	NextSeq 500	blood	SRS3942910	SRP165946	P08	
WM_18 .60	SRR8068 059	PRJNA 480138	SAMN102458 42	SRX4896341	Australia	Candidaemia	NextSeq 500	blood	SRS3942909	SRP165946	P08	
WM_18 .62	SRR8068 060	PRJNA 480138	SAMN102458 43	SRX4896340	Australia	Candidaemia	NextSeq 500	blood	SRS3942908	SRP165946	P11	
WM_18 .63	SRR8068 061	PRJNA 480138	SAMN102458 44	SRX4896339	Australia	Candidaemia	NextSeq 500	blood	SRS3942907	SRP165946	P11	
WM_18 .64	SRR8068 062	PRJNA 480138	SAMN102458 45	SRX4896338	Australia	Candidaemia	NextSeq 500	blood	SRS3942944	SRP165946	P11	
SAT01 BAL	SRR8241 569	PRJNA 506893	SAMN104754 01	SRX5059823	France	Myloblastic leukaemia	Illumina HiSeq 2000	Bronchiol o-alveolar lavage	SRS4075807	SRP170662	P02	Carreté, L., Ksiezopolska, E., Gómez-Molero, E., Angoulvant, A., Bader, O., Fairhead, C., Gabaldón, T. (2019) Genome comparisons of Candida glabrata serial clinical isolates reveal patterns of genetic variation in infecting clonal populations. Front Microbiol. 10: 112. doi:
SAT02P C	SRR8241 570	PRJNA 506893	SAMN104754 02	SRX5059822	France	Myloblastic leukaemia	Illumina HiSeq 2000	Fluid	SRS4075806	SRP170662	P02	
SAT03 BC	SRR8241 571	PRJNA 506893	SAMN104754 03	SRX5059821	France	Myloblastic leukaemia	Illumina HiSeq 2000	blood	SRS4075805	SRP170662	P02	
UAB04 7	UAB047	NA	NA	NA	Mariland	Candidemia	NextSeq 510	vaginal swab	NA	NA	P07	
REF	REF	REF	REF	REF	REF	REF	REF	lab	REF	REF	P11	

												10.3389/fmicb.2019.0
CG_UHB_01	SRR14381471	PRJNA589840	SAMN18953774	SRR14381471	UK	unknown	Illumiuna HiSeq 2000	blood	SRR14381471	PRJNA589840	P04	this study
CG_UHB_02	SRR14381470	PRJNA589840	SAMN18953775	SRR14381470	UK	unknown	Illumiuna HiSeq 2000	sputum	SRR14381470	PRJNA589840	Qmosaic	this study
CG_UHB_03	SRR14381458	PRJNA589840	SAMN18953776	SRR14381458	UK	unknown	Illumiuna HiSeq 2000	Fluid	SRR14381458	PRJNA589840	Qmosaic	this study
CG_UHB_04	SRR14381451	PRJNA589840	SAMN18953777	SRR14381451	UK	unknown	Illumiuna HiSeq 2000	Fluid	SRR14381451	PRJNA589840	P12	this study
CG_UHB_05	SRR14381447	PRJNA589840	SAMN18953778	SRR14381447	UK	unknown	Illumiuna HiSeq 2000	sputum	SRR14381447	PRJNA589840	Qmosaic	this study
CG_UHB_06	SRR14381446	PRJNA589840	SAMN18953779	SRR14381446	UK	unknown	Illumiuna HiSeq 2000	Culture for ID	SRR14381446	PRJNA589840	P04	this study
CG_UHB_07	SRR14381445	PRJNA589840	SAMN18953780	SRR14381445	UK	unknown	Illumiuna HiSeq 2000	blood	SRR14381445	PRJNA589840	P06	this study
CG_UHB_08	SRR14381444	PRJNA589840	SAMN18953781	SRR14381444	UK	unknown	Illumiuna HiSeq 2000	Culture for ID	SRR14381444	PRJNA589840	P02	this study
CG_UHB_09	SRR14381443	PRJNA589840	SAMN18953782	SRR14381443	UK	unknown	Illumiuna HiSeq 2000	Fluid	SRR14381443	PRJNA589840	Qmosaic	this study
CG_UHB_10	SRR14381442	PRJNA589840	SAMN18953783	SRR14381442	UK	unknown	Illumiuna HiSeq 2000	blood	SRR14381442	PRJNA589840	Qmosaic	this study
CG_UHB_11	SRR14381469	PRJNA589840	SAMN18953784	SRR14381469	UK	unknown	Illumiuna HiSeq 2000	BAL	SRR14381469	PRJNA589840	P09	this study
CG_UHB_12	SRR14381468	PRJNA589840	SAMN18953785	SRR14381468	UK	unknown	Illumiuna HiSeq 2000	Fluid	SRR14381468	PRJNA589840	P10	this study
CG_UHB_13	SRR14381466	PRJNA589840	SAMN18953786	SRR14381466	UK	unknown	Illumiuna HiSeq 2000	wound swab	SRR14381466	PRJNA589840	P12	this study
CG_UHB_14	SRR14381465	PRJNA589840	SAMN18953787	SRR14381465	UK	unknown	Illumiuna HiSeq 2000	Culture for ID	SRR14381465	PRJNA589840	P12	this study
CG_UHB_15	SRR14381464	PRJNA589840	SAMN18953788	SRR14381464	UK	unknown	Illumiuna HiSeq 2000	blood	SRR14381464	PRJNA589840	P14	this study
CG_UHB_16	SRR14381467	PRJNA589840	SAMN18953789	SRR14381467	UK	unknown	Illumiuna HiSeq 2000	blood	SRR14381467	PRJNA589840	P02	this study
CG_UHB_17	SRR14381463	PRJNA589840	SAMN18953790	SRR14381463	UK	unknown	Illumiuna HiSeq 2000	blood	SRR14381463	PRJNA589840	P02	this study

CG_UHB_18	SRR14381462	PRJNA589840	SAMN18953791	SRR14381462	UK	unknown	Illumiuna HiSeq 2000	Culture for ID	SRR14381462	PRJNA589840	P06	this study
CG_UHB_19	SRR14381461	PRJNA589840	SAMN18953792	SRR14381461	UK	unknown	Illumiuna HiSeq 2000	Culture for ID	SRR14381461	PRJNA589840	P04	this study
CG_UHB_20	SRR14381460	PRJNA589840	SAMN18953793	SRR14381460	UK	unknown	Illumiuna HiSeq 2000	Culture for ID	SRR14381460	PRJNA589840	P04	this study
CG_UHB_21	SRR14381457	PRJNA589840	SAMN18953794	SRR14381457	UK	unknown	Illumiuna HiSeq 2000	blood	SRR14381457	PRJNA589840	Qmosaic	this study
CG_UHB_22	SRR14381456	PRJNA589840	SAMN18953795	SRR14381456	UK	unknown	Illumiuna HiSeq 2000	blood	SRR14381456	PRJNA589840	P12	this study
CG_UHB_23	SRR14381459	PRJNA589840	SAMN18953796	SRR14381459	UK	unknown	Illumiuna HiSeq 2000	venous blood culture	SRR14381459	PRJNA589840	P02	this study
CG_UHB_24	SRR14381454	PRJNA589840	SAMN18953797	SRR14381454	UK	unknown	Illumiuna HiSeq 2000	blood	SRR14381454	PRJNA589840	P04	this study
CG_UHB_25	SRR14381455	PRJNA589840	SAMN18953798	SRR14381455	UK	unknown	Illumiuna HiSeq 2000	blood	SRR14381455	PRJNA589840	P04	this study
CG_UHB_26	SRR14381453	PRJNA589840	SAMN18953799	SRR14381453	UK	unknown	Illumiuna HiSeq 2000	blood	SRR14381453	PRJNA589840	P04	this study
CG_UHB_27	SRR14381452	PRJNA589840	SAMN18953800	SRR14381452	UK	unknown	Illumiuna HiSeq 2000	Culture for ID	SRR14381452	PRJNA589840	Qmosaic	this study
CG_UHB_28	SRR14381450	PRJNA589840	SAMN18953801	SRR14381450	UK	unknown	Illumiuna HiSeq 2000	blood	SRR14381450	PRJNA589840	P04	this study
CG_UHB_29	SRR14381449	PRJNA589840	SAMN18953802	SRR14381449	UK	unknown	Illumiuna HiSeq 2000	blood	SRR14381449	PRJNA589840	P10	this study
CG_UHB_30	SRR14381448	PRJNA589840	SAMN18953803	SRR14381448	UK	unknown	Illumiuna HiSeq 2000	Fluid	SRR14381448	PRJNA589840	P06	this study

Back to the index

Supplementary information

Supplementary Table 2

Information on the presence/absence of chromosomal rearrangements in the *C. glabrata* strains isolated in this or previous studies.

Table S2
Information on the presence/absence of chromosomal rearrangements in the C. glabrata strains isolated over this or previous studies

Run: NCBI Run ID

BioProject: NCBI BioProject ID

BioSample: NCBI BioSample ID

Country: Country of isolation

isolation_source: source of isolation

Selective_Stress: Stress imposed to the strain (for experimental evolution studies)

reference: study reporting the corresponding data; 1= Biswas, C., Marcelino, V.R., Van Hal, S., Halliday, C., Martinez, E., Wang, Q., Kidd, S., Kennedy, K., Marriott, D., Morrissey, C.O., Arthur, I., Weeks, K., Slavin, M.A., Sorrell, T.C., Sintchenko, V., Meyer, W., C.-A. Chen, S. (2018) Whole genome sequencing of Australian Candida glabrata isolates reveals genetic diversity and Novel Sequence Types. Front Microbiol. 9: 2946. doi: 10.3389/fmicb.2018.02946; 2= Carreté, L., Ksiezopolska, E., Gómez-Molero, E., Angoulvant, A., Bader, O., Fairhead, C., Gabaldón, T. (2019) Genome comparisons of Candida glabrata serial clinical isolates reveal patterns of genetic variation in infecting clonal populations. Front Microbiol. 10: 112. doi: 10.3389/fmicb.2019.00112; 3= Guo, X., Zhang, R., Li, Y., Wang, Z., Ishchuk, O.P., Ahmad, K.M., Wee, J., Piskur, J., Shapiro, J.A., Gu, Z. (2020) Understand the genomic diversity and evolution of fungal pathogen Candida glabrata by genome-wide analysis of genetic variations. Methods. 176:82-90. doi: 10.1016/j.ymeth.2019.05.002

inversion_chrL: presence/absence of the inversion in chromosome L. yes= present; no= absent; NA= data do not allow detection of the inversion

translocation_ChrI-ChrL: presence/absence of the translocation of the left arm of chromosome I to chromosome L. yes= present; no= absent; NA= data do not allow detection of the translocation

rearrangements: presence/absence of chromosomal rearrangments. Both= both the inversion and the translocation are present; none= nor the inversion nor the translocation are present; translocation= only the translocation is present; inversion= only the inversion is present; NA= data do not allow detection of the inversion nor of the translocation

Run	BioProject	BioSample	Country	Isolation source	Selective_Stress	reference	Inversion chrL	Translocation ChrI-ChrL	rearrangements
1A	NA	NA	Norway	blood		NA	no	no	none
1B	NA	NA	Norway	blood		NA	no	no	none
2A	NA	NA	Norway	blood		NA	NA	NA	NA
2B	NA	NA	Norway	blood		NA	no	no	none
3A	NA	NA	Norway	blood		NA	NA	NA	NA
3B	NA	NA	Norway	blood		NA	NA	NA	NA
CCTCC.M202019	NA	NA	NA	industry		NA	no	no	none
DSY562	NA	NA	CH	Mouth		NA	yes	yes	both
DSY565	NA	NA	CH	Mouth		NA	yes	yes	both
FFUL887	NA	NA	Portugal	urine		NA	yes	yes	both
UAB047	NA	NA	USA	vaginal swab		NA	yes	yes	both
ERR1938042	PRJEB20459	SAMEA103980802	Denmark	NA		NA	yes	yes	both
ERR1938043	PRJEB20459	SAMEA103980803	Denmark	NA		NA	yes	yes	both
ERR1938044	PRJEB20459	SAMEA103980804	Denmark	blood		NA	no	no	none
ERR1938045	PRJEB20459	SAMEA103980805	Denmark	blood		NA	yes	yes	both
ERR1938046	PRJEB20459	SAMEA103980806	Denmark	blood		NA	yes	yes	both
ERR1938047	PRJEB20459	SAMEA103980807	Denmark	NA		NA	yes	yes	both
ERR1938048	PRJEB20459	SAMEA103980808	Denmark	blood		NA	no	yes	transposition
ERR1938049	PRJEB20459	SAMEA103980809	Denmark	blood		NA	yes	yes	both
ERR1938050	PRJEB20459	SAMEA103980810	Denmark	blood		NA	yes	yes	both
ERR1938051	PRJEB20459	SAMEA103980811	Denmark	blood		NA	yes	yes	both
ERR1938052	PRJEB20459	SAMEA103980812	Denmark	blood		NA	no	no	none
ERR1938053	PRJEB20459	SAMEA103980813	Denmark	blood		NA	yes	yes	both
ERR1938054	PRJEB20459	SAMEA103980814	Denmark	blood		NA	yes	yes	both
ERR1938055	PRJEB20459	SAMEA103980815	Denmark	Lymph node		NA	yes	yes	both
ERR1938056	PRJEB20459	SAMEA103980816	Denmark	Palate		NA	no	no	none
ERR1938057	PRJEB20459	SAMEA103980817	Denmark	blood		NA	no	no	none
ERR1938058	PRJEB20459	SAMEA103980818	Denmark	Urine		NA	no	no	none
ERR1938059	PRJEB20459	SAMEA103980819	Denmark	standard strain		NA	no	no	none
ERR1938060	PRJEB20459	SAMEA103980820	Denmark	feces		NA	yes	yes	both
ERR1938061	PRJEB20459	SAMEA103980821	Denmark	throat		NA	yes	yes	both
ERR1938062	PRJEB20459	SAMEA103980822	Denmark	NA		NA	no	no	none
ERR1938063	PRJEB20459	SAMEA103980823	Denmark	Gland		NA	no	no	none

ERR1938064	PRJEB20459	SAMEA103980824	Denmark	NA		NA	no	no	none
ERR1938065	PRJEB20459	SAMEA103980825	Denmark	blood		NA	yes	yes	both
ERR1938066	PRJEB20459	SAMEA103980826	Denmark	Catheter spit		NA	no	no	none
ERR1938067	PRJEB20459	SAMEA103980827	Denmark	blood		NA	no	no	none
ERR1938068	PRJEB20459	SAMEA103980828	Denmark	feces		NA	yes	yes	both
ERR1938069	PRJEB20459	SAMEA103980829	Denmark	blood		NA	yes	yes	both
ERR1938070	PRJEB20459	SAMEA103980830	Denmark	blood		NA	yes	yes	both
ERR1938071	PRJEB20459	SAMEA103980831	Denmark	blood		NA	yes	yes	both
ERR1938072	PRJEB20459	SAMEA103980832	Denmark	feces		NA	yes	yes	both
ERR1938073	PRJEB20459	SAMEA103980833	Denmark	blood		NA	no	no	none
ERR1938074	PRJEB20459	SAMEA103980834	Denmark	blood		NA	no	no	none
ERR1938075	PRJEB20459	SAMEA103980835	Denmark	blood		NA	no	no	none
ERR1938076	PRJEB20459	SAMEA103980836	Denmark	blood		NA	no	yes	transposition
ERR1938077	PRJEB20459	SAMEA103980837	Denmark	feces		NA	yes	yes	both
ERR1938078	PRJEB20459	SAMEA103980838	Denmark	blood		NA	yes	yes	both
ERR1938079	PRJEB20459	SAMEA103980839	Denmark	feces		NA	yes	yes	both
ERR1938080	PRJEB20459	SAMEA103980840	Denmark	feces		NA	no	no	none
ERR1938081	PRJEB20459	SAMEA103980841	Denmark	blood		NA	no	no	none
ERR1938082	PRJEB20459	SAMEA103980842	Denmark	blood		NA	no	no	none
ERR1938083	PRJEB20459	SAMEA103980843	Denmark	blood		NA	no	no	none
ERR1938084	PRJEB20459	SAMEA103980844	Denmark	blood		NA	yes	yes	both
ERR1938085	PRJEB20459	SAMEA103980845	UK	NA		NA	yes	yes	both
ERR1938086	PRJEB20459	SAMEA103980846	USA	blood		NA	no	no	none
ERR1938087	PRJEB20459	SAMEA103980847	Denmark	blood		NA	yes	yes	both
ERR1938088	PRJEB20459	SAMEA103980848	Denmark	blood		NA	yes	yes	both
ERR1938089	PRJEB20459	SAMEA103980849	NA	NA		NA	yes	yes	both
SRR3146184	PRJNA310957	SAMN04457271	Australia	blood		2	no	no	none
SRR3151533	PRJNA310957	SAMN04457319	Australia	blood		2	no	no	none
SRR3151534	PRJNA310957	SAMN04457320	Australia	blood		2	no	no	none
SRR3151582	PRJNA310957	SAMN04457396	Australia	blood		2	yes	yes	both
SRR3151591	PRJNA310957	SAMN04457444	Australia	Fluid		2	no	no	none
SRR3154166	PRJNA310957	SAMN04457317	Australia	blood		2	yes	yes	both
SRR3154234	PRJNA310957	SAMN04457318	Australia	blood		2	yes	yes	both
SRR3154235	PRJNA310957	SAMN04457445	Australia	blood		2	yes	yes	both
SRR3154236	PRJNA310957	SAMN04457446	Australia	blood		2	no	no	none
SRR3154237	PRJNA310957	SAMN04457448	Australia	blood		2	yes	yes	both
SRR3154238	PRJNA310957	SAMN04457449	Australia	blood		2	yes	yes	both

SRR4198629	PRJNA310957	SAMN04457321	Australia	Pelvis		2	no	no	none
SRR4237306	PRJNA310957	SAMN04457333	Australia	urine		2	no	no	none
SRR3925732	PRJNA329124	SAMN05407972	NA	NA		NA	no	no	none
SRR3925733	PRJNA329124	SAMN05392109	NA	NA		NA	yes	yes	both
SRR3925734	PRJNA329124	SAMN05392118	NA	NA		NA	yes	yes	both
SRR3925735	PRJNA329124	SAMN05392119	NA	NA		NA	no	no	none
SRR3925736	PRJNA329124	SAMN05392120	NA	NA		NA	yes	no	inversion
SRR3925737	PRJNA329124	SAMN05392121	NA	NA		NA	no	no	none
SRR3925738	PRJNA329124	SAMN05392122	NA	NA		NA	no	no	none
SRR3925739	PRJNA329124	SAMN05392123	NA	NA		NA	no	no	none
SRR3925740	PRJNA329124	SAMN05392124	NA	NA		NA	yes	yes	both
SRR3925741	PRJNA329124	SAMN05392125	NA	NA		NA	no	no	none
SRR3925742	PRJNA329124	SAMN05392126	NA	NA		NA	no	yes	transposition
SRR3925743	PRJNA329124	SAMN05392127	NA	NA		NA	no	no	none
SRR3925744	PRJNA329124	SAMN05392110	NA	NA		NA	yes	yes	both
SRR3925745	PRJNA329124	SAMN05392128	NA	NA		NA	no	no	none
SRR3925746	PRJNA329124	SAMN05392129	NA	NA		NA	yes	yes	both
SRR3925747	PRJNA329124	SAMN05392130	NA	NA		NA	no	no	none
SRR3925748	PRJNA329124	SAMN05392131	NA	NA		NA	yes	yes	both
SRR3925749	PRJNA329124	SAMN05392132	NA	NA		NA	yes	yes	both
SRR3925750	PRJNA329124	SAMN05392133	NA	NA		NA	yes	yes	both
SRR3925751	PRJNA329124	SAMN05392134	NA	NA		NA	yes	yes	both
SRR3925752	PRJNA329124	SAMN05392135	NA	NA		NA	yes	yes	both
SRR3925753	PRJNA329124	SAMN05392136	NA	NA		NA	yes	yes	both
SRR3925754	PRJNA329124	SAMN05392137	NA	NA		NA	yes	yes	both
SRR3925755	PRJNA329124	SAMN05392111	NA	NA		NA	yes	yes	both
SRR3925756	PRJNA329124	SAMN05392138	NA	NA		NA	no	no	none
SRR3925757	PRJNA329124	SAMN05392139	NA	NA		NA	yes	yes	both
SRR3925758	PRJNA329124	SAMN05392140	NA	NA		NA	yes	yes	both
SRR3925759	PRJNA329124	SAMN05392141	NA	NA		NA	yes	yes	both
SRR3925760	PRJNA329124	SAMN05392142	NA	NA		NA	yes	yes	both
SRR3925761	PRJNA329124	SAMN05392143	NA	NA		NA	yes	yes	both
SRR3925762	PRJNA329124	SAMN05392144	NA	NA		NA	yes	yes	both
SRR3925763	PRJNA329124	SAMN05392145	NA	NA		NA	yes	yes	both
SRR3925764	PRJNA329124	SAMN05392146	NA	NA		NA	yes	yes	both
SRR3925765	PRJNA329124	SAMN05392147	NA	NA		NA	yes	yes	both
SRR3925766	PRJNA329124	SAMN05392112	NA	NA		NA	yes	yes	both

SRR3925767	PRJNA329124	SAMN05392148	NA	NA		NA	yes	yes	both
SRR3925768	PRJNA329124	SAMN05392149	NA	NA		NA	yes	yes	both
SRR3925769	PRJNA329124	SAMN05392113	NA	NA		NA	no	no	none
SRR3925770	PRJNA329124	SAMN05392114	NA	NA		NA	yes	yes	both
SRR3925771	PRJNA329124	SAMN05392115	NA	NA		NA	no	no	none
SRR3925772	PRJNA329124	SAMN05392116	NA	NA		NA	no	no	none
SRR3925773	PRJNA329124	SAMN05392117	NA	NA		NA	yes	no	inversion
SRR5239753	PRJNA361477	SAMN06115393	USA	blood		2	yes	yes	both
SRR5239754	PRJNA361477	SAMN06233985	France	blood		2	yes	yes	both
SRR5239755	PRJNA361477	SAMN06233962	France	blood		2	yes	yes	both
SRR5239756	PRJNA361477	SAMN06234143	Belgium	Mouth		2	yes	yes	both
SRR5239757	PRJNA361477	SAMN06233961	USA	blood		2	yes	yes	both
SRR5239758	PRJNA361477	SAMN06234144	France	blood		2	yes	yes	both
SRR5239759	PRJNA361477	SAMN06234138	Germany	stool		2	yes	yes	both
SRR5239760	PRJNA361477	SAMN06234135	USA	blood		2	yes	yes	both
SRR5239761	PRJNA361477	SAMN06233986	USA	blood		2	yes	yes	both
SRR5239762	PRJNA361477	SAMN06233960	Taiwan	Mouth		2	yes	yes	both
SRR5239763	PRJNA361477	SAMN06233959	Taiwan	Mouth		2	yes	yes	both
SRR5239764	PRJNA361477	SAMN06233958	France	blood		2	no	no	none
SRR5239765	PRJNA361477	SAMN06233957	France	blood		2	no	no	none
SRR5239766	PRJNA361477	SAMN06233956	France	stool		2	no	no	none
SRR5239767	PRJNA361477	SAMN06233955	France	blood		2	no	no	none
SRR5239768	PRJNA361477	SAMN06233954	France	blood		2	no	no	none
SRR5239769	PRJNA361477	SAMN06233945	Italy	blood		2	no	no	none
SRR5239770	PRJNA361477	SAMN06233931	Italy	blood		2	yes	yes	both
SRR5239771	PRJNA361477	SAMN06233926	USA	blood		2	yes	yes	both
SRR5239772	PRJNA361477	SAMN06233918	USA	blood		2	yes	yes	both
SRR5239773	PRJNA361477	SAMN06135318	France	blood		2	yes	yes	both
SRR5239774	PRJNA361477	SAMN06115384	USA	blood		2	yes	yes	both
SRR5239775	PRJNA361477	SAMN06115381	USA	blood		2	yes	yes	both
SRR5239776	PRJNA361477	SAMN06115077	Belgium	stool		2	yes	yes	both
SRR5239777	PRJNA361477	SAMN06115074	USA	blood		2	yes	yes	both
SRR5239778	PRJNA361477	SAMN06115073	USA	blood		2	yes	yes	both
SRR5239779	PRJNA361477	SAMN06115072	USA	blood		2	yes	yes	both
SRR5239780	PRJNA361477	SAMN06115071	USA	blood		2	yes	yes	both
SRR5239781	PRJNA361477	SAMN06115070	Belgium	stool		2	yes	yes	both
SRR5239782	PRJNA361477	SAMN06114929	Belgium	Mouth		2	yes	yes	both

SRR5239783	PRJNA361477	SAMN06114790	Belgium	stool		2	yes	yes	both
SRR5239784	PRJNA361477	SAMN06114788	Belgium	Mouth		2	yes	yes	both
SRR5839059	PRJNA393577	SAMN07337702	USA	lab	Periodic challenge at 50°C for 30 minutes	3	no	no	none
SRR5839060	PRJNA393577	SAMN07337701	USA	lab	Periodic challenge at 50°C for 30 minutes	3	no	no	none
SRR5839061	PRJNA393577	SAMN07337694	USA	lab	Periodic challenge at 48°C for 30 minutes	3	no	no	none
SRR5839062	PRJNA393577	SAMN07337693	USA	lab	Periodic challenge at 47°C for 30 minutes	3	no	no	none
SRR5839063	PRJNA393577	SAMN07337696	USA	lab	Periodic challenge at 50°C for 30 minutes	3	no	no	none
SRR5839064	PRJNA393577	SAMN07337695	USA	lab	Periodic challenge at 49°C for 30 minutes	3	no	no	none
SRR5839065	PRJNA393577	SAMN07337698	USA	lab	Periodic challenge at 47°C for 30 minutes	3	no	no	none
SRR5839066	PRJNA393577	SAMN07337697	USA	lab	Periodic challenge at 50°C for 30 minutes	3	no	no	none
SRR5839067	PRJNA393577	SAMN07337700	USA	lab	Periodic challenge at 49°C for 30 minutes	3	no	no	none
SRR5839068	PRJNA393577	SAMN07337699	USA	lab	Periodic challenge at 48°C for 30 minutes	3	no	no	none
SRR5839069	PRJNA393577	SAMN07337714	USA	lab	Periodic challenge with 350 mM of hydrogen peroxide for 1 hour	3	no	no	none
SRR5839070	PRJNA393577	SAMN07337713	USA	lab	Periodic challenge with 300 mM of hydrogen peroxide for 1 hour	3	no	no	none
SRR5839071	PRJNA393577	SAMN07337711	USA	lab	Periodic challenge with 80 mM of hydrogen peroxide for 1 hour	3	no	no	none
SRR5839072	PRJNA393577	SAMN07337712	USA	lab	Periodic challenge with 140 mM of hydrogen peroxide for 1 hour	3	no	no	none
SRR5839073	PRJNA393577	SAMN07337707	USA	lab	Periodic challenge with 80 mM of hydrogen peroxide for 1 hour	3	no	no	none
SRR5839074	PRJNA393577	SAMN07337708	USA	lab	Periodic challenge with 140 mM of hydrogen peroxide for 1 hour	3	no	no	none
SRR5839075	PRJNA393577	SAMN07337709	USA	lab	Periodic challenge with 300 mM of hydrogen peroxide for 1 hour	3	no	no	none
SRR5839076	PRJNA393577	SAMN07337710	USA	lab	Periodic challenge with 350 mM of hydrogen peroxide for 1 hour	3	no	no	none
SRR5839077	PRJNA393577	SAMN07337703	USA	lab	Periodic challenge with 80 mM of hydrogen peroxide for 1 hour	3	yes	no	inversion
SRR5839078	PRJNA393577	SAMN07337704	USA	lab	Periodic challenge with 140 mM of hydrogen peroxide for 1 hour	3	no	no	none
SRR5839079	PRJNA393577	SAMN07337705	USA	lab	Periodic challenge with 300 mM of hydrogen peroxide for 1 hour	3	no	no	none
SRR5839080	PRJNA393577	SAMN07337706	USA	lab	Periodic challenge with 350 mM of hydrogen peroxide for 1 hour	3	no	no	none
SRR5839081	PRJNA393577	SAMN07337692	USA	lab	Periodic challenge at 50°C for 30 minutes	3	no	no	none
SRR5839082	PRJNA393577	SAMN07337691	USA	lab	Periodic challenge at 49°C for 30 minutes	3	no	no	none
SRR5839083	PRJNA393577	SAMN07337686	USA	lab	Periodic challenge at 48°C for 30 minutes	3	no	no	none

SRR5839084	PRJNA393577	SAMN07337685	USA	lab	Periodic challenge at 47°C for 30 minutes	3	yes	no	inversion
SRR5839085	PRJNA393577	SAMN07337684	USA	lab		3	no	no	none
SRR5839086	PRJNA393577	SAMN07337683	USA	lab		3	no	no	none
SRR5839087	PRJNA393577	SAMN07337690	USA	lab	Periodic challenge at 48°C for 30 minutes	3	no	no	none
SRR5839088	PRJNA393577	SAMN07337689	USA	lab	Periodic challenge at 47°C for 30 minutes	3	yes	no	inversion
SRR5839089	PRJNA393577	SAMN07337688	USA	lab	Periodic challenge at 50°C for 30 minutes	3	no	no	none
SRR5839090	PRJNA393577	SAMN07337687	USA	lab	Periodic challenge at 49°C for 30 minutes	3	no	no	none
SRR8068012	PRJNA480138	SAMN10245837	Australia	blood		1	no	no	none
SRR8068013	PRJNA480138	SAMN10245836	Australia	blood		1	no	no	none
SRR8068014	PRJNA480138	SAMN10245829	Australia	blood		1	yes	yes	both
SRR8068015	PRJNA480138	SAMN10245828	Australia	blood		1	yes	yes	both
SRR8068016	PRJNA480138	SAMN10245831	Australia	blood		1	yes	no	inversion
SRR8068017	PRJNA480138	SAMN10245830	Australia	blood		1	yes	yes	both
SRR8068018	PRJNA480138	SAMN10245833	Australia	blood		1	yes	yes	both
SRR8068019	PRJNA480138	SAMN10245832	Australia	blood		1	no	no	none
SRR8068020	PRJNA480138	SAMN10245835	Australia	Fluid		1	yes	yes	both
SRR8068021	PRJNA480138	SAMN10245834	Australia	blood		1	yes	yes	both
SRR8068022	PRJNA480138	SAMN10245811	Australia	blood		1	yes	yes	both
SRR8068023	PRJNA480138	SAMN10245810	Australia	blood		1	yes	yes	both
SRR8068024	PRJNA480138	SAMN10245809	Australia	blood		1	no	no	none
SRR8068025	PRJNA480138	SAMN10245808	Australia	blood		1	yes	yes	both
SRR8068026	PRJNA480138	SAMN10245815	Australia	blood		1	yes	yes	both
SRR8068027	PRJNA480138	SAMN10245814	Australia	Fluid		1	yes	yes	both
SRR8068028	PRJNA480138	SAMN10245813	Australia	blood		1	no	no	none
SRR8068029	PRJNA480138	SAMN10245812	Australia	blood		1	no	no	none
SRR8068030	PRJNA480138	SAMN10245817	Australia	blood		1	yes	yes	both
SRR8068031	PRJNA480138	SAMN10245816	Australia	blood		1	yes	yes	both
SRR8068032	PRJNA480138	SAMN10245848	Australia	Tissue		1	no	no	none
SRR8068033	PRJNA480138	SAMN10245807	Australia	blood		1	yes	yes	both
SRR8068034	PRJNA480138	SAMN10245806	Australia	blood		1	yes	yes	both
SRR8068035	PRJNA480138	SAMN10245799	Australia	blood		1	yes	no	inversion
SRR8068036	PRJNA480138	SAMN10245423	Australia	blood		1	yes	yes	both
SRR8068037	PRJNA480138	SAMN10245801	Australia	blood		1	yes	no	inversion
SRR8068038	PRJNA480138	SAMN10245800	Australia	blood		1	no	no	none
SRR8068039	PRJNA480138	SAMN10245803	Australia	blood		1	yes	no	inversion
SRR8068040	PRJNA480138	SAMN10245802	Australia	blood		1	yes	yes	both
SRR8068041	PRJNA480138	SAMN10245805	Australia	blood		1	yes	yes	both

SRR8068042	PRJNA480138	SAMN10245804	Australia	blood		1	no	no	none
SRR8068043	PRJNA480138	SAMN10245826	Australia	blood		1	no	no	none
SRR8068044	PRJNA480138	SAMN10245827	Australia	blood		1	yes	no	inversion
SRR8068045	PRJNA480138	SAMN10245820	Australia	blood		1	no	no	none
SRR8068046	PRJNA480138	SAMN10245821	Australia	blood		1	no	no	none
SRR8068047	PRJNA480138	SAMN10245818	Australia	blood		1	yes	yes	both
SRR8068048	PRJNA480138	SAMN10245819	Australia	blood		1	yes	yes	both
SRR8068049	PRJNA480138	SAMN10245824	Australia	blood		1	yes	yes	both
SRR8068050	PRJNA480138	SAMN10245825	Australia	blood		1	no	no	none
SRR8068051	PRJNA480138	SAMN10245822	Australia	blood		1	no	no	none
SRR8068052	PRJNA480138	SAMN10245823	Australia	blood		1	yes	no	inversion
SRR8068053	PRJNA480138	SAMN10245846	Australia	blood		1	yes	no	inversion
SRR8068054	PRJNA480138	SAMN10245847	Australia	blood		1	yes	yes	both
SRR8068055	PRJNA480138	SAMN10245838	Australia	blood		1	yes	yes	both
SRR8068056	PRJNA480138	SAMN10245839	Australia	blood		1	no	no	none
SRR8068057	PRJNA480138	SAMN10245840	Australia	blood		1	yes	yes	both
SRR8068058	PRJNA480138	SAMN10245841	Australia	blood		1	yes	yes	both
SRR8068059	PRJNA480138	SAMN10245842	Australia	blood		1	yes	yes	both
SRR8068060	PRJNA480138	SAMN10245843	Australia	blood		1	yes	yes	both
SRR8068061	PRJNA480138	SAMN10245844	Australia	blood		1	yes	yes	both
SRR8068062	PRJNA480138	SAMN10245845	Australia	blood		1	yes	yes	both
SRR8241569	PRJNA506893	SAMN10475401	France	Bronchiolo-alveolar lavage		2	yes	yes	both
SRR8241570	PRJNA506893	SAMN10475402	France	Fluid		2	yes	yes	both
SRR8241571	PRJNA506893	SAMN10475403	France	blood		2	yes	yes	both
REF	REF	REF	REF	REF		NA	no	no	none
Scerevisiae	REF	REF	REF	REF		NA	NA	NA	NA
SRR14381464	PRJNA589840	SAMN18953788	UK	blood		this study	no	no	none
SRR14381442	PRJNA589840	SAMN18953783	UK	blood		this study	no	no	none
SRR14381467	PRJNA589840	SAMN18953789	UK	blood		this study	yes	yes	both
SRR14381463	PRJNA589840	SAMN18953790	UK	blood		this study	yes	yes	both
SRR14381468	PRJNA589840	SAMN18953785	UK	Fluid		this study	yes	yes	both
SRR14381462	PRJNA589840	SAMN18953791	UK	Culture for ID		this study	yes	yes	both
SRR14381461	PRJNA589840	SAMN18953792	UK	Culture for ID		this study	yes	yes	both
SRR14381460	PRJNA589840	SAMN18953793	UK	Culture for ID		this study	yes	yes	both
SRR14381457	PRJNA589840	SAMN18953794	UK	blood		this study	yes	yes	both

SRR14381456	PRJNA589840	SAMN18953795	UK	blood		this study	no	no	none
SRR14381445	PRJNA589840	SAMN18953780	UK	blood		this study	yes	yes	both
SRR14381459	PRJNA589840	SAMN18953796	UK	venous blood culture		this study	yes	yes	both
SRR14381471	PRJNA589840	SAMN18953774	UK	blood		this study	yes	yes	both
SRR14381454	PRJNA589840	SAMN18953797	UK	blood		this study	yes	yes	both
SRR14381455	PRJNA589840	SAMN18953798	UK	blood		this study	yes	yes	both
SRR14381453	PRJNA589840	SAMN18953799	UK	blood		this study	yes	yes	both
SRR14381465	PRJNA589840	SAMN18953787	UK	Culture for ID		this study	no	yes	transposition
SRR14381444	PRJNA589840	SAMN18953781	UK	Culture for ID		this study	yes	yes	both
SRR14381446	PRJNA589840	SAMN18953779	UK	Culture for ID		this study	yes	yes	both
SRR14381452	PRJNA589840	SAMN18953800	UK	Culture for ID		this study	no	no	none
SRR14381466	PRJNA589840	SAMN18953786	UK	wound swab		this study	no	no	none
SRR14381450	PRJNA589840	SAMN18953801	UK	blood		this study	yes	yes	both
SRR14381469	PRJNA589840	SAMN18953784	UK	BAL		this study	no	no	none
SRR14381447	PRJNA589840	SAMN18953778	UK	sputum		this study	yes	yes	both
SRR14381458	PRJNA589840	SAMN18953776	UK	Fluid		this study	no	no	none
SRR14381449	PRJNA589840	SAMN18953802	UK	blood		this study	yes	yes	both
SRR14381448	PRJNA589840	SAMN18953803	UK	Fluid		this study	yes	yes	both
SRR14381451	PRJNA589840	SAMN18953777	UK	Fluid		this study	no	yes	transposition
SRR14381470	PRJNA589840	SAMN18953775	UK	sputum		this study	no	no	none
SRR14381443	PRJNA589840	SAMN18953782	UK	Fluid		this study	yes	yes	both

Back to the index

Supplementary information

Supplementary Table 3

Information on genes highly conserved (dN/dS=0) or subjected to positive selection (dN/dS=1) in the 30 *C. glabrata* strains genomes sequenced in this study.

Table S3
Information on genes highly conserved (dN/dS=0) or subjected to positive selection (dN/dS=1) in the 30 *C. glabrata* strains genomes sequenced over this study.

~~Constraint~~.
List of genes with dN/dS>1 in every C. glabrata strain investigated in this study.
~~Highly variable~~ (dN/dS>1). in every
List of genes with dN/dS>1 in every C. glabrata strain investigated in this study.

Candida glabrata ID=gene name;
chromosome= chromosome name;
start_coordinate (REF)= coordinate of the first nucleotide of the gene in the chromosome, in reference to the reference genome;
end_coordinate (REF)=coordinate of the last nucleotide of the gene in the chromosome, in reference to the reference genome;
strand= strand of the coding sequence;
Loci with synonymous mutations (out of the 30 strains)= number of loci in the gene ORF having a variant in at least one of the genomes sequenced over this study;
description= description of the gene, obtained from the *Candida* genome database;
S.cerevisiae ortholog=name, if present of the ortholog gene in *S. cerevisiae*;
description of *S.cerevisiae* ortholog= description of the ortholog gene in *S. cerevisiae*;
Essential (in *S.cerevisiae)*= the ortholog gene is essential ("yes") or not essential ("no") in *S. cerevisiae* ("NA" if there is not an ortholog in *S. cerevisiae*);
status in other strains (only for constraint genes)= if the dN/dS of the gene is =0, then "DN=0 in every sequence available", otherwise "with non-synonymous variants";
strains with non-synonymous variants (isolation source, Country of isolation) (only for constraint genes)= list of strains bearing non-synonimous variants in the gene. Details on the isolation source and Country of isolation are listed in brackets.

Constraint

Candida glabrata ID	chromosome	Start coordinate (REF)	End coordinate (REF)	strand	Loci with synonymous mutations	description	*S.cerevisiae ortholog*	description of *S. cerevisiae* ortholog	Essential	status in other strains	strains with non-synonymous variants (isolation source, Country of isolation)
CAGL0B04345g	B	424411	425589	+	15	Has domain(s) with predicted GTP binding activity	OLA1 (YBR025C)	P-loop ATPase with similarity to human OLA1 and bacterial YchF; identified as specifically interacting with the proteasome; null mutant displays increased translation rate and increased readthrough of premature stop codons; protein abundance increases in response to hydrogen peroxide and to DNA replication stress	no	DN=0 in every sequence available	none
CAGL0H08327g	H	815939	816682	+	3	TPI1; Putative triose-phosphate isomerase; protein abundance decreased in ace2 mutant cells	TPI1 (YDR050C)	Triose phosphate isomerase, abundant glycolytic enzyme; mRNA half-life is regulated by iron availability; transcription is controlled by activators Reb1p, Gcr1p, and Rap1p through binding sites in the 5' non-coding region; inhibition of Tpi1p activity by PEP (phosphoenolpyruvate) stimulates redox metabolism in respiring cells; E104D mutation in human homolog TPI1 causes a rare autosomal disease; human TPI1 can complement yeast null mutant	no	DN=0 in every sequence available	none
CAGL0J03212g	J	310357	311907	+	24	ALD5; Putative mitochondrial aldehyde dehydrogenase (NAD+); protein	ALD5 (YER073W)	Mitochondrial aldehyde dehydrogenase; involved in regulation or biosynthesis of electron transport chain components and acetate	no	DN=0 in every sequence available	none

						abundance increased in ace2 mutant cells		formation; activated by K+; utilizes NADP+ as the preferred coenzyme; constitutively expressed			
CAGL0A 04521g	A	447271	447873	+	3	Ortholog(s) have structural constituent of ribosome activity, role in maturation of SSU-rRNA from tricistronic rRNA transcript (SSU-rRNA, 5.8S rRNA, LSU-rRNA) and cytosolic small ribosomal subunit localization	RPS8A (YBL072C)	Protein component of the small (40S) ribosomal subunit; homologous to mammalian ribosomal protein S8, no bacterial homolog; RPS8A has a paralog, RPS8B, that arose from the whole genome duplication	no (but has a paralog)	DN=0 in every sequence available	none
CAGL0E 04752g	E	456205	456645	-	0	Ortholog(s) have proteasome binding, ubiquitin conjugating enzyme activity, ubiquitin-protein transferase activity	UBC5 (YDR059C)	Ubiquitin-conjugating enzyme; mediates selective degradation of short-lived, abnormal, or excess proteins, including histone H3; central component of the cellular stress response; expression is heat inducible; protein abundance increases in response to DNA replication stress; UBC5 has a paralog, UBC4, that arose from the whole genome duplication	no (but has a paralog)	DN=0 in every sequence available	none
CAGL0D 00440g	D	52247	52798	-	7	Ortholog(s) have role in poly(A)+ mRNA export from nucleus, ribosomal large subunit export from nucleus, ribosomal small subunit export from nucleus and nuclear RNA export factor complex localization	MTR2 (YKL186C)	mRNA transport regulator; essential nuclear protein; Mex67p and Mtr2p form a mRNA export complex which binds to RNA	yes	DN=0 in every sequence available	none
CAGL0F	F	258829	259341	-	9	Ortholog(s) have	PRB7	RNA polymerase II subunit	yes	DN=0 in every	none

02673g						RNA-directed 5'-3' RNA polymerase activity, single-stranded DNA binding, single-stranded RNA binding, translation initiation factor binding activity	(YDR404C)	B16; forms dissociable heterodimer with Rpb4p; Rpb4/7 dissociates from RNAPII as Ser2 CTD phosphorylation increases; Rpb4/7 regulates cellular lifespan via mRNA decay process; involved in recruitment of 3'-end processing factors to transcribing RNA polymerase II complex, export of mRNA to cytoplasm under stress conditions; also involved in translation initiation		sequence available	
CAGL0K 08140g	K	809979	810569	+	6	Ortholog(s) have Rab guanyl-nucleotide exchange factor activity	BET3 (YKR068C)	Core component of transport protein particle (TRAPP) complexes I-III; TRAPP complexes are related multimeric guanine nucleotide-exchange factors for the GTPase Ypt1, regulating ER-Golgi traffic (TRAPPI), intra-Golgi traffic (TRAPPII), endosome-Golgi traffic (TRAPPII and III) and autophagy (TRAPPIII); hydrophilic homodimeric protein that acts in conjunction with SNARE proteins in targeting and fusion of ER to Golgi transport vesicles	yes	DN=0 in every sequence available	none
CAGL0M 12386g	M	1236543	1236968	+	2	Ortholog(s) have role in isoleucine biosynthetic process, mitochondrial translation and mitochondrial matrix, mitochondrion localization	MMF1 (YIL051C)	Mitochondrial protein required for transamination of isoleucine; but not of valine or leucine; may regulate specificity of branched-chain transaminases Bat1p and Bat2p; induction of expression in response to stress is mediated by a Hog1p-regulated antisense RNA and gene looping; interacts genetically with	yes	DN=0 in every sequence available	none

								mitochondrial ribosomal protein genes; MMF1 has a paralog, HMF1, that arose from the whole genome duplication			
CAGL0A 01023g	A	102793	104025	+	24	Ortholog(s) have nicotinamide-nucleotide adenylyltransferase activity and role in NAD biosynthetic process (1)	NMA1 (YLR328W)	Nicotinic acid mononucleotide adenylyltransferase; catalyzes the transfer of the adenylyl moiety of ATP to nicotinamide mononucleotide to form NAD; involved in pathways of NAD biosynthesis, including the de novo, NAD(+) salvage, and nicotinamide riboside salvage pathways; homolog of human NMNAT; NMA1 has a paralog, NMA2, that arose from the whole genome duplication	no (but has a paralog)	with non-synonymous variants	ERR1938062 (NA, Denmark), SRR3151582 (blood, Australia), SRR3151591 (fluid, Australia), SRR4198629 (pelvis, Australia), SRR8068027 (fluid, Australia), SRR8068031 (blood, Australia)
CAGL0C 00649g	C	66983	68509	+	32	Putative H1-ATPase V1 domain 60 KD subunit	VMA2 (YBR127C)	Subunit B of V1 peripheral membrane domain of vacuolar H+-ATPase; electrogenic proton pump found throughout the endomembrane system; contains nucleotide binding sites; also detected in the cytoplasm; protein abundance increases in response to DNA replication stress; human homolog ATP6V1B1, implicated in autosomal-recessive distal renal tubular acidosis (RTA) with sensorineural deafness, complements yeast null mutant	no	with non-synonymous variants	SRR3151591 (fluid, Australia), SRR4198629 (pelvis, Australia), SRR8068014 (blood, Australia), SRR8068028 (blood, Australia)
CAGL0D 00198g	D	27559	28707	+	3	Ortholog(s) have (R,R)-butanediol dehydrogenase activity and role in alcohol metabolic process, butanediol biosynthetic process	BDH1 (YAL060W)	NAD-dependent (R,R)-butanediol dehydrogenase; catalyzes oxidation of (R,R)-2,3-butanediol to (3R)-acetoin, oxidation of meso-butanediol to (3S)-acetoin, and reduction of acetoin; enhances use of 2,3-butanediol as an aerobic carbon	no	with non-synonymous variants	ERR1938081 (NA, Denmark), ERR1938058 (NA, Denmark)

								source			
CAGL0D 01936g	D	202715	203398	-	6	SNF7; Ortholog(s) have role in ATP export, ESCRT III complex assembly, cellular response to anoxia, intralumenal vesicle formation and late endosome to vacuole transport	SNF7 (YLR025W)	One of four subunits of the ESCRT-III complex; involved in the sorting of transmembrane proteins into the multivesicular body (MVB) pathway; recruited from the cytoplasm to endosomal membranes; ESCRT-III stands for endosomal sorting complex required for transport III	no	with non-synonymous variants	ERR1938088 (NA, Denmark), SRR5239762 (mouth, Taiwan), SRR5239763 (mouth, Taiwan), SRR8068025 (blood, Australia), SRR8241569 (Bronchiolo-alveolar lavage, France), SRR8241570 (fluid, France), SRR8241571 (blood, France)
CAGL0E 02937g	E	279285	279626	-	3	Protein of unknown function	CGR1 (YGL029W)	Protein involved in nucleolar integrity and processing of pre-rRNA; has a role in processing rRNA for the 60S ribosome subunit; transcript is induced in response to cytotoxic stress but not genotoxic stress; relocalizes from nucleus to nucleolus upon DNA replication stress	no	with non-synonymous variants	SRR8068027 (fluid, Australia), SRR8068031 (blood, Australia), SRR3151582 (blood, Australia)
CAGL0F 02255g	F	221723	222160	+	0	Ortholog(s) have protein phosphatase activator activity, protein serine/threonine phosphatase inhibitor activity	YPI1 (YFR003C)	Regulatory subunit of the type I protein phosphatase (PP1) Glc7p; Glc7p participates in the regulation of a variety of metabolic processes including mitosis and glycogen metabolism; in vitro evidence suggests Ypi1p is an inhibitor of Glc7p while in vivo evidence suggests it is an activator; overproduction causes decreased cellular content of glycogen; partial depletion causes lithium sensitivity, while overproduction confers lithium-tolerance	yes	with non-synonymous variants	SRR5239753 (blood, USA), SRR5239756 (mouth, Belgium), SRR5239763 (mouth, Taiwan)
CAGL0F 04213g	F	419473	420390	+	10	Ortholog(s) have ATP:ADP antiporter	PET9 (YBL030C)	Major ADP/ATP carrier of the mitochondrial inner membrane;	not in W303,	with non-synonymous	ERR1938069 (NA, Denmark), SRR8068013

						activity		exchanges cytosolic ADP for mitochondrially synthesized ATP; also imports heme and ATP; required for viability in many lab strains that carry a sal1 mutation; PET9 has a paralog, AAC3, that arose from the whole genome duplication; human homolog SLC25A4 implicated in progressive external ophthalmoplegia can complement yeast null mutant	essential in other strain backgrounds	variants	(blood, Australia)
CAGL0F 04895g	F	495596	498292	+	29	Ortholog(s) have glycogen phosphorylase activity, role in glycogen catabolic process and cytoplasm localization	GPH1 (YPR160W)	Glycogen phosphorylase required for the mobilization of glycogen; non-essential; regulated by cyclic AMP-mediated phosphorylation; phosphorylation by Cdc28p may coordinately regulate carbohydrate metabolism and the cell cycle; expression is regulated by stress-response elements and by the HOG MAP kinase pathway	no	with non-synonymous variants	ERR1938061 (NA, Denmark), ERR1938062 (NA, Denmark), ERR1938081 (NA, Denmark), SRR3151591 (fluid, Australia), SRR8068015 (blood, Australia), SRR8068027 (fluid, Australia), SRR8068046 (blood, Australia)
CAGL0F 07029g	F	689331	691130	+	29	MET13; Ortholog(s) have methylenetetrahydrofolate reductase (NAD(P)H) activity and role in methionine biosynthetic process, one-carbon metabolic process	MET13 (YGL125W)	Major isozyme of methylenetetrahydrofolate reductase; catalyzes the reduction of 5,10-methylenetetrahydrofolate to 5-methyltetrahydrofolate in the methionine biosynthesis pathway	no	with non-synonymous variants	SRR3154166 (blood, Australia), SRR3154234 (blood, Australia), SRR3925733 (NA, NA), SRR3925744 (NA, NA), SRR3925746 (NA, NA), SRR3925749 (NA, NA), SRR3925770 (NA, NA), SRR5239762 (mouth, Taiwan), SRR5239763 (mouth, Taiwan), SRR8068020 (fluid, Australia), ERR1938049 (NA, Denmark), ERR1938045 (NA, Denmark)

CAGL0G 04895g	G	470015	470542	+	6	Ortholog(s) have actin filament binding activity	ARC18 (YLR370C)	Subunit of the ARP2/3 complex; ARP2/3 is required for the motility and integrity of cortical actin patches	no	with non-synonymous variants	SRR3154166 (blood, Australia), SRR3925733 (NA, NA), SRR3925744 (NA, NA), SRR3925746 (NA, NA), SRR3925749 (NA, NA), SRR3925766 (NA, NA), SRR3925770 (NA, NA), SRR3925773 (NA, NA)
CAGL0G 07529g	G	713742	714065	+	6	Ortholog(s) have unfolded protein binding activity, role in cytoskeleton organization, positive regulation of transcription elongation from RNA polymerase II promoter, protein folding and prefoldin complex localization	PFD1 (YJL179W)	Subunit of heterohexameric prefoldin; prefoldin binds cytosolic chaperonin and transfers target proteins to it; involved in the biogenesis of actin and of alpha- and gamma-tubulin; prefoldin complex also localizes to chromatin of actively transcribed genes in the nucleus and facilitates transcriptional elongation	no	with non-synonymous variants	ERR1938051, ERR1938057, ERR1938058, ERR1938068, ERR1938070, ERR1938074, ERR1938075, ERR1938078, ERR1938081, ERR1938084, ERR1938085, ERR1938088, ---CHECK some non-synonymous mutations in our genomes!
CAGL0H 04301g	H	406981	407529	+	7	Ortholog(s) have peptidyl-prolyl cis-trans isomerase activity, role in apoptotic process, protein folding and mitochondrion localization	CPR3 (YML078W)	Mitochondrial peptidyl-prolyl cis-trans isomerase (cyclophilin); catalyzes the cis-trans isomerization of peptide bonds N-terminal to proline residues; involved in protein refolding after import into mitochondria	no	with non-synonymous variants	SRR3154166 (blood, Australia), SRR3154234 (blood, Australia), SRR3925733 (NA, NA), SRR3925744 (NA, NA), SRR3925746 (NA, NA), SRR3925749 (NA, NA), SRR3925766 (NA, NA), SRR3925770 (NA, NA), SRR5239762 (mouth, Taiwan), SRR5239763 (mouth, Taiwan), SRR5239778 (blood, USA), SRR8068022 (blood, Australia), SRR3925756 (NA, NA)

CAGL0I05544g	I	524951	526099	-	16	Ortholog(s) have ATP binding, ATPase activity, DNA binding, DNA-dependent ATPase activity, Swi5-Sfr1 complex binding, double-stranded DNA binding, recombinase activity, single-stranded DNA binding activity	RAD51 (YER095W)	Strand exchange protein; forms helical filament with DNA that searches for homology; involved in recombinational repair of DNA double-strand breaks during vegetative growth and meiosis; direct phosphorylation by Cdc28p in G2/M phase promotes DNA binding, strand invasion, and primer extension; homolog of Dmc1p and bacterial RecA protein; RAD51 and RAD4 pathways confer resistance to benzo[a]pyrene dihydrodiol	no	with non-synonymous variants	SRR8068056 (blood, Australia)
CAGL0I05874g	I	558105	558845	-	16	Haloacid dehalogenase-like hydrolase	GPP1 (YIL053W)	Constitutively expressed DL-glycerol-3-phosphate phosphatase; also known as glycerol-1-phosphatase; involved in glycerol biosynthesis, induced in response to both anaerobic and osmotic stress; GPP1 has a paralog, GPP2, that arose from the whole genome duplication	no (but has a paralog)	with non-synonymous variants	SRR5239773 (blood, France), ERR1938055 (NA, NA)
CAGL0K04477g	K	424537	425460	+	11	Ortholog(s) have C-4 methylsterol oxidase activity, role in ergosterol biosynthetic process and endoplasmic reticulum membrane, plasma membrane localization	ERG25 (YGR060W)	C-4 methyl sterol oxidase; catalyzes the first of three steps required to remove two C-4 methyl groups from an intermediate in ergosterol biosynthesis; mutants accumulate the sterol intermediate 4,4-dimethylzymosterol; human MSMO1 functionally complements the growth defect caused by repression of ERG25 expression	yes	with non-synonymous variants	SRR3151591 (fluid, Australia), SRR4198629 (pelvis, Australia), SRR8068027 (fluid, Australia)
CAGL0K12100g	K	1184212	1185192	+	9	Putative coproporphyrinogen	HEM13 (YDR044W	Coproporphyrinogen III oxidase; oxygen-requiring	no	with non-synonymous	ERR1938078 (NA, Denmark), SRR3154237

						III oxidase; protein differentially expressed in azole resistant strain	)	enzyme that catalyzes sixth step in heme biosynthetic pathway; transcription is repressed by oxygen and heme (via Rox1p and Hap1p); human homolog CPOX can complement yeast mutant and allow growth of haploid null after sporulation of a heterozygous diploid		variants	(blood, Australia)
CAGL0L 00759g	L	95845	96762	+	11	HIS1; ATP phosphoribosyltransf erase; protein abundance increased in ace2 mutant cells	HIS1 (YER055C)	ATP phosphoribosyltransferase; a hexameric enzyme, catalyzes the first step in histidine biosynthesis; mutations cause histidine auxotrophy and sensitivity to Cu, Co, and Ni salts; transcription is regulated by general amino acid control	no	with non-synonymous variants	SRR3151591 (fluid, Australia), SRR3154166 (blood, Australia), SRR8068027 (fluid, Australia), SRR8068034 (blood, Australia)
CAGL0L 07722g	L	849746	850993	-	8	PGK1; Putative 3-phosphoglycerate kinase; protein differentially expressed in azole resistant strain; protein abundance increased in ace2 mutant cells	PGK1 (YCR012W)	3-phosphoglycerate kinase; catalyzes transfer of high-energy phosphoryl groups from the acyl phosphate of 1,3-bisphosphoglycerate to ADP to produce ATP; key enzyme in glycolysis and gluconeogenesis	yes	with non-synonymous variants	SRR8068027 (fluid, Australia), SRR3151591 (fluid, Australia)
CAGL0L 11792g	L	1261571	1263307	+	17	Ortholog(s) have poly(A) binding, promoter-specific chromatin binding, protein kinase A catalytic subunit binding, ribonuclease inhibitor activity	PAB1 (YER165W)	Poly(A) binding protein; interacts with the cleavage factor complex CF I, mediates interactions between the 5' cap structure and the 3' mRNA poly(A) tail, involved in control of poly(A) tail length, interacts with translation factor eIF-4G; stimulates, but is not required for the deadenylation activity of the Pan2p-Pan3p poly(A)-ribonuclease complex	yes	with non-synonymous variants	ERR1938057 (NA, NA), SRR8068020 (fluid, Australia)

~~Highly variable~~ (dN/dS>1)

Candida glabrata ID	chromosome	Start coordinate (REF)	End coordinate (REF)	strand	description	*S. cerevisiae* ortholog	description of *S. cerevisiae* ortholog	Essential in *S. cerevisiae*
CAGL0D00374g	D	45775	46083	+	Protein of unknown function	NA	NA	NA
CAGL0I04136g	I	364420	364821	+	Has domain(s) with predicted cytochrome-c oxidase activity	MTC3 (YGL226W)	Protein of unknown function; green fluorescent protein (GFP)-fusion protein localizes to the mitochondrion; mtc3 is synthetically sick with cdc13-1	no
CAGL0K07183g	K	705897	706217	+	Protein of unknown function	NA	NA	NA
CAGL0L07194g	L	800960	801427	+	Protein of unknown function	NA	NA	NA
CAGL0L08068g	L	883198	883439	-	Ortholog(s) have single-stranded DNA binding activity, role in mitochondrial genome maintenance, positive regulation of mitochondrial DNA replication and mitochondrial nucleoid, mitochondrion localization	RIM1 (YCR028C-A)	ssDNA-binding protein essential for mitochondrial genome maintenance; involved in mitochondrial DNA replication; stimulates utilization by Mip1p DNA polymerase of RNA primers synthesized by Rpo41p	no

Back to the index

Supplementary information

Supplementary Table 4

Associations between genetic variations (non-synonymous mutations) in genes known to be associated with the resistance to antifungals and the susceptibility of the strains isolated in this study against the tested antifungals.

Table S4

Associations between genetic variations (non-synonymous mutations) in genes known to be associated with the resistance to antifungals and the susceptibility of the strains isolated over this study against the tested antifungals.

summary_of_associations: summary of associations between not-synonymous mutations in genes known to be associated with antifungal resistance and phenotype (response to the tested antifungals). Red= potential associations. “Drug resist. Type”= the gene is associated with the resistance to this antifungal or class of antifungals; Aa mutation= not-synonymous mutation with position (relative to the coordinate of the reference gene); Fluconazole R if EC50>16mg/l, I if 0.001mg/l<EC50<16mg/l; S if EC50<0.001; Voriconazole R if EC50>1mg/l, S if EC50<1mg/l; Caspofungin R if EC50>0.5mg/l, I if 0.125mg/l<EC50<0.5mg/l, S if EC50<0.125mg/l; Flucytosine R if EC50>0.25mg/l, S if EC50<0.25mg/l; the number of strains classified according to each classification (R/I/S) and bearing the corresponding not-synonymous mutation is reported as the numerator in the ratio over the total number of strains showing the same response.

For each strain, it is also reported the corresponding EC50 of the tested antifungals. The numbers in the first row indicate the coordinate (in the gene of the reference strain) of the aminoacid:

CDR1_Aa_variations. Aminoacid variants in the CDR1 gene found in at least one of the 30 strains sequenced over this study.
ERG9_Aa_variations. Aminoacid variants in the ERG9 gene found in at least one of the 30 strains sequenced over this study.
ERG11_Aa_variations. Aminoacid variants in the ERG11 gene found in at least one of the 30 strains sequenced over this study.
FCY2_Aa_variations. Aminoacid variants in the FCY2 gene found in at least one of the 30 strains sequenced over this study.
FKS1_Aa_variations. Aminoacid variants in the FKS1 gene found in at least one of the 30 strains sequenced over this study.
FKS2_Aa_variations. Aminoacid variants in the FKS2 gene found in at least one of the 30 strains sequenced over this study.
FKS3_Aa_variations. Aminoacid variants in the FKS3 gene found in at least one of the 30 strains sequenced over this study.
FLR1_Aa_variations. Aminoacid variants in the FLR1 gene found in at least one of the 30 strains sequenced over this study.
FPS1_Aa_variations. Aminoacid variants in the FPS1 gene found in at least one of the 30 strains sequenced over this study.
FPS2_Aa_variations. Aminoacid variants in the FPS2 gene found in at least one of the 30 strains sequenced over this study.
PDR1_Aa_variations. Aminoacid variants in the PDR1 gene found in at least one of the 30 strains sequenced over this study.
SNQ2_Aa_variations. Aminoacid variants in the SNQ2 gene found in at least one of the 30 strains sequenced over this study.

Note: to navigate through the file, click on the underlined text

Summary of associations

gene	Drug resist. Type	Aa mutation	strains with mutation	Fluconazole			Voriconazole		Caspofungin			Flucytosine	
				R	I	S	R	S	R	I	S	R	S
CDR1	azoles	Thr16Ile	1	0/18	1/12	0/0	0/13	1/17	0/1	1/22	0/7	0/19	1/11
CDR1	azoles	His58Tyr	11	4/18	7/12	0/0	5/13	6/17	1/1	9/22	1/7	6/19	5/11
CDR1	azoles	His58Asn	2	1/18	1/12	0/0	1/13	1/17	0/1	1/22	1/7	2/19	0/11
CDR1	azoles	His240Gln	1	1/18	0/12	0/0	1/13	0/17	0/1	1/22	0/7	1/19	0/11
CDR1	azoles	His271Arg	1	0/18	1/12	0/0	0/13	1/17	0/1	1/22	0/7	0/19	1/11
CDR1	azoles	Asp248Lys	1	0/18	1/12	0/0	0/13	1/17	0/1	1/22	0/7	1/19	0/11
CDR1	azoles	Asn445Asp	1	1/18	0/12	0/0	0/13	1/17	0/1	0/22	1/7	1/19	0/11
CDR1	azoles	Phe551Ser	1	1/18	0/12	0/0	0/13	1/17	0/1	1/22	0/7	0/19	1/11
CDR1	azoles	Gly787Ser	1	1/18	0/12	0/0	0/13	1/17	0/1	0/22	1/7	1/19	0/11
CDR1	azoles	Asp813Tyr	1	0/18	1/12	0/0	1/13	0/17	0/1	1/22	0/7	1/19	0/11
CDR1	azoles	Ser815Thr	1	1/18	0/12	0/0	0/13	1/17	0/1	0/22	1/7	1/19	0/11
CDR1	azoles	Leu1019Met	2	1/18	1/12	0/0	0/13	2/17	0/1	1/22	1/7	1/19	1/11
CDR1	azoles	Ile1478Met	1	1/18	0/12	0/0	1/13	0/17	0/1	1/22	0/7	1/19	0/11
CDR1	azoles	Thr1158Ser	1	1/18	0/12	0/0	0/13	1/17	0/1	0/22	1/7	1/19	0/11
CDR1	azoles	Ile1478Met	1	1/18	0/12	0/0	0/13	1/17	0/1	1/22	0/7	1/19	0/11
ERG9	azoles	Lys173Thr	1	1/18	0/12	0/0	0/13	1/17	0/1	0/22	1/7	1/19	0/11
ERG9	azoles	Asp236Glu	1	0/18	1/12	0/0	1/13	0/17	0/1	1/22	0/7	0/19	1/11
ERG9	azoles	Lys259Arg	1	0/18	1/12	0/0	0/13	1/17	0/1	1/22	0/7	0/19	1/11
ERG9	azoles	Asn260Lys	1	1/18	0/12	0/0	0/13	1/17	0/1	0/22	1/7	1/19	0/11
ERG9	azoles	Cys344Tyr	9	8/18	1/12	0/0	5/13	4/17	0/1	7/22	2/7	7/19	2/11
ERG9	azoles	Val416Met	1	0/18	1/12	0/0	1/13	0/17	0/1	1/22	0/7	0/19	1/11
ERG9	azoles	Phe424Leu	2	1/18	1/12	0/0	0/13	2/17	0/1	2/22	0/7	1/19	1/11
ERG11	azoles	Leu341Phe	1	0/18	1/12	0/0	0/13	1/17	0/1	122	0/7	1/19	0/11
FCY2	flucytosine	Arg137Leu	1	1/18	0/12	0/0	1/13	0/17	**1/1**	**0/22**	**0/7**	0/19	1/11

FCY2	flucytosine	Ala236Thr	1	0/18	1/12	0/0	1/13	0/17	0/1	1/22	0/7	0/19	1/11
FCY2	flucytosine	Ile384Phe	1	0/18	1/12	0/0	0/13	1/17	0/1	1/22	0/7	0/19	1/11
FKS1	echinocandin	Gly14Ser	8	7/18	1/12	0/0	5/13	3/17	0/1	7/22	1/7	6/19	2/11
FKS1	echinocandin	Leu144Pro	1	1/18	0/12	0/0	1/13	0/17	0/1	1/22	0/7	1/19	0/11
FKS1	echinocandin	Phe625Ile	1	1/18	0/12	0/0	1/13	0/17	0/1	0/22	1/7	0/19	1/11
FKS1	echinocandin	Thr829Ala	1	1/18	0/12	0/0	1/13	0/17	0/1	1/22	0/7	0/19	1/11
FKS1	echinocandin	Glu976Asp	1	0/18	1/12	0/0	1/13	0/17	0/1	1/22	0/7	0/19	1/11
FKS1	echinocandin	Tyr1211Stop	2	1/18	1/12	0/0	0/13	2/17	0/1	2/22	0/7	1/19	1/11
FKS2	echinocandin	Asn16Asp	1	0/18	1/12	0/0	0/13	1/17	0/1	0/22	1/7	1/19	0/11
FKS2	echinocandin	Glu78Asp	4	1/18	3/12	0/0	2/13	2/17	1/1	2/22	1/7	3/19	1/11
FKS2	echinocandin	Ala650Val	1	1/18	0/12	0/0	1/13	0/17	0/1	0/22	1/7	1/19	0/11
FKS2	echinocandin	Phe659Ser	1	1/18	0/12	0/0	1/13	0/17	**1/1**	**0/22**	**0/7**	0/19	1/11
FKS2	echinocandin	Thr926Pro	14	9/18	5/12	0/0	8/13	7/17	1/1	9/22	4/7	11/19	3/11
FKS2	echinocandin	Leu1531Phe	1	1/18	0/12	0/0	1/13	0/17	0/1	1/22	0/7	0/19	1/11
FKS2	echinocandin	Asp1615Glu	2	1/18	1/12	0/0	1/13	1/17	0/1	2/22	0/7	1/19	1/11
FKS2	echinocandin	Tyr1897Stop	1	0/18	1/12	0/0	0/13	1/17	0/1	0/22	1/7	1/19	0/11
FKS3	echinocandin	Ile3Thr	14	6/18	8/12	0/0	4/13	10/17	1/1	8/22	5/7	9/19	5/11
FKS3	echinocandin	Glu40Val	1	0/18	1/12	0/0	0/13	1/17	0/1	0/22	1/7	1/19	0/11
FKS3	echinocandin	Ala42Gly	4	2/18	2/12	0/0	1/13	3/17	0/1	4/22	0/7	3/19	1/11
FKS3	echinocandin	Ala42Val	8	7/18	1/12	0/0	5/13	3/17	0/1	7/22	1/7	6/19	2/11
FKS3	echinocandin	Ile89Val	1	0/18	1/12	0/0	0/13	1/17	0/1	0/22	1/7	1/19	0/11
FKS3	echinocandin	Lys108Asn	1	0/18	1/12	0/0	0/13	1/17	0/1	0/22	1/7	1/19	0/11
FKS3	echinocandin	Ile110Leu	1	0/18	1/12	0/0	0/13	1/17	0/1	0/22	1/7	1/19	0/11
FKS3	echinocandin	Lys187Asn	4	2/18	2/12	0/0	1/13	3/17	0/1	4/22	0/7	3/19	1/11
FKS3	echinocandin	Lys206Glu	1	1/18	0/12	0/0	1/13	0/17	0/1	1/22	0/7	1/19	0/11
FKS3	echinocandin	Asp210Asn	1	1/18	0/12	0/0	1/13	0/17	0/1	1/22	0/7	1/19	0/11
FKS3	echinocandin	Ser212Asn	1	0/18	1/12	0/0	0/13	1/17	0/1	0/22	1/7	1/19	0/11
FKS3	echinocandin	Met271Ile	1	0/18	1/12	0/0	0/13	1/17	0/1	0/22	1/7	1/19	0/11

FKS3	echinocandin	Gly280Glu	1	0/18	1/12	0/0	0/13	1/17	0/1	0/22	1/7	1/19	0/11
FKS3	echinocandin	Met429Ile	1	1/18	0/12	0/0	0/13	1/17	0/1	1/22	0/7	1/19	0/11
FKS3	echinocandin	Val645Ile	1	1/18	0/12	0/0	0/13	1/17	0/1	1/22	0/7	1/19	0/11
FKS3	echinocandin	Glu826Lys	1	0/18	1/12	0/0	1/13	0/17	0/1	1/22	0/7	1/19	0/11
FKS3	echinocandin	Asp829Glu	1	0/18	1/12	0/0	0/13	1/17	0/1	0/22	1/7	1/19	0/11
FKS3	echinocandin	Ala844Thr	1	0/18	1/12	0/0	0/13	1/17	0/1	0/22	1/7	1/19	0/11
FKS3	echinocandin	Asp851His	1	1/18	0/12	0/0	1/13	0/17	0/1	1/22	0/7	1/19	0/11
FKS3	echinocandin	Ser854Arg	2	0/18	2/12	0/0	1/13	1/17	0/1	2/22	0/7	2/19	0/11
FKS3	echinocandin	Gln864Arg	1	1/18	0/12	0/0	0/13	1/17	0/1	0/22	1/7	1/19	0/11
FKS3	echinocandin	Asn865Ser	1	0/18	1/12	0/0	0/13	1/17	0/1	0/22	1/7	1/19	0/11
FKS3	echinocandin	Leu884Val	1	1/18	0/12	0/0	0/13	1/17	0/1	1/22	0/7	0/19	1/11
FKS3	echinocandin	Trp979Asn	1	0/18	1/12	0/0	1/13	0/17	0/1	1/22	0/7	0/19	1/11
FKS3	echinocandin	Arg1039Leu	1	1/18	0/12	0/0	0/13	1/17	0/1	0/22	1/7	1/19	0/11
FKS3	echinocandin	Asn1301Tyr	13	6/18	6/12	0/0	4/13	9/17	1/1	8/22	4/7	8/19	5/11
FKS3	echinocandin	Arg1472Gln	9	4/18	5/12	0/0	3/13	6/17	1/1	4/22	4/7	5/19	4/11
FKS3	echinocandin	Ile1512Val	1	1/18	0/12	0/0	0/13	1/17	0/1	1/22	0/7	1/19	0/11
FKS3	echinocandin	Thr1676Ser	3	2/18	1/12	0/0	1/13	2/17	0/1	2/22	1/7	0/19	3/11
FKS3	echinocandin	Thr1686Ser	10	8/18	2/12	0/0	6/13	4/17	0/1	9/22	1/7	7/19	3/11
FKS3	echinocandin	Tyr1731Cys	10	4/18	6/12	0/0	3/13	7/17	1/1	4/22	5/7	6/19	4/11
FKS3	echinocandin	Val1764Ala	1	1/18	0/12	0/0	0/13	1/17	0/1	1/22	0/7	1/19	0/11
FKS3	echinocandin	Phe1768Ile	4	2/18	2/12	0/0	1/13	3/17	0/1	4/22	0/7	3/19	1/11
FKS3	echinocandin	Asn1825Ser	1	1/18	0/12	0/0	0/13	1/17	0/1	1/22	0/7	0/19	1/11
FKS3	echinocandin	Ser1840Tyr	8	3/18	5/12	0/0	3/13	5/17	1/1	4/22	3/7	4/19	4/11
FLR1	azoles	Ser50gap	3	2/18	1/12	0/0	1/13	2/17	0/1	2/22	1/7	0/19	3/11
FLR1	azoles	Met103Ile	13	8/18	5/12	0/0	7/13	6/17	0/1	10/22	3/7	9/19	4/11
FLR1	azoles	Ile116Met	13	8/18	5/12	0/0	7/13	6/17	0/1	10/22	3/7	9/19	4/11
FLR1	azoles	Ile186Val	1	0/18	1/12	0/0	1/13	0/17	0/1	1/22	0/7	1/19	0/11
FLR1	azoles	Val254Ile	5	2/18	3/12	0/0	1/13	4/17	0/1	4/22	1/7	3/19	2/11

FLR1	azoles	Val254Leu	1	1/18	0/12	0/0	0/13	1/17	0/1	0/22	1/7	1/19	0/11
FLR1	azoles	Gln326Leu	1	1/18	0/12	0/0	0/13	1/17	0/1	1/22	0/7	0/19	1/11
FLR1	azoles	Phe332Val	1	0/18	1/12	0/0	0/13	1/17	0/1	1/22	0/7	1/19	0/11
FLR1	azoles	Asp354Asn	1	1/18	0/12	0/0	0/13	1/17	0/1	1/22	0/7	1/19	0/11
FLR1	azoles	Arg496stop	1	1/18	0/12	0/0	0/13	1/17	0/1	1/22	0/7	1/19	0/11
FPS1	flucytosine	Ala316Ser	8	5/18	3/12	0/0	1/13	7/17	0/1	6/22	2/7	4/19	4/11
FPS2	flucytosine	Asn17Asp	11	8/18	3/12	0/0	6/13	5/17	0/1	8/22	3/7	9/19	2/11
FPS2	flucytosine	Ala85Val	1	1/18	0/12	0/0	0/13	1/17	0/1	0/22	1/7	1/19	0/11
FPS2	flucytosine	His128Asn	8	7/18	1/12	0/0	5/13	3/17	0/1	6/22	2/7	6/19	2/11
FPS2	flucytosine	His128Tyr	1	1/18	0/12	0/0	0/13	1/17	0/1	1/22	0/7	1/19	0/11
FPS2	flucytosine	Ala227Ser	3	1/18	2/12	0/0	1/13	2/17	0/1	1/22	2/7	3/19	0/11
FPS2	flucytosine	Ile228Val	1	0/18	1/12	0/0	0/13	1/17	0/1	1/22	0/7	0/19	1/11
FPS2	flucytosine	Ala409Thr	11	8/18	3/12	0/0	6/13	5/17	0/1	8/22	3/7	9/19	2/11
FPS2	flucytosine	Lys474Arg	12	8/18	4/12	0/0	7/13	5/17	0/1	9/22	3/7	9/19	3/11
FPS2	flucytosine	Asp511Glu	1	1/18	0/12	0/0	0/13	1/17	0/1	1/22	0/7	0/19	1/11
FPS2	flucytosine	Asp517Tyr	8	7/18	1/12	0/0	5/13	3/17	0/1	7/22	1/7	6/19	2/11
PDR1	azoles	Ser75Pro	21	11/18	10/12	0/0	8/13	13/17	1/1	14/22	6/7	13/19	8/11
PDR1	azoles	Val91Ile	30	18/18	12/12	0/0	13/13	17/17	1/1	22/22	7/7	19/19	11/11
PDR1	azoles	Leu98Ser	30	18/18	12/12	0/0	13/13	17/17	1/1	22/22	7/7	19/19	11/11
PDR1	azoles	Val134Ala	1	0/18	1/12	0/0	0/13	1/17	0/1	1/22	0/7	0/19	1/11
PDR1	azoles	Thr143Pro	21	11/18	10/12	0/0	8/13	13/17	1/1	14/22	6/7	13/19	8/11
PDR1	azoles	Gly189Val	1	0/18	1/12	0/0	1/13	0/17	0/1	1/22	0/7	0/19	1/11
PDR1	azoles	Asp243Asn	8	7/18	1/12	0/0	5/13	3/17	0/1	7/22	1/7	6/19	2/11
PDR1	azoles	Asn255K	1	0/18	1/12	0/0	0/13	1/17	0/1	0/22	1/7	1/19	0/11
PDR1	azoles	Asn307Lys	1	1/18	0/12	0/0	0/13	1/17	0/1	1/22	0/7	0/19	1/11
PDR1	azoles	His308Tyr	1	1/18	0/12	0/0	0/13	1/17	0/1	1/22	0/7	0/19	1/11
PDR1	azoles	Gln337Arg	1	1/18	0/12	0/0	1/13	0/17	0/1	0/22	1/7	1/19	0/11
PDR1	azoles	Ser343Phe	1	1/18	0/12	0/0	1/13	0/17	0/1	1/22	0/7	1/19	0/11

PDR1	azoles	Gln345Lys	1	1/18	0/12	0/0	1/13	0/17	0/1	1/22	0/7	1/19	0/11
PDR1	azoles	Met461Ile	1	0/18	1/12	0/0	0/13	1/17	0/1	0/22	1/7	1/19	0/11
PDR1	azoles	Met540Ile	1	1/18	0/12	0/0	1/13	0/17	0/1	1/22	0/7	0/19	1/11
PDR1	azoles	Thr745Ala	2	1/18	1/12	0/0	0/13	2/17	0/1	0/22	2/7	2/19	0/11
PDR1	azoles	Thr745gap	1	1/18	0/12	0/0	0/13	1/17	0/1	0/22	1/7	1/19	0/11
PDR1	azoles	Leu935Ser	1	1/18	0/12	0/0	1/13	0/17	**1/1**	**0/22**	**0/7**	0/19	1/11
PDR1	azoles	Leu935gap	1	1/18	0/12	0/0	0/13	1/17	0/1	1/22	0/7	1/19	0/11
PDR1	azoles	Ile939Val	1	1/18	0/12	0/0	0/13	1/17	0/1	1/22	0/7	0/19	1/11
PDR1	azoles	Ile939gap	1	1/18	0/12	0/0	0/13	1/17	0/1	1/22	0/7	1/19	0/11
PDR1	azoles	Leu1090Ser	1	0/18	1/12	0/0	0/13	1/17	0/1	0/22	1/7	1/19	0/11
PDR1	azoles	Leu1090gap	1	1/18	0/12	0/0	0/13	1/17	0/1	1/22	0/7	1/19	0/11
SNQ2	azoles	Arg433Ser	1	1/18	0/12	0/0	1/13	0/17	**1/1**	**0/22**	**0/7**	0/19	1/11
SNQ2	azoles	Phe633Ser	1	1/18	0/12	0/0	1/13	0/17	**1/1**	**0/22**	**0/7**	0/19	1/11
SNQ2	azoles	Arg634Glu	1	1/18	0/12	0/0	1/13	0/17	**1/1**	**0/22**	**0/7**	0/19	1/11
SNQ2	azoles	Val635Tyr	1	1/18	0/12	0/0	1/13	0/17	**1/1**	**0/22**	**0/7**	0/19	1/11
SNQ2	azoles	Tyr636Thr	1	1/18	0/12	0/0	1/13	0/17	**1/1**	**0/22**	**0/7**	0/19	1/11
SNQ2	azoles	Leu637Ser	1	1/18	0/12	0/0	1/13	0/17	**1/1**	**0/22**	**0/7**	0/19	1/11
SNQ2	azoles	Phe638Ser	1	1/18	0/12	0/0	1/13	0/17	**1/1**	**0/22**	**0/7**	0/19	1/11
SNQ2	azoles	Leu639stop	1	1/18	0/12	0/0	1/13	0/17	**1/1**	**0/22**	**0/7**	0/19	1/11
SNQ2	azoles	Ile700Phe	1	0/18	1/12	0/0	0/13	1/17	0/1	1/22	0/7	1/19	0/11
SNQ2	azoles	Ala703Gly	1	1/18	0/12	0/0	1/13	0/17	0/1	0/22	1/7	1/19	0/11
SNQ2	azoles	Glu1000Asp	1	0/18	1/12	0/0	0/13	1/17	0/1	0/22	1/7	1/19	0/11
SNQ2	azoles	Leu1005Ser	1	1/18	0/12	0/0	1/13	0/17	0/1	0/22	1/7	0/19	1/11
SNQ2	azoles	Asp1085Asn	1	0/18	1/12	0/0	1/13	0/17	0/1	1/22	0/7	0/19	1/11
SNQ2	azoles	His1147Gln	1	0/18	1/12	0/0	0/13	1/17	0/1	1/22	0/7	0/19	1/11
SNQ2	azoles	Lys1156Gln	1	0/18	1/12	0/0	0/13	1/17	0/1	1/22	0/7	0/19	1/11
SNQ2	azoles	Ser1506Pro	1	1/18	0/12	0/0	1/13	0/17	0/1	1/22	0/7	1/19	0/11

CDR1_Aa_variations. Aminoacid variants in the CDR1 gene found in at least one of the 30 strains sequenced over this study.

strain		16	58	240	271	248	445	551	787	813	815	1019	1478	1158	1478	Fluconazole (S<0.001; R>16)[3]	Voriconazole (ECOFF=1)[4]	Caspofungin (S≤0.125, R>0.5)[2]	Flucytosine (S≤0.25)[1]
6687_CDR1	CG_UHB_01	T	H	H	H	N	D	F	G	N	S	L	M	T	I	24.992826459	0.0292335204	0.2442450412	0.338206565813
5877_CDR1	CG_UHB_02	T	Y	H	H	N	D	F	G	N	S	L	I	T	I	75.256762069	10.0175324564	0.1428591927	0.463307194328
3165_CDR1	CG_UHB_03	T	Y	H	H	N	D	F	G	N	S	L	M	T	I	6.485379354	5.0361687161	0.1750437573	0.222244322763
6958_CDR1	CG_UHB_04	T	Y	H	H	N	D	F	G	N	S	L	M	T	I	28.038581483	2.3531077481	1.9226507595	0.230171702338
5916_CDR1	CG_UHB_05	T	H	H	H	N	D	F	G	N	S	M	M	T	I	0.0175335349	0.0771340654	0.1112586026	0.315609593781
3380_CDR1	CG_UHB_06	T	H	H	H	N	D	F	G	N	S	L	M	T	I	21.513628533	0.8285869773	0.1465884888	0.51479943005
7051_CDR1	CG_UHB_07	T	H	H	H	N	D	F	G	N	S	L	M	T	I	53.382429003	1.7163482074	0.1078147722	0.248789259094
2821_CDR1	CG_UHB_08	T	Y	H	H	N	D	F	G	Y	S	L	M	T	I	1.4084005883	1.9798090513	0.1362509252	0.357580700276
9296_CDR1	CG_UHB_09	T	H	H	H	N	D	F	G	N	S	L	M	T	M	33.097918309	0.0024313751	0.1398946985	0.254800566985
2390_CDR1	CG_UHB_10	T	N	H	H	N	N	F	G	N	T	L	M	T	I	44.230069257	0.7647018742	0.1116464109	0.268614925351
5852_CDR1	CG_UHB_11	T	H	H	H	N	D	F	G	N	S	L	M	T	I	41.622568289	0.3158076542	0.2502661914	0.231487375204
2362_CDR1	CG_UHB_12	T	H	H	H	N	D	F	S	N	S	L	M	S	I	46.697201766	0.862347048	0.0980061073	0.276120791094
7805_CDR1	CG_UHB_13	T	N	H	H	N	D	F	G	N	S	L	M	T	I	4.1825530175	2.9496066212	0.1592401497	0.254940462661
1008_2_CDR1	CG_UHB_14	T	Y	H	H	N	D	F	G	N	S	L	M	T	I	2.3460137588	0.2572101048	0.1148310572	0.255128601536
6769_CDR1	CG_UHB_15	T	H	H	H	N	D	F	G	N	S	L	M	T	I	30.722437937	2.8454505973	0.1484817795	0.330173641052
S07_CDR1	CG_UHB_16	T	Y	H	H	N	D	F	G	N	S	L	M	T	I	50.601449711	0.6201736152	0.131398177	0.519292978593
S04_CDR1	CG_UHB_17	T	Y	H	H	N	D	F	G	N	S	M	M	T	I	27.805362103	0.8933812651	0.1641645327	0.196297425094
S01_CDR1	CG_UHB_18	T	H	H	H	N	D	S	G	N	S	L	M	T	I	25.877517864	0.0278081202	0.3612798088	0.226123850101
S14_CDR1	CG_UHB_19	T	H	H	H	N	D	F	G	N	S	L	M	T	I	46.792660356	8	0.1430666604	0.551207404981
S16_CDR1	CG_UHB_20	T	H	H	H	N	D	F	G	N	S	L	M	T	I	46.792660356	1.8073399474	0.1473993519	0.254041886441
S09_CDR1	CG_UHB_21	I	Y	H	R	N	D	F	G	N	S	L	M	T	I	1.0081444478	0.1090642362	0.1316574475	0.229206981372
S11_CDR1	CG_UHB_22	T	Y	H	H	K	D	F	G	N	S	L	M	T	I	9.5958707786	0.0645033266	0.2462582915	0.540216751762
S13_CDR1	CG_UHB_24	T	H	H	H	N	D	F	G	N	S	L	M	T	I	110.20258619	1.0435264917	0.1844128702	0.220850648581
S05_CDR1	CG_UHB_25	T	H	H	H	N	D	F	G	N	S	L	M	T	I	1.9977305272	0.1272006695	0.135539175	0.630044611458
S03_CDR1	CG_UHB_26	T	H	Q	H	N	D	F	G	N	S	L	M	T	I	43.577880403	3.6122595201	0.1893451974	0.183280622211
S02_CDR1	CG_UHB_27	T	Y	H	H	N	D	F	G	N	S	L	M	T	I	0.7108136564	0.0579915138	0.1512232029	0.278898079923
S12_CDR1	CG_UHB_27	T	Y	H	H	N	D	F	G	N	S	L	M	T	I	12.430878476	8	0.2108859976	0.201750584959
S08_CDR1	CG_UHB_28	T	H	H	H	N	D	F	G	N	S	L	M	T	I	30.283985743	3.1878757748	0.0994995657	0.639822196409
S10_CDR1	CG_UHB_29	T	H	H	H	N	D	F	G	N	S	L	M	T	I	2.2987638742	0.7853533034	0.0898027763	0.272406238885
S15_CDR1	CG_UHB_30	T	H	H	H	N	D	F	G	N	S	L	M	T	I	3.8802347424	0.0156744012	0.1747224101	0.168898845789
REF-CBS138|CDR1	REF	T	H	H	H	N	D	F	G	N	S	L	M	T	I				

ERG9_Aa_variations. Aminoacid variants in the ERG9 gene found in at least one of the 30 strains sequenced over this study.

strain		173	236	259	260	344	416	424	Fluconazole (S<0.001; R>16)[3]	Voriconazole (ECOFF=1)[4]	Caspofungin (S≤0.125, R>0.5)[2]	Flucytosine (S≤0.25)[1]
>6687_ERG9	CG_UHB_01	K	D	K	N	Y	V	L	24.992826459	0.0292335204	0.2442450412	0.33820656581264
>5877_ERG9	CG_UHB_02	K	D	K	N	C	V	F	75.256762069	10.0175324564	0.1428591927	0.46330719432754
>3165_ERG9	CG_UHB_03	K	E	K	N	C	V	F	6.485379354	5.0361687161	0.1750437573	0.22224432276299
>6958_ERG9	CG_UHB_04	K	D	K	N	C	V	F	28.038581483	2.3531077481	1.9226507595	0.23017170233811
>5916_ERG9	CG_UHB_05	K	D	K	N	C	V	F	0.0175335349	0.0771340654	0.1112586026	0.31560959378108
>3380_ERG9	CG_UHB_06	K	D	K	N	Y	V	F	21.513628533	0.8285869773	0.1465884888	0.51479943004991
>7051_ERG9	CG_UHB_07	K	D	K	N	C	V	F	53.382429003	1.7163482074	0.1078147722	0.24878925909414
>2821_ERG9	CG_UHB_08	K	D	K	N	C	V	F	1.4084005883	1.9798090513	0.1362509252	0.35758070027615
>9296_ERG9	CG_UHB_09	K	D	K	N	C	V	F	33.097918309	0.0024313751	0.1398946985	0.25480056698541
>2390_ERG9	CG_UHB_10	T	D	K	N	C	V	F	44.230069257	0.7647018742	0.1116464109	0.26861492535113
>5852_ERG9	CG_UHB_11	K	D	K	N	C	V	F	41.622568269	0.3158076542	0.2502661914	0.23148737520416
>2362_ERG9	CG_UHB_12	K	D	K	K	Y	V	F	46.697201766	0.862347048	0.0980061073	0.27612079109376
>7805_ERG9	CG_UHB_13	K	D	K	N	C	V	F	4.1825530175	2.9496066212	0.1592401497	0.25494046266135
>1008_2_ERG9	CG_UHB_14	K	D	K	N	C	V	F	2.3460137588	0.2572101048	0.1148310572	0.25512860153569
>6769_ERG9	CG_UHB_15	K	D	K	N	C	V	F	30.722437937	2.8454505973	0.1484817795	0.33017364105177
>S07_ERG9	CG_UHB_16	K	D	K	N	C	V	F	50.601449711	0.6201736152	0.131398177	0.51929297859304
>S04_ERG9	CG_UHB_17	K	D	K	N	C	V	F	27.805362103	0.8933812651	0.1641645327	0.1962974250942
>S01_ERG9	CG_UHB_18	K	D	K	N	C	V	F	25.877517864	0.0278081202	0.3612798088	0.22612385010122
>S14_ERG9	CG_UHB_19	K	D	K	N	Y	V	F	46.792660356	8	0.1430666604	0.55120740498098
>S16_ERG9	CG_UHB_20	K	D	K	N	Y	V	F	46.792660356	1.8073399474	0.1473993519	0.25404188644058
>S09_ERG9	CG_UHB_21	K	D	R	N	C	V	L	1.0081444478	0.1090642362	0.1316574475	0.22920698137162
>S11_ERG9	CG_UHB_22	K	D	K	N	C	V	F	9.5958707786	0.0645033266	0.2462582915	0.54021675176234
>S13_ERG9	CG_UHB_24	K	D	K	N	Y	V	F	110.20258619	1.0435264917	0.1844128702	0.22085064858132
>S05_ERG9	CG_UHB_25	K	D	K	N	Y	V	F	1.9977305272	0.1272006695	0.135539175	0.6300446114581
>S03_ERG9	CG_UHB_26	K	D	K	N	Y	V	F	43.577880403	3.6122595201	0.1893451974	0.18328062221057
>S02_ERG9	CG_UHB_27	K	D	K	N	C	V	F	0.7108136564	0.0579915138	0.1512232029	0.27889807992321
>S12_ERG9	CG_UHB_27	K	D	K	N	C	M	F	12.430878476	8	0.2108859976	0.20175058495854
>S08_ERG9	CG_UHB_28	K	D	K	N	Y	V	F	30.283985743	3.1878757748	0.0994995657	0.63982219640928
>S10_ERG9	CG_UHB_29	K	D	K	N	C	V	F	2.2987638742	0.7853533034	0.0898027763	0.2724062388854
>S15_ERG9	CG_UHB_30	K	D	K	N	C	V	F	3.8802347424	0.0156744012	0.1747224101	0.16889884578903
>REF-CBS138|ERG9	REF	K	D	K	N	C	V	F				

ERG11_Aa_variations. Aminoacid variants in the ERG11 gene found in at least one of the 30 strains sequenced over this study.

strain		341	Fluconazole (S<0.001; R>16)[3]	Voriconazole (ECOFF=1)[4]	Caspofungin (S≤0.125, R>0.5)[2]	Flucytosine (S≤0.25)[1]
6687_CDR1	CG_UHB_01	L	24.9928264591	0.0292335204	0.2442450412	0.338206565813
5877_CDR1	CG_UHB_02	L	75.2567620688	10.0175324564	0.1428591927	0.463307194328
3165_CDR1	CG_UHB_03	L	6.485379354	5.0361687161	0.1750437573	0.222244322763
6958_CDR1	CG_UHB_04	L	28.0385814828	2.3531077481	1.9226507595	0.230171702338
5916_CDR1	CG_UHB_05	L	0.0175335349	0.0771340654	0.1112586026	0.315609593781
3380_CDR1	CG_UHB_06	L	21.5136285325	0.8285869773	0.1465884888	0.51479943005
7051_CDR1	CG_UHB_07	L	53.3824290034	1.7163482074	0.1078147722	0.248789259094
2821_CDR1	CG_UHB_08	L	1.4084005883	1.9798090513	0.1362509252	0.357580700276
9296_CDR1	CG_UHB_09	L	33.097918309	0.0024313751	0.1398946985	0.254800566985
2390_CDR1	CG_UHB_10	L	44.2300692573	0.7647018742	0.1116464109	0.268614925351
5852_CDR1	CG_UHB_11	L	41.6225682891	0.3158076542	0.2502661914	0.231487375204
2362_CDR1	CG_UHB_12	L	46.6972017657	0.862347048	0.0980061073	0.276120791094
7805_CDR1	CG_UHB_13	L	4.1825530175	2.9496066212	0.1592401497	0.254940462661
1008_2_CDR1	CG_UHB_14	L	2.3460137588	0.2572101048	0.1148310572	0.255128601536
6769_CDR1	CG_UHB_15	L	30.7224379372	2.8454505973	0.1484817795	0.330173641052
S07_CDR1	CG_UHB_16	L	50.6014497112	0.6201736152	0.131398177	0.519292978593
S04_CDR1	CG_UHB_17	L	27.8053621027	0.8933812651	0.1641645327	0.196297425094
S01_CDR1	CG_UHB_18	L	25.8775178635	0.0278081202	0.3612798088	0.226123850101
S14_CDR1	CG_UHB_19	L	46.7926603557	8	0.1430666604	0.551207404981
S16_CDR1	CG_UHB_20	L	46.7926603557	1.8073399474	0.1473993519	0.254041886441
S09_CDR1	CG_UHB_21	L	1.0081444478	0.1090642362	0.1316574475	0.229206981372
S11_CDR1	CG_UHB_22	F	9.5958707786	0.0645033266	0.2462582915	0.540216751762
S13_CDR1	CG_UHB_24	L	110.202588191	1.0435264917	0.1844128702	0.220850648581
S05_CDR1	CG_UHB_25	L	1.9977305272	0.1272006695	0.135539175	0.630044611458
S03_CDR1	CG_UHB_26	L	43.5778804033	3.6122595201	0.1893451974	0.183280622211
S02_CDR1	CG_UHB_27	L	0.7108136564	0.0579915138	0.1512232029	0.278898079923
S12_CDR1	CG_UHB_27	L	12.4308784764	8	0.2108859976	0.201750584959
S08_CDR1	CG_UHB_28	L	30.2839857427	3.1878757748	0.0994995657	0.639822196409
S10_CDR1	CG_UHB_29	L	2.2987638742	0.7853533034	0.0898027763	0.272406238885
S15_CDR1	CG_UHB_30	L	3.8802347424	0.0156744012	0.1747224101	0.168898845789

FCY2_Aa_variations. Aminoacid variants in the FCY2 gene found in at least one of the 30 strains sequenced over this study.

		137	236	384	Fluconazole (S<0.001; R>16)[3]	Voriconazole (ECOFF=1)[4]	Caspofungin (S≤0.125, R>0.5)[2]	Flucytosine (S≤0.25)[1]
>6687_FCY2	CG_UHB_01	R	A	I	24.992826459	0.0292335204	0.2442450412	0.3382065658126
>5877_FCY2	CG_UHB_02	R	A	I	75.256762069	10.0175324564	0.1428591927	0.4633071943275
>3165_FCY2	CG_UHB_03	R	A	I	6.485379354	5.0361687161	0.1750437573	0.222244322763
>6958_FCY2	CG_UHB_04	L	A	I	28.038581483	2.3531077481	1.9226507595	0.2301717023381
>5916_FCY2	CG_UHB_05	R	A	I	0.0175335349	0.0771340654	0.1112586026	0.3156095937811
>3380_FCY2	CG_UHB_06	R	A	I	21.513628533	0.8285869773	0.1465884888	0.5147994300499
>7051_FCY2	CG_UHB_07	R	A	I	53.382429003	1.7163482074	0.1078147722	0.2487892590941
>2821_FCY2	CG_UHB_08	R	T	I	1.4084005883	1.9798090513	0.1362509252	0.3575807002762
>9296_FCY2	CG_UHB_09	R	A	I	33.097918309	0.0024313751	0.1398946985	0.2548005669854
>2390_FCY2	CG_UHB_10	R	A	I	44.230069257	0.7647018742	0.1116464109	0.2688149253511
>5852_FCY2	CG_UHB_11	R	A	I	41.622568289	0.3158076542	0.2502661914	0.2314873752042
>2362_FCY2	CG_UHB_12	R	A	I	46.697201766	0.862347048	0.0980061073	0.2761207910938
>7805_FCY2	CG_UHB_13	R	A	I	4.1825530175	2.9496066212	0.1592401497	0.2549404626613
>1008_2_FCY2	CG_UHB_14	R	A	I	2.3460137588	0.2572101048	0.1148310572	0.2551286015357
>6769_FCY2	CG_UHB_15	R	A	I	30.722437937	2.8454505973	0.1484817795	0.3301736410518
>S07_FCY2	CG_UHB_16	R	A	I	50.601449711	0.6201736152	0.131398177	0.519292978593
>S04_FCY2	CG_UHB_17	R	A	I	27.805362103	0.8933812651	0.1641645327	0.1962974250942
>S01_FCY2	CG_UHB_18	R	A	I	25.877517864	0.0278081202	0.3612798088	0.2261238501012
>S14_FCY2	CG_UHB_19	R	A	I	46.792660356	8	0.1430666604	0.551207404981
>S16_FCY2	CG_UHB_20	R	A	I	46.792660356	1.8073399474	0.1473993519	0.2540418864406
>S09_FCY2	CG_UHB_21	R	A	F	1.0081444478	0.1090642362	0.1316574475	0.2292069813716
>S11_FCY2	CG_UHB_22	R	A	I	9.5958707786	0.0645033266	0.2462582915	0.5402167517623
>S13_FCY2	CG_UHB_24	R	A	I	110.20258619	1.0435264917	0.1844128702	0.2208506485813
>S05_FCY2	CG_UHB_25	R	A	I	1.9977305272	0.1272006695	0.135539175	0.6300446114581
>S03_FCY2	CG_UHB_26	R	A	I	43.577880403	3.6122595201	0.1893451974	0.1832806222106
>S02_FCY2	CG_UHB_27	R	A	I	0.7108136564	0.0579915138	0.1512232029	0.2788980799232
>S12_FCY2	CG_UHB_27	R	A	I	12.430878476	8	0.2108859976	0.2017505849585
>S08_FCY2	CG_UHB_28	R	A	I	30.283985743	3.1878757748	0.0994995657	0.6398221964093
>S10_FCY2	CG_UHB_29	R	A	I	2.2987638742	0.7853533034	0.0898027763	0.2724062388854
>S15_FCY2	CG_UHB_30	R	A	I	3.8802347424	0.0156744012	0.1747224101	0.168898845789
>REF-CBS138|FCY2		R	A	I				

FKS1_Aa_variations. Aminoacid variants in the FKS1 gene found in at least one of the 30 strains sequenced over this study.

		14	144	625	829	976	1211	Fluconazole (S<0.001; R>16)[3]	Voriconazole (ECOFF=1)[4]	Caspofungin (S≤0.125, R>0.5)[2]	Flucytosine (S≤0.25)[1]
6687_FKS1	CG_UHB_01	S	F	T	E	L	A	24.9928264591	0.0292335204	0.2442450412	0.3382065658126
5877_FKS1	CG_UHB_02	G	F	T	E	L	A	75.2567620688	10.0175324564	0.1428591927	0.4633071943275
3165_FKS1	CG_UHB_03	G	F	T	E	P	A	6.485379354	5.0361687161	0.1750437573	0.222244322763
6958_FKS1	CG_UHB_04	G	F	T	E	L	A	28.0385814828	2.3531077481	1.9226507595	0.2301717023381
5916_FKS1	CG_UHB_05	G	F	T	E	L	A	0.0175335349	0.0771340654	0.1112586026	0.3156095937811
3380_FKS1	CG_UHB_06	S	F	T	E	L	A	21.5136285325	0.8285869773	0.1465884888	0.5147994300499
7051_FKS1	CG_UHB_07	G	F	A	E	L	A	53.3824290034	1.7163482074	0.1078147722	0.2487892590941
2821_FKS1	CG_UHB_08	G	F	T	E	L	A	1.4084005883	1.9796090513	0.1362509252	0.3575807002762
9296_FKS1	CG_UHB_09	G	F	T	E	L	A	33.097918309	0.0024313751	0.1398946985	0.2548005669854
2390_FKS1	CG_UHB_10	G	F	T	E	L	A	44.2300692573	0.7647018742	0.1116464109	0.2686149253511
5852_FKS1	CG_UHB_11	G	F	T	E	L	A	41.6225682891	0.3158076542	0.2502661914	0.2314873752042
2362_FKS1	CG_UHB_12	G	F	T	E	L	A	46.6972017657	0.862347048	0.0980061073	0.2761207910938
7805_FKS1	CG_UHB_13	G	F	T	E	L	A	4.1825530175	2.9496066212	0.1592401497	0.2549404626613
1008_2_FKS1	CG_UHB_14	G	F	T	E	L	A	2.3460137588	0.2572101048	0.1148310572	0.2551286015357
6769_FKS1	CG_UHB_15	G	F	T	E	L	A	30.7224379372	2.8454505973	0.1484817795	0.3301736410518
S07_FKS1	CG_UHB_16	G	F	T	E	L	A	50.6014497112	0.6201736152	0.131398177	0.519292978593
S04_FKS1	CG_UHB_17	G	F	T	E	L	T	27.8053621027	0.8933812651	0.1641645327	0.1962974250942
S01_FKS1	CG_UHB_18	G	F	T	E	L	A	25.8775178635	0.0278081202	0.3612798088	0.2261238501012
S14_FKS1	CG_UHB_19	S	F	T	E	L	A	46.7926603557	8	0.1430666604	0.551207404981
S16_FKS1	CG_UHB_20	S	L	T	E	L	A	46.7926603557	1.8073399474	0.1473993519	0.2540418864406
S09_FKS1	CG_UHB_21	G	F	T	E	L	A	1.0081444478	0.1090642362	0.1316574475	0.2292069813716
S11_FKS1	CG_UHB_22	G	F	T	E	L	A	9.5958707786	0.0645033266	0.2462582915	0.5402187517623
S13_FKS1	CG_UHB_24	S	F	T	D	L	A	110.202586191	1.0435264917	0.1844128702	0.2208506485813
S05_FKS1	CG_UHB_25	S	F	T	E	L	A	1.9977305272	0.1272006695	0.135539175	0.6300446114581
S03_FKS1	CG_UHB_26	S	F	T	E	L	A	43.5778804033	3.6122595201	0.1893451974	0.1832806222106
S02_FKS1	CG_UHB_27	G	F	T	E	L	T	0.7108136564	0.0579915138	0.1512232029	0.2788980799232
S12_FKS1	CG_UHB_27	G	F	T	E	L	A	12.4308784764	8	0.2108859976	0.2017505849585
S08_FKS1	CG_UHB_28	S	F	T	E	L	A	30.2839857427	3.1878757748	0.0994995657	0.6398221964093
S10_FKS1	CG_UHB_29	G	F	T	E	L	A	2.2987638742	0.7853533034	0.0898027763	0.2724062388854
S15_FKS1	CG_UHB_30	G	F	T	E	L	A	3.8802347424	0.0156744012	0.1747224101	0.168898845789
REF-CAGL0G01034g|FKS1		G	F	T	E	L	A				

FKS2_Aa_variations. Aminoacid variants in the FKS2 gene found in at least one of the 30 strains sequenced over this study.

		16	78	650	659	926	1531	1615	1897	Fluconazole (S<0.001; R>16)[3]	Voriconazole (ECOFF=1)[4]	Caspofungin (S≤0.125, R>0.5)[2]	Flucytosine (S≤0.25)[1]
6687_FKS2	CG_UHB_01	N	E	A	F	P	L	D	I	24.992826459	0.0292335204	0.2442450412	0.338206565813
5877_FKS2	CG_UHB_02	N	E	A	F	T	L	D	I	75.256762069	10.0175324564	0.1428591927	0.463307194328
3165_FKS2	CG_UHB_03	N	E	A	F	T	L	D	I	6.485379354	5.0361687161	0.1750437573	0.222244322763
6958_FKS2	CG_UHB_04	N	D	A	S	P	L	D	I	28.038581483	2.3531077481	1.9226507595	0.230171702338
5916_FKS2	CG_UHB_05	D	E	A	F	P	L	D	I	0.0175335349	0.0771340654	0.1112586026	0.315609593781
3380_FKS2	CG_UHB_06	N	E	A	F	P	L	D	I	21.513628533	0.8285869773	0.1465884888	0.51479943005
7051_FKS2	CG_UHB_07	N	E	A	F	T	L	D	I	53.382429003	1.7163482074	0.1078147722	0.248789259094
2821_FKS2	CG_UHB_08	N	E	A	F	T	L	D	I	1.4084005883	1.9798090513	0.1362509252	0.357580700276
9296_FKS2	CG_UHB_09	N	E	A	F	T	L	D	I	33.097918309	0.0024313751	0.1398946985	0.254800566985
2390_FKS2	CG_UHB_10	N	E	A	F	T	L	D	I	44.230069257	0.7647018742	0.1116464109	0.268614925351
5852_FKS2	CG_UHB_11	N	E	A	F	T	L	D	I	41.622568289	0.3158076542	0.2502661914	0.231487375204
2362_FKS2	CG_UHB_12	N	E	A	F	P	L	D	I	46.697201766	0.862347048	0.0980061073	0.276120791094
7805_FKS2	CG_UHB_13	N	D	A	F	P	L	D	I	4.1825530175	2.9496066212	0.1592401497	0.254940462661
1008_2_FKS2	CG_UHB_14	N	D	A	F	P	L	D	I	2.3460137588	0.2572101048	0.1148310572	0.255128601536
6769_FKS2	CG_UHB_15	N	E	A	F	T	L	D	I	30.722437937	2.8454505973	0.1484817795	0.330173641052
S07_FKS2	CG_UHB_16	N	E	A	F	T	L	D	I	50.601449711	0.6201736152	0.131398177	0.519292978593
S04_FKS2	CG_UHB_17	N	E	A	F	T	L	D	I	27.805382103	0.8933812651	0.1641645327	0.196297425094
S01_FKS2	CG_UHB_18	N	E	A	F	T	L	D	I	25.877517864	0.0278081202	0.3612798088	0.226123850101
S14_FKS2	CG_UHB_19	N	E	A	F	P	L	D	I	46.792660356	8	0.1430666604	0.551207404981
S16_FKS2	CG_UHB_20	N	E	A	F	P	L	D	I	46.792660356	1.8073399474	0.1473993519	0.254041886441
S09_FKS2	CG_UHB_21	N	E	A	F	T	L	D	I	1.0081444478	0.1090642362	0.1316574475	0.229206981372
S11_FKS2	CG_UHB_22	N	D	A	F	P	L	D	I	9.5958707786	0.0645033266	0.2462582915	0.540216751762
S13_FKS2	CG_UHB_24	N	E	A	F	P	F	D	I	110.20258619	1.0435264917	0.1844128702	0.220850648581
S05_FKS2	CG_UHB_25	N	E	A	F	P	L	E	I	1.9977305272	0.1272006695	0.135539175	0.630044611458
S03_FKS2	CG_UHB_26	N	E	A	F	P	L	E	I	43.577880403	3.6122595201	0.1893451974	0.183280622211
S02_FKS2	CG_UHB_27	N	E	A	F	T	L	D	I	0.7108136564	0.0579915138	0.1512232029	0.278898079923
S12_FKS2	CG_UHB_27	N	E	A	F	T	L	D	I	12.430878476	8	0.2108859976	0.201750584959
S08_FKS2	CG_UHB_28	N	E	V	F	P	L	D	I	30.283985743	3.1878757748	0.0994995657	0.639822196409
S10_FKS2	CG_UHB_29	N	E	A	F	T	L	D	M	2.2987638742	0.7853533034	0.0898027763	0.272408238885
S15_FKS2	CG_UHB_30	N	E	A	F	T	L	D	I	3.8802347424	0.0156744012	0.1747224101	0.168898845789
REF-CBS138|FKS2		N	E	A	F	T	L	D	I				

FKS3_Aa_variations. Aminoacid variants in the FKS3 gene found in at least one of the 30 strains sequenced over this study.

		3	40	42	89	108	110	187	206	210	212	271	280	429	645	751	826	829	844	851	854	864	865	884	979	1039	1301	1472	1512	1676	1686	1731	1764	1768	1825	1840	Fluconazole (S<0.001; R>16)[3]	Voriconazole (ECOFF=1)[4]	Caspofungin (S≤0.125, R>0.5)[2]	Flucytosine (S≤0.25)[1]
6687_FKS3	CG_UHB_01	I	E	V	I	K	I	K	D	S	M	G	M	V	E	D	A	D	S	Q	N	L	N	R	A	N	R	I	L	A	S	Y	V	F	N	S	24.992826459	0.0292335204	0.2442450412	0.33820656581264
5877_FKS3	CG_UHB_02	I	E	A	I	K	I	K	D	S	M	G	M	V	E	D	A	D	S	Q	N	L	N	R	A	N	R	I	L	A	T	Y	V	F	N	S	75.256762069	10.0175324564	0.1428591927	0.46330719432754
3165_FKS3	CG_UHB_03	I	E	A	I	K	I	K	D	S	M	G	M	V	E	D	A	D	S	Q	N	L	N	R	T	N	R	I	L	A	T	Y	V	F	N	S	6.485379354	5.0361687161	0.1750437573	0.22224432276299
6958_FKS3	CG_UHB_04	T	E	A	I	K	I	K	D	S	M	G	M	V	E	D	A	D	S	Q	N	L	N	R	A	N	Q	V	L	A	T	C	V	F	N	Y	28.038581483	2.3531077481	1.9226507595	0.23017170233811
5916_FKS3	CG_UHB_05	T	E	A	I	K	I	K	D	S	M	G	M	V	E	D	A	D	S	Q	N	L	K	R	A	N	Q	V	L	A	T	C	V	F	N	Y	0.0175335349	0.0771340654	0.1112586026	0.31560959378105
3380_FKS3	CG_UHB_06	I	E	V	I	K	I	K	D	S	M	G	M	V	E	D	A	D	S	Q	N	L	N	R	A	N	R	I	L	A	S	Y	V	F	N	S	21.513628533	0.8285869773	0.1465884888	0.51479943004991
7051_FKS3	CG_UHB_07	T	E	A	I	K	I	K	D	S	M	G	M	V	E	D	A	D	S	Q	N	L	N	R	A	N	Q	V	L	T	T	C	V	F	N	Y	53.382429003	1.7163482074	0.1078147722	0.24878925909414
2821_FKS3	CG_UHB_08	T	E	G	I	K	I	E	D	S	M	G	M	V	E	D	A	D	S	Q	S	L	N	R	A	N	Q	I	L	A	T	Y	V	I	N	S	1.4084005883	1.9798090513	0.1362509252	0.35758070027615
9296_FKS3	CG_UHB_09	I	E	A	I	K	I	K	D	S	M	G	M	I	K	D	A	D	S	Q	N	L	N	R	A	N	R	I	M	A	T	Y	A	F	N	S	33.097918309	0.0024313751	0.1398946985	0.25480056698541
2390_FKS3	CG_UHB_10	I	E	A	I	K	I	K	D	S	M	G	M	V	E	D	A	D	S	Q	N	L	N	R	A	N	R	I	L	A	T	Y	V	F	N	S	44.230069257	0.7647018742	0.1116464109	0.26861492535113
5852_FKS3	CG_UHB_11	I	E	A	I	K	I	K	D	S	M	G	M	V	E	D	A	D	S	Q	N	L	N	L	A	N	R	I	L	A	T	Y	V	F	S	S	41.622568289	0.3158076542	0.2502661914	0.23148737520416
2362_FKS3	CG_UHB_12	T	E	A	I	K	I	K	D	S	M	G	M	V	E	E	A	D	S	Q	N	V	N	R	A	Y	Q	V	L	A	T	C	V	F	N	S	46.697201766	0.862347048	0.0980061073	0.27612079109378
7805_FKS3	CG_UHB_13	T	E	A	I	K	I	K	D	S	M	G	M	V	E	D	T	D	S	Q	N	L	N	R	A	N	Q	V	L	A	T	C	V	F	N	Y	4.1825530175	2.9496066212	0.1592401497	0.25494046266135
1008_2_FKS3	CG_UHB_14	T	E	A	I	K	I	K	D	S	I	G	M	V	E	D	A	D	S	Q	N	L	N	R	A	N	Q	V	L	A	T	C	V	F	N	Y	2.3460137588	0.2572101048	0.1148310572	0.25512860153569
6769_FKS3	CG_UHB_15	I	E	A	I	N	L	K	N	N	M	G	M	V	E	D	A	D	S	R	N	L	N	R	A	N	R	I	L	A	S	Y	V	F	N	S	30.722437937	2.8454505973	0.1484817795	0.33017364105177
S07_FKS3	CG_UHB_16	T	E	G	I	K	I	E	D	S	M	G	M	V	E	D	A	D	S	Q	S	L	N	R	A	N	Q	I	L	A	T	Y	V	I	N	S	50.601449711	0.6201736152	0.131398177	0.51929297859304
S04_FKS3	CG_UHB_17	T	E	G	I	K	I	E	D	S	M	G	M	V	E	D	A	D	S	Q	S	L	N	R	A	N	Q	I	L	A	T	Y	V	I	N	S	27.805362103	0.8933812651	0.1641645327	0.1962974250942
S01_FKS3	CG_UHB_18	T	E	A	I	K	I	K	D	S	M	G	M	V	E	D	A	D	S	Q	N	L	N	R	A	N	Q	V	L	T	T	C	V	F	N	Y	25.877517864	0.0278081202	0.3612798088	0.22612385010122
S14_FKS3	CG_UHB_19	I	E	V	I	K	I	K	D	S	M	G	M	V	E	D	A	D	S	Q	N	L	N	R	A	N	R	I	L	A	S	Y	V	F	N	S	46.792660356	8	0.1430666604	0.55120740498096
S16_FKS3	CG_UHB_20	I	E	V	I	K	I	K	D	S	M	G	M	V	E	D	A	D	S	Q	N	L	N	R	A	N	R	I	L	A	S	Y	V	F	N	S	46.792660356	1.8073399474	0.1473993519	0.25404188644058
S09_FKS3	CG_UHB_21	I	E	A	I	K	I	K	D	S	M	G	M	V	E	D	A	D	S	Q	N	L	N	R	A	N	R	I	L	A	S	Y	V	F	N	S	1.0081444478	0.1090642362	0.1316574475	0.22920698137162
S11_FKS3	CG_UHB_22	T	E	A	I	K	I	K	D	S	M	G	M	V	E	D	A	D	S	Q	N	L	N	R	A	N	Q	V	L	A	T	C	V	F	N	Y	9.5958707786	0.0645033266	0.2462582915	0.54021675176234
S13_FKS3	CG_UHB_24	I	E	V	I	K	I	K	D	S	M	G	M	V	E	D	A	D	S	Q	N	L	N	R	A	N	R	I	L	A	S	Y	V	F	N	S	110.20258619	1.0435264917	0.1844128702	0.22085064858132
S05_FKS3	CG_UHB_25	I	E	V	I	K	I	K	D	S	M	G	M	V	E	D	A	D	S	Q	N	L	N	R	A	N	R	I	L	A	S	Y	V	F	N	S	1.9977305272	0.1272006695	0.135539175	0.6300446114581
S03_FKS3	CG_UHB_26	I	E	V	I	K	I	K	D	S	M	G	M	V	E	D	A	D	S	Q	N	L	N	R	A	N	R	I	L	A	S	Y	V	F	N	S	43.577880403	3.6122595201	0.1893451974	0.18328062221057
S02_FKS3	CG_UHB_27	T	E	G	I	K	I	E	D	S	M	G	M	V	E	D	A	D	S	Q	S	L	N	R	A	N	Q	I	L	A	T	Y	V	I	N	S	0.7108136564	0.0579915138	0.1512232029	0.27889807992321
S12_FKS3	CG_UHB_27	I	E	A	I	K	I	K	D	S	M	G	M	V	E	D	A	D	S	Q	N	L	N	R	A	N	R	I	L	A	T	Y	V	F	N	S	12.430878476	8	0.2108859976	0.20175058495854
S08_FKS3	CG_UHB_28	I	E	V	I	K	I	K	D	S	M	G	M	V	E	D	A	D	S	Q	N	L	N	R	A	N	R	I	L	A	S	Y	V	F	N	S	30.283985743	3.1878757748	0.0994995657	0.63982219640928
S10_FKS3	CG_UHB_29	T	V	A	V	N	L	K	D	S	M	E	I	V	E	D	A	H	R	Q	N	L	N	R	A	N	R	V	L	A	T	C	V	F	N	S	2.2987638742	0.7853533034	0.0898027763	0.27240623888854
S15_FKS3	CG_UHB_30	T	E	A	I	K	I	K	D	S	M	G	M	V	E	D	A	D	S	Q	N	L	N	R	A	N	Q	V	L	T	T	C	V	F	N	Y	3.8802347424	0.0156744012	0.1747224101	0.16889884578903
REF-CBS138|FKS3		I	E	A	I	K	I	K	D	S	M	G	M	V	E	D	A	D	S	Q	N	L	N	R	A	N	R	I	L	A	T	Y	V	F	N	S				

FLR1_Aa_variations. Aminoacid variants in the FLR1 gene found in at least one of the 30 strains sequenced over this study.

		50	103	116	186	254	326	332	354	496	Fluconazole (S<0.001; R>16)[3]	Voriconazole (ECOFF=1)[4]	Caspofungin (S≤0.125, R>0.5)[2]	Flucytosine (S≤0.25)[1]
6687 FLR1 reverse complement	CG_UHB_01	S	I	V	I	V	Q	F	N	R	24.992826459	0.0292335204	0.2442450412	0.338206565813
5877 FLR1 reverse complement	CG_UHB_02	S	M	I	I	V	Q	F	D	R	75.256762069	10.0175324564	0.1428591927	0.463307194328
3165 FLR1 reverse complement	CG_UHB_03	S	I	V	I	V	Q	F	D	R	6.485379354	5.0361687181	0.1750437573	0.222244322763
6958 FLR1 reverse complement	CG_UHB_04	S	M	I	I	V	Q	F	D	R	28.038581483	2.3531077481	1.9226507595	0.230171702338
5916 FLR1 reverse complement	CG_UHB_05	S	M	I	I	I	Q	F	D	R	0.0175335349	0.0771340654	0.1112586026	0.315609593781
3380 FLR1 reverse complement	CG_UHB_06	S	I	V	I	V	Q	F	D	R	21.513628533	0.8285869773	0.1465884888	0.51479943005
7051 FLR1 reverse complement	CG_UHB_07	-	M	I	I	V	Q	F	D	R	53.382429003	1.7163482074	0.1078147722	0.248789259094
2821 FLR1 reverse complement	CG_UHB_08	S	I	V	V	V	Q	F	D	R	1.4084005883	1.9798090513	0.1362509252	0.357580700276
9296 FLR1 reverse complement	CG_UHB_09	S	M	I	I	L	Q	F	D	*	33.097918309	0.0024313751	0.1398946985	0.254800566985
2390 FLR1 reverse complement	CG_UHB_10	S	M	I	I	V	Q	F	D	R	44.230069257	0.7647018742	0.1116464109	0.268614925351
5852 FLR1 reverse complement	CG_UHB_11	S	M	I	I	V	Q	F	D	R	41.622568289	0.3158076542	0.2502661914	0.231487375204
2362 FLR1 reverse complement	CG_UHB_12	S	I	V	I	V	Q	F	D	R	46.697201766	0.862347048	0.0980061073	0.276120791094
7805 FLR1 reverse complement	CG_UHB_13	S	M	I	I	V	Q	F	D	R	4.1825530175	2.9496066212	0.1592401497	0.254940462661
1008 2 FLR1 reverse complement	CG_UHB_14	S	M	I	I	V	Q	F	D	R	2.3460137588	0.2572101048	0.1148310572	0.255128601536
6769 FLR1 reverse complement	CG_UHB_15	S	M	I	I	V	Q	F	D	R	30.722437937	2.8454505973	0.1484817795	0.330173641052
S07 FLR1 reverse complement	CG_UHB_16	S	M	I	I	I	Q	F	D	R	50.601449711	0.6201736152	0.131398177	0.519292978593
S04 FLR1 reverse complement	CG_UHB_17	S	M	I	I	I	Q	F	D	R	27.805362103	0.8933812651	0.1641645327	0.196297425094
S01 FLR1 reverse complement	CG_UHB_18	-	M	I	I	V	Q	F	D	R	25.877517864	0.0278081202	0.3612798088	0.226123850101
S14 FLR1 reverse complement	CG_UHB_19	S	I	V	I	V	Q	F	D	R	46.792660356	8	0.1430666604	0.551207404981
S16 FLR1 reverse complement	CG_UHB_20	S	I	V	I	V	Q	F	D	R	46.792660356	1.8073399474	0.1473993519	0.254041886441
S09 FLR1 reverse complement	CG_UHB_21	S	I	V	I	V	L	F	D	R	1.0081444478	0.1090642362	0.1316574475	0.229206981372
S11 FLR1 reverse complement	CG_UHB_22	S	M	I	I	V	Q	V	D	R	9.5958707786	0.0645033266	0.2462582915	0.540216751762
S13 FLR1 reverse complement	CG_UHB_24	S	I	V	I	V	Q	F	D	R	110.20258619	1.0435264917	0.1844128702	0.220850648581
S05 FLR1 reverse complement	CG_UHB_25	S	I	V	I	V	Q	F	D	R	1.9977305272	0.1272006695	0.135539175	0.630044611458
S03 FLR1 reverse complement	CG_UHB_26	S	I	V	I	V	Q	F	D	R	43.577880403	3.6122595201	0.1893451974	0.183280622211
S02 FLR1 reverse complement	CG_UHB_27	S	M	I	I	I	Q	F	D	R	0.7108136564	0.0579915138	0.1512232029	0.278898079923
S12 FLR1 reverse complement	CG_UHB_27	S	M	I	I	I	Q	F	D	R	12.430878476	8	0.2108859976	0.201750584959
S08 FLR1 reverse complement	CG_UHB_28	S	I	V	I	V	Q	F	D	R	30.283985743	3.1878757748	0.0994995657	0.639822196409
S10 FLR1 reverse complement	CG_UHB_29	S	I	V	I	V	Q	F	D	R	2.2987638742	0.7853533034	0.0898027763	0.272406238885
S15 FLR1 reverse complement	CG_UHB_30	-	M	I	I	V	Q	F	D	R	3.8802347424	0.0156744012	0.1747224101	0.168898845789

FPS1_Aa_variations. Aminoacid variants in the FPS1 gene found in at least one of the 30 strains sequenced over this study.

		316	Fluconazole (S<0.001; R>16)[3]	Voriconazole (ECOFF=1)[4]	Caspofungin (S≤0.125, R>0.5)[2]	Flucytosine (S≤0.25)[1]
6687	CG_UHB_01	A	24.992826459	0.0292335204	0.2442450412	0.3382065658126
5877	CG_UHB_02	A	75.256762069	10.0175324564	0.1428591927	0.4633071943275
3165	CG_UHB_03	A	6.485379354	5.0361687161	0.1750437573	0.222244322763
6958	CG_UHB_04	A	28.038581483	2.3531077481	1.9226507595	0.2301717023381
5916	CG_UHB_05	S	0.0175335349	0.0771340654	0.1112586026	0.3156095937811
3380	CG_UHB_06	A	21.513628533	0.8285869773	0.1465884888	0.5147994300499
7051	CG_UHB_07	S	53.382429003	1.7163482074	0.1078147722	0.2487892590941
2821	CG_UHB_08	A	1.4084005883	1.9798090513	0.1362509252	0.3575807002762
9296	CG_UHB_09	S	33.097918309	0.0024313751	0.1398946985	0.2548005669854
2390	CG_UHB_10	A	44.230069257	0.7647018742	0.1116464109	0.2686149253511
5852	CG_UHB_11	A	41.622568289	0.3158076542	0.2502661914	0.2314873752042
2362	CG_UHB_12	A	46.697201766	0.862347048	0.0980061073	0.2761207910938
7805	CG_UHB_13	A	4.1825530175	2.9496066212	0.1592401497	0.2549404626613
1008_2	CG_UHB_14	A	2.3460137588	0.2572101048	0.1148310572	0.2551286015357
6769	CG_UHB_15	A	30.722437937	2.8454505973	0.1484817795	0.3301736410518
S07	CG_UHB_16	S	50.601449711	0.6201736152	0.131398177	0.519292978593
S04	CG_UHB_17	S	27.805362103	0.8933812651	0.1641645327	0.1962974250942
S01	CG_UHB_18	S	25.877517864	0.0278081202	0.3612798088	0.2261238501012
S14	CG_UHB_19	A	46.792660356	8	0.1430666604	0.551207404981
S16	CG_UHB_20	A	46.792660356	1.8073399474	0.1473993519	0.2540418864406
S09	CG_UHB_21	A	1.0081444478	0.1090642362	0.1316574475	0.2292069813716
S11	CG_UHB_22	A	9.5958707786	0.0645033266	0.2462582915	0.5402167517623
S13	CG_UHB_24	A	110.20258619	1.0435264917	0.1844128702	0.2208506485813
S05	CG_UHB_25	A	1.9977305272	0.1272006695	0.135539175	0.6300446114581
S03	CG_UHB_26	A	43.577880403	3.6122595201	0.1893451974	0.1832806222106
S02	CG_UHB_27	S	0.7108136564	0.0579915138	0.1512232029	0.2788980799232
S12	CG_UHB_27	A	12.430878476	8	0.2108859976	0.2017505849585
S08	CG_UHB_28	A	30.283985743	3.1878757748	0.0994995657	0.6398221964093
S10	CG_UHB_29	A	2.2987638742	0.7853533034	0.0898027763	0.2724062388854
S15	CG_UHB_30	S	3.8802347424	0.0156744012	0.1747224101	0.168898845789
REF-CBS138		A				

FPS2_Aa_variations. Aminoacid variants in the FPS2 gene found in at least one of the 30 strains sequenced over this study.

		17	85	128	227	228	409	474	511	517	Fluconazole (S<0.001; R>16)[3]	Voriconazole (ECOFF=1)[4]	Caspofungin (S≤0.125, R>0.5)[2]	Flucytosine (S≤0.25)[1]
6687	CG_UHB_01	D	A	N	A	V	T	R	D	Y	24.992826459	0.0292335204	0.2442450412	0.3382065658126
5877	CG_UHB_02	N	A	H	A	V	A	K	D	D	75.256762069	10.0175324564	0.1428591927	0.4633071943275
3165	CG_UHB_03	N	A	H	A	V	A	K	D	D	6.485379354	5.0361687161	0.1750437573	0.222244322763
6958	CG_UHB_04	N	A	H	A	V	A	K	D	D	28.038581483	2.3531077481	1.9226507595	0.2301717023381
5916	CG_UHB_05	N	A	H	A	V	A	K	D	D	0.0175335349	0.0771340654	0.1112586026	0.3156095937811
3380	CG_UHB_06	D	A	Y	A	V	T	R	D	Y	21.513628533	0.8285869773	0.1465884888	0.5147994300499
7051	CG_UHB_07	N	A	H	A	V	A	K	D	D	53.382429003	1.7163482074	0.1078147722	0.2487892590941
2821	CG_UHB_08	D	A	H	S	V	T	R	D	D	1.4084005883	1.9798090513	0.1362509252	0.3575807002762
9296	CG_UHB_09	N	A	H	A	V	A	K	D	D	33.097918309	0.0024313751	0.1398946985	0.2548005869854
2390	CG_UHB_10	N	A	H	A	V	A	K	D	D	44.230069257	0.7647018742	0.1116464109	0.2686149253511
5852	CG_UHB_11	N	A	H	A	V	A	K	E	D	41.622568289	0.3158076542	0.2502661914	0.2314873752042
2362	CG_UHB_12	D	V	N	S	V	T	R	D	D	46.697201766	0.862347048	0.0980061073	0.2761207910938
7805	CG_UHB_13	N	A	H	A	V	A	K	D	D	4.1825530175	2.9496066212	0.1592401497	0.2549404626613
1008_2	CG_UHB_14	N	A	H	A	V	A	K	D	D	2.3460137588	0.2572101048	0.1148310572	0.2551286015357
6769	CG_UHB_15	N	A	H	A	V	A	K	D	D	30.722437937	2.8454505973	0.1484817795	0.3301736410518
S07	CG_UHB_16	N	A	H	A	V	A	K	D	D	50.601449711	0.6201736152	0.131398177	0.519292978593
S04	CG_UHB_17	N	A	H	A	V	A	K	D	D	27.805362103	0.8933812651	0.1641645327	0.1962974250942
S01	CG_UHB_18	N	A	H	A	V	A	K	D	D	25.877517864	0.0278081202	0.3612798088	0.2261238501012
S14	CG_UHB_19	D	A	N	A	V	T	R	D	Y	46.792660356	8	0.1430666604	0.551207404981
S16	CG_UHB_20	D	A	N	A	V	T	R	D	Y	46.792660356	1.8073399474	0.1473993519	0.2540418864406
S09	CG_UHB_21	N	A	H	A	V	A	K	D	D	1.0081444478	0.1090642362	0.1316574475	0.2292069813716
S11	CG_UHB_22	N	A	H	A	V	A	K	D	D	9.5958707786	0.0645033266	0.2462582915	0.5402167517623
S13	CG_UHB_24	D	A	N	A	V	T	R	D	Y	110.20258619	1.0435264917	0.1844128702	0.2208506485813
S05	CG_UHB_25	D	A	N	A	V	T	R	D	Y	1.9977305272	0.1272006695	0.135539175	0.6300446114581
S03	CG_UHB_26	D	A	N	A	V	T	R	D	Y	43.577880403	3.6122595201	0.1893451974	0.1832806222106
S02	CG_UHB_27	N	A	H	A	V	A	K	D	D	0.7108136564	0.0579915138	0.1512232029	0.2788980799232
S12	CG_UHB_27	N	A	H	A	V	A	R	D	D	12.430878476	8	0.2108859976	0.2017505849585
S08	CG_UHB_28	D	A	N	A	V	T	R	D	Y	30.283985743	3.1878757748	0.0994995657	0.6398221964093
S10	CG_UHB_29	D	A	H	S	V	T	R	D	D	2.2987638742	0.7853533034	0.0898027763	0.2724062388854
S15	CG_UHB_30	N	A	H	A	I	A	K	D	D	3.8802347424	0.0156744012	0.1747224101	0.168898845789
REF-CBS138		N	A	H	A	V	A	K	D	D				

PDR1_Aa_variations. Aminoacid variants in the PDR1 gene found in at least one of the 30 strains sequenced over this study.

		75	91	98	134	143	189	243	255	307	308	337	343	345	461	540	745	935	939	1090	Fluconazole (S<0.001; R>16)[3]	Voriconazole (ECOFF=1)[4]	Caspofungin (S≤0.125, R>0.5)[2]	Flucytosine (S≤0.25)[1]
6687	CG_UHB_01	S	I	S	V	T	G	N	N	N	H	Q	S	Q	M	M	T	L	I	L	24.992826459	0.0292335204	0.2442450412	0.338206565813
5877	CG_UHB_02	P	I	S	V	P	G	D	N	N	H	Q	F	Q	M	M	T	L	I	L	75.256762069	10.0175324564	0.1428591927	0.463307194328
3165	CG_UHB_03	P	I	S	V	P	V	D	N	N	H	Q	S	Q	M	M	T	L	I	L	6.485379354	5.0361687161	0.1750437573	0.222244322763
6958	CG_UHB_04	P	I	S	V	P	G	D	N	N	H	Q	S	Q	M	M	T	S	I	L	28.038581483	2.3531077481	1.9226507595	0.230171702338
5916	CG_UHB_05	P	I	S	V	P	G	D	N	N	H	Q	S	Q	I	M	T	L	I	L	0.0175335349	0.0771340654	0.1112586026	0.315609593781
3380	CG_UHB_06	S	I	S	V	T	G	N	N	N	H	Q	S	Q	M	M	T	L	I	L	21.513628533	0.8285869773	0.1465884888	0.51479943005
7051	CG_UHB_07	P	I	S	V	P	G	D	N	N	H	Q	S	Q	M	I	T	L	I	L	53.382429003	1.7163482074	0.1078147722	0.248789259094
2821	CG_UHB_08	P	I	S	V	P	G	D	N	N	H	Q	S	Q	M	M	T	L	I	L	1.4084005883	1.9798090513	0.1362509252	0.357580700278
9296	CG_UHB_09	P	I	S	V	P	G	D	N	N	H	Q	S	Q	M	M	T	L	I	L	33.097918309	0.0024313751	0.1398946985	0.254800566985
2390	CG_UHB_10	P	I	S	V	P	G	D	N	N	H	Q	S	Q	M	M	-	-	-	-	44.230069257	0.7647018742	0.1116464109	0.268614925351
5852	CG_UHB_11	P	I	S	V	P	G	D	N	N	H	Q	S	Q	M	M	T	L	I	L	41.622568289	0.3158076542	0.2502661914	0.231487375204
2362	CG_UHB_12	P	I	S	V	P	G	D	N	N	H	Q	S	Q	M	M	A	L	I	L	46.697201766	0.862347048	0.0980061073	0.276120791094
7805	CG_UHB_13	P	I	S	V	P	G	D	N	N	H	Q	S	Q	M	M	T	L	I	L	4.1825530175	2.9496066212	0.1592401497	0.254940462661
1008_2	CG_UHB_14	P	I	S	V	P	G	D	N	N	H	Q	S	Q	M	M	T	L	I	L	2.3460137588	0.2572101048	0.1148310572	0.255128601536
6769	CG_UHB_15	P	I	S	V	P	G	D	N	N	H	Q	S	K	M	M	T	L	I	L	30.722437937	2.8454505973	0.1484817795	0.330173641052
S07	CG_UHB_16	P	I	S	V	P	G	D	N	N	H	Q	S	Q	M	M	T	L	I	L	50.601449711	0.6201736152	0.131398177	0.519292978593
S04	CG_UHB_17	P	I	S	V	P	G	D	N	N	H	Q	S	Q	M	M	T	L	I	L	27.805362103	0.8933812651	0.1641645327	0.196297425094
S01	CG_UHB_18	P	I	S	V	P	G	D	N	K	Y	Q	S	Q	M	M	T	L	V	L	25.877517864	0.0278081202	0.3612798088	0.226123850101
S14	CG_UHB_19	S	I	S	V	T	G	N	N	N	H	Q	S	Q	M	M	T	L	I	L	46.792660356	8	0.1430666604	0.551207404981
S16	CG_UHB_20	S	I	S	V	T	G	N	N	N	H	Q	S	Q	M	M	T	L	I	L	46.792660356	1.8073399474	0.1473993519	0.254041886441
S09	CG_UHB_21	S	I	S	A	T	G	D	N	N	H	Q	S	Q	M	M	T	L	I	L	1.0081444478	0.1090642362	0.1316574475	0.229206981372
S11	CG_UHB_22	P	I	S	V	P	G	D	N	N	H	Q	S	Q	M	M	T	L	I	L	9.5958707786	0.0645033266	0.2462582915	0.540216751762
S13	CG_UHB_24	S	I	S	V	T	G	N	N	N	H	Q	S	Q	M	M	T	L	I	L	110.20258619	1.0435264917	0.1844128702	0.220850648581
S05	CG_UHB_25	S	I	S	V	T	G	N	N	N	H	Q	S	Q	M	M	T	L	I	S	1.9977305272	0.1272006695	0.135539175	0.630044611458
S03	CG_UHB_26	S	I	S	V	T	G	N	N	N	H	Q	S	Q	M	M	T	L	I	L	43.577880403	3.6122595201	0.1893451974	0.183280622211
S02	CG_UHB_27	P	I	S	V	P	G	D	N	N	H	Q	S	Q	M	M	T	L	I	L	0.7108136564	0.0579915138	0.1512232029	0.278898079923
S12	CG_UHB_27	P	I	S	V	P	G	D	N	N	H	Q	S	Q	M	M	T	L	I	L	12.430878476	8	0.2108859976	0.201750584959
S08	CG_UHB_28	S	I	S	V	T	G	N	N	N	H	R	S	Q	M	M	T	L	I	L	30.283985743	3.1878757748	0.0994995657	0.639822196409
S10	CG_UHB_29	P	I	S	V	P	G	D	K	N	H	Q	S	Q	M	M	A	L	I	L	2.2987638742	0.7853533034	0.0898027763	0.272406238885
S15	CG_UHB_30	P	I	S	V	P	G	D	N	N	H	Q	S	Q	M	M	T	L	I	L	3.8802347424	0.0156744012	0.1747224101	0.168898845789
REF-CBS138		S	V	L	V	T	G	D	N	N	H	Q	S	Q	M	M	T	L	I	L				

SNQ2_Aa_variations. Aminoacid variants in the SNQ2 gene found in at least one of the 30 strains sequenced over this study.

		433	633	634	635	636	637	638	639	700	703	1000	1005	1085	1147	1156	1506	Fluconazole ($S<0.001$; $R>16$)[3]	Voriconazole (ECOFF=1)[4]	Caspofungin ($S\leq0.125$, $R>0.5$)[2]	Flucytosine ($S\leq0.25$)[1]
6687_SNQ2_reverse_complement	CG_UHB_01	R	F	R	V	Y	L	F	L	I	A	E	L	D	H	K	S	24.992826459	0.0292335204	0.2442450412	0.33820656581
5877_SNQ2_reverse_complement	CG_UHB_02	R	F	R	V	Y	L	F	L	I	A	E	L	D	H	K	S	75.256762069	10.0175324564	0.1428591927	0.46330719433
3165_SNQ2_reverse_complement	CG_UHB_03	R	F	R	V	Y	L	F	L	I	A	E	L	N	H	K	S	6.485379354	5.0361687161	0.1750437573	0.22224432276
6958_SNQ2_reverse_complement	CG_UHB_04	R	S	E	Y	T	S	S	*	-	-	-	-	-	-	-	-	26.038581483	2.3531077481	1.9226507595	0.23017170234
5916_SNQ2_reverse_complement	CG_UHB_05	R	F	R	V	Y	L	F	L	I	A	E	L	D	H	K	S	0.0175335349	0.0771340654	0.1112586026	0.31560959378
3380_SNQ2_reverse_complement	CG_UHB_06	R	F	R	V	Y	L	F	L	I	A	E	L	D	H	K	S	21.513628533	0.8285869773	0.1465884888	0.51479943005
7051_SNQ2_reverse_complement	CG_UHB_07	R	F	R	V	Y	L	F	L	I	A	E	S	D	H	K	S	53.382429003	1.7163482074	0.1078147722	0.24878925909
2821_SNQ2_reverse_complement	CG_UHB_08	R	F	R	V	Y	L	F	L	I	A	E	L	D	H	K	S	1.4084005883	1.9798090513	0.1362509252	0.35758070028
9296_SNQ2_reverse_complement	CG_UHB_09	R	F	R	V	Y	L	F	L	I	A	E	L	D	H	K	S	33.097918309	0.0024313751	0.1398946985	0.25480056699
2390_SNQ2_reverse_complement	CG_UHB_10	R	F	R	V	Y	L	F	L	I	A	E	L	D	H	K	S	44.230069257	0.7647018742	0.1116464109	0.26861492535
5852_SNQ2_reverse_complement	CG_UHB_11	R	F	R	V	Y	L	F	L	I	A	E	L	D	H	K	S	41.622568289	0.3158076542	0.2502661914	0.2314873752
2362_SNQ2_reverse_complement	CG_UHB_12	R	F	R	V	Y	L	F	L	I	A	E	L	D	H	K	S	46.697201766	0.862347048	0.0980061073	0.27612079109
7805_SNQ2_reverse_complement	CG_UHB_13	R	F	R	V	Y	L	F	L	I	A	E	L	D	H	K	S	4.1825530175	2.9496066212	0.1592401497	0.25494046266
1008_2_SNQ2_reverse_complement	CG_UHB_14	R	F	R	V	Y	L	F	L	I	A	D	L	D	H	K	S	2.3460137588	0.2572101048	0.1148310572	0.25512860154
6769_SNQ2_reverse_complement	CG_UHB_15	R	F	R	V	Y	L	F	L	I	A	E	L	D	H	K	P	30.722437937	2.8454505973	0.1484817795	0.33017364105
S07_SNQ2_reverse_complement	CG_UHB_16	R	F	R	V	Y	L	F	L	I	A	E	L	D	H	K	S	50.601449711	0.6201736152	0.131398177	0.51929297859
S04_SNQ2_reverse_complement	CG_UHB_17	R	F	R	V	Y	L	F	L	I	A	E	L	D	H	K	S	27.805362103	0.8933812651	0.1641645327	0.19629742509
S01_SNQ2_reverse_complement	CG_UHB_18	S	F	R	V	Y	L	F	L	I	A	E	L	D	H	K	S	25.877517864	0.0278081202	0.3612798088	0.2261238501
S14_SNQ2_reverse_complement	CG_UHB_19	R	F	R	V	Y	L	F	L	I	A	E	L	D	H	K	S	46.792660356	8	0.1430666604	0.55120740498
S16_SNQ2_reverse_complement	CG_UHB_20	R	F	R	V	Y	L	F	L	I	A	E	L	D	H	K	S	46.792660356	1.8073399474	0.1473993519	0.25404188644
S09_SNQ2_reverse_complement	CG_UHB_21	R	F	R	V	Y	L	F	L	I	A	E	L	D	Q	Q	S	1.0081444478	0.1090642362	0.1316574475	0.22920698137
S11_SNQ2_reverse_complement	CG_UHB_22	R	F	R	V	Y	L	F	L	F	A	E	L	D	H	K	S	9.5958707786	0.0645033266	0.2462582915	0.54021675176
S13_SNQ2_reverse_complement	CG_UHB_24	R	F	R	V	Y	L	F	L	I	A	E	L	D	H	K	S	110.20258619	1.0435264917	0.1844128702	0.22085064858
S05_SNQ2_reverse_complement	CG_UHB_25	R	F	R	V	Y	L	F	L	I	A	E	L	D	H	K	S	1.9977305272	0.1272006695	0.135539175	0.63004461146
S03_SNQ2_reverse_complement	CG_UHB_26	R	F	R	V	Y	L	F	L	I	A	E	L	D	H	K	S	43.577880403	3.6122595201	0.1893451974	0.18328062221
S02_SNQ2_reverse_complement	CG_UHB_27	R	F	R	V	Y	L	F	L	I	A	E	L	D	H	K	S	0.7108136564	0.0579915138	0.1512232029	0.27889807992
S12_SNQ2_reverse_complement	CG_UHB_27	S	F	R	V	Y	L	F	L	I	A	E	L	D	H	K	S	12.430878476	8	0.2108859976	0.20175058496
S08_SNQ2_reverse_complement	CG_UHB_28	R	F	R	V	Y	L	F	L	I	G	E	L	D	H	K	S	30.283985743	3.1878757748	0.0994995657	0.63982219641
S10_SNQ2_reverse_complement	CG_UHB_29	R	F	R	V	Y	L	F	L	I	A	E	L	D	H	K	S	2.2987638742	0.7853533034	0.0898027763	0.27240623889
S15_SNQ2_reverse_complement	CG_UHB_30	R	F	R	V	Y	L	F	L	I	A	E	L	D	H	K	S	3.8802347424	0.0156744012	0.1747224101	0.16889884579
REF-CBS138|SNQ2	REF	R	F	R	V	Y	L	F	L	I	A	E	L	D	H	K	S				

Back to the index

Supplementary information

Supplementary Table 5

List of missense (non-synonymous) mutations found to be associated with the phenotype (resistance to antifungals).

Table S5

List of missense (non-synonymouos) mutations found to be associated with the phenotype (resistance to antifungals).

To navigate in the file, click on the underlined text.

Caspofungin: list of missense mutations found to be associated with the response to caspofungin. “antifungal”=name of the antifungal; “chromosome”= chromosome including the identified missense SNP; “strand”= strand of the coding region (W=Watson, C=Crick); “coordinate”= chromosome coordinate of the missense SNP; “Gene ID”= ID of the gene; “Entrez”= Entrez ID of the gene; “geneBankID”= Gene Bank ID of the gene; “nucleotide_alleles”= detail on the SNP, defined as the nucleotide in the reference genome, the coordinate of the gene (referred to the CDS sequence), and the nucleotide of the variant found to be associated with the phenotype; “aminoacid_alleles”= detail on the aminoacid change resulting from the mutation; “Description”= description of the gene (from the Candida Genome Database). Red text indicates missense SNPs in genes coding for adhesin or adhesin-like proteins or involved in the development of filamentous structures or biofilm.

caspofungin_annotation: GO terms and pathways annotations of the genes whose variants have been found to be associated with the response to caspofungin. “Gene ID”= ID of the gene; “Entrez”= Entrez ID of the gene; “KEGG_PATHWAY”= KEGG pathway encompassing the gene; “GOTERM_BP_DIRECT”= Biological Process GO term associated with the gene; “GO_TERM_CC_DIRECT”= Cellular Component GO term associated with the gene; “GO_TERM_MF_DIRECT”= Molecular Function GO term associated with the gene

fluconazole: list of missense mutations found to be associated with the response to fluconazole. “antifungal”=name of the antifungal; “chromosome”= chromosome including the identified missense SNP; “strand”= strand of the coding region (W=Watson, C=Crick); “coordinate”= chromosome coordinate of the missense SNP; “Gene ID”= ID of the gene; “Entrez”= Entrez ID of the gene; “geneBankID”= Gene Bank ID of the gene; “nucleotide_alleles”= detail on the SNP, defined as the nucleotide in the reference genome, the coordinate of the gene (referred to the CDS sequence), and the nucleotide of the variant found to be associated with the phenotype; “aminoacid_alleles”= detail on the aminoacid change resulting from the mutation; “Description”= description of the gene (from the Candida Genome Database). Red text indicates missense SNPs in genes coding for adhesin or adhesin-like proteins or involved in the development of filamentous structures or biofilm.

fluconazole_annotation: GO terms and pathways annotations of the genes whose variants have been found to be associated with the response to fluconazole. “Gene ID”= ID of the gene; “Entrez”= Entrez ID of the gene; “KEGG_PATHWAY”= KEGG pathway encompassing the gene; “GOTERM_BP_DIRECT”= Biological Process GO term associated with the gene; “GO_TERM_CC_DIRECT”= Cellular Component GO term associated with the gene; “GO_TERM_MF_DIRECT”= Molecular Function GO term associated with the gene.

Voriconazole: list of missense mutations found to be associated with the response to voriconazole. “antifungal”=name of the antifungal; “chromosome”= chromosome including the identified missense SNP; “strand”= strand of the coding region (W=Watson, C=Crick); “coordinate”= chromosome coordinate of the missense SNP; “Gene ID”= ID of the gene; “Entrez”= Entrez ID of the gene; “geneBankID”= Gene Bank ID of the gene; “nucleotide_alleles”= detail on the SNP, defined as the nucleotide in the reference genome, the coordinate of the gene (referred to the CDS sequence), and the nucleotide of the variant found to be associated with the phenotype; “aminoacid_alleles”= detail on the aminoacid change resulting from the mutation; “Description”= description of the gene (from the Candida Genome Database). Red text indicates missense SNPs in genes coding for adhesin or adhesin-like proteins or involved in the development of filamentous structures or biofilm.

voriconazole_annotation: GO terms and pathways annotations of the genes whose variants have been found to be associated with the response to voriconazole. “Gene ID”= ID of the gene;

“Entrez”= Entrez ID of the gene; “KEGG_PATHWAY”= KEGG pathway encompassing the gene; “GOTERM_BP_DIRECT”= Biological Process GO term associated with the gene; “GO_TERM_CC_DIRECT”= Cellular Component GO term associated with the gene; “GO_TERM_MF_DIRECT”= Molecular Function GO term associated with the gene.

Flucytosine: list of missense mutations found to be associated with the response to flucytosine. “antifungal”=name of the antifungal; “chromosome”= chromosome including the identified missense SNP; “strand”= strand of the coding region (W=Watson, C=Crick); “coordinate”= chromosome coordinate of the missense SNP; “Gene ID”= ID of the gene; “Entrez”= Entrez ID of the gene; “geneBankID”= Gene Bank ID of the gene; “nucleotide_alleles”= detail on the SNP, defined as the nucleotide in the reference genome, the coordinate of the gene (referred to the CDS sequence), and the nucleotide of the variant found to be associated with the phenotype; “aminoacid_alleles”= detail on the aminoacid change resulting from the mutation; “Description”= description of the gene (from the Candida Genome Database). Red text indicates missense SNPs in genes coding for adhesin or adhesin-like proteins or involved in the development of filamentous structures or biofilm.

flucytosine_annotation: GO terms and pathways annotations of the genes whose variants have been found to be associated with the response to flucytosine. “Gene ID”= ID of the gene; “Entrez”= Entrez ID of the gene; “KEGG_PATHWAY”= KEGG pathway encompassing the gene; “GOTERM_BP_DIRECT”= Biological Process GO term associated with the gene; “GO_TERM_CC_DIRECT”= Cellular Component GO term associated with the gene; “GO_TERM_MF_DIRECT”= Molecular Function GO term associated with the gene.

Enrichment: list of Gene Ontologies and pathways enriched in the genes whose variants have been found to be associated with the response to the tested antifungals output of GO and KEGG pathway enrichmen analysis carried out by using the David genomics web-tool.

Caspofungin

chromosome	strand	coordinate	Gene ID	Entrez		geneBankID	nucleotide_alleles	aminoacid_alleles	REF	ALLELE	Description
NC_006031.1	W	1046688	CAGL0H10626g	2888521	id2701	XM_447260.1	A2746G	Ile916Val	A	G	Putative adhesin-like protein; belongs to adhesin cluster I; appears artificially broken into fragments due to sequencing errors; predicted GPI anchor
NC_006031.1	W	1046690	CAGL0H10626g	2888521	id2701	XM_447260.1	C2748G	Ile916Met	C	G	Putative adhesin-like protein; belongs to adhesin cluster I; appears artificially broken into fragments due to sequencing errors; predicted GPI anchor
NC_005967.2	C	153426	CAGL0A01474g	2886376	id58	XM_444831.1	T272C	Ala91Val	C	T	Ortholog(s) have cell surface, extracellular region, fungal-type cell wall localization
NC_005967.2	C	153445	CAGL0A01474g	2886376	id58	XM_444831.1	T291A	Asp97Glu	T	A	Ortholog(s) have cell surface, extracellular region, fungal-type cell wall localization
NC_005967.2	C	230866	CAGL0A02211g	2886382	id94	XM_444860.1	G349A	Val117Ile	C	T	Ortholog(s) have glucose transmembrane transporter activity, pentose transmembrane transporter activity, role in glucose transmembrane transport and plasma membrane localization
NC_005968.1	W	170083	CAGL0B01859g	2886698	id301	XM_445050.1	G1108A	Asp370Asn	G	A	Ortholog(s) have ubiquitin-protein transferase activity, role in anaphase-promoting complex-dependent catabolic process, protein ubiquitination and anaphase-promoting complex, nuclear periphery localization
NC_006026.1	C	390017	CAGL0C03960g	2886882	id625	XM_445348.1	G1105A	Val369Ile	C	T	Has domain(s) with predicted transferase activity, transferring glycosyl groups activity and role in protein glycosylation
NC_006026.1	C	390018	CAGL0C03960g	2886882	id625	XM_445348.1	T1104G	Asp368Glu	A	C	Has domain(s) with predicted transferase activity, transferring glycosyl groups activity and role in protein glycosylation
NC_006026.1	C	390022	CAGL0C03960g	2886882	id625	XM_445348.1	C1100A	Thr367Asn	G	T	Has domain(s) with predicted transferase activity, transferring glycosyl groups activity and role in protein glycosylation
NC_006026.1	C	390028	CAGL0C03960g	2886882	id625	XM_445348.1	C1094T	Thr365Ile	G	A	Has domain(s) with predicted transferase activity, transferring glycosyl groups activity and role in protein glycosylation
NC_006028.2	C	13148	CAGL0E00187g	2887325	id1015	XM_445699.1	G80C	Gly27Ala	C	G	Putative adhesin-like protein; belongs to adhesin cluster IV
NC_006028.2	C	13166	CAGL0E00187g	2887325	id1015	XM_445699.1	G62C	Gly21Ala	G	C	Putative adhesin-like protein; belongs to adhesin cluster IV
NC_006028.2	C	13176	CAGL0E00187g	2887325	id1015	XM_445699.1	G52T	Ala18Ser	C	A	Putative adhesin-like protein; belongs to adhesin cluster IV
NC_006028.2	C	670480	CAGL0E06666g	2887561	id1311	XM_445980.1	A4213T	Ile1405Leu	T	A	Epithelial adhesion protein; predicted GPI-anchor; belongs to adhesin cluster I

NC_006028.2	C	673782	CAGL0E06666g	2887561	id1311	XM_445980.1	G911A	Gly304Glu	C	T	Epithelial adhesion protein; predicted GPI-anchor; belongs to adhesin cluster I
NC_006028.2	C	673783	CAGL0E06666g	2887561	id1311	XM_445980.1	G910A	Gly304Arg	C	T	Epithelial adhesion protein; predicted GPI-anchor; belongs to adhesin cluster I
NC_006028.2	C	674371	CAGL0E06666g	2887561	id1311	XM_445980.1	A322G	Lys108Glu	T	C	Epithelial adhesion protein; predicted GPI-anchor; belongs to adhesin cluster I
NC_006028.2	C	674391	CAGL0E06666g	2887561	id1311	XM_445980.1	T302A	Phe101Tyr	A	T	Epithelial adhesion protein; predicted GPI-anchor; belongs to adhesin cluster I
NC_006029.1	W	754153	CAGL0F07733g	2887615	id1657	XM_446304.1	G393A	Met131Ile	G	A	Ortholog(s) have ATP binding, ATPase activity, bubble DNA binding, dinucleotide insertion or deletion binding, heteroduplex DNA loop binding, single-stranded DNA binding activity
NC_006030.1	W	73814	CAGL0G00814g	2888154	id1760	XM_446397.1	T1364A	Val455Asp	T	A	Ortholog(s) have small GTPase binding activity
NC_006030.1	W	736970	CAGL0G07755g	2888422	id2098	XM_446699.1	G859A	Asp287Asn	G	A	Ortholog(s) have telomeric DNA binding activity, role in protein localization to chromosome, telomere maintenance via telomerase and nuclear chromosome, telomeric region localization
NC_006030.1	W	957190	CAGL0G09999g	2888156	id2203	XM_446795.1	A374C	Glu125Ala	A	C	Ortholog(s) have protein kinase regulator activity
NC_006031.1	C	257646	CAGL0H02805g	2888562	id2335	XM_446916.1	A2696G	Glu899Gly	T	C	Ortholog(s) have ATPase activity, DNA binding, cohesin ATPase activity, double-stranded DNA binding, protein kinase binding, topological DNA entrapment activity
NC_006031.1	W	338700	CAGL0H03663g	2888820	id2375	XM_446953.1	T1168G	Tyr390Asp	T	G	Ortholog(s) have isocitrate dehydrogenase (NADP+) activity, role in fatty acid beta-oxidation and peroxisomal importomer complex, peroxisome localization
NC_006031.1	W	487956	CAGL0H05071g	2888697	id2440	XM_447016.1	G1058A	Ser353Asn	G	A	Ortholog(s) have SUMO transferase activity, damaged DNA binding, double-stranded DNA-dependent ATPase activity, single-stranded DNA binding activity
NC_006032.2	W	305072	CAGL0I03586g	2889183	id2858	XM_447404.1	A1130G	Lys377Arg	A	G	Ortholog(s) have role in reciprocal meiotic recombination
NC_006032.2	W	679817	CAGL0I07051g	2889184	id3026	XM_447556.1	G538A	Gly180Ser	G	A	Ortholog(s) have cyclin-dependent protein serine/threonine kinase regulator activity, role in positive regulation of macroautophagy and cyclin-dependent protein kinase holoenzyme complex localization
NC_006032.2	W	970991	CAGL0I10147g	9488014	id3168	XM_002999517.1	G12751C	Ala4251Pro	G	C	Protein with 32 tandem repeats; putative adhesin-like protein; belongs to adhesin cluster II
NC_006033.2	W	247474	CAGL0J02508g	2889767	id3316	XM_447814.1	A1349T	Glu450Val	A	T	Adhesin-like protein; identified in cell wall extracts by mass spectrometry; belongs to adhesin cluster VI; predicted GPI anchor
NC_006033.	C	250846	CAGL0J	288976	id3317	XM_4478	C2048T	Ala683Val	G	A	Putative adhesion protein; predicted GPI-anchor; belongs to adhesin

2			02530g	8		15.1					cluster VI
NC_006033.2	W	684665	CAGL0J07084g	2889630	id3532	XM_448014.1	G544A	Ala182Thr	G	A	Ortholog(s) have oxidoreductase activity, acting on the CH-OH group of donors, NAD or NADP as acceptor activity
NC_006033.2	C	830033	CAGL0J08349g	2889704	id3591	XM_448072.1	A2201G	Asn734Ser	T	C	Ortholog(s) have role in regulation of transcription by RNA polymerase II
NC_006033.2	W	872358	CAGL0J08822g	2889678	id3612	XM_448092.1	A492C	Arg164Ser	A	C	Ortholog(s) have cyclin-dependent protein serine/threonine kinase regulator activity
NC_006034.2	W	590076	CAGL0K06039g	2890450	id4035	XM_448485.1	A446G	Asp149Gly	A	G	Ortholog(s) have chromatin DNA binding, structural molecule activity, role in meiotic recombination checkpoint, positive regulation of catalytic activity, synaptonemal complex assembly and lateral element localization
NC_006034.2	W	796412	CAGL0K08008g	2890371	id4126	XM_448569.1	G1819A	Ala607Thr	G	A	Ortholog of S. cerevisiae : YPR089W, C. albicans SC5314 : C1_04860W_A, C. dubliniensis CD36 : Cd36_04600, C. parapsilosis CDC317 : CPAR2_105630 and Candida tenuis NRRL Y-1498 : CANTEDRAFT_129373. Sce Ortholog: Protein of unknown function; exhibits genetic interaction with ERG11 and protein-protein interaction with Hsp82p
NC_006036.2	C	126710	CAGL0M01144g	2891164	id5031	XM_449394.1	C385T	Pro129Ser	G	A	Ortholog(s) have nuclear export signal receptor activity, role in tRNA re-export from nucleus and cytoplasm, nucleus localization
NC_006036.2	C	1285285	CAGL0M13035g	2891200	id5583	XM_449910.1	G802C	Ala268Pro	C	G	Ortholog(s) have role in protein ubiquitination, telomere maintenance, transcription-coupled nucleotide-excision repair, ubiquitin-dependent protein catabolic process and nucleus localization
NC_006036.2	C	1289915	CAGL0M13123g	2891204	id5587	XM_449914.1	T1691A	Leu564Gln	A	T	Protein of unknown function
NC_006036.2	W	1395763	CAGL0M14091g	2891619	id5632	XM_449957.1	C216A	Phe72Leu	C	A	Putative quinone reductase/NADPH dehydrogenase; gene is upregulated in azole-resistant strain
NC_006036.2	W	1396008	CAGL0M14091g	2891619	id5632	XM_449957.1	C461A	Thr154Lys	C	A	Putative quinone reductase/NADPH dehydrogenase; gene is upregulated in azole-resistant strain
NC_006036.2	W	1396019	CAGL0M14091g	2891619	id5632	XM_449957.1	C472T	Pro158Ser	C	T	Putative quinone reductase/NADPH dehydrogenase; gene is upregulated in azole-resistant strain

Caspofungin annotation

Gene ID	Entrez	Description	KEGG_PATHWAY	GOTERM_BP_DIRECT	GOTERM_CC_DIRECT	GOTERM_MF_DIRECT
CAGL0A01474g	2886376	Ortholog(s) have cell surface, extracellular region, fungal-type cell wall localization	NA		GO:0009277~fungal-type cell wall,	
CAGL0A02211g	2886382	Ortholog(s) have glucose transmembrane transporter activity, pentose transmembrane transporter activity, role in glucose transmembrane transport and plasma membrane localization	cgr04113:Meiosis - yeast,		GO:0016021~integral component of membrane,	GO:0022891~substrate-specific transmembrane transporter activity,
CAGL0B01859g	2886698	Ortholog(s) have ubiquitin-protein transferase activity, role in anaphase-promoting complex-dependent catabolic process, protein ubiquitination and anaphase-promoting complex, nuclear periphery localization	cgr04111:Cell cycle - yeast,cgr04113:Meiosis - yeast,cgr04120:Ubiquitin mediated proteolysis,	GO:0031145~anaphase-promoting complex-dependent catabolic process,	GO:0005680~anaphase-promoting complex,	GO:0004842~ubiquitin-protein transferase activity,
CAGL0C03960g	2886882	Has domain(s) with predicted transferase activity, transferring glycosyl groups activity and role in protein glycosylation	NA	GO:0006486~protein glycosylation,	GO:0016021~integral component of membrane,	GO:0016757~transferase activity, transferring glycosyl groups,
CAGL0E06666g	2887561	Epithelial adhesion protein; predicted GPI-anchor; belongs to adhesin cluster I	NA		GO:0016021~integral component of membrane,	
CAGL0F07733g	2887615	Ortholog(s) have ATP binding, ATPase activity, bubble DNA binding, dinucleotide insertion or deletion binding, heteroduplex DNA loop binding, single-stranded DNA binding activity	cgr03430:Mismatch repair,	GO:0000710~meiotic mismatch repair,GO:0000713~meiotic heteroduplex formation,GO:0007131~reciprocal meiotic recombination,	GO:0005739~mitochondrion,GO:0005829~cytosol,GO:0032389~MutLalpha complex,GO:0032390~MutLbeta complex,GO:0044732~mitotic spindle pole body,GO:0097587~MutLgamma complex,	GO:0000404~heteroduplex DNA loop binding,GO:0003697~single-stranded DNA binding,GO:0005524~ATP binding,GO:0016887~ATPase activity,GO:0032139~dinucleotide insertion or deletion binding,
CAGL0G00814g	2888154	Ortholog(s) have small GTPase binding activity	NA	GO:0000282~cellular bud site selection,GO:0006031~chitin biosynthetic process,GO:0006039~cell wall chitin catabolic process,GO:0006355~regulation of transcription, DNA-	GO:0005829~cytosol,GO:0034044~exomer complex,GO:0043332~mating projection tip,	

				templated,GO:0006893~Golgi to plasma membrane transport,GO:0009250~glucan biosynthetic process,GO:0030476~ascospore wall assembly,GO:0032219~cell wall macromolecule catabolic process involved in cytogamy,GO:0032220~plasma membrane fusion involved in cytogamy,		
CAGL0G09999g	2888156	Ortholog(s) have protein kinase regulator activity	cgr04140:Regulation of autophagy,	GO:0000422~mitophagy,GO:0000747~conjugation with cellular fusion,GO:0006995~cellular response to nitrogen starvation,GO:0032147~activation of protein kinase activity,GO:0032258~CVT pathway,GO:0034497~protein localization to pre-autophagosomal structure,GO:0034727~piecemeal microautophagy of nucleus,GO:0044805~late nucleophagy,	GO:0000407~pre-autophagosomal structure,GO:0005829~cytosol,GO:0019898~extrinsic component of membrane,GO:1990316~ATG1/ULK1 kinase complex,	GO:0019887~protein kinase regulator activity,
CAGL0G07755g	2888422	Ortholog(s) have telomeric DNA binding activity, role in protein localization to chromosome, telomere maintenance via telomerase and nuclear chromosome, telomeric region localization	NA	GO:0007004~telomere maintenance via telomerase,GO:0034502~protein localization to chromosome,	GO:0000784~nuclear chromosome, telomeric region,	GO:0042162~telomeric DNA binding,
CAGL0H10626g	2888521	Putative adhesin-like protein; belongs to adhesin cluster I; appears artificially broken into fragments due to sequencing errors; predicted GPI anchor	NA		GO:0009277~fungal-type cell wall,	
CAGL0H02805g	2888562	Ortholog(s) have ATPase activity, DNA binding, cohesin ATPase activity, double-stranded DNA binding, protein kinase binding, topological DNA entrapment activity	cgr04111:Cell cycle - yeast,cgr04113:Meiosis - yeast,	GO:0006281~DNA repair,GO:0007064~mitotic sister chromatid cohesion,	GO:0005634~nucleus,GO:0008280~cohesin core heterodimer,	GO:0003682~chromatin binding,GO:0005524~ATP binding,
CAGL0H05071g	2888697	Ortholog(s) have SUMO transferase activity, damaged DNA binding, double-stranded DNA-	NA	GO:0000724~double-strand break repair via homologous	GO:0005634~nucleus,GO:0030915~Smc5-Smc6	GO:0003684~damaged DNA binding,

		dependent ATPase activity, single-stranded DNA binding activity		recombination,GO:0051304~chromosome separation,GO:0071139~resolution of recombination intermediates,	complex,GO:0035861~site of double-strand break,	
CAGL0H03663g	2888820	Ortholog(s) have isocitrate dehydrogenase (NADP+) activity, role in fatty acid beta-oxidation and peroxisomal importomer complex, peroxisome localization	cgr00020:Citrate cycle (TCA cycle),cgr00480:Glutathione metabolism,cgr01100:Metabolic pathways,cgr01110:Biosynthesis of secondary metabolites,cgr01130:Biosynthesis of antibiotics,cgr01200:Carbon metabolism,cgr01210:2-Oxocarboxylic acid metabolism,cgr01230:Biosynthesis of amino acids,cgr04146:Peroxisome,	GO:0006099~tricarboxylic acid cycle,GO:0006102~isocitrate metabolic process,GO:0006635~fatty acid beta-oxidation,	GO:0005777~peroxisome,	GO:0000287~magnesium ion binding,GO:0004450~isocitrate dehydrogenase (NADP+) activity,GO:0051287~NAD binding,
CAGL0I03586g	2889183	Ortholog(s) have role in reciprocal meiotic recombination	NA	GO:0006298~mismatch repair,GO:0007131~reciprocal meiotic recombination,	GO:0005634~nucleus,	GO:0005524~ATP binding,GO:0030983~mismatched DNA binding,
CAGL0I07051g	2889184	Ortholog(s) have cyclin-dependent protein serine/threonine kinase regulator activity, role in positive regulation of macroautophagy and cyclin-dependent protein kinase holoenzyme complex localization	NA	GO:0000079~regulation of cyclin-dependent protein serine/threonine kinase activity,GO:0016239~positive regulation of macroautophagy,	GO:0000307~cyclin-dependent protein kinase holoenzyme complex,GO:0005634~nucleus,	GO:0016538~cyclin-dependent protein serine/threonine kinase regulator activity,
CAGL0J07084g	2889630	Ortholog(s) have oxidoreductase activity, acting on the CH-OH group of donors, NAD or NADP as acceptor activity	NA			GO:0016616~oxidoreductase activity, acting on the CH-OH group of donors, NAD or NADP as acceptor,GO:0051287~NA

						D binding,
CAGL0J08822g	2889678	Ortholog(s) have cyclin-dependent protein serine/threonine kinase regulator activity	cgr04111:Cell cycle - yeast,cgr04113:Meiosis - yeast,	GO:0000079~regulation of cyclin-dependent protein serine/threonine kinase activity,GO:0000086~G2/M transition of mitotic cell cycle,GO:0010696~positive regulation of spindle pole body separation,	GO:0005634~nucleus,GO:0005737~cytoplasm,GO:0005816~spindle pole body,	GO:0016538~cyclin-dependent protein serine/threonine kinase regulator activity,
CAGL0J08349g	2889704	Ortholog(s) have role in regulation of transcription by RNA polymerase II	cgr03018:RNA degradation,	GO:0006357~regulation of transcription from RNA polymerase II promoter,	GO:0030014~CCR4-NOT complex,	
CAGL0J02508g	2889767	Adhesin-like protein; identified in cell wall extracts by mass spectrometry; belongs to adhesin cluster VI; predicted GPI anchor	NA		GO:0009277~fungal-type cell wall,	
CAGL0K08008g	2890371	Ortholog of S. cerevisiae : YPR089W, C. albicans SC5314 : C1_04860W_A, C. dubliniensis CD36 : Cd36_04600, C. parapsilosis CDC317 : CPAR2_105630 and Candida tenuis NRRL Y-1498 : CANTEDRAFT_129373. Sce Ortholog: Protein of unknown function; exhibits genetic interaction with ERG11 and protein-protein interaction with Hsp82p	NA		GO:0005794~Golgi apparatus,	
CAGL0K06039g	2890450	Ortholog(s) have chromatin DNA binding, structural molecule activity, role in meiotic recombination checkpoint, positive regulation of catalytic activity, synaptonemal complex assembly and lateral element localization	cgr04113:Meiosis - yeast,	GO:0007130~synaptonemal complex assembly,GO:0007131~reciprocal meiotic recombination,GO:0043085~positive regulation of catalytic activity,GO:0051598~meiotic recombination checkpoint,	GO:0000800~lateral element,	GO:0005198~structural molecule activity,GO:0031490~chromatin DNA binding,
CAGL0M01144g	2891164	Ortholog(s) have nuclear export signal receptor activity, role in tRNA re-export from nucleus and cytoplasm, nucleus localization	cgr03013:RNA transport,	GO:0006611~protein export from nucleus,GO:0071528~tRNA re-export from nucleus,	GO:0005635~nuclear envelope,GO:0005829~cytosol,	
CAGL0M13035g	2891200	Ortholog(s) have role in protein ubiquitination, telomere maintenance, transcription-coupled nucleotide-excision repair, ubiquitin-dependent protein catabolic process and nucleus localization	NA	GO:0000723~telomere maintenance,GO:0006283~transcription-coupled nucleotide-excision	GO:0000781~chromosome, telomeric region,GO:0005634~nucleus,	GO:0003677~DNA binding,

				repair,GO:0006511~ubiquitin-dependent protein catabolic process,GO:0016567~protein ubiquitination,		
CAGL0E00 187g	2887325	Putative adhesin-like protein; belongs to adhesin cluster IV	NA	NA	NA	NA
CAGL0J02 530g	2889768	Putative adhesion protein; predicted GPI-anchor; belongs to adhesin cluster VI	NA	NA	NA	NA
CAGL0M1 3123g	2891204	Protein of unknown function	NA	NA	NA	NA
CAGL0M1 4091g	2891619	Putative quinone reductase/NADPH dehydrogenase; gene is upregulated in azole-resistant strain	NA	NA	NA	NA
CAGL0I10 147g	9488014	Protein with 32 tandem repeats; putative adhesin-like protein; belongs to adhesin cluster II	NA	NA	NA	NA

Fluconazole

chromosome	strand	coordinate	Gene ID	Entrez		geneBank ID	nucleotide_alleles	aminoacid_alleles	REF	ALLELE	description
NC_005967.2	W	170144	CAGL0A01716g	2886357	id70	XM_444840.1	A578C	Glu193Ala	A	C	Ortholog(s) have nicotinamidase activity, role in chromatin silencing at rDNA, chromatin silencing at telomere, negative regulation of DNA amplification, replicative cell aging and biofilm matrix, nucleus, peroxisome localization
NC_006026.1	C	33989	CAGL0C00319g	2886851	id461	XM_445195.1	A89G	Tyr30Cys	T	C	Ortholog(s) have carboxypeptidase activity, role in proteolysis involved in cellular protein catabolic process and fungal-type vacuole lumen localization
NC_006026.1	C	33868	CAGL0C00319g	2886851	id461	XM_445195.1	G210T	Leu70Phe	C	A	Ortholog(s) have carboxypeptidase activity, role in proteolysis involved in cellular protein catabolic process and fungal-type vacuole lumen localization
NC_006026.1	C	33931	CAGL0C00319g	2886851	id461	XM_445195.1	T147A	Ser49Arg	A	T	Ortholog(s) have carboxypeptidase activity, role in proteolysis involved in cellular protein catabolic process and fungal-type vacuole lumen localization
NC_006026.1	C	61866	CAGL0C00561g	2886768	id473	XM_445206.1	G28T	Gly10Cys	C	A	Ortholog(s) have phosphatidylinositol-3-phosphate binding, phosphatidylinositol-5-phosphate binding, phosphatidylserine binding activity
NC_006026.1	W	62562	CAGL0C00583g	2886844	id474	XM_445207.1	A339T	Leu113Phe	A	T	Ortholog(s) have mRNA binding, sequence-specific mRNA binding activity, role in endoplasmic reticulum inheritance, intracellular mRNA localization, mating type switching and cellular bud tip localization
NC_006026.1	W	62734	CAGL0C00583g	2886844	id474	XM_445207.1	A511G	Ile171Val	A	G	Ortholog(s) have mRNA binding, sequence-specific mRNA binding activity, role in endoplasmic reticulum inheritance, intracellular mRNA localization, mating type switching and cellular bud tip localization
NC_006027.1	W	294958	CAGL0D02816g	2887173	id829	XM_445538.1	A528T	Glu176Asp	A	T	Ortholog(s) have polynucleotide 5'-hydroxyl-kinase activity
NC_006028.2	W	87514	CAGL0E00957g	2887359	id1049	XM_445729.1	C833T	Ala278Val	C	T	Ortholog(s) have sequence-specific mRNA binding activity
NC_006028.2	W	87671	CAGL0E00957g	2887359	id1049	XM_445729.1	A990G	Ile330Met	A	G	Ortholog(s) have role in Golgi to plasma membrane transport, endoplasmic reticulum inheritance, establishment or maintenance of cell polarity, exocyst assembly
NC_006028.2	W	87991	CAGL0E00957g	2887359	id1049	XM_445729.1	G1310A	Gly437Glu	G	A	Ortholog(s) have role in Golgi to plasma membrane transport, endoplasmic reticulum inheritance, establishment or maintenance of cell polarity, exocyst assembly

NC_006029.1	W	283387	CAGL0F02915g	2887745	id1437	XM_446097.1	A2378G	Lys793Arg	A	G	Ortholog(s) have role in generation of catalytic spliceosome for first transesterification step
NC_006029.1	W	556978	CAGL0F05511g	2887805	id1555	XM_446210.1	T1333C	Trp445Arg	T	C	Ortholog(s) have protein-lysine N-methyltransferase activity and role in peptidyl-lysine monomethylation, peptidyl-lysine trimethylation
NC_006031.1	C	440553	CAGL0H04609g	2888643	id2418	XM_446995.1	G1238A	Gly413Asp	C	T	Ortholog(s) have protein serine/threonine kinase activity, telomeric DNA binding activity and role in DNA damage induced protein phosphorylation, double-strand break repair, histone phosphorylation, telomere maintenance
NC_006031.1	C	441678	CAGL0H04609g	2888643	id2418	XM_446995.1	T113G	Phe38Cys	A	C	Ortholog(s) have protein serine/threonine kinase activity, telomeric DNA binding activity and role in DNA damage induced protein phosphorylation, double-strand break repair, histone phosphorylation, telomere maintenance
NC_006031.1	C	674724	CAGL0H06765g	2888896	id2521	XM_447090.1	G1780A	Gly594Ser	C	T	Ortholog(s) have profilin binding activity, role in barbed-end actin filament capping, formin-nucleated actin cable assembly, positive regulation of actin cytoskeleton reorganization and cell division site, cellular bud neck localization
NC_006032.2	C	259332	CAGL0I02948g	2889138	id2829	XM_447379.1	T158C	Ile53Thr	A	G	Ortholog(s) have role in cellular response to drug, fungal-type cell wall organization, retrograde vesicle-mediated transport, Golgi to ER and SNARE complex, endoplasmic reticulum, integral component of membrane, peroxisome localization
NC_006033.2	W	417147	CAGL0J04422g	2889636	id3407	XM_447898.1	A633T	Glu211Asp	A	T	Ortholog(s) have ATP-dependent peptidase activity and role in chaperone-mediated protein complex assembly, mitochondrial protein catabolic process, protein quality control for misfolded or incompletely synthesized proteins
NC_006033.2	C	474667	CAGL0J04950g	2889563	id3432	XM_447921.1	C178T	Leu60Phe	G	A	Ortholog(s) have role in mitotic recombination and cellular bud localization
NC_006033.2	C	474664	CAGL0J04950g	2889563	id3432	XM_447921.1	G181A	Ala61Thr	C	T	Ortholog(s) have role in mitotic recombination and cellular bud localization
NC_006033.2	C	502935	CAGL0J05214g	2889504	id3442	XM_447931.1	G1135A	Val379Ile	C	T	Ortholog(s) have role in mRNA export from nucleus, protein import into nucleus, ribosomal large subunit export from nucleus, ribosomal small subunit export from nucleus
NC_006033.2	C	504849	CAGL0J05236g	2889505	id3443	XM_447932.1	G2224A	Val742Ile	C	T	Ortholog(s) have mannose-ethanolamine phosphotransferase activity
NC_006033.2	C	504690	CAGL0J05236g	2889505	id3443	XM_447932.1	T2383G	Cys795Gly	A	C	Ortholog(s) have mannose-ethanolamine phosphotransferase activity
NC_006033.2	W	981055	CAGL0J09988g	288942	id3667	XM_448144.1	T656G	Leu219Arg	T	G	Ortholog(s) have S-adenosylmethionine-dependent methyltransferase activity and role in translational readthrough

				9							
NC_006036.2	W	1279838	CAGL0M12969g	2891197	id5580	XM_449907.1	G987C	Glu329Asp	G	C	S.cerevisiae ortholog: YIL007C; Putative protein of unknown function
NC_006036.2	C	1283338	CAGL0M13013g	2891199	id5582	XM_449909.1	G316A	Asp106Asn	C	T	Ortholog(s) have 3-oxoacyl-[acyl-carrier-protein] reductase (NADPH) activity, role in aerobic respiration, fatty acid metabolic process and mitochondrion localization

Fluconazole annotations

Gene ID	Entrez	description	KEGG_PATHWAY	GOTERM_BP_DIRECT	GOTERM_CC_DIRECT	GOTERM_MF_DIRECT
CAGL0A01716g	2886357	Ortholog(s) have nicotinamidase activity, role in chromatin silencing at rDNA, chromatin silencing at telomere, negative regulation of DNA amplification, replicative cell aging and biofilm matrix, nucleus, peroxisome localization	cgr00760:Nicotinate and nicotinamide metabolism,cgr01100:Metabolic pathways,	GO:0000183~chromatin silencing at rDNA,GO:0001302~replicative cell aging,GO:0006348~chromatin silencing at telomere,GO:0008152~metabolic process,	GO:0000781~chromosome, telomeric region,GO:0005634~nucleus,GO:0005777~peroxisome,GO:0005829~cytosol,	GO:0008936~nicotinamidase activity,
CAGL0C00561g	2886768	Ortholog(s) have phosphatidylinositol-3-phosphate binding, phosphatidylinositol-5-phosphate binding, phosphatidylserine binding activity		GO:0006914~autophagy,GO:0044395~protein targeting to vacuolar membrane,GO:0048278~vesicle docking,	GO:0032585~multivesicular body membrane,GO:0035658~Mon1-Ccz1 complex,	GO:0001786~phosphatidylserine binding,GO:0010314~phosphatidylinositol-5-phosphate binding,GO:0017112~Rab guanyl-nucleotide exchange factor activity,GO:0032266~phosphatidylinositol-3-phosphate binding,
CAGL0C00583g	2886844	Ortholog(s) have mRNA binding, sequence-specific mRNA binding activity, role in endoplasmic reticulum inheritance, intracellular mRNA localization, mating type switching and cellular bud tip localization		GO:0048309~endoplasmic reticulum inheritance,GO:0051028~mRNA transport,	GO:0005789~endoplasmic reticulum membrane,	GO:0003723~RNA binding,
CAGL0C00319g	2886851	Ortholog(s) have carboxypeptidase activity, role in proteolysis involved in cellular protein catabolic process and fungal-type vacuole lumen localization		GO:0006807~nitrogen compound metabolic process,GO:0051603~proteolysis involved in cellular protein catabolic process,	GO:0000328~fungal-type vacuole lumen,GO:0016021~integral component of membrane,	GO:0004181~metallocarboxypeptidase activity,
CAGL0D02816g	2887173	Ortholog(s) have polynucleotide 5'-hydroxyl-kinase activity		GO:0000448~cleavage in ITS2 between 5.8S rRNA and LSU-rRNA of tricistronic rRNA transcript (SSU-rRNA, 5.8S rRNA, LSU-rRNA),GO:0006363~termination of RNA polymerase I transcription,GO:0006364~rRNA	GO:0005724~nuclear telomeric heterochromatin,GO:0030874~nucleolar chromatin,GO:0031618~nuclear pericentric heterochromatin,GO:003	GO:0005524~ATP binding,GO:0051731~polynucleotide 5'-hydroxyl-kinase activity,

				processing,GO:0030702~chromatin silencing at centromere,	1934~mating-type region heterochromatin,GO:0097356~perinucleolar compartment,	
CAGL0E00957g	2887359	Ortholog(s) have sequence-specific mRNA binding activity		GO:0001927~exocyst assembly,GO:0006893~Golgi to plasma membrane transport,GO:0048309~endoplasmic reticulum inheritance,	GO:0000131~incipient cellular bud site,GO:0000145~exocyst,GO:0005934~cellular bud tip,GO:0005935~cellular bud neck,	
CAGL0F02915g	2887745	Ortholog(s) have role in generation of catalytic spliceosome for first transesterification step	cgr03040:Spliceosome,	GO:0000349~generation of catalytic spliceosome for first transesterification step,	GO:0000974~Prp19 complex,GO:0005829~cytosol,GO:0071004~U2-type prespliceosome,GO:0071006~U2-type catalytic step 1 spliceosome,GO:0071007~U2-type catalytic step 2 spliceosome,GO:0071008~U2-type post-mRNA release spliceosomal complex,	
CAGL0F05511g	2887805	Ortholog(s) have protein-lysine N-methyltransferase activity and role in peptidyl-lysine monomethylation, peptidyl-lysine trimethylation		GO:0018023~peptidyl-lysine trimethylation,GO:0018026~peptidyl-lysine monomethylation,	GO:0005730~nucleolus, GO:0005829~cytosol,	GO:0016279~protein-lysine N-methyltransferase activity,
CAGL0H04609g	2888643	Ortholog(s) have protein serine/threonine kinase activity, telomeric DNA binding activity and role in DNA damage induced protein phosphorylation, double-strand break repair, histone phosphorylation, telomere maintenance		GO:0000077~DNA damage checkpoint,GO:0000723~telomere maintenance,GO:0006302~double-strand break repair,GO:0006975~DNA damage induced protein phosphorylation,GO:0010212~response to ionizing radiation,GO:0016572~histone phosphorylation,GO:0090399~replicative senescence,	GO:0000781~chromosome, telomeric region,GO:0005634~nucleus,	GO:0004674~protein serine/threonine kinase activity,GO:0005524~ATP binding,GO:0042162~telomeric DNA binding,
CAGL0	288	Ortholog(s) have profilin binding		GO:0051016~barbed-end actin filament	GO:0005935~cellular	

H06765g	8896	activity, role in barbed-end actin filament capping, formin-nucleated actin cable assembly, positive regulation of actin cytoskeleton reorganization and cell division site, cellular bud neck localization		capping,GO:0070649~formin-nucleated actin cable assembly,GO:2000251~positive regulation of actin cytoskeleton reorganization,	bud neck,	
CAGL0I02948g	2889138	Ortholog(s) have role in cellular response to drug, fungal-type cell wall organization, retrograde vesicle-mediated transport, Golgi to ER and SNARE complex, endoplasmic reticulum, integral component of membrane, peroxisome localization	cgr04130:SNARE interactions in vesicular transport,	GO:0006890~retrograde vesicle-mediated transport, Golgi to ER,	GO:0005783~endoplasmic reticulum,GO:0016021~integral component of membrane,GO:0031201~SNARE complex,	
CAGL0J09988g	2889429	Ortholog(s) have S-adenosylmethionine-dependent methyltransferase activity and role in translational readthrough			GO:0005737~cytoplasm,	GO:0003676~nucleic acid binding,GO:0008276~protein methyltransferase activity,
CAGL0J05214g	2889504	Ortholog(s) have role in mRNA export from nucleus, protein import into nucleus, ribosomal large subunit export from nucleus, ribosomal small subunit export from nucleus		GO:0000055~ribosomal large subunit export from nucleus,GO:0000056~ribosomal small subunit export from nucleus,GO:0006406~mRNA export from nucleus,GO:0006606~protein import into nucleus,GO:0006611~protein export from nucleus,	GO:0005829~cytosol,GO:0044612~nuclear pore linkers,GO:0044614~nuclear pore cytoplasmic filaments,	
CAGL0J05236g	2889505	Ortholog(s) have mannose-ethanolamine phosphotransferase activity	cgr00563:Glycosylphosphatidylinositol(GPI)-anchor biosynthesis,	GO:0006506~GPI anchor biosynthetic process,	GO:0005887~integral component of plasma membrane,GO:0030176~integral component of endoplasmic reticulum membrane,	GO:0051377~mannose-ethanolamine phosphotransferase activity,
CAGL0J04950g	2889563	Ortholog(s) have role in mitotic recombination and cellular bud localization		GO:0006312~mitotic recombination,	GO:0005933~cellular bud,GO:0016021~integral component of membrane,	
CAGL0J04422g	2889636	Ortholog(s) have ATP-dependent peptidase activity and role in chaperone-mediated protein complex		GO:0006515~misfolded or incompletely synthesized protein catabolic process,GO:0034599~cellular	GO:0005759~mitochondrial matrix,	GO:0004176~ATP-dependent peptidase activity,GO:0004252~serine-

		assembly, mitochondrial protein catabolic process, protein quality control for misfolded or incompletely synthesized proteins		response to oxidative stress,GO:0051131~chaperone-mediated protein complex assembly,GO:0070407~oxidation-dependent protein catabolic process,GO:0090296~regulation of mitochondrial DNA replication,		type endopeptidase activity,GO:0005524~ATP binding,GO:0043565~sequence-specific DNA binding,
CAGL0M12969g	2891197	S.cerevisiae ortholog: YIL007C; Putative protein of unknown function			GO:0016021~integral component of membrane,	
CAGL0M13013g	2891199	Ortholog(s) have 3-oxoacyl-[acyl-carrier-protein] reductase (NADPH) activity, role in aerobic respiration, fatty acid metabolic process and mitochondrion localization	cgr00061:Fatty acid biosynthesis,cgr00780:Biotin metabolism,cgr01040:Biosynthesis of unsaturated fatty acids,cgr01100:Metabolic pathways,cgr01212:Fatty acid metabolism,	GO:0006631~fatty acid metabolic process,GO:0009060~aerobic respiration,	GO:0005739~mitochondrion,	GO:0004316~3-oxoacyl-[acyl-carrier-protein] reductase (NADPH) activity,

Voriconazole

chromosome	strand	coordinate	Gene ID	Entrez		geneBankID	nucleotide_alleles	aminoacid_alleles	REF	ALLELE	Description
NC_005967.2	W	350898	CAGL0A03432g	2886385	id156	XM_444917.1	T1015A	Phe339Ile	T	A	Ortholog(s) have DNA-dependent ATPase activity, Y-form DNA binding, four-way junction DNA binding, four-way junction helicase activity, ubiquitin protein ligase activity
NC_005967.2	W	358393	CAGL0A03432g	2886385	id159	XM_444920.1	G1286T	Thr429Asn	G	T	Ortholog(s) have DNA-dependent ATPase activity, Y-form DNA binding, four-way junction DNA binding, four-way junction helicase activity, ubiquitin protein ligase activity
NC_005968.1	W	289504	CAGL0B02948g	2886719	id354	XM_445100.1	T296A	Tyr99Phe	T	A	Beta mannosyltransferase
NC_005968.1	W	437036	CAGL0B04455g	2886660	id423	XM_445164.1	T1600C	Phe534Leu	T	C	Ortholog(s) have L-glutamine transmembrane transporter activity, L-isoleucine transmembrane transporter activity, L-tyrosine transmembrane transporter activity and role in amino acid transmembrane export from vacuole
NC_005968.1	W	437421	CAGL0B04477g	2886560	id424	XM_445165.1	G788A	Ala263Val	G	A	Ortholog(s) have cellular bud neck, plasma membrane localization
NC_006026.1	W	388124	CAGL0C03938g	2886892	id624	XM_445347.1	T463A	Thr155Ser	T	A	Has domain(s) with predicted transferase activity, transferring glycosyl groups activity and role in protein glycosylation
NC_006026.1	C	393677	CAGL0C03982g	2886873	id626	XM_445349.1	A1213G	Ser405Pro	C	A	Has domain(s) with predicted transferase activity, transferring glycosyl groups activity and role in protein glycosylation
NC_006026.1	C	392757	CAGL0C03982g	2886873	id626	XM_445349.1	G1042T	His348Asn	G	T	Has domain(s) with predicted transferase activity, transferring glycosyl groups activity and role in protein glycosylation
NC_006026.1	C	392586	CAGL0C03982g	2886873	id626	XM_445349.1	C122T	Ser41Ile	A	G	Has domain(s) with predicted transferase activity, transferring glycosyl groups activity and role in protein glycosylation
NC_006026.1	C	469584	CAGL0C05005g	2886923	id673	XM_445391.1	A1273C	Ser425Ala	T	G	Ortholog(s) have protein kinase activity, role in meiotic spindle disassembly, mitotic cytokinesis, protein phosphorylation, regulation of exit from mitosis and cellular bud neck, spindle pole body localization
NC_006027.1	W	73725	CAGL0D00572g	2887254	id723	XM_445438.1	C2359T	Val787Ile	C	T	Ortholog(s) have Rab GTPase binding, Ras guanyl-nucleotide exchange factor activity, phosphatidylinositol binding activity
NC_006027.1	W	292374	CAGL0D02794g	2887008	id828	XM_445537.1	A341C	Asn114Thr	A	C	Protein of unknown function
NC_006027.1	W	338800	CAGL0D03278g	2887020	id850	XM_445559.1	C65G	Ala22Gly	C	G	Ortholog(s) have role in chaperone-mediated protein complex assembly, nuclear-transcribed mRNA catabolic process, non-stop decay, proteasome assembly and intracellular localization

NC_006027.1	W	565026	CAGL0D05918g	2887255	id975	XM_445666.1	C1639T	Arg547Cys	C	T	Putative alcohol acetyltransferase involved in steroid detoxification; gene is upregulated in azole-resistant strain
NC_006028.2	W	210977	CAGL0E02101g	2887400	id1104	XM_445781.1	A1421T	Asp474Val	A	T	Ortholog(s) have 5'-deoxyribose-5-phosphate lyase activity, ATP-dependent 3'-5' RNA helicase activity, polynucleotide adenylyltransferase activity
NC_006028.2	W	213651	CAGL0E02167g	2887280	id1107	XM_445784.1	A389G	Val130Ala	A	G	Ortholog(s) have role in cell morphogenesis involved in conjugation with cellular fusion, posttranslational protein targeting to endoplasmic reticulum membrane, protein insertion into ER membrane
NC_006028.2	W	488902	CAGL0E05038g	2887378	id1238	XM_445907.1	G117A	Met39Ile	G	A	Ortholog(s) have ATP-dependent 3'-5' DNA helicase activity, ATPase activity
NC_006028.2	W	489473	CAGL0E05038g	2887378	id1238	XM_445907.1	G688T	Asp230Tyr	G	T	Ortholog(s) have 3'-5' DNA helicase activity, ATPase activity
NC_006028.2	W	492305	CAGL0E05038g	2887378	id1238	XM_445907.1	G3520A	Asp1174Asn	G	A	Ortholog(s) have 3'-5' DNA helicase activity, ATPase activity
NC_006028.2	W	492309	CAGL0E05038g	2887378	id1238	XM_445907.1	C3524T	Ser1175Phe	C	T	Ortholog(s) have 3'-5' DNA helicase activity, ATPase activity
NC_006028.2	W	577611	CAGL0E05808g	2887447	id1273	XM_445941.1	A713C	Asn238Thr	A	C	Bifunctional enzyme of thiamine biosynthesis, with thiamine-phosphate pyrophosphorylase and 4-methyl-5-beta-hydroxyethylthiazole kinase activities; active as a homohexamer
NC_006028.2	W	581492	CAGL0E05874g	2887443	id1275	XM_445944.1	G736A	Ala246Thr	G	A	Ortholog(s) have GTP binding, GTPase activity, U3 snoRNA binding activity
NC_006028.2	W	589049	CAGL0E05940g	2887441	id1279	XM_445947.1	C1924T	Ala642Thr	C	T	Ortholog(s) have FAD transmembrane transporter activity, calcium channel activity
NC_006028.2	W	597430	CAGL0E06006g	2887415	id1282	XM_445950.1	A1055T	Asp352Val	A	T	Ortholog(s) have role in cellular iron ion homeostasis and mitochondrion localization
NC_006028.2	W	598167	CAGL0E06028g	2887416	id1283	XM_445951.1	G148A	Asp50Asn	G	A	Ortholog(s) have dolichyl-phosphate beta-glucosyltransferase activity and role in dolichol-linked oligosaccharide biosynthetic process
NC_006028.2	W	605376	CAGL0E06116g	2887354	id1287	XM_445955.1	G1411A	Pro471Ser	G	A	Ortholog(s) have role in positive regulation of transcription by RNA polymerase II and cytosol, nuclear chromatin localization
NC_006030.1	W	988772	CAGL0G10219g	2888103	id2213	XM_446805.1	G7C	Gly3Arg	G	C	Adhesin-like protein with 5 tandem repeats; belongs to adhesin cluster VII; predicted GPI-anchor; similarity to S. cerevisiae flocculins, cell wall proteins that mediate adhesion
NC_006029.1	W	290133	CAGL0F03003g	2887960	id1441	XM_446100.1	G211A	Val71Ile	G	A	Ortholog(s) have osmosensor activity and role in (1->3)-beta-D-glucan biosynthetic process, cellular bud site selection, fungal-type cell wall organization, hyperosmotic response, osmosensory signaling pathway via Sho1 osmosensor
NC_006029.1	W	869037	CAGL0F08789g	2887777	id1706	XM_446351.1	G1036A	Ala346Thr	G	A	Ortholog of S. cerevisiae : MCY1; Putative cysteine synthase

NC_006029.1	W	870492	CAGL0F08811g	2887569	id1707	XM_446352.1	A1026T	Lys342Asn	A	T	Ortholog(s) have RNA binding activity, role in mRNA splicing, via spliceosome and U1 snRNP, U2-type prespliceosome localization
NC_006029.1	W	870554	CAGL0F08811g	2887569	id1707	XM_446352.1	G1088A	Ser363Asn	G	A	Ortholog(s) have RNA binding activity, role in mRNA splicing, via spliceosome and U1 snRNP, U2-type prespliceosome localization
NC_006029.1	W	874546	CAGL0F08833g	2887570	id1708	XM_446353.1	C698T	Ser233Phe	C	T	Putative adhesin-like protein
NC_006029.1	W	879542	CAGL0F08943g	2887851	id1713	XM_446356.1	G107T	Gly36Val	G	T	Ortholog(s) have role in mitochondrial translation and mitochondrion localization
NC_006029.1	W	884967	CAGL0F09009g	2887603	id1717	XM_446359.1	T1004C	Val335Ala	T	C	Ortholog(s) have role in snoRNA metabolic process
NC_006030.1	W	261203	CAGL0G02827g	2888354	id1859	XM_446486.1	T616C	Thr206Ala	T	C	Ortholog(s) have phosphatidylinositol-4,5-bisphosphate binding, sphingolipid binding activity
NC_006030.1	W	273378	CAGL0G02959g	2888352	id1865	XM_446492.1	T859C	Ser287Pro	T	C	Ortholog of S. cerevisiae : NNF2; Protein that exhibits physical and genetic interactions with Rpb8p
NC_006030.1	W	809081	CAGL0G08602g	2888126	id2137	XM_446736.1	G727A	Val243Ile	G	A	Ortholog(s) have GTPase regulator activity, role in Ras protein signal transduction, fungal-type cell wall biogenesis, positive regulation of transcription by RNA polymerase II and nucleus localization
NC_006030.1	W	894398	CAGL0G09361g	2888035	id2173	XM_446769.1	C785T	Ser262Leu	C	T	Ortholog(s) have structural molecule activity, role in acetyl-CoA biosynthetic process from pyruvate, filamentous growth, single-species biofilm formation on inanimate substrate and mitochondrial pyruvate dehydrogenase complex localization
NC_006031.1	W	466237	CAGL0H04873g	2888854	id2431	XM_447007.1	A188G	Val63Ala	A	G	Ortholog(s) have sequence-specific DNA binding activity and role in filamentous growth
NC_006031.1	W	920827	CAGL0H09394g	2888537	id2643	XM_447207.1	C547T	Asp183Asn	C	T	Protein of unknown function
NC_006032.2	W	351910	CAGL0I04004g	2889095	id2876	XM_447423.1	C648G	Leu216Phe	C	G	Protein of unknown function
NC_006032.2	W	480131	CAGL0I05104g	2889090	id2933	XM_447472.1	C575T	Arg192Lys	C	T	Has domain(s) with predicted ATP binding, aminoacyl-tRNA ligase activity, proline-tRNA ligase activity and role in prolyl-tRNA aminoacylation, tRNA aminoacylation for protein translation
NC_006032.2	W	602148	CAGL0I06248g	2889154	id2986	XM_447522.1	C1798T	Leu600Phe	C	T	Ortholog(s) have role in cellular cation homeostasis, cellular protein localization, protein dephosphorylation
NC_006032.2	W	776549	CAGL0I07953g	2889235	id3068	XM_447595.1	C1829A	Thr610Asn	C	A	Ortholog(s) have phosphatidylinositol-4,5-bisphosphate binding activity and role in establishment or maintenance of actin cytoskeleton polarity, fungal-type cell wall organization, regulation of cell growth
NC_006032.2	W	906599	CAGL0I09460g	2888931	id3138	XM_447658.1	T1038A	Lys346Asn	T	A	Ortholog(s) have role in mismatch repair, proteasome regulatory particle assembly and cytosol, nucleus, proteasome regulatory particle, base

											subcomplex localization
NC_006032.2	W	1065473	CAGL0I10769g	2889399	id3192	XM_447708.1	T485G	Asn162Thr	T	G	Has domain(s) with predicted DNA binding, protein dimerization activity
NC_006033.2	W	268976	CAGL0J02706g	2889752	id3324	XM_447822.1	C129A	Glu43Asp	C	A	Ortholog(s) have role in meiotic DNA double-strand break formation and condensed nuclear chromosome localization
NC_006033.2	W	268992	CAGL0J02706g	2889752	id3324	XM_447822.1	G113A	Ser38Leu	G	A	Ortholog(s) have role in meiotic DNA double-strand break formation and condensed nuclear chromosome localization
NC_006033.2	W	631211	CAGL0J06556g	2889543	id3507	XM_447991.1	T234A	Ser78Arg	T	A	Ortholog(s) have phosphatidylinositol-3-phosphate binding activity, role in mitochondrion inheritance, nucleus inheritance, vacuole-ER tethering and nucleus-vacuole junction localization
NC_006034.2	W	26890	CAGL0K00275g	2889973	id3763	XM_448236.1	C1387A	Ala463Ser	C	A	Ortholog(s) have phospholipase activity, ubiquitin binding activity
NC_006034.2	W	45865	CAGL0K00429g	2890189	id3770	XM_448243.1	G607T	Ala203Ser	G	T	Has domain(s) with predicted nucleotide binding activity
NC_006034.2	W	47218	CAGL0K00451g	2890187	id3771	XM_448244.1	A121C	Cys41Gly	A	C	Ortholog(s) have role in proteasome assembly and cytosol localization
NC_006034.2	W	54270	CAGL0K00495g	2890185	id3773	XM_448246.1	G809A	Arg270Lys	G	A	Ortholog(s) have eukaryotic initiation factor 4E binding activity and role in deadenylation-dependent decapping of nuclear-transcribed mRNA, negative regulation of translation
NC_006034.2	W	60054	CAGL0K00517g	2890183	id3774	XM_448247.1	G3109A	Leu1037Phe	G	A	Ortholog(s) have protein serine/threonine kinase activity
NC_006034.2	W	70635	CAGL0K00605g	2890231	id3778	XM_448250.1	T1524G	Asn508Lys	T	G	Ortholog(s) have ATP binding, ATPase activity, DNA replication origin binding, GTP binding, GTPase activity, chromatin binding, protein serine/threonine kinase activity
NC_006034.2	W	76884	CAGL0K00693g	2890514	id3783	XM_448254.1	C1213T	Asp405Asn	C	T	Ortholog(s) have protein tyrosine kinase activity
NC_006034.2	W	77831	CAGL0K00693g	2890514	id3783	XM_448254.1	G266A	Ser89Phe	G	A	Ortholog(s) have protein tyrosine kinase activity
NC_006034.2	W	129878	CAGL0K01485g	2890059	id3819	XM_448289.1	C1318G	Glu440Gln	C	G	Ortholog(s) have pseudouridine synthase activity, role in tRNA pseudouridine synthesis and mitochondrion localization
NC_006034.2	W	250729	CAGL0K02761g	2890271	id3879	XM_448345.1	A837C	Arg279Ser	A	C	Ortholog(s) have ubiquitin binding activity
NC_006034.2	W	251790	CAGL0K02783g	2890272	id3880	XM_448346.1	C153A	Asn51Lys	C	A	Ortholog(s) have actin filament binding activity
NC_006034.2	W	253406	CAGL0K02805g	2890128	id3881	XM_448347.1	A219G	Ile73Met	A	G	Predicted inositolphosphorylceramide (IPC) synthase, catalyzes the essential step in sphingolipid biosynthesis; potential antifungal drug target

NC_006034.2	W	422622	CAGL0K04455g	2889998	id3959	XM_448419.1	A832C	Ile278Leu	A	C	Ortholog(s) have role in ascospore formation and ascospore wall, prospore membrane, septin complex localization
NC_006034.2	W	535263	CAGL0K05445g	2890486	id4005	XM_448462.1	G584A	Ser195Asn	G	A	Ortholog(s) have role in centromere complex assembly, establishment of protein localization to chromosome, kinetochore organization and maintenance of meiotic sister chromatid cohesion
NC_006035.2	W	170176	CAGL0L01507g	2890878	id4422	XM_448838.1	A2968G	Ile990Val	A	G	Ortholog(s) have U2 snRNA binding activity, role in spliceosomal complex assembly and U2 snRNP, U2-type prespliceosome localization
NC_006035.2	W	216184	CAGL0L01881g	2890673	id4440	XM_448851.1	T35C	Val12Ala	T	C	Has domain(s) with predicted integral component of membrane localization
NC_006035.2	W	217308	CAGL0L01881g	2890673	id4440	XM_448851.1	A1159G	Ile387Val	A	G	Has domain(s) with predicted integral component of membrane localization
NC_006035.2	W	303918	CAGL0L02585g	2890856	id4473	XM_448883.1	A1929C	Asp643Glu	A	C	Has domain(s) with predicted DNA binding, chromatin binding activity
NC_006035.2	W	307163	CAGL0L02607g	2890859	id4474	XM_448884.1	A1184G	Leu395Ser	A	G	Has domain(s) with predicted hydrolase activity and role in nucleotide catabolic process
NC_006035.2	W	307754	CAGL0L02607g	2890859	id4474	XM_448884.1	T593C	Lys198Arg	T	C	Has domain(s) with predicted hydrolase activity and role in nucleotide catabolic process
NC_006035.2	W	318808	CAGL0L02695g	2890861	id4478	XM_448888.1	T811G	Phe271Val	T	G	Protein of unknown function
NC_006035.2	W	379046	CAGL0L03289g	2890726	id4506	XM_448915.1	C217G	Pro73Ala	C	G	Ortholog(s) have role in autophagy of mitochondrion, fungal-type cell wall biogenesis, fungal-type cell wall organization
NC_006035.2	W	536521	CAGL0L04642g	2890647	id4570	XM_448977.1	G135T	Asp45Glu	G	T	Ortholog(s) have 1-acylglycerol-3-phosphate O-acyltransferase activity, 1-acylglycerophosphocholine O-acyltransferase activity, role in glycerophospholipid biosynthetic process and endoplasmic reticulum localization
NC_006035.2	W	551281	CAGL0L04796g	2890707	id4577	XM_448984.1	C344T	Ala115Val	C	T	Protein of unknown function
NC_006035.2	W	553443	CAGL0L04818g	2890708	id4578	XM_448985.1	G211A	Arg71Cys	G	A	Ortholog of S. cerevisiae : YGR117C; Putative protein of unknown function
NC_006035.2	W	554360	CAGL0L04832g	9488040	id4579	XM_002999543.1	T119G	His40Pro	T	G	Protein of unknown function
NC_006035.2	W	653058	CAGL0L05852g	2890809	id4627	XM_449029.1	G844T	Pro282Thr	G	T	Ortholog(s) have structural constituent of nuclear pore activity
NC_006035.2	W	658880	CAGL0L05874g	2890810	id4628	XM_449030.1	A3944G	Glu1315Gly	A	G	Ortholog(s) have 5'-3' exoribonuclease activity, chromatin binding, magnesium ion binding, microtubule binding, recombinase activity
NC_006035.2	W	659047	CAGL0L05874g	2890810	id4628	XM_449030.1	A4111T	Met1371Leu	A	T	Ortholog(s) have 5'-3' exoribonuclease activity, chromatin binding, magnesium ion binding, microtubule binding, recombinase activity

NC_0060 35.2	W	65989 7	CAGL0L0 5896g	2890811	id462 9	XM_44903 1.1	A434 G	Val145 Ala	A	G	Ortholog(s) have role in mRNA splicing, via spliceosome and RES complex, U2-type spliceosomal complex localization
NC_0060 35.2	W	71824 9	CAGL0L0 6358g	2890651	id465 1	XM_44905 2.1	T215C	Lys72A rg	T	C	Ortholog of S. cerevisiae : TMS1; Vacuolar membrane protein of unknown function
NC_0060 35.2	W	72108 2	CAGL0L0 6402g	2890653	id465 4	XM_44905 3.1	A360T	Gln120 His	A	T	Ortholog(s) have bilirubin transmembrane transporter activity, glutathione S-conjugate-exporting ATPase activity, phytochelatin transmembrane transporter ATPase activity
NC_0060 35.2	W	93765 3	CAGL0L0 8580g	2890625	id476 0	XM_44914 9.1	A430 G	Asn144 Asp	A	G	Putative 26S proteasome regulatory subunit; protein abundance decreased in ace2 mutant cells
NC_0060 35.2	W	94671 7	CAGL0L0 8646g	2890628	id476 3	XM_44915 2.1	A64G	Ile22Va l	A	G	Ortholog(s) have SUMO-specific isopeptidase activity, role in G2/M transition of mitotic cell cycle, protein desumoylation and nuclear envelope, nuclear pore, nucleolus localization
NC_0060 35.2	W	94681 1	CAGL0L0 8646g	2890628	id476 3	XM_44915 2.1	C158T	Ala53V al	C	T	Ortholog(s) have SUMO-specific isopeptidase activity, role in G2/M transition of mitotic cell cycle, protein desumoylation and nuclear envelope, nuclear pore, nucleolus localization
NC_0060 35.2	W	94716 6	CAGL0L0 8646g	2890628	id476 3	XM_44915 2.1	G513 C	Met171 Ile	G	C	Ortholog(s) have SUMO-specific isopeptidase activity, role in G2/M transition of mitotic cell cycle, protein desumoylation and nuclear envelope, nuclear pore, nucleolus localization
NC_0060 35.2	W	13111 13	CAGL0L1 2188g	2890688	id492 4	XM_44929 8.1	A146 G	Asn49S er	A	G	Ortholog(s) have ATPase activity, DNA/DNA annealing activity, chromatin binding, double-stranded DNA binding, single-stranded DNA binding activity and role in mitotic chromosome condensation, rDNA condensation, tRNA gene clustering
NC_0060 35.2	W	13250 19	CAGL0L1 2232g	2890690	id492 6	XM_44930 0.1	G266 A	Ser89A sn	G	A	Ortholog(s) have role in attachment of GPI anchor to protein and GPI-anchor transamidase complex localization
NC_0060 35.2	W	13260 35	CAGL0L1 2232g	2890690	id492 6	XM_44930 0.1	G1282 T	Ala428 Ser	G	T	Ortholog(s) have role in attachment of GPI anchor to protein and GPI-anchor transamidase complex localization
NC_0060 35.2	W	13300 14	CAGL0L1 2276g	2890692	id492 8	XM_44930 2.1	G548 A	Arg183 Lys	G	A	Ortholog(s) have ATPase activator activity, role in protein import into mitochondrial matrix and integral component of mitochondrial outer membrane localization
NC_0060 35.2	W	13305 10	CAGL0L1 2276g	2890692	id492 8	XM_44930 2.1	A1044 T	Glu348 Asp	A	T	Ortholog(s) have ATPase activator activity, role in protein import into mitochondrial matrix and integral component of mitochondrial outer membrane localization
NC_0060 35.2	W	13604 31	CAGL0L1 2650g	2890604	id494 5	XM_44931 8.1	G347 A	Ser116 Asn	G	A	Ortholog(s) have RNA polymerase II carboxy-terminal domain kinase activity, cyclin-dependent protein serine/threonine kinase activity
NC_0060 36.2	W	74388 2	CAGL0M0 7337g	2891716	id532 2	XM_44966 7.1	T80A	Tyr27P he	T	A	Ortholog(s) have role in histone methylation, histone ubiquitination, premeiotic DNA replication, protein monoubiquitination and nucleus localization

NC_006036.2	W	1040100	CAGL0M10395g	2891363	id5463	XM_449799.1	C1241A	Ala414Asp	C	A	Protein of unknown function
NC_006036.2	W	1040658	CAGL0M10395g	2891363	id5463	XM_449799.1	A1799G	Asn600Ser	A	G	Protein of unknown function
NC_006036.2	W	1044813	CAGL0M10417g	2891364	id5464	XM_449800.1	T436C	Thr146Ala	T	C	Ortholog(s) have role in vesicle-mediated transport and fungal-type vacuole, vesicle localization
NC_006036.2	W	1138787	CAGL0M11550g	2891412	id5516	XM_449843.1	G1658T	Ala553Asp	G	T	Ortholog(s) have DNA replication origin binding, chromatin binding, single-stranded DNA binding activity

Voriconazole annotations

Gene ID	Entrez	description	KEGG_PATHWAY	GOTERM_BP_DIRECT	GOTERM_CC_DIRECT	GOTERM_MF_DIRECT
CAGL0A03432g	2886385	Ortholog(s) have DNA-dependent ATPase activity, Y-form DNA binding, four-way junction DNA binding, four-way junction helicase activity, ubiquitin protein ligase activity	NA	GO:0000209~protein polyubiquitination,GO:0006289~nucleotide-excision repair,GO:0006302~double-strand break repair,GO:0010994~free ubiquitin chain polymerization,GO:0042276~error-prone translesion synthesis,	GO:0000781~chromosome, telomeric region,GO:0000790~nuclear chromatin,GO:0005737~cytoplasm,GO:0035861~site of double-strand break,	GO:0000400~four-way junction DNA binding,GO:0000403~Y-form DNA binding,GO:0005524~ATP binding,GO:0008094~DNA-dependent ATPase activity,GO:0008270~zinc ion binding,GO:0009378~four-way junction helicase activity,
CAGL0B04477g	2886560	Ortholog(s) have cellular bud neck, plasma membrane localization	NA	NA	GO:0005886~plasma membrane,GO:0005935~cellular bud neck,GO:0016021~integral component of membrane,	NA
CAGL0B04455g	2886660	Ortholog(s) have L-glutamine transmembrane transporter activity, L-isoleucine transmembrane transporter activity, L-tyrosine transmembrane transporter activity and role in amino acid transmembrane export from vacuole	NA	NA	GO:0016021~integral component of membrane,	NA
CAGL0B02948g	2886719	Beta mannosyltransferase	NA	NA	NA	NA
CAGL0C03982g	2886873	Has domain(s) with predicted transferase activity, transferring glycosyl groups activity and role in protein glycosylation	NA	GO:0006486~protein glycosylation,	GO:0016021~integral component of membrane,	GO:0016757~transferase activity, transferring glycosyl groups,
CAGL0C03938g	2886892	Has domain(s) with predicted transferase activity, transferring glycosyl groups activity and role in protein glycosylation	NA	GO:0006486~protein glycosylation,	NA	GO:0016757~transferase activity, transferring glycosyl groups,
CAGL0C05005g	2886923	Ortholog(s) have protein kinase activity, role in meiotic spindle disassembly, mitotic cytokinesis, protein phosphorylation, regulation of exit from mitosis and cellular bud neck, spindle pole body localization	cgr04111:Cell cycle - yeast,cgr04113:Meiosis	GO:0000281~mitotic cytokinesis,GO:0007096~regulation of exit from mitosis,GO:0051229~meiotic	GO:0005816~spindle pole body,GO:0005935~cellular bud neck,	GO:0004672~protein kinase activity,GO:0005524~ATP binding,

			- yeast,	spindle disassembly,		
CAGL0D02794g	2887008	Protein of unknown function	NA	GO:0006612~protein targeting to membrane,GO:0009408~response to heat,GO:0045047~protein targeting to ER,	GO:0005789~endoplasmic reticulum membrane,GO:0016021~integral component of membrane,	GO:0008565~protein transporter activity,
CAGL0D03278g	2887020	Ortholog(s) have role in chaperone-mediated protein complex assembly, nuclear-transcribed mRNA catabolic process, non-stop decay, proteasome assembly and intracellular localization	NA	GO:0043248~proteasome assembly,GO:0051131~chaperone-mediated protein complex assembly,GO:0070481~nuclear-transcribed mRNA catabolic process, non-stop decay,	GO:0005622~intracellular,	NA
CAGL0D00572g	2887254	Ortholog(s) have Rab GTPase binding, Ras guanyl-nucleotide exchange factor activity, phosphatidylinositol binding activity	NA	GO:0016192~vesicle-mediated transport,GO:0032889~regulation of vacuole fusion, non-autophagic,GO:0034727~piecemeal microautophagy of nucleus,GO:0035542~regulation of SNARE complex assembly,GO:0042144~vacuole fusion, non-autophagic,	GO:0000329~fungal-type vacuole membrane,GO:0005634~nucleus,GO:0005829~cytosol,GO:0030897~HOPS complex,	GO:0017112~Rab guanyl-nucleotide exchange factor activity,GO:0035091~phosphatidylinositol binding,
CAGL0D05918g	2887255	Putative alcohol acetyltransferase involved in steroid detoxification; gene is upregulated in azole-resistant strain	NA	GO:0009636~response to toxic substance,GO:0034209~sterol acetylation,GO:1900619~acetate ester metabolic process,	GO:0030176~integral component of endoplasmic reticulum membrane,	GO:0004026~alcohol O-acetyltransferase activity,
CAGL0E02167g	2887280	Ortholog(s) have role in cell morphogenesis involved in conjugation with cellular fusion, posttranslational protein targeting to endoplasmic reticulum membrane, protein insertion into ER membrane	NA	GO:0000753~cell morphogenesis involved in conjugation with cellular fusion,GO:0006620~posttranslational protein targeting to membrane,GO:0045048~protein insertion into ER membrane,	GO:0005634~nucleus,GO:0005840~ribosome,GO:0072380~TRC complex,	NA
CAGL0E06116g	2887354	Ortholog(s) have role in positive regulation of transcription by RNA polymerase II and cytosol, nuclear chromatin localization	NA	GO:0000436~carbon catabolite activation of transcription from RNA polymerase II promoter,GO:0061416~regulation of transcription from RNA polymerase II promoter in	GO:0005634~nucleus,	GO:0043565~sequence-specific DNA binding,GO:0046872~metal ion binding,

				response to salt stress,		
CAGL0E05038g	2887378	Ortholog(s) have ATP-dependent 3'-5' DNA helicase activity, ATPase activity	NA	GO:0006281~DNA repair,GO:0006338~chromatin remodeling,GO:0006351~transcription, DNA-templated,GO:0006355~regulation of transcription, DNA-templated,GO:0016569~covalent chromatin modification,	GO:0031011~Ino80 complex,	GO:0003677~DNA binding,GO:0004386~helicase activity,GO:0005524~ATP binding,GO:0016887~ATPase activity,
CAGL0E02101g	2887400	Ortholog(s) have 5'-deoxyribose-5-phosphate lyase activity, ATP-dependent 3'-5' RNA helicase activity, polynucleotide adenylyltransferase activity	cgr03018:RNA degradation,	GO:0006284~base-excision repair,GO:0006400~tRNA modification,GO:0034475~U4 snRNA 3'-end processing,GO:0042138~meiotic DNA double-strand break formation,GO:0045910~negative regulation of DNA recombination,GO:0071031~nuclear mRNA surveillance of mRNA 3'-end processing,GO:0071035~nuclear polyadenylation-dependent rRNA catabolic process,GO:0071036~nuclear polyadenylation-dependent snoRNA catabolic process,GO:0071037~nuclear polyadenylation-dependent snRNA catabolic process,GO:0071038~nuclear polyadenylation-dependent tRNA catabolic process,GO:0071039~nuclear polyadenylation-dependent CUT catabolic process,GO:0071040~nuclear polyadenylation-dependent antisense transcript catabolic process,GO:0071042~nuclear polyadenylation-dependent	GO:0005730~nucleolus,GO:0005829~cytosol,GO:0031499~TRAMP complex,	GO:0004652~polynucleotide adenylyltransferase activity,GO:0051575~5'-deoxyribose-5-phosphate lyase activity,

				mRNA catabolic process,GO:0071044~histone mRNA catabolic process,GO:0071050~snoRNA polyadenylation,GO:0071051~polyadenylation-dependent snoRNA 3'-end processing,		
CAGL0E06006g	2887415	Ortholog(s) have role in cellular iron ion homeostasis and mitochondrion localization	NA	GO:0006879~cellular iron ion homeostasis,	GO:0005739~mitochondrion,GO:0016021~integral component of membrane,	GO:0008324~cation transmembrane transporter activity,
CAGL0E06028g	2887416	Ortholog(s) have dolichyl-phosphate beta-glucosyltransferase activity and role in dolichol-linked oligosaccharide biosynthetic process	cgr00510:N-Glycan biosynthesis,cgr01100:Metabolic pathways,	GO:0006487~protein N-linked glycosylation,	GO:0005789~endoplasmic reticulum membrane,GO:0016021~integral component of membrane,	GO:0004581~dolichyl-phosphate beta-glucosyltransferase activity,
CAGL0E05940g	2887441	Ortholog(s) have FAD transmembrane transporter activity, calcium channel activity	NA	NA	GO:0016021~integral component of membrane,	NA
CAGL0E05874g	2887443	Ortholog(s) have GTP binding, GTPase activity, U3 snoRNA binding activity	cgr03008:Ribosome biogenesis in eukaryotes,	GO:0006364~rRNA processing,GO:0042255~ribosome assembly,	GO:0005730~nucleolus,GO:0005739~mitochondrion,GO:0030686~90S preribosome,GO:0072686~mitotic spindle,	GO:0003924~GTPase activity,GO:0005524~ATP binding,GO:0005525~GTP binding,GO:0034511~U3 snoRNA binding,
CAGL0E05808g	2887447	Bifunctional enzyme of thiamine biosynthesis, with thiamine-phosphate pyrophosphorylase and 4-methyl-5-beta-hydroxyethylthiazole kinase activities; active as a homohexamer	cgr00730:Thiamine metabolism,cgr01100:Metabolic pathways,	GO:0009228~thiamine biosynthetic process,	GO:0005829~cytosol,	GO:0000166~nucleotide binding,GO:0004417~hydroxyethylthiazole kinase activity,GO:0004789~thiamine-phosphate diphosphorylase activity,GO:0046872~metal ion binding,
CAGL0F08811g	2887569	Ortholog(s) have RNA binding activity, role in mRNA splicing, via spliceosome and U1 snRNP, U2-type prespliceosome localization	NA	GO:0000398~mRNA splicing, via spliceosome,	GO:0005685~U1 snRNP,GO:0005737~cytoplasm,GO:0071004~U2-type prespliceosome,	GO:0003723~RNA binding,

CAGL0 F08833g	2887570	Putative adhesin-like protein	NA	GO:0001402~signal transduction involved in filamentous growth,GO:0006972~hyperosmotic response,GO:0007232~osmosensory signaling pathway via Sho1 osmosensor,GO:0030010~establishment of cell polarity,	GO:0005887~integral component of plasma membrane,GO:0030427~site of polarized growth,	GO:0005034~osmosensor activity,
CAGL0 F09009g	2887603	Ortholog(s) have role in snoRNA metabolic process	NA	GO:0016074~snoRNA metabolic process,	GO:0005634~nucleus,GO:0005737~cytoplasm,	NA
CAGL0 F08789g	2887777	Ortholog of S. cerevisiae : MCY1; Putative cysteine synthase	cgr00270:Cysteine and methionine metabolism,cgr00920:Sulfur metabolism,cgr01100:Metabolic pathways,cgr01110:Biosynthesis of secondary metabolites,cgr01130:Biosynthesis of antibiotics,cgr01200:Carbon metabolism,cgr01230:Biosynthesis of amino acids,	GO:0006535~cysteine biosynthetic process from serine,	NA	NA
CAGL0 F08943g	2887851	Ortholog(s) have role in mitochondrial translation and mitochondrion localization	NA	GO:0032543~mitochondrial translation,	GO:0005739~mitochondrion,	NA

CAGL0F03003g	2887960	Ortholog(s) have osmosensor activity and role in (1->3)-beta-D-glucan biosynthetic process, cellular bud site selection, fungal-type cell wall organization, hyperosmotic response, osmosensory signaling pathway via Sho1 osmosensor	NA	GO:0000282~cellular bud site selection,GO:0006075~(1->3)-beta-D-glucan biosynthetic process,GO:0006972~hyperosmotic response,GO:0007232~osmosensory signaling pathway via Sho1 osmosensor,GO:0031505~fungal-type cell wall organization,	GO:0005886~plasma membrane,GO:0016021~integral component of membrane,GO:0030427~site of polarized growth,	GO:0005034~osmosensor activity,
CAGL0G09361g	2888035	Ortholog(s) have structural molecule activity, role in acetyl-CoA biosynthetic process from pyruvate, filamentous growth, single-species biofilm formation on inanimate substrate and mitochondrial pyruvate dehydrogenase complex localization	NA	GO:0006086~acetyl-CoA biosynthetic process from pyruvate,	GO:0005967~mitochondrial pyruvate dehydrogenase complex,	GO:0005198~structural molecule activity,GO:0016746~transferase activity, transferring acyl groups,
CAGL0G10219g	2888103	Adhesin-like protein with 5 tandem repeats; belongs to adhesin cluster VII; predicted GPI-anchor; similarity to S. cerevisiae flocculins, cell wall proteins that mediate adhesion	NA	NA	GO:0009277~fungal-type cell wall,	NA
CAGL0G08602g	2888126	Ortholog(s) have GTPase regulator activity, role in Ras protein signal transduction, fungal-type cell wall biogenesis, positive regulation of transcription by RNA polymerase II and nucleus localization	NA	GO:0007265~Ras protein signal transduction,GO:0009272~fungal-type cell wall biogenesis,GO:0045944~positive regulation of transcription from RNA polymerase II promoter,GO:0050790~regulation of catalytic activity,	GO:0005634~nucleus,	NA
CAGL0G02959g	2888352	Ortholog of S. cerevisiae : NNF2; Protein that exhibits physical and genetic interactions with Rpb8p	NA	NA	GO:0016021~integral component of membrane,	NA
CAGL0G02827g	2888354	Ortholog(s) have phosphatidylinositol-4,5-bisphosphate binding, sphingolipid binding activity	NA	GO:0001558~regulation of cell growth,GO:0016197~endosomal transport,GO:0030950~establishment or maintenance of actin cytoskeleton polarity,GO:0038203~TORC2 signaling,GO:0051017~actin filament bundle assembly,GO:0070941~eisoso	GO:0005886~plasma membrane,GO:0031932~TORC2 complex,	GO:0005546~phosphatidylinositol-4,5-bisphosphate binding,GO:0046625~sphingolipid binding,

				me assembly,		
CAGL0H09394g	2888537	Protein of unknown function	NA	NA	NA	NA
CAGL0H04873g	2888854	Ortholog(s) have sequence-specific DNA binding activity and role in filamentous growth	NA	NA	NA	GO:0003676~nucleic acid binding,GO:0046872~metal ion binding,
CAGL0I09460g	2888931	Ortholog(s) have role in mismatch repair, proteasome regulatory particle assembly and cytosol, nucleus, proteasome regulatory particle, base subcomplex localization	NA	GO:0006298~mismatch repair,GO:0070682~proteasome regulatory particle assembly,	GO:0005634~nucleus,GO:0005829~cytosol,	NA
CAGL0I05104g	2889090	Has domain(s) with predicted ATP binding, aminoacyl-tRNA ligase activity, proline-tRNA ligase activity and role in prolyl-tRNA aminoacylation, tRNA aminoacylation for protein translation	cgr00970:Aminoacyl-tRNA biosynthesis,	GO:0006433~prolyl-tRNA aminoacylation,	GO:0005739~mitochondrion,	GO:0004827~proline-tRNA ligase activity,GO:0005524~ATP binding,
CAGL0I04004g	2889095	Protein of unknown function	NA	GO:0006813~potassium ion transport,GO:0015992~proton transport,	GO:0031305~integral component of mitochondrial inner membrane,	NA
CAGL0I06248g	2889154	Ortholog(s) have role in cellular cation homeostasis, cellular protein localization, protein dephosphorylation	NA	NA	NA	GO:0004674~protein serine/threonine kinase activity,GO:0005524~ATP binding,
CAGL0I07953g	2889235	Ortholog(s) have phosphatidylinositol-4,5-bisphosphate binding activity and role in establishment or maintenance of actin cytoskeleton polarity, fungal-type cell wall organization, regulation of cell growth	NA	GO:0001558~regulation of cell growth,GO:0030950~establishment or maintenance of actin cytoskeleton polarity,GO:0031505~fungal-type cell wall organization,	GO:0005737~cytoplasm,GO:0005886~plasma membrane,GO:0031932~TORC2 complex,	GO:0005546~phosphatidylinositol-4,5-bisphosphate binding,
CAGL0I10769g	2889399	Has domain(s) with predicted DNA binding, protein dimerization activity	cgr04011:MAPK signaling pathway - yeast,cgr04111:Cell cycle - yeast,	GO:0006351~transcription, DNA-templated,GO:0045944~positive regulation of transcription from RNA polymerase II promoter,	GO:0005634~nucleus,	GO:0000982~transcription factor activity, RNA polymerase II core promoter proximal region sequence-specific binding,GO:0000987~core promoter proximal region sequence-specific DNA binding,

CAGL0J06556g	2889543	Ortholog(s) have phosphatidylinositol-3-phosphate binding activity, role in mitochondrion inheritance, nucleus inheritance, vacuole-ER tethering and nucleus-vacuole junction localization	NA	GO:0000001~mitochondrion inheritance,	GO:0016021~integral component of membrane,	GO:0032266~phosphatidylinositol-3-phosphate binding,
CAGL0J02706g	2889752	Ortholog(s) have role in meiotic DNA double-strand break formation and condensed nuclear chromosome localization	NA	NA	NA	NA
CAGL0K00275g	2889973	Ortholog(s) have phospholipase activity, ubiquitin binding activity	cgr04141:Protein processing in endoplasmic reticulum,	GO:0006303~double-strand break repair via nonhomologous end joining,GO:0006511~ubiquitin-dependent protein catabolic process,GO:0010992~ubiquitin homeostasis,	GO:0005737~cytoplasm,	NA
CAGL0K04455g	2889998	Ortholog(s) have role in ascospore formation and ascospore wall, prospore membrane, septin complex localization	NA	GO:0030437~ascospore formation,	GO:0005619~ascospore wall,GO:0005628~prospore membrane,GO:0031105~septin complex,	GO:0005525~GTP binding,
CAGL0K01485g	2890059	Ortholog(s) have pseudouridine synthase activity, role in tRNA pseudouridine synthesis and mitochondrion localization	cgr00740:Riboflavin metabolism, cgr01100:Metabolic pathways,	GO:0031119~tRNA pseudouridine synthesis,	GO:0005739~mitochondrion,	GO:0003723~RNA binding,GO:0009982~pseudouridine synthase activity,
CAGL0K02805g	2890128	Predicted inositolphosphorylceramide (IPC) synthase, catalyzes the essential step in sphingolipid biosynthesis; potential antifungal drug target	NA	GO:0006673~inositolphosphoceramide metabolic process,	GO:0016021~integral component of membrane,GO:0070916~inositol phosphoceramide synthase complex,	GO:0045140~inositol phosphoceramide synthase activity,
CAGL0K00517g	2890183	Ortholog(s) have protein serine/threonine kinase activity	NA	GO:0010507~negative regulation of autophagy,GO:0030950~establishment or maintenance of actin cytoskeleton polarity,GO:0031929~TOR signaling,GO:0035025~positive regulation of Rho protein signal transduction,GO:0042254~ribosome	GO:0000329~fungal-type vacuole membrane,GO:0031234~extrinsic component of cytoplasmic side of plasma membrane,GO:0031931~TORC1 complex,GO:0031932~TORC2 complex,	GO:0004674~protein serine/threonine kinase activity,GO:0005524~ATP binding,GO:0044877~macromolecular complex binding,

				biogenesis,GO:0045807~positive regulation of endocytosis,GO:2001108~positive regulation of Rho guanyl-nucleotide exchange factor activity,		
CAGL0K00495g	2890185	Ortholog(s) have eukaryotic initiation factor 4E binding activity and role in deadenylation-dependent decapping of nuclear-transcribed mRNA, negative regulation of translation	NA	GO:0000290~deadenylation-dependent decapping of nuclear-transcribed mRNA,GO:0017148~negative regulation of translation,	GO:0005844~polysome, GO:0005845~mRNA cap binding complex,GO:0010494~cytoplasmic stress granule,	NA
CAGL0K00451g	2890187	Ortholog(s) have role in proteasome assembly and cytosol localization	NA	GO:0030433~ER-associated ubiquitin-dependent protein catabolic process,GO:0043248~proteasome assembly,	GO:0005829~cytosol,	NA
CAGL0K00429g	2890189	Has domain(s) with predicted nucleotide binding activity	NA	GO:0007064~mitotic sister chromatid cohesion,GO:0044376~RNA polymerase II complex import to nucleus,	GO:0005829~cytosol,	GO:0003924~GTPase activity,GO:0016887~ATPase activity,
CAGL0K00605g	2890231	Ortholog(s) have ATP binding, ATPase activity, DNA replication origin binding, GTP binding, GTPase activity, chromatin binding, protein serine/threonine kinase activity	cgr04111:Cell cycle - yeast,cgr04113:Meiosis - yeast,	GO:0000082~G1/S transition of mitotic cell cycle,GO:0006267~pre-replicative complex assembly involved in nuclear cell cycle DNA replication,GO:0006270~DNA replication initiation,GO:0031938~regulation of chromatin silencing at telomere,GO:0033314~mitotic DNA replication checkpoint,GO:0036289~peptidyl-serine autophosphorylation,GO:0051301~cell division,GO:1903468~positive regulation of DNA replication initiation,GO:1990443~peptidy	GO:0000790~nuclear chromatin,GO:0005656~nuclear pre-replicative complex,GO:0031261~DNA replication preinitiation complex,	GO:0003682~chromatin binding,GO:0003688~DNA replication origin binding,GO:0003924~GTPase activity,GO:0004674~protein serine/threonine kinase activity,GO:0005524~ATP binding,GO:0005525~GTP binding,GO:0016887~ATPase activity,

				l-threonine autophosphorylation,		
CAGL0K02761g	2890271	Ortholog(s) have ubiquitin binding activity	cgr04144:Endocytosis,	GO:0006623~protein targeting to vacuole,GO:0045324~late endosome to vacuole transport,	GO:0010008~endosome membrane,GO:0033565~ESCRT-0 complex,	NA
CAGL0K02783g	2890272	Ortholog(s) have actin filament binding activity	cgr04144:Endocytosis,	GO:0000281~mitotic cytokinesis,GO:0008360~regulation of cell shape,GO:0030036~actin cytoskeleton organization,GO:0034613~cellular protein localization,GO:0051016~barbed-end actin filament capping,	GO:0005884~actin filament,GO:0008290~F-actin capping protein complex,GO:0030479~actin cortical patch,GO:0032153~cell division site,GO:0043332~mating projection tip,GO:0051286~cell tip,	NA
CAGL0K05445g	2890486	Ortholog(s) have role in centromere complex assembly, establishment of protein localization to chromosome, kinetochore organization and maintenance of meiotic sister chromatid cohesion	cgr04113:Meiosis - yeast,	GO:0000070~mitotic sister chromatid segregation,GO:0007094~mitotic spindle assembly checkpoint,GO:0031134~sister chromatid biorientation,GO:0034090~maintenance of meiotic sister chromatid cohesion,GO:0034096~positive regulation of maintenance of meiotic sister chromatid cohesion,GO:0034508~centromere complex assembly,GO:0051301~cell division,GO:0051383~kinetochore organization,GO:0051757~meiotic sister chromatid separation,GO:0070199~establishment of protein localization to chromosome,	GO:0000778~condensed nuclear chromosome kinetochore,	NA

CAGL0K00693g	2890514	Ortholog(s) have protein tyrosine kinase activity	cgr04111:Cell cycle - yeast,cgr04113:Meiosis - yeast,	GO:0000079~regulation of cyclin-dependent protein serine/threonine kinase activity,GO:0000086~G2/M transition of mitotic cell cycle,GO:0000320~re-entry into mitotic cell cycle,GO:0010697~negative regulation of spindle pole body separation,GO:0036289~peptidyl-serine autophosphorylation,GO:0038083~peptidyl-tyrosine autophosphorylation,GO:0044387~negative regulation of protein kinase activity by regulation of protein phosphorylation,GO:0044878~mitotic cytokinesis checkpoint,GO:0045835~negative regulation of meiotic nuclear division,GO:0072453~signal transduction involved in G2 cell size control checkpoint,GO:1902402~signal transduction involved in mitotic DNA damage checkpoint,	GO:0005634~nucleus,GO:0005935~cellular bud neck,GO:0044732~mitotic spindle pole body,GO:0071341~medial cortical node,	GO:0004674~protein serine/threonine kinase activity,GO:0004712~protein serine/threonine/tyrosine kinase activity,GO:0004713~protein tyrosine kinase activity,GO:0005524~ATP binding,
CAGL0L12650g	2890604	Ortholog(s) have RNA polymerase II carboxy-terminal domain kinase activity, cyclin-dependent protein serine/threonine kinase activity	NA	GO:0000435~positive regulation of transcription from RNA polymerase II promoter by galactose,GO:0006351~transcription, DNA-templated,GO:0010971~positive regulation of G2/M transition of mitotic cell cycle,GO:0031648~protein destabilization,GO:0060258~negative regulation of filamentous growth,GO:0070481~nuclear-transcribed mRNA catabolic	GO:0016592~mediator complex,	GO:0004693~cyclin-dependent protein serine/threonine kinase activity,GO:0005524~ATP binding,GO:0008353~RNA polymerase II carboxy-terminal domain kinase activity,GO:0046872~metal ion binding,

				process, non-stop decay,GO:0070816~phosphorylation of RNA polymerase II C-terminal domain,GO:1900387~negative regulation of cell-cell adhesion by negative regulation of transcription from RNA polymerase II promoter,		
CAGL0L08580g	2890625	Putative 26S proteasome regulatory subunit; protein abundance decreased in ace2 mutant cells	cgr03050:Proteasome,	GO:0006511~ubiquitin-dependent protein catabolic process,	GO:0005634~nucleus,GO:0005829~cytosol,GO:0008540~proteasome regulatory particle, base subcomplex,	GO:0005198~structural molecule activity,
CAGL0L08646g	2890628	Ortholog(s) have SUMO-specific isopeptidase activity, role in G2/M transition of mitotic cell cycle, protein desumoylation and nuclear envelope, nuclear pore, nucleolus localization	NA	NA	NA	GO:0008234~cysteine-type peptidase activity,
CAGL0L04642g	2890647	Ortholog(s) have 1-acylglycerol-3-phosphate O-acyltransferase activity, 1-acylglycerophosphocholine O-acyltransferase activity, role in glycerophospholipid biosynthetic process and endoplasmic reticulum localization	cgr00561:Glycerolipid metabolism,cgr00564:Glycerophospholipid metabolism,cgr00565:Ether lipid metabolism,cgr01100:Metabolic pathways,cgr01110:Biosynthesis of secondary metabolites,	GO:0046474~glycerophospholipid biosynthetic process,	GO:0005783~endoplasmic reticulum,GO:0005840~ribosome,GO:0016021~integral component of membrane,	GO:0003841~1-acylglycerol-3-phosphate O-acyltransferase activity,GO:0047184~1-acylglycerophosphocholine O-acyltransferase activity,
CAGL0L06358g	2890651	Ortholog of S. cerevisiae : TMS1; Vacuolar membrane protein of unknown function	NA	NA	GO:0016021~integral component of membrane,	NA

CAGL0L06402g	2890653	Ortholog(s) have bilirubin transmembrane transporter activity, glutathione S-conjugate-exporting ATPase activity, phytochelatin transmembrane transporter ATPase activity	NA	GO:0006749~glutathione metabolic process,GO:0036246~phytochelatin 2 import into vacuole,GO:0042144~vacuole fusion, non-autophagic,GO:0045454~cell redox homeostasis,GO:0071996~glutathione transmembrane import into vacuole,GO:0098849~cellular detoxification of cadmium ion,	GO:0000329~fungal-type vacuole membrane,GO:0005794~Golgi apparatus,GO:0016021~integral component of membrane,	GO:0005524~ATP binding,GO:0015127~bilirubin transmembrane transporter activity,GO:0015431~glutathione S-conjugate-exporting ATPase activity,GO:0044604~phytochelatin transmembrane transporter ATPase activity,
CAGL0L01881g	2890673	Has domain(s) with predicted integral component of membrane localization	NA	NA	GO:0016021~integral component of membrane,	NA
CAGL0L12188g	2890688	Ortholog(s) have ATPase activity, DNA/DNA annealing activity, chromatin binding, double-stranded DNA binding, single-stranded DNA binding activity and role in mitotic chromosome condensation, rDNA condensation, tRNA gene clustering	cgr04111:Cell cycle - yeast,	GO:0006268~DNA unwinding involved in DNA replication,GO:0007076~mitotic chromosome condensation,GO:0070058~tRNA gene clustering,GO:0070550~rDNA condensation,	GO:0000799~nuclear condensin complex,	GO:0003682~chromatin binding,GO:0005524~ATP binding,
CAGL0L12232g	2890690	Ortholog(s) have role in attachment of GPI anchor to protein and GPI-anchor transamidase complex localization	cgr00563:Glycosylphosphatidylinositol(GPI)-anchor biosynthesis,cgr01100:Metabolic pathways,	GO:0016255~attachment of GPI anchor to protein,	GO:0042765~GPI-anchor transamidase complex,	GO:0003923~GPI-anchor transamidase activity,
CAGL0L12276g	2890692	Ortholog(s) have ATPase activator activity, role in protein import into mitochondrial matrix and integral component of mitochondrial outer membrane localization	NA	GO:0006457~protein folding,GO:0030150~protein import into mitochondrial matrix,	GO:0005634~nucleus,GO:0005783~endoplasmic reticulum,GO:0031307~integral component of mitochondrial outer membrane,	GO:0001671~ATPase activator activity,

CAGL0L04796g	2890707	Protein of unknown function	NA	GO:0033962~cytoplasmic mRNA processing body assembly,GO:0034063~stress granule assembly,GO:0045947~negative regulation of translational initiation,	GO:0000932~cytoplasmic mRNA processing body,GO:0010494~cytoplasmic stress granule,	GO:0003729~mRNA binding,
CAGL0L04818g	2890708	Ortholog of S. cerevisiae : YGR117C; Putative protein of unknown function	NA	NA	GO:0005737~cytoplasm,	NA
CAGL0L03289g	2890726	Ortholog(s) have role in autophagy of mitochondrion, fungal-type cell wall biogenesis, fungal-type cell wall organization	NA	GO:0000422~mitophagy,GO:0009272~fungal-type cell wall biogenesis,	GO:0009277~fungal-type cell wall,GO:0031314~extrinsic component of mitochondrial inner membrane,	NA
CAGL0L05852g	2890809	Ortholog(s) have structural constituent of nuclear pore activity	cgr03013:RNA transport,	GO:0000055~ribosomal large subunit export from nucleus,GO:0006409~tRNA export from nucleus,GO:0006606~protein import into nucleus,GO:0016973~poly(A)+ mRNA export from nucleus,	GO:0005737~cytoplasm, GO:0034399~nuclear periphery,GO:0044613~nuclear pore central transport channel,	GO:0005487~nucleocytoplasmic transporter activity,GO:0017056~structural constituent of nuclear pore,
CAGL0L05874g	2890810	Ortholog(s) have 5'-3' exoribonuclease activity, chromatin binding, magnesium ion binding, microtubule binding, recombinase activity	cgr03008:Ribosome biogenesis in eukaryotes, cgr03018:RNA degradation,	GO:0000184~nuclear-transcribed mRNA catabolic process, nonsense-mediated decay,GO:0007089~traversing start control point of mitotic cell cycle,GO:0032968~positive regulation of transcription elongation from RNA polymerase II promoter,GO:0060261~positive regulation of transcription initiation from RNA polymerase II promoter,GO:0070651~nonfunctional rRNA decay,	GO:0000932~cytoplasmic mRNA processing body,GO:0005634~nucleus,GO:0010494~cytoplasmic stress granule,	GO:0003682~chromatin binding,GO:0003723~RNA binding,GO:0004534~5'-3' exoribonuclease activity,

CAGL0 L05896g	2890811	Ortholog(s) have role in mRNA splicing, via spliceosome and RES complex, U2-type spliceosomal complex localization	NA	GO:0000282~cellular bud site selection,GO:0000398~mRNA splicing, via spliceosome,GO:0006406~mRNA export from nucleus,	GO:0005684~U2-type spliceosomal complex,GO:0005829~cytosol,GO:0070274~RES complex,	NA
CAGL0 L02585g	2890856	Has domain(s) with predicted DNA binding, chromatin binding activity	NA	GO:0000105~histidine biosynthetic process,GO:0006366~transcription from RNA polymerase II promoter,GO:0007131~reciprocal meiotic recombination,GO:0009113~purine nucleobase biosynthetic process,GO:0045944~positive regulation of transcription from RNA polymerase II promoter,	GO:0005634~nucleus,	GO:0000981~RNA polymerase II transcription factor activity, sequence-specific DNA binding,GO:0001046~core promoter sequence-specific DNA binding,GO:0001135~transcription factor activity, RNA polymerase II transcription factor recruiting,
CAGL0 L02607g	2890859	Has domain(s) with predicted hydrolase activity and role in nucleotide catabolic process	NA	GO:0009166~nucleotide catabolic process,	GO:0000324~fungal-type vacuole,GO:0005829~cytosol,	GO:0016787~hydrolase activity,
CAGL0 L02695g	2890861	Protein of unknown function	NA	GO:0000042~protein targeting to Golgi,GO:0006888~ER to Golgi vesicle-mediated transport,	GO:0000139~Golgi membrane,	NA
CAGL0 L01507g	2890878	Ortholog(s) have U2 snRNA binding activity, role in spliceosomal complex assembly and U2 snRNP, U2-type prespliceosome localization	cgr03040:Spliceosome,	GO:0000245~spliceosomal complex assembly,GO:0045292~mRNA cis splicing, via spliceosome,	GO:0005686~U2 snRNP,GO:0071004~U2-type prespliceosome,	GO:0030620~U2 snRNA binding,
CAGL0 M10395 g	2891363	Protein of unknown function	NA	NA	NA	NA
CAGL0 M10417 g	2891364	Ortholog(s) have role in vesicle-mediated transport and fungal-type vacuole, vesicle localization	NA	NA	GO:0016021~integral component of membrane,	NA

CAGL0M11550g	2891412	Ortholog(s) have DNA replication origin binding, chromatin binding, single-stranded DNA binding activity	cgr04111:Cell cycle - yeast,cgr04113:Meiosis - yeast,	GO:0000727~double-strand break repair via break-induced replication,GO:0006267~pre-replicative complex assembly involved in nuclear cell cycle DNA replication,GO:0006270~DNA replication initiation,GO:0031938~regulation of chromatin silencing at telomere,GO:1902977~mitotic DNA replication preinitiation complex assembly,	GO:0000790~nuclear chromatin,GO:0005656~nuclear pre-replicative complex,GO:0005829~cytosol,GO:0031261~DNA replication preinitiation complex,GO:0031298~replication fork protection complex,	GO:0003682~chromatin binding,GO:0003688~DNA replication origin binding,GO:0003697~single-stranded DNA binding,
CAGL0M07337g	2891716	Ortholog(s) have role in histone methylation, histone ubiquitination, premeiotic DNA replication, protein monoubiquitination and nucleus localization	NA	GO:0006351~transcription, DNA-templated,GO:0006355~regulation of transcription, DNA-templated,GO:0016569~covalent chromatin modification,	GO:0005634~nucleus,	NA
CAGL0L04832g	9488040	Protein of unknown function	NA	NA	NA	NA

Flucytosine

chromosome	strand	coordinate	Gene ID	Entrez		geneBankID	nucleotide_alleles	aminoacid_alleles	REF	ALLELE	Description
NC_006028.2	C	18226	CAGL0E00231g	2887315	id1016	XM_002999488.1	C2821G	Leu941Val	G	C	Putative adhesin-like protein; contains tandem repeats and a predicted GPI-anchor; belongs to adhesin cluster III
NC_006028.2	C	18183	CAGL0E00231g	2887315	id1016	XM_002999488.1	T2864A	Phe955Tyr	A	T	Putative adhesin-like protein; contains tandem repeats and a predicted GPI-anchor; belongs to adhesin cluster III
NC_006028.2	C	18177	CAGL0E00231g	2887315	id1016	XM_002999488.1	T2870C	Leu957Ser	A	G	Putative adhesin-like protein; contains tandem repeats and a predicted GPI-anchor; belongs to adhesin cluster III
NC_006028.2	C	18147	CAGL0E00231g	2887315	id1016	XM_002999488.1	A2900T	Asn967Ile	T	A	Putative adhesin-like protein; contains tandem repeats and a predicted GPI-anchor; belongs to adhesin cluster III
NC_006031.1	C	3140	CAGL0H00110g	2888690	id2215	XM_446806.1	A572G	Asn191Ser	T	C	Adhesin-like protein with internal repeats; predicted GPI-anchor; likely a C-terminal fragment of a single ORF with CAGL0H00132g; belongs to adhesin cluster V
NC_006031.1	W	1046978	CAGL0H10626g	2888521	id2701	XM_447260.1	G3036T	Glu1012Asp	G	T	Predicted cell wall adhesin with a role in adhesion; belongs to adhesin cluster III; predicted GPI anchor; contains tandem repeats
NC_006032.2	W	397413	CAGL0I04466g	2889052	id2900	XM_447443.1	T242C	Val81Ala	T	C	Ortholog(s) have protein phosphatase regulator activity
NC_006032.2	W	587671	CAGL0I06138g	2889376	id2981	XM_447517.1	A1799T	Lys600Ile	A	T	Ortholog(s) have cyclin-dependent protein serine/threonine kinase inhibitor activity and role in chemotropism, maintenance of protein location in nucleus, mitotic cell cycle G1 arrest in response to pheromone
NC_006033.2	C	165962	CAGL0J01774g	9488027	id3279	XM_002999521.	C6112G	His2038Asp	G	C	Putative adhesin-like protein; has glycine and serine rich repeats; belongs to adhesin cluster VI
NC_006033.2	C	490159	CAGL0J05159g	9488022	id3441	XM_002999525.1	G4178A	Ser1393Asn	C	T	Putative adhesin-like protein
NC_004691.1	W	293	CaglfMp02	807019	gene5422	XM_449759.1	G113A	Ser38Asn	G	A	Probable mitochondrial ribosomal protein of the small subunit, orthologous to S. cerevisiae VAR1

Flucytosine annotations

Gene ID	Entrez	description	KEGG_PATHWAY	GOTERM_BP_DIRECT	GOTERM_CC_DIRECT	GOTERM_MF_DIRECT
CAGL0E00231g	2887315	Putative adhesin-like protein; contains tandem repeats and a predicted GPI-anchor; belongs to adhesin cluster III	NA	NA	GO:0016021~integral component of membrane,	
CAGL0H10626g	2888521	Predicted cell wall adhesin with a role in adhesion; belongs to adhesin cluster III; predicted GPI anchor; contains tandem repeats	NA	NA	GO:0009277~fungal-type cell wall,	
CAGL0I04466g	2889052	Ortholog(s) have protein phosphatase regulator activity	NA	GO:0000903~regulation of cell shape during vegetative growth phase,GO:0006355~regulation of transcription, DNA-templated,GO:0007010~cytoskeleton organization,GO:0030837~negative regulation of actin filament polymerization,GO:0032880~regulation of protein localization,	GO:0000131~incipient cellular bud site,GO:0005934~cellular bud tip,GO:0005935~cellular bud neck,	GO:0008599~protein phosphatase type 1 regulator activity,
CAGL0I06138g	2889376	Ortholog(s) have cyclin-dependent protein serine/threonine kinase inhibitor activity and role in chemotropism, maintenance of protein location in nucleus, mitotic cell cycle G1 arrest in response to pheromone	NA	GO:0000750~pheromone-dependent signal transduction involved in conjugation with cellular fusion,GO:0000751~mitotic cell cycle arrest in response to pheromone,GO:0051457~maintenance of protein location in nucleus,	GO:0005634~nucleus,GO:0005737~cytoplasm,GO:0016020~membrane,GO:0043332~mating projection tip,	GO:0004861~cyclin-dependent protein serine/threonine kinase inhibitor activity,GO:0008270~zinc ion binding,
CaglfMp02	807019	Probable mitochondrial ribosomal protein of the small subunit, orthologous to S. cerevisiae VAR1	NA	GO:0032543~mitochondrial translation,	GO:0005763~mitochondrial small ribosomal subunit,	GO:0003735~structural constituent of ribosome,
CAGL0J01774g	9488027	Putative adhesin-like protein; has glycine and serine rich repeats; belongs to adhesin cluster VI	NA	NA	NA	NA
CAGL0J05159g	9488022	Putative adhesin-like protein	NA	NA	NA	NA
CAGL0H00110g	2888690	Adhesin-like protein with internal repeats; predicted GPI-anchor;	NA	NA	NA	NA

		likely a C-terminal fragment of a single ORF with CAGL0H00132g; belongs to adhesin cluster V				

Enrichments

antifungal	Category	Term	Count	%	PValue	Genes	List Total	Pop Hits	Pop Total	Fold Enrichment	Bonferroni	Benjamini	FDR
caspofungin	KEGG_PATHWAY	cgr04113:Meiosis - yeast	5	17.86	0.0009	2890450, 2886698, 2889678, 2886382, 2888562	10	107	1923	8.986	0.015	0.015	0.015

Back to the index
